# New patchoulol- and guaiane-type sesquiterpenoids from *Sanguisorba officinalis* with anti-melanogenic activity

**DOI:** 10.1007/s13659-026-00616-0

**Published:** 2026-05-12

**Authors:** Long-Long Wu, Jia-Rui Wu, Kai-Xian Chen, Liu-Qiang Zhang, Yi-Ming Li

**Affiliations:** 1 School of Pharmacy, Shanghai University of Traditional Chinese Medicine, Shanghai, 201203 People’s Republic of China; 2 Institute of Chinese Medicine Resources, Anhui College of Traditional Chinese Medicine, Wuhu, 241000 People’s Republic of China

**Keywords:** *Sanguisorba officinalis*, Patchoulol-type sesquiterpenoids, Guaiane-type sesquiterpenoids, Anti-melanogenic activity, Whitening agents

## Abstract

Twelve new sesquiterpenoids, sanguisorbaols A–L (**1–12**), along with twenty-five known compounds (**13–37**), were isolated from the roots of *Sanguisorba officinalis*. Structures were determined through comprehensive analysis of HRESIMS, 1D and 2D NMR spectroscopic data, ECD and NMR calculations, and X-ray crystallographic analysis. Among them, compounds **1–6** were identified as new patchoulol-type sesquiterpenoids bearing hydroxyl groups at different positions, the further structural elucidation revealed that compounds **5** and **6** are nor-patchoulol-type sesquiterpenoids due to the absence of the C-11 carbon atom. Compounds **7–11** were characterized as guaiane-type sesquiterpenoids incorporating a [5,7]-bicyclic framework, among which **7** and **8** constitute a pair of C-11 epimers. Notably, compound **12** possesses a rare bicyclo[4.3.1]decane core scaffold. All the patchoulol-type sesquiterpenoids reported here were obtained for the first time from the genus *Sanguisorba*, and a plausible biosynthetic pathway was proposed. Furthermore, the anti-melanogenic bioactivity of all isolates was assessed using IBMX-stimulated B16F10 melanoma cells. Compounds **3–5**, **8–10**, **12–14**, **17**, **19**, **21**, **22**, **25–27**, **29**, **31**, **32** and **34** demonstrated significant inhibitory activity against melanogenesis at concentrations ranging from 6.25 to 50 μM. These results highlighted the potential of *S. officinalis* as a natural anti-melanogenesis agent for skin-whitening or for the treatment of hyperpigmentation disorders.

**Graphical Abstract:**

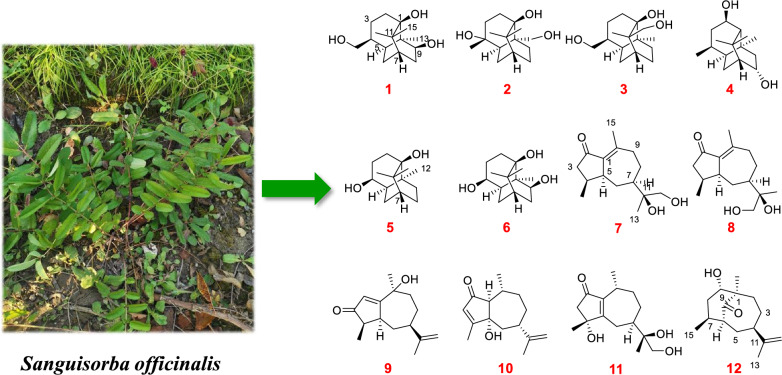

**Supplementary Information:**

The online version contains supplementary material available at 10.1007/s13659-026-00616-0.

## Highlights

- Twelve new sesquiterpenoids, along with twenty-five known sesquiterpenoids were isolated from the roots of *Sanguisorba officinalis*.
- Their structures were elucidated by NMR, HRESIMS, single crystal X-ray diffraction analysis, computational NMR and ECD spectra.
- Multiple sesquiterpenoids exhibited potent anti-melanogenic activities in IBMX-stimulated B16F10 melanoma cells.

**Supplementary Information:**

The online version contains supplementary material available at 10.1007/s13659-026-00616-0.

## Introduction

*Sanguisorba officinalis* L. (known as Di-Yu in Chinese) is a Rosaceae perennial herbaceous, distributed across Asia, Western Europe, and North America. Its roots are valued traditional Chinese medicine (TCM) material historically applied to arrest bleeding and heal ailments such as burns, scalds, enteritis, and leucopenia [1–3]. Previous phytochemical studies of *S. officinalis* have revealed a chemical profile rich in triterpenoids, tannins, phenols, flavonoids, and lignans [4–6]. Notably, ursane- and oleanane-type pentacyclic triterpenoids (2.4%–4.0% of dry root weight) along with hydrolysable and condensed tannins (approximately 17% of dry root weight) served as marker components for authenticating *S. officinalis*. Previous studies have demonstrated that these compounds possess diverse biological activities, for instance, hemostatic [7], anti-inflammatory [8–10], antioxidant [11], antitumor [12], antibacterial [13], antidiabetic [14], and antiviral [15] activities.

**Fig. 1:**
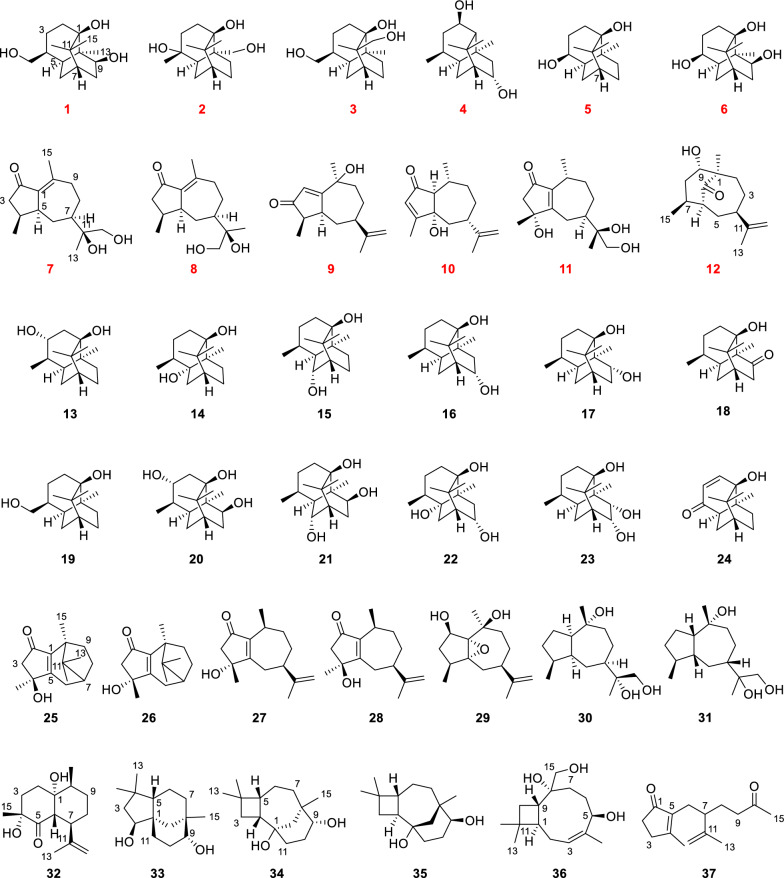
Compounds **1–37** isolated from the roots of *S. officinalis* and their chemical structures

The bioactivity of *S. officinalis* may involve not only its abundant triterpenoids but also other terpenoid constituents in the ethyl acetate extract. In a continued effort to discover bioactive terpenoids from this plant, twelve new (**1–12**) and twenty-five known (**13–37**) sesquiterpenoids were isolated from the ethyl acetate fraction of *S. officinalis* (Fig. 1). Their structures were elucidated through comprehensive spectroscopic analyses, including HRESIMS, NMR, X-ray diffraction, and quantum chemical calculations. *S. officinalis* has been widely used in dermatology due to its notable skin repair and whitening effects [16–19]. Our previous study has further revealed that sesquiterpenoids exhibited significant inhibitory effects on melanin production [20]. In this study, the anti-melanogenic potential of all isolated sesquiterpenoids was investigated employing 3-isobutyl-1-methylxanthine (IBMX)-stimulated B16F10 melanoma cells. The results indicated that twenty sesquiterpenoids markedly reduced melanin synthesis.

## Results and discussion

### Structural elucidation of isolated new sesquiterpenoids (1–12)

**Fig. 2:**
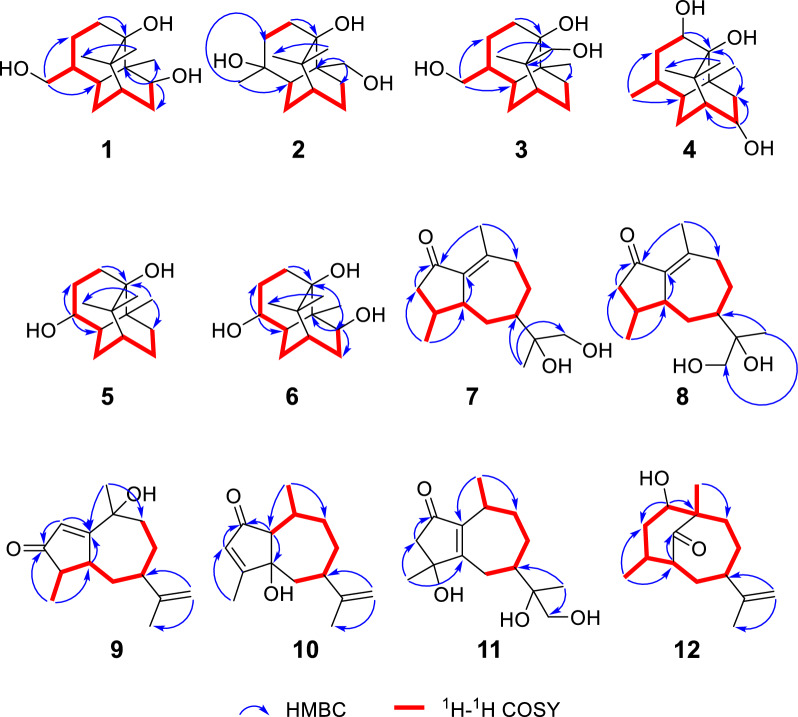
Key ^1^H–^1^H COSY and HMBC correlations of compounds **1–12**

**Fig. 3:**
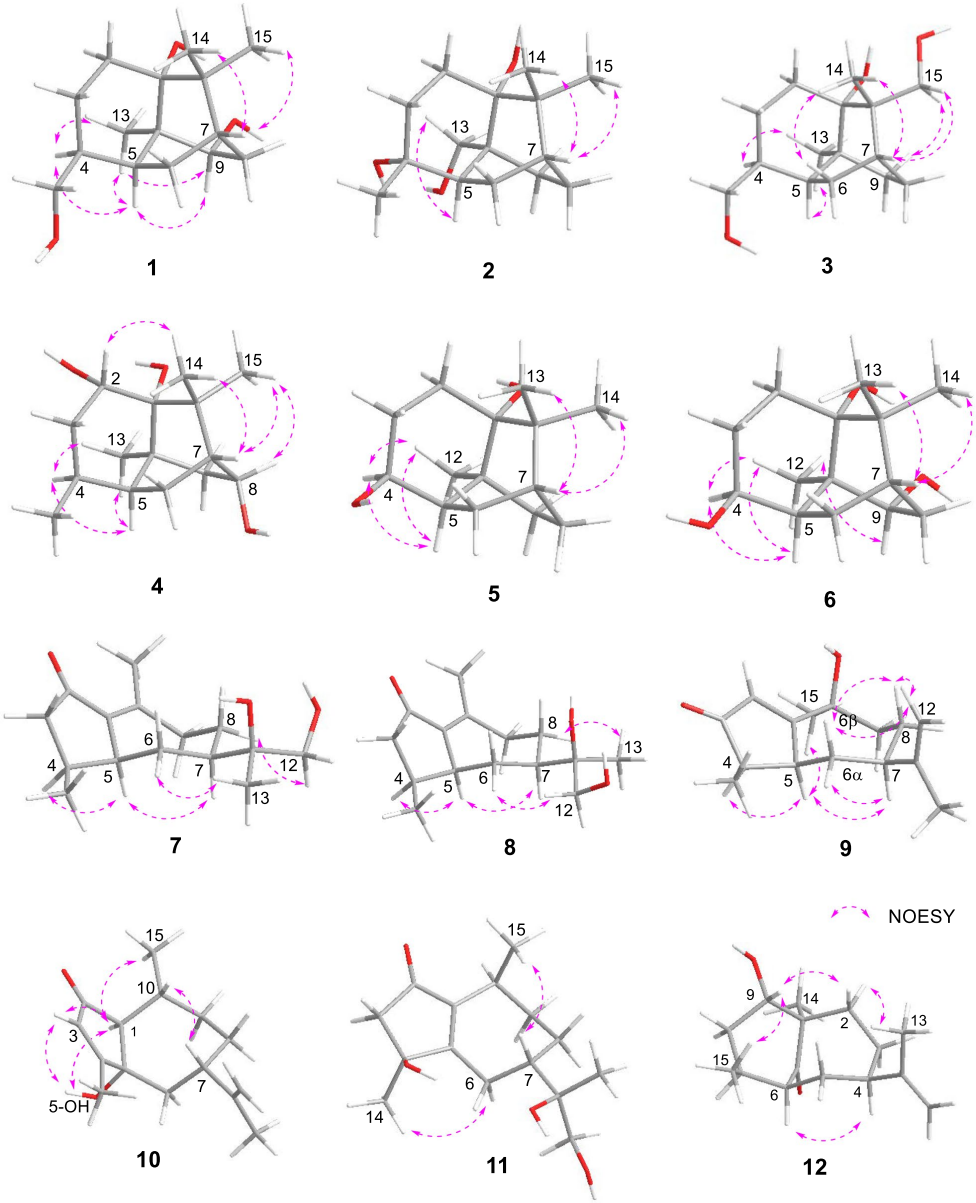
Key NOESY correlations of compounds **1–12**

**Fig. 4:**
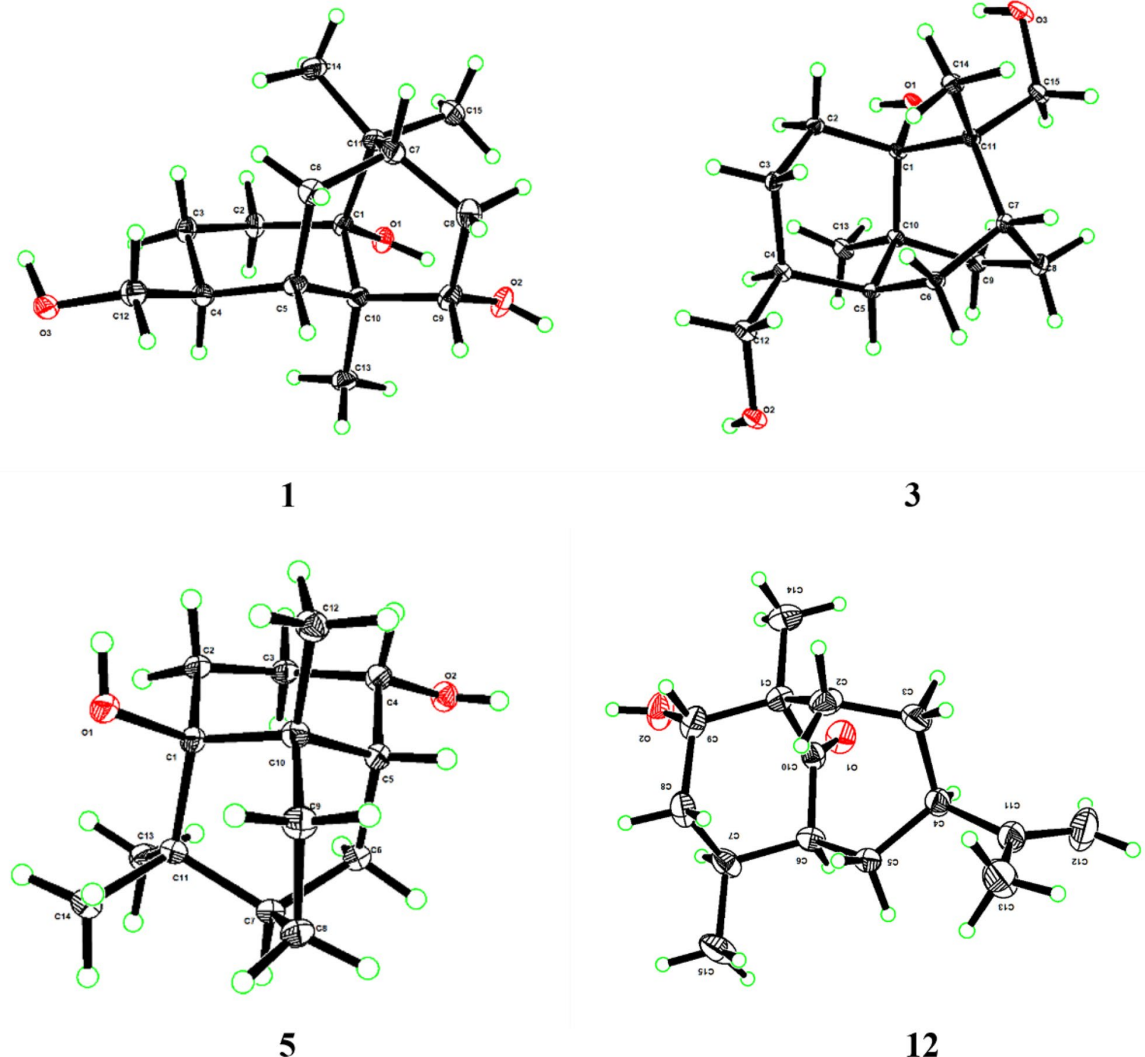
X‑ray crystal structures of compounds **1**, **3**, **5** and **12**

Compound **1** was isolated as colorless needle crystals (MeOH), assigned the molecular formula C_15_H_26_O_3_ according to the HRESIMS ion peak at *m/z* 277.1775 [M + Na]^+^ (calcd for C_15_H_26_O_3_Na, 277.1774), requiring an index of hydrogen deficiency (IHD) of three. The ^1^H NMR data showed an *O*-bearing methine at *δ*_H_ 3.56 (1H, brd, *J* = 8.9 Hz, H-9), a pair of oxygenated methylene at 3.38 (1H, dd, *J* = 10.7, 8.0 Hz, H-12a) and 3.35 (1H, dd, *J* = 10.7, 6.5 Hz, H-12b), three methyl singlets at *δ*_H_ 1.11 (3H, s, Me-13), 1.16 (3H, s, Me-14) and 1.17 (3H, s, Me-15), and multiple methylene and methine signals in high fields *δ*_H_ 1.22–2.13 (Table 1). In its ^13^C and DEPT-135 NMR spectra (Table 2), fifteen carbons were observed including three quaternary carbons containing an oxygenated carbon at *δ*_C_ 78.4 (C-1), four methine carbons with an *O*-bearing at *δ*_C_ 76.7 (C-9), five methylene carbons including an oxygenated one at *δ*_C_ 65.4 (C-12), as well as three methyl carbons at *δ*_C_ 18.2 (C-13), 25.1 (C-14), 27.5 (C-15). The above ^13^C NMR profile showed close agreement with that of the known patchoulol-type sesquiterpenoid, (9*R*)-9-hydroxypatchoulol [21]. Notably, compound **1** featured a hydroxymethyl group at C-4 [*δ*_C_ 65.4 (C-12), *δ*_H_ 3.38 (1H, dd, *J* = 10.7, 8.0 Hz, H-12a), 3.35 (1H, dd, *J* = 10.7, 6.5 Hz, H-12b)], whereas the known analogue (9*R*)-9-hydroxypatchoulol is substituted with a methyl group at C-12. The proposed structure of **1** was consistent with its molecular formula. Key HMBC correlations from H_2_-12 to C-3, C-4, and C-5 positioned the hydroxyl group at C-12 (Fig. 2). Furthermore, the contiguous proton spin system of H_2_-2/H_2_-3/H-4/H-5(H_2_-12)/H_2_-6/H-7/H_2_-8/H-9 observed in the ^1^H–^1^H COSY spectrum (Fig. 2), unequivocally confirmed the planar framework of **1**. The relative configuration of **1** was assigned based on NOESY correlations in conjunction with biosynthetic analogy. Within patchoulol-type sesquiterpenoids isolated from *Pogostemon cablin*, Me-13 commonly adopts *α*-oriented [21, 22]. The NOESY spectra (Fig. 3) of Me-13/H-4/H-5/H-9 supported the assignment H-4, H-5, and H-9 as *α*-oriented. Therefore, both of 9-OH and 12-CH_2_OH adopt a *β*-orientation. Finally, the absolute configuration of **1** was unambiguously defined as (1*R*,4*S*,5*S*,7*S*,9*S*,10*R*) [Cu-K*α* radiation, Flack parameter = 0.05(11), CCDC 2,333,284, Fig. 4] by the X-ray crystallographic analysis. Thus, compound **1** was identified as (1*R*,4*S*,5*S*,7*S*,9*S*,10*R*)-9,12-dihydroxypatchoulol, named sanguisorbaol A.

**Table 1:** ^1^H NMR data of compounds **1–12** in CD_3_OD (*δ* in ppm, *J* in Hz, measured at 600 MHz)

No	**1**	**2**	**3**	**4**	**5**	**6**	**7**	**8**	**9**	**10**	**11**	**12**
1	–	–	–	–	–	–	–	–	–	2.50 (m)	–	–
2	1.72 (dd, 12.4, 4.5) 1.61 (dd, 13.8, 6.7)	2.01 (m) 1.71 (m)	1.77 (dd, 12.7, 5.4) 1.60 (dd, 13.6, 6.4)	3.81 (t, 2.7)	1.78 (m) 1.61 (dd, 13.1, 6.2)	1.76 (m) 1.63 (dd, 13.6, 5.5)	–	–	6.15 (br s)	–	–	2.20 (td, 14.6, 3.0) 1.26 (ddd, 14.6, 5.7, 3.0)
3	1.57 (m) 1.32 (m)	1.98 (m) 1.51 (m)	1.57 (m) 1.30 (m)	1.76 (m) 1.71 (m)	1.77 (m) 1.70 (m)	1.80 (m) 1.67 (m)	2.42 (dd, 16.2, 7.2) 2.02 (dd, 16.2, 6.3)	2.42 (m) 2.04 (m)	–	5.88 (q,1.4)	2.49 (s)	1.75 (m) 1.47 (m)
4	2.11 (m)	–	2.03 overlapped	2.21 (m)	4.08 (ddd, 10.3, 6.2, 3.7)	4.16 (ddd, 10.4, 6.3, 4.2)	2.33 (m)	2.33 (m)	2.04 (dq, 7.4, 1.1)	–	–	2.23 (m)
5	1.55 (m)	1.78 (dd, 5.6, 2.8)	1.73 (ddd, 11.5, 5.9, 2.6)	1.48 (m)	1.81 (ddd, 13.5, 9.7, 6.2)	1.60 (m)	2.88 (t, 9.4)	2.89 (t, 9.4)	2.61 (ddt, 12.1, 2.6, 1.2)	–	–	2.02 (td, 13.4, 9.0) 1.45 (dt, 13.4, 9.4)
6	1.49 (ddd, 13.7, 5.5, 2.7) 1.21 (dt, 13.7, 3.3)	1.51 (m) 1.41 (m)	1.82 (m) 1.36 (m)	1.83 (m) 1.26 (m)	1.82 (m) 1.37 (m)	1.76 (m) 1.23 (m)	2.09 (m) 1.09 (m)	1.87 (m) 1.09 (m)	2.12 (m) 1.31 (m)	1.97 (dt, 14.4, 1.4) 1.84 (dd, 14.4, 10.6)	3.08 (dt, 15.6, 1.7) 2.24 (dd, 15.6,11.1)	2.51 (td, 9.4, 3.9)
7	1.29 (m)	1.19 (m)	1.27 (m)	1.30 (m)	1.21 (m)	1.32 (m)	1.83 (m)	1.83 (m)	2.24 (m)	2.54 (m)	1.63 overlapped	2.39 (m)
8	2.13 (m) 1.75 (ddd, 14.4, 9.8, 2.0)	1.93 (m) 1.27 (m)	1.52 (m) 1.34 (m)	4.15 (dd, 9.2, 4.4)	1.94 (m) 1.33 (m)	2.13 (m) 1.76 (m)	1.91 (m) 1.19 (m)	2.06 (m) 1.30 (m)	1.66 (m)	1.68 (m)	1.91 (m) 1.63 overlapped	2.35 (td, 13.1, 2.7) 1.63 (dt, 13.1, 2.7)
9	3.56 (br d, 8.9)	1.47 (m) 1.24 (m)	2.03 overlapped 1.09 (m)	2.34 (dd, 15.1, 9.2) 0.78 (d, 15.1)	1.90 (m) 1.06 (m)	3.54 (dd, 9.8, 1.5)	2.37 (m)	2.37 (m)	1.94 (m) 1.89 (m)	1.93 (m) 1.73 (m)	1.82 (m) 1.54 (m)	3.81 (t, 2.7)
10	–	–	–	–	–	–	–	–	–	2.45 (m)	2.90 (m)	–
11	–	–	–	–	–	–	–	–	–	–	–	–
12	3.38 (dd, 10.7, 8.0) 3.35 (dd, 10.7, 6.5)	1.17 (s)	3.38 (dd, 10.7, 8.0) 3.34 (dd, 10.7, 6.7)	0.84 (d, 6.7)	0.88 (s)	1.11 (s)	3.50 (d, 11.2) 3.44 (d, 11.2)	3.52 (d, 11.2) 3.43 (d, 11.2)	4.71 (d, 1.8) 4.66 (d, 1.8)	4.74 (d, 1.8) 4.67 (d, 1.8)	3.57 (d, 11.4) 3.47 (d, 11.4)	4.63 (br s) 4.58 (br s)
13	1.11 (s)	4.00 (d, 11.4) 3.50 (d, 11.4)	0.87 (s)	1.02 (s)	1.14 (s)	1.20 (s)	1.06 (s)	1.06 (s)	1.71 (br s)	1.77 (br s)	1.13 (s)	1.68 (br s)
14	1.16 (s)	1.07 (s)	1.18 (s)	1.07 (s)	1.08 (s)	1.17 (s)	0.98 (d, 6.9)	0.96 (d, 6.9)	1.15 (d, 7.4)	2.08 (d, 1.4)	1.39 (s)	1.03 (s)
15	1.17 (s)	1.09 (s)	3.95 (d, 10.7) 3.36 (d, 10.7)	1.06 (s)	–	–	2.23 (d, 2.1)	2.23 (d, 2.1)	1.38 (s)	0.83 (d, 7.4)	1.00 (d, 7.2)	1.00 (d, 6.7)

**Table 2:** ^13^C NMR data of compounds **1–12** in CD_3_OD (*δ* in ppm, measured at 150 MHz)

No	**1**	**2**	**3**	**4**	**5**	**6**	**7**	**8**	**9**	**10**	**11**	**12**
1	78.4	77.3	78.1	75.8	76.3	77.6	138.2	137.6	194.6	65.1	146.0	54.3
2	31.9	30.5	33.4	72.2	31.9	31.6	209.9	209.9	128.0	212.0	207.5	34.0
3	23.6	35.1	24.4	36.8	29.5	28.9	48.8	49.1	214.7	130.6	51.7	27.7
4	36.9	74.2	37.5	25.7	68.4	67.9	33.2	33.3	52.7	183.3	76.9	41.1
5	37.4	44.8	39.6	43.6	45.7	42.9	46.4	46.4	52.0	82.8	177.8	26.7
6	24.8	30.4	24.4	16.4	25.3	24.7	29.0	30.2	43.9	41.4	28.2	55.9
7	40.0	40.6	36.5	47.1	40.2	39.8	49.1	48.8	50.8	43.1	46.1	32.7
8	36.1	24.9	25.5	66.5	25.4	36.3	27.5	26.4	29.5	31.9	27.3	34.1
9	76.7	25.6	30.2	41.9	30.1	76.4	38.8	38.7	41.4	35.5	33.7	78.5
10	40.9	42.3	38.2	39.6	38.8	41.3	157.4	157.8	75.2	33.9	28.4	220.2
11	41.6	41.8	44.9	40.9	41.4	41.6	76.1	76.1	151.9	153.2	76.1	152.4
12	65.4	28.9	65.3	18.7	21.3	18.0	68.9	69.0	109.6	109.0	68.7	109.0
13	18.2	68.8	20.9	22.1	24.7	25.1	20.9	20.7	20.6	20.7	20.4	19.5
14	25.1	27.5	18.9	25.4	27.7	27.5	16.3	16.3	17.0	13.8	26.4	20.8
15	27.5	24.5	70.8	26.7	–	–	21.7	21.8	32.1	14.3	17.5	17.9

**Fig. 5:**
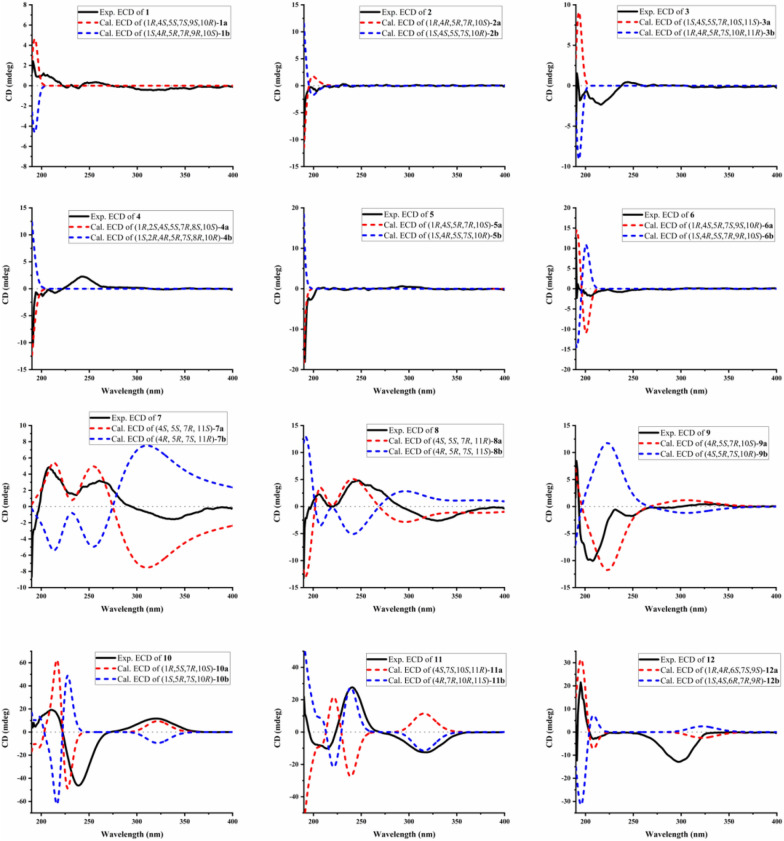
The experimental and calculated ECD spectra of compounds **1–12**

**Fig. 6:**
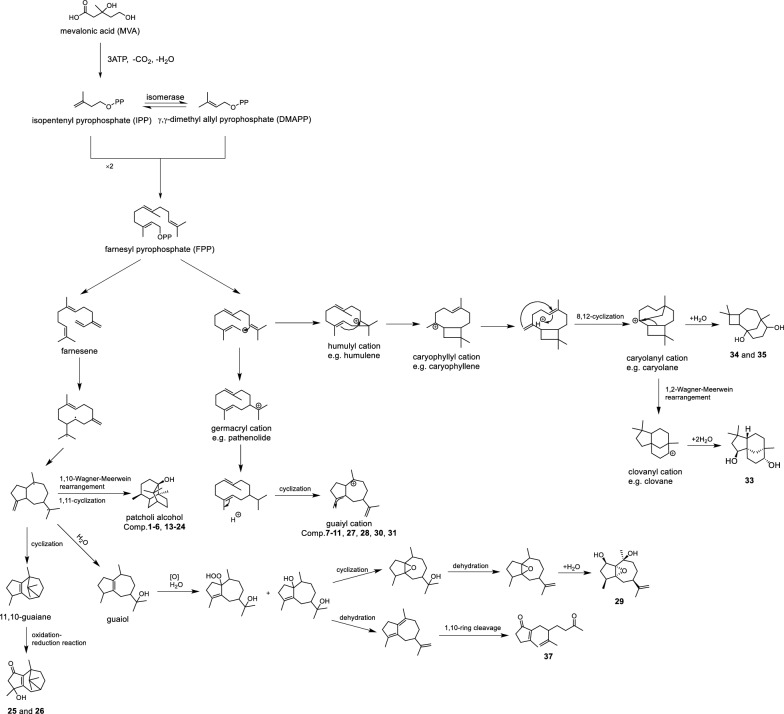
Two hypothetical biosynthetic pathways of patchoulol- and guaiane-type sesquiterpenoids in *S. officinalis* (radical mechanisms on the left and ionic mechanisms on the right)

Obtained as a colorless oil, compound **2** exhibited the same molecular formula as **1**, as evidenced by its HRESIMS peak at *m/z* 277.1775 [M + Na]^+^ (calcd for C_15_H_26_O_3_Na, 277.1774). This formula corresponded to three IHD. The ^1^H NMR data showed a pair of oxidized methylene protons at *δ*_H_ 4.00 (1H, d, *J* = 11.4 Hz, H-13a) and 3.50 (1H, d, *J* = 11.4 Hz, H-13b), three methyls at *δ*_H_ 1.17 (3H, s, Me-12), 1.07 (3H, s, Me-14) and 1.09 (3H, s, Me-15), as well as multiple methylene and methine signals in high fields *δ*_H_ 1.19–2.01 (Table 1). The 15 carbons signals in the ^13^C NMR spectrum and HSQC of **2** were classified as four fully substituted carbons atoms [including two oxygenated at *δ*_C_ 77.3 (C-1) and 74.2 (C-4)], two methine, six methylene [including one oxygenated at *δ*_C_ 68.8 (C-13)] and three methyls [*δ*_C_ 28.9 (C-12), 27.5 (C-14), 24.5 (C-15)] (Table 2). Comparative 1D NMR spectra revealed that **2** was structurally analogous to (4R*)-4 hydroxypatchoulol [22]. The principal distinction between **2** and the known analogue lies in the substitution of a methyl group by an oxygenated methylene at *δ*_C_ 68.8. The assignment of the hydroxymethyl at C-10 was corroborated by HMBC analysis of *δ*_H_ 4.00 (1H, d, *J* = 11.4 Hz, H-13a), 3.50 (1H, d, *J* = 11.4 Hz, H-13b) to *δ*_C_ 77.3 (C-1), 44.8 (C-5), 25.6 (C-9) and 42.3 (C-10) (Fig. 2). The relative configuration of **2** was established by virtue of NOESY correlation (Fig. 3). The observed correlation between H-5 and H_2_-13 indicated that both are *α*-oriented, whereas no correlative signal was detected between H_2_-13 and Me-12 suggested a *β*-orientation for Me-12. According to the calculated ECD spectral data (Fig. 5) together with the consistent biosynthetic pathway (Fig. 6), the absolute configuration of **2** was determined to be 1*R*,4*R*,5*R*,7*R*,10*S*. Consequently, the structure of **2** was established and designated sanguisorbaol B.

Colorless needle crystals of compound **3** were isolated from MeOH, and its molecular formula C_15_H_26_O_3_ was corroborated by the quasi-molecular ion peak at *m/z* 277.1773 [M + Na]^+^ (calcd for C_15_H_26_O_3_Na, 277.1774) in HREIMS. The ^1^H NMR data (Table 1) displayed two pairs of *O*-bearing methylene protons at *δ*_H_ 3.38 (1H, dd, *J* = 10.7, 8.0 Hz, H-12a) and 3.34 (1H, dd, *J* = 10.7, 6.7 Hz, H-12b), 3.95 (1H, d, *J* = 10.7 Hz, H-15a) and 3.36 (1H, d, *J* = 10.7 Hz, H-15b), two methyl singlets at *δ*_H_ 0.87 (3H, s, Me-13) and 1.18 (3H, s, Me-14), and multiple methylene and methine signals in high fields *δ*_H_ 1.09–2.03. Its ^13^C NMR spectrum displayed 15 carbons combined with DEPT-135 corresponding to three quaternary carbon atoms, three methine carbons, seven methylene, and two methyls. Among them, *δ*_C_ 78.1 (C-1), 65.3 (C-12), 70.8 (C-15) were three oxygenated carbons. The ^1^H and ^13^C NMR spectra of compound **3** closely resembled those of the known compound **19** [23], with the exception of the characteristic substitution of a singlet methyl (C-12) by a hydroxymethyl group in **3** (Tables 1 and 2). Key HMBC corrections from H_2_-12 (*δ*_H_ 3.38 and 3.35) to C-3 (*δ*_C_ 24.4), C-4 (*δ*_C_ 37.5) and C-5 (*δ*_C_ 39.6), and H_2_-15 (*δ*_H_ 3.95 and 3.36) to C-1 (*δ*_C_ 78.1), C-7 (*δ*_C_ 36.5), C-11 (*δ*_C_ 44.9) and C-14 (*δ*_C_ 18.9), could confirm that two hydroxyl groups were connected at C-12 and C-15, respectively (Fig. 2). NOESY correlations (Fig. 3) of H-7/Me-14/H_2_-15 and Me-13/H-4/H-5 revealed H-4, H-5 and Me-13 were *α*-oriented, while H-7 and H_2_-12 were *β*-oriented, consistent with the commonly observed in patchoulol-type sesquiterpenoids. In addition, NOESY correlations of Me-14 (*δ*_H_ 1.17) and H-6*β* (*δ*_H_ 1.36), H_2_-15 (*δ*_H_ 3.95 and 3.36) and H-9*β* (*δ*_H_ 2.03) demonstrated that Me-14 and C-6 were situated on the same face, while CH_2_-15 and C-9 were located on the opposite face. Finally, direct verification via X-ray crystallography established the absolute configuration of compound **3** as (1*S*,4*S*,5*S*,7*R*,10*S*,11*S*) [Cu K*α* radiation, Flack parameter = 0.10(11), CCDC 2,333,293, Fig. 4]. Hence, the structure of **3** was finally determined and named as sanguisorbaol C.

Compound **4** was isolated as a colorless oil, possessing the molecular formula C_15_H_26_O_3_ relying on the peak signal at *m/z* 237.1833 [M − H_2_O + H]^+^ (calcd for C_15_H_25_O_2_, 237.1849) from HRESIMS, with an IHD of three. By meticulously comparing the 1D NMR spectroscopic data of **4** with those of 3*α*,8*α*-dihydroxypathoulol [21], the two compounds were found to be structurally analogous (Tables 1 and 2). The major differences between them lie in the down-field shift of C-2 (*δ*_C_ 72.2 vs. 42.7) and the up-field shifts of C-3 (*δ*_C_ 36.8 vs. 72.9) and C-4 (*δ*_C_ 25.7 vs. 37.7), suggesting the transfer of the hydroxyl group from C-3 to C-2 in **4**, as further verified via the HMBC corrections from H-2 (*δ*_H_ 3.81) to C-1 (*δ*_C_ 75.8), C-3 (*δ*_C_ 36.8), C-4 (*δ*_C_ 25.7), C-10 (*δ*_C_ 39.6) and C-11 (*δ*_C_ 40.9) (Fig. 2). In addation, the correlations (Fig. 2) of H-2/H_2_-3/H-4/H-5(Me-12)/H_2_-6/H-7/H-8/H_2_-9 in the ^1^H–^1^H COSY spectrum unambiguously confirmed the planar framework of **4**. Correlations of H-5/H-4/Me-13 in the NOESY spectrum (Fig. 3) suggested these protons were cofacial, and were assigned an *α*-orientation. In contrast, NOESY interactions among H-7/H-8/Me-14/Me-15 and H-2/Me-14 suggested that these protons were *β*-orientation, and the 2-OH and 8-OH in **4** were *α*-orientation. Finally, the absolute configuration of **4** was established as 1*R*,2*S*,4*S*,5*S*,7*R*,8*S*,10*S*, based on the shared biosynthetic pathway (Fig. 6) and supporting evidence from ECD calculations (Fig. 5). Thus, the compound **4** was determined to be (1*R*,2*S*,4*S*,5*S*,7*R*,8*S*,10*S*)-2,8-dihydroxypatchoulol and named trivially as sanguisorbaol D.

Colorless needle-like crystals of compound **5** were purified from EtOH, possessing the molecular formula C_14_H_24_O_2_ according to the HRESIMS ion peak at *m/z* 207.1742 [M − H_2_O + H]^+^ (calcd for C_14_H_23_O, 207.1743), and corresponding to an IHD of three. Analysis of its ^1^H NMR spectrum (Table 1) exhibited an oxygenated methine signal at *δ*_H_ 4.08 (1H, ddd, *J* = 10.3, 6.2, 3.7 Hz, H-4), and three methyls at *δ*_H_ 0.88 (3H, s, C-12), 1.14 (3H, s, C-13) and 1.08 (3H, s, C-14). In combination with the DEPT spectrum, the ^13^C NMR data (Table 2) revealed 14 carbon resonances, which included three quaternary carbons [one oxygenated at *δ*_C_ 76.3 (C-1)], three methine carbons [one oxygenated at *δ*_C_ 68.4 (C-4)], five methylenes and three methyls [*δ*_C_ 21.3 (C-12), 24.7 (C-13), 27.7 (C-14)]. The above 1D NMR data showed some features analogous to those of **24** [24], apart from the substitution of the *α*,*β*-unsaturated ketone moiety by two methylenes and one oxygenated methine group in **5**. The above analyses suggested that compound **5** belongs to the *nor*-patchoulol-type sesquiterpenoid skeleton. The key HMBC correlations from H-4 to C-2, C-3, C-5, C-6 and C-10 additionally confirmed the absence of -CH_3_ and the attachment of a hydroxyl group at C-4 (Fig. 2), with the assistance of ^1^H–^1^H COSY correlations of H_2_-2/H_2_-3/H-4/H-5/H_2_-6/H-7/H_2_-8/H_2_-9. The NOESY spectrum of Me-12/H-4/H-5 (Fig. 3) implied that H-4, H-5 along with Me-12 were *α*-orientations, and 4-OH was *β*-orientation. In the X-ray diffraction experiment [Cu-K*α* radiation, Flack parameter = 0.00(8), CCDC 2,333,300], the (1*R*,4*S*,5*R*,7*R*,10*S*) absolute configuration was established as **5** (Fig. 4). Thus, the structure of **5** was determined as (1*R*,4*S*,5*R*,7*R*,10*S*)-4-hydroxy-*nor*-patchoulol and designated as sanguisorbaol E.

Compound **6** featured the molecular formula of C_14_H_24_O_3_ (IHD of three) determined by HRESIMS at *m/z* 263.1617 [M + Na]^+^ (calcd for C_14_H_24_O_3_Na, 263.1618). Thorough investigation of the ^1^H and ^13^C NMR of **6** indicated that it had a structural similarity to **5**, apart from an extra hydroxyl group in **6** (Tables 1 and 2), which was in line with the increase in molecular weight of **6** by 16-units. The key HMBC cross-peaks of H-9 with C-1, C-5, C-7, C-8, C-10 and C-12, as well as the ^1^H–^1^H COSY correlations of H_2_-2/H_2_-3/H-4/H-5/H_2_-6/H-7/H_2_-8/H-9, located the other hydroxyl group at C-9 (Fig. 2). In the NOESY spectrum (Fig. 3), the correlation from H-9 to H-5 and Me-12 suggested the 9-OH was *β*-orientation. Guided by the shared biosynthetic pathway (Fig. 6) and supported by ECD calculations (Fig. 5), the absolute configuration of **6** was elucidated as 1*R*,4*S*,5*R*,7*S*,9*S*,10*R*. In consequence, the structure was characterized as (1*R*,4*S*,5*R*,7*S*,9*S*,10*R*)-4,9-dihydroxy-*nor*-patchoulol and designated as sanguisorbaol F.

Compounds **7** and** 8** were both colorless oils. Their molecular formulas were determined using HRESIMS to be C_15_H_24_O_3_ (IHD of four) at *m/z* 253.1798, and 253.1800 ([M + H]^+^ calcd for C_15_H_25_O_3_, 253.1798), respectively. The ^1^H NMR signals (Table 1) of **7** displayed a pair of oxygenated methylene signals at *δ*_H_ 3.50 (1H, d, *J* = 11.2 Hz, H-12a) and 3.44 (1H, d, *J* = 11.2 Hz, H-12b), three methyl signals at *δ*_H_ 1.06 (3H, s, H-13), 0.98 (3H, d, *J* = 6.9 Hz, H-14) and 2.23 (3H, d, *J* = 2.1 Hz, H-15). Its ^13^C NMR (Table 2) together with DEPT data showed 15 carbon resonances attributable to a carbonyl quaternary carbon at *δ*_C_ 209.9 (C-2), two olefinic carbons at *δ*_C_ 138.2 (C-1) and 157.4 (C-10), an oxygenated quaternary carbon at *δ*_C_ 76.1 (C-11), three methines, five methylenes containing an *O*-bearing one at *δ*_C_ 68.9 (C-12), and three methyls at *δ*_C_ 20.9 (C-13), 16.3 (C-14) and 21.7 (C-15). Detailed examination of 1D NMR data showed that the framework of **7** was close to the known guaiane-type sesquiterpenoid xylaguaianol D [25], only differing by incorporation of a carbonyl quaternary carbon (*δ*_C_ 209.9) in **7** instead of one methylene carbon in xylaguaianol D. The placement of the carbonyl at C-2 was corroborated by the HMBC correlations (Fig. 2) of H_2_-3 with C-2, C-4, C-5 and C-14, of Me-15 with C-1, C-2, C-9 and C-10. Besides, key HMBC correlations of Me-13 with C-7, C-11 and C-12 and ^1^H–^1^H COSY correlations of H_2_-3/H-4/H-5(H_3_-14)/H_2_-6/H-7/H_2_-8/H_2_-9 (Fig. 2) unequivocally confirmed the planar structure of **7**. Correlations of H-4/H-5/H-6*α* (*δ*_H_ 2.09)/H-7 and Me-14/H-6*β* (*δ*_H_ 1.09)/Me-13 in the NOESY spectrum (Fig. 3) revealed the coplanarity of H-4, H-5 and H-7, which were arbitrarily designated as having an *α*-orientation, while Me-15 exhibited a *β*-orientation.

The planar structure of **8** was found to closely resemble that of **7** through comparing the ^1^H and ^13^C NMR spectra. By careful comparison of the ^13^C NMR (Table 2) signals between **7** and **8**, subtle differences were observed at C-6 (*δ*_C_ 29.0 for **7**, *δ*_C_ 30.2 for **8**) and C-8 (*δ*_C_ 27.5 for **7**, *δ*_C_ 26.4 for **8**), implying that **7** and **8** formed a pair of *R*/*S* epimers at C-11 position. Key NOESY correlations of H-12/H-8 and Me-13/H-6 in **7** implied that C-12 and C-8 were on the same side, and Me-13 and C-6 on the other side. On the contrary, the NOESY correlations of H-12/H-6 and Me-13/H-8 in **8** implied that C-12 and C-6 were on the same side, and Me-13 and C-8 on the other side (Fig. 3). This enabled us to distinguish the 11S* configuration for **7** and the 11R* for **8** by assuming 7*R*-configuration for both compounds. Subsequently, four molecular models (4*S*,5*S*,7*R*,11*S*)-**7a**, (4*R*,5*R*,7*S*,11*R*)-**7b**, (4*S*,5*S*,7*R*,11*R*)-**8a**, and (4*R*,5*R*,7*S*,11*S*)-**8b** were established and ECD calculations were performed. Subsequently, compared with experimental ECD spectra, it was found that compounds **7** and **8** matched well with (4*S*,5*S*,7*R*,11*S*)-**7a** and (4*S*,5*S*,7*R*,11*R*)-**8a**. Based on the excellent consistency between the calculated ECD spectrum and the experimental one (Fig. 5), the absolute configuration of compounds **7** and **8** were ascertained to be (4*S*,5*S*,7*R*,11*S*) and (4*S*,5*S*,7*R*,11*R*), respectively. Accordingly, the complete structures of compound **7** and **8** were fully identified as (4*S*,5*S*,7*R*,11*S*)-4*α*,5*α*,7*α*(H)-2-oxo-1(10)-guaiaene-11,12-diol and (4*S*,5*S*,7*R*,11*R*)-4*α*,5*α*,7*α*(H)-2-oxo-1(10)-guaiaene-11,12-diol, and named as sanguisorbaol G and sanguisorbaol H, respectively.

Compound **9** was afforded as colorless oil with the molecular formula C_15_H_22_O_2_ deduced from HRESIMS at *m/z* 235.1696 [M + H]^+^ (calcd for *m/z* C_15_H_23_O_2_, 235.1693), implying an IHD of five. Detailed analysis of NMR spectrum (Tables 1, 2 and Fig. 2) demonstrated the structure of **9** closely parallels that of stelleranoid L [26], differing only in the relative configuration at C-5. Key NOESY correlations between H-5 and H-4, H-7 and Me-15 revealed that H-4, H-5, H-7 and Me-15 were placed on the same side of the [5, 7] bicyclic guaiane-type sesquiterpenoid and assigned as *α*-orientation, while the Me-14, isopropenyl-7 and 10-OH were *β*-orientation in **9** (Fig. 3). Ultimately, the absolute configuration of **9** was ascertained as (4*R*,5*S*,7*R*,10*S*) via analysis of theoretical and experimental ECD curves (Fig. 5), and **9** was thus trivially named as sanguisorbaol I.

Compound **10**, a colorless oil, had its molecular formula determined to be C_15_H_22_O_2_ supported by the ion peak at *m/z* 235.1692 [M + H]^+^ (calcd for C_15_H_23_O_2_, 235.1693) from HRESIMS, with an IHD of five. The NMR spectroscopic data of **10** (Fig. 2, Table 1 and 2) showed it shared the same planar structure with 2-keto-5-hydroxyguai-3,11-diene [27], along with the main variation was ascribed to the C-7 relative configuration. Key NOESY correlations (Fig. 3) of H-1/Me-15 and H-7/H-10 suggested that H-1 and Me-15 occupied cofacial and were designated as *α*-oriented, while H-7 was *β*-oriented. Additionally, key NOESY correlations of 5-OH [*δ*_H_ 5.13 (1H, s)]/H-1/H-3 in the DMSO-*d*_6_ determined that 5-OH was *α*-orientation. Ultimately, the measured ECD spectrum of **10** (Fig. 5) was consistent with the computed ECD spectrum for (1*S*,5*S*,7*S*,10*R*), thus determining the absolute configuration of **10** as delineated. Eventually, the structure of **10** was established as (1*S*,5*S*,7*S*,10*R*)-5*α*-hydroxy-1*α*,7*β*,10*β*,(H)-guaia-3(4),11(12)-dien-2-oxo and named as sanguisorbaol J.

The molecular formula of **11** (colorless oil) was deduced to be C_15_H_24_O_4_ from its HRESIMS ions at *m/z* 269.1746 [M + H]^+^ (calcd for C_15_H_25_O_4_, 269.1747) together with ^13^C NMR spectroscopic signals, requiring an IHD of four. Comparative analysis of the NMR spectral info (Fig. 2, Table 1 and 2) of compound **11** with (4*R*,7*S*,10*S*)-2-oxo-4*α*,11-dihydroxyguaia-1(5)-ene revealed substantial similarities [28], with the exception that one oxygenated methylene group in **11** replaced the methyl group. These observations were supported by the HMBC correlations (Fig. 2) from Me-13 to C-7, C-11 and C-12, and located the oxygenated methylene at C-12. The NOESY correlations (Fig. 3) of Me-15/H-6*β* (*δ*_H_ 2.24)/H-7 indicated that H-7 and Me-15 were *β*-oriented, since the NOESY cross-peaks of Me-14/H-6*α* (*δ*_H_ 3.08) confirmed these protons were *α*-oriented. However, it was not feasible to determine the relative configuration at C-11 based on the above data. In response to the uncertainty of C-11, four possible isomers models, namely, (4S*,7S*,10S*,11S*), (4S*,7S*,10S*,11R*), (4R*,7R*,10R*,11R*) and (4R*,7R*,10R*,11S*), which constitute a pair of epimers and their enantiomers. Two epimers (4S*,7S*,10S*,11S*)-1 and (4S*,7S*,10S*,11R*)-2 underwent GIAO based quantum chemical ^1^H and ^13^C NMR calculation at MPW1PW91/6-311G(d,p) level utilizing the solvation model based on density (SMD) in methanol. Then, the DP4 + analysis was performed and the results (Figure S5–101) showed that (4S*,7S*,10S*,11R*)-2 (99.90%) was significantly better than (4S*,7S*,10S*,11S*)-1 (0.10%), which could be determined the relative configuration of **11** was (4S*,7S*,10S*,11R*) or (4R*,7R*,10R*,11S*). Eventually, the absolute configuration of compound **11** was established as (4*R*,7*R*,10*R*,11*S*) through analysis of the theoretical and measured ECD curves (Fig. 5), and **11** was elucidated as (4*R*,7*R*,10*R*,11*S*)-7*α*,10*β*,(H)-2-oxo-1(5)-guaiaene-4*α*,11,12-triol and designated as sanguisorbaol K.

Compound **12** was purified as colorless needle crystals (MeOH). The molecular formula of compound **12** was assigned as C_15_H_24_O_2_ according to the HRESIMS at *m/z* 237.1847 [M + H]^+^ (calcd for C_15_H_25_O_2_, 237.1849), with four IHD. The ^1^H NMR data (Table 1) exhibited a pair of olefinic protons at *δ*_H_ 4.63 (1H, br s, H-12a) and 4.58 (1H, br s, H-12b), one *O*-bearing methine at *δ*_H_ 3.81 (1H, t,* J* = 2.7 Hz, H-9), and three methyls at *δ*_H_ 1.68 (3H, br s, H-13), 1.03 (3H, s, H-14) and 1.00 (3H, d, *J* = 6.7 Hz, H-15). A detailed analysis of the ^13^C NMR (Table 2) and DEPT spectrum exhibited 15 carbon resonances, encompassing three quaternary carbons containing a carbonyl group at *δ*_C_ 220.2 (C-10) and an olefinic one at *δ*_C_ 152.4 (C-11), four methines with an oxygenated one at *δ*_C_ 78.5 (C-9), five methylenes with an olefinic carbon at *δ*_C_ 109.0 (C-12), and three methyls. Among which, *δ*_C_ 152.4 (C-11), 109.0 (C-12) and 19.5 (C-13) indicated the presence of an isopropenyl unit in the structure. Taking into account that two IHDs were ascribed to the carbonyl group and the double bond, **12** should also be a bicyclic sesquiterpene. The ^1^H–^1^H COSY correlation of H_2_-2/H_2_-3/H-4/H_2_-5/H-6/H-7/H_2_-8(Me-15)/H-9, complemented by a series of HMBC correlations (Fig. 2) from Me-14 to C-1, C-2, C-9 and C-10, H-6 to C-1, C-4, C-5, C-7, C-8, C-10 and C-15, and H_2_-2, H_2_-5, H-9 to C-10, unambiguously confirmed a 6/7-fused bicyclic framework featuring C-1 and C-6 as bridgehead carbons and the carbonyl group located on the bridge. Additionally, the HMBC correlations from Me-15 to C-6, C-7, and C-8 indicated that Me-15 was attached to C-7. Simultaneously, the HMBC correlations of Me-13 with C-4, C-11, and C-12, combined with those of H_2_-12 with C-4, C-11, and C-13 revealed the presence of an isopropenyl at C-4. Moreover, C-9 was deduced to be functionalized with a hydroxyl group based on its oxygenated characteristic and molecular formula. The NOESY correlations (Fig. 3) of H-4 with H-6, H-9 with Me-15, and H-9 with H_2_-2 demonstrated that H-4, H-6, H-7, Me-14, and 9-OH adopted a uniform orientation and assigned as *α*, thereby disclosing that the bridged carbonyl group resided beneath the bicyclic framework. Thereafter, ECD simulations were employed to speculate the absolute stereochemistry of **12**. The calculated ECD spectrum of (1*R*,4*R*,6*S*,7*S*,9*S*)-**12a** matched well with the experimental one (Fig. 5), confirmed by X-ray crystallography experiment [Cu-K*α* radiation, Flack parameter = 0.01(10), CCDC 2,333,292] (Fig. 4). As a result, the structure of **12** was elucidated and given the name sanguisorbaol L. Structurally, **12** was the third natural occurring isopropenyl-branched bicyclo [4.3.1] decanone sesquiterpenoid featuring an angular methyl and a secondary methyl, following pogocablenes A and B from *P. cablin* [24]. The absolute configuration was first definitively identified using single-crystal X-ray diffraction technology.

In addition, twenty-five known compounds (**13–37**) were isolated from *S. officinalis*. Their structures were determined to be (3R*)-3 hydroxypatchoulol [22] (**13**), (5*R*)-5-hydroxypatchoulol [29] (**14**), 6*β*-hydroxypatchoulol [21] (**15**), (8S*)-8 hydroxypatchoulol [22] (**16**), (9R*)-9 hydroxypatchoulol [22] (**17**), 1,3,4,4*α*,5,6,7,8*α*-octahydro-1-hydroxy-4,8*α*,9,9-tertramethyl-1,6-methanonaphthalen-8-(2H)one [30] (**18**), ( ±)-hydroxy patchouli alcohol [23] (**19**), 3*α*,9*β*-dihydroxypathoulol [23] (**20**), (5*R*,8*S*)-5,8 dihydroxypatchoulol [22] (**21**), (6S*,9S*)-6,9 dihydroxypatchoulol [22] (**22**), 8*α*,9*α*-dihydroxypatchoulol [21] (**23**), pogocablene M [24] (**24**), 2-keto-1(5)-*β*-patchoulene-4*β*-ol [31] (**25**), 2-keto-1(5)-*β*-patchoulene-4*α*-ol [31] (**26**), (3*R*,5*R*,8*S*)-3-hydroxy-3,8-dimethyl-5-(prop-1-en-2-yl)-3,4,5,6,7,8-hexahydroazulen-1(2H)-one [32] (**27**), (3*S*,5*R*,8*S*)-3-hydroxy-3,8-dimethyl-5-(prop-1-en-2-yl)-3,4,5,6,7,8-hexahydroazulen-1(2H)-one [32] (**28**), pogocablene J [24] (**29**), (1*R*,4*S*,5*S*,7*R*,10*R*,11*R*)-guaiane-10,11,12-triol [33] (**30**), xylaguaianol C [25] (**31**), pogocablene O [24] (**32**), clovane-2*β*,9*α*-diol [34, 35] (**33**), senecrassidiol [36] (**34**), caryolane-1,9*β*-diol [34] (**35**), linariophyllene A [37] (**36**) and mandassidione [38] (**37**) through comparison of their NMR spectra with information from the literature. Among them, compound **18** represented the first naturally occurring patchoulol-type sesquiterpenoid, having been previously reported only as a synthetic product. Additionally, compounds **13–37** were first isolated from plants of the Rosaceae family.

It was worth mentioned that patchoulol-type sesquiterpenoids were isolated for the first time from the genus *Sanguisorba*, and there have been few reports of patchoulol-type sesquiterpenoids in the Rosaceae family. UPLC–Q–TOF–MS/MS was used for the analysis and verification of patchoulol-type sesquiterpenoids in *S. officinalis* (Supplementary material). Six patchoulol-type sesquiterpenoids (**3**–**5**, **14**, **15** and **19**) underwent UPLC–Q–TOF–MS/MS detection in the manner of positive ions, yielding accurate mass measurements and MS^2^ data as shown in Table S18.

The above results showed that six patchoulol-type sesquiterpenoids were all prone to dehydration to form characteristic molecular ions peaks of [M − nH_2_O + H]^+^ along with diagnostic fragment ion peaks with *m/z* 147, 133, 119, 105, 91, 77, 63, 67, and 55 (Table S18), which were also detected in the methanol extract of *S. officinalis* (Figure S18). Additionally, the hypothetical biosynthetic pathway for patchoulol- and guaiane-type sesquiterpenoids is proposed in Fig. 6. Herein, two mechanistic pathways are proposed for the biosynthesis: the free radical mechanism and the carbocation mechanism.

Free Radical Mechanism

Through the mevalonic acid (MVA) pathway, farnesyl pyrophosphate (FPP) is biosynthesized via the condensation of two ∆^3^-isopentenyl pyrophosphate (IPP) molecules with *γ*,*γ*-dimethylallyl pyrophosphate (DMAPP). Subsequent elimination of pyrophosphate from FPP generates farnesene, which undergoes cyclization to form a guaiane-type radical intermediate. Structural diversification occurs through: (i) C-5 to C-1,10 hydride shifts, followed by C-1/C-11 cyclization and site-specific redox modifications, yielding patchoulol-type sesquiterpenoids (e.g., compounds **1–6** and **13–24**) [39]. (ii) C-10,11 cyclization of the guaiane radical to form 10,11-tricyclic guaiane-type sesquiterpenoids, with subsequent redox transformations affording compounds **25** and **26 **[40, 41]. (iii) Hydration of the guaiane radical produces guaiol. Photooxidation of guaiol generates peroxide intermediates and guaiane diol, which undergoes 1,5-cyclization with dehydration to form epoxy-containing guaiane derivatives (e.g., compound **29**). Alternatively, guaiane diol undergoes dehydration and 1,10-cleavage to yield compound **37**.

Carbocation Mechanism

Farnesyl cation intermediates undergo cyclization and electron transfers to generate guaiane carbocations. Further redox modifications produce diverse guaiane derivatives (e.g., compounds **7–11**, **27**, **28**, **30** and **31**) [42]. Notably, farnesyl cations exhibit divergent cyclization patterns: (i) C-2,5 cyclization forms the caryophyllene-type 10-membered bicyclic system. Subsequent C-8/C-12 cyclization generates the caryolanyl cation, which upon hydration produces sesquiterpenoids **34** and **35** [43]. (ii) The strained four-membered ring caryolanyl cation undergoes Wagner-Meerwein rearrangement to form the five-membered ring clovanyl cation. Hydration of this intermediate yields compound **33** [44, 45].

### Anti-melanogenic activity of sesquiterpenoids in IBMX-stimulated B16F10 melanoma cells

**Fig. 7:**
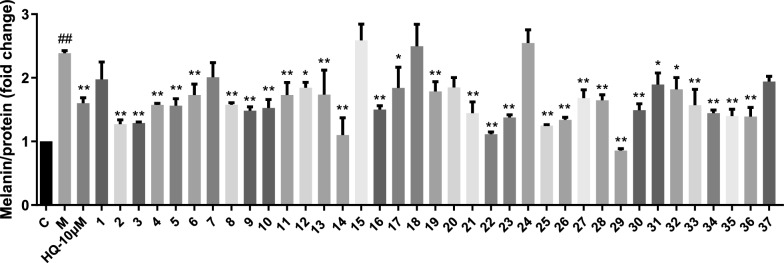
Anti-melanogenic activity of compounds **1–37** in IBMX-stimulated B16F10 melanoma cells that were co-treated with compounds **1–37** (except for compound **5** with a concentration of 6.25 µM and **27** with 25 µM, all other compounds have a concentration of 50 µM) or hydroquinone (HQ, 10 µM). ^##^*p* < 0.01 compared with the control group; *p < 0.05, **p < 0.01 compared with the model group. Values are presented as the mean ± SEM (n = 3)

Given that *S. officinalis* has been widely used in dermatology due to its excellent skin repair and whitening effects [16–19]. Our preliminary research has further revealed that sesquiterpenoids exhibited significant inhibitory effects on melanin production [20]. All isolates from *S. officinalis* were further assessed for their efficacy in inhibiting melanin synthesis in B16F10 mouse-derived melanoma cells. The results (Fig. 7) revealed that compounds **2–6**, **8–14**, **16**, **17**, **19**, **21–23** and **25–36** exhibited significant inhibitory activities on melanin biosynthesis. However, the CCK-8 assay results (Figure S20) revealed that compounds **2**, **6**, **11**, **16**, **23**, **28**, **30**, **33**, **35** and **36** showed significant cytotoxicity in B16F10 cells at the tested concentration over 48 h, which could lead to false-positive results and were thus excluded from further consideration. Consequently, compounds **3–5**, **8–10**, **12–14**, **17**, **19**, **21**, **22**, **25–27**, **29**, **31**, **32** and **34** were identified as effective melanogenesis inhibitors without apparent cytotoxicity. Among them, compounds **3–5**, **8–10**, **14**, **21**, **22**, **25**, **26**, **29** and **34** demonstrated superior melanogenesis inhibition compared to HQ (57% inhibiton), with inhibition rates ranging from 58 to 110% relative to the model group (Table S19). Notably, compound **29** displayed the highest potency (110% inhibiton), which may be attributed to the incorporation of a 1,5-epoxy ring within its molecular structure. Detailed structural-activity relationship (SAR) analysis revealed that carbonyl substitution on the patchoulol-type sesquiterpenoids skeleton (e.g., compounds **18** and **24**) markedly reduced anti-melanogenic activity. In contrast, nor-patchoulol-type maintained comparable potency (e.g., **5** vs. **13** and **14**). Among 18 patchoulol-type derivatives, the number of hydroxyl groups on the core scaffold showed negligible impact on activity. However, the hydroxylation at C-7 position resulted in significant loss of efficacy, as evidenced by comparisons of **15** vs. **14** and **17**. For guaiane-type sesquiterpenoids (**7–11** and **25–31**), most derivatives displayed robust activity, with cyclization at the C-10–C-11 positions notably enhancing potency. These discoveries highlighted the structural diversity of sesquiterpenoids as a valuable resource for rational design of anti-melanogenic agents, while the underlying molecular mechanisms warrant further investigation.

## Conclusion

In summary, thirty-seven sesquiterpenoids, including twelve novel compounds (**1–12**) along with twenty-five previously identified ones (**13–37**), were isolated from *S. officinalis*. Among them, compounds **1–6** and **13–24** were patchoulol-type sesquiterpenoids, while compounds **7–11** and **25–31** were guaiane-type sesquiterpenoids. Structurally, the absence of the C-11 carbon atom was confirmed in compounds **5**, **6**, and **24**, characterizing them as nor-patchoulol-type sesquiterpenoids. Additionally, compounds **7** and **8** were found to be two C-11 epimers. Compound **12** is particularly notable for possessing a rare carbon skeleton based on a bicyclo[4.3.1]decane core, making it a rare example of this scaffold isolated from natural sources. All above compounds mark the first report of such structures within the genus *Sanguisorba*, the further analysis using UPLC–Q–TOF–MS/MS provided robust experimental evidence for an expanded phytochemical profile of *S. officinalis*. These findings not only enrich the sesquiterpenoid profile of this genus but also establish a critical foundation for exploring their biogenetic pathways and pharmacological potential. Following anti-melanogenic activity assay, twenty compounds showed potential inhibitory activities. In special, compounds **3–5**, **8–10**, **14**, **21**, **22**, **25**, **26**, **29** and **34** demonstrated superior melanogenesis inhibitory activity to that of HQ (57% inhibition), with inhibition rates relative to the model group ranging from 58 to 110%. In conclusion, our study highlights the sesquiterpenoids from *S. officinalis* as a valuable natural resource with the potentiality for development into novel anti-melanogenic agents in the cosmeceutical products.

## Materials and methods

### General experimental procedures

These are provided in the Supplementary Material.

### Plant material

The roots of *Sanguisorba officinalis* (Lot.:181,227), purchased from Shanghai Kangqiao Chinese Herb Slices Co., Ltd. (Shanghai, China), was originated from Jilin Province and harvested in October–November (autumn). The voucher specimen (No. SDY181227) was authenticated by one of the authors (Yi-Ming Li) and deposited in the Science and Technology Innovation Center, Shanghai University of Traditional Chinese Medicine.

### Extraction and isolation of new compounds 1–12

The dried roots of *S. officinalis* (20 kg) were powdered and subjected to reflux extraction with 95% EtOH at 85 ℃ for three times (200 L, 1.5 h each). After concentration, a crude residue (6.0 kg) was produced. The following was then suspended in water (20 L) and subsequently partitioned with PE (3 × 20.0 L) and EA (3 × 20.0 L) to obtain EA-soluble fractions.

The EA fraction (2578 g) was dispersed in water (5.0 L) and then processed on a 25.0 L Diaion 101 macroporous resin CC eluted with 0%, 20%, 40%, 60%, 80% and 95% EtOH and detected by TLC to yield six fractions (Fr. I–VI). Fr. II (978 g) was chromatographed on a polyamide CC (100 × 22 cm, 10.0 kg) eluting with 0%, 20%, 40%, 60%, 80% and 95% EtOH to afford six major fractions (Fr. IIA–F). Fr. IIA and Fr. IIB, which were enriched with sesquiterpenoids, were further isolated to afford compounds **1–37**. Detailed isolation procedures of the known compounds **13–37** are provided in the Supplementary Material.

Fr.IIA (369 g) was separated on a silica gel (200–300 mesh) column and using a gradual slope of CH_2_Cl_2_–MeOH (200:1 to 1:5) to give ten fractions (Fr.IIA1–10). Fr.IIA7 (5560 mg) was conducted on a silica gel CC, eluting with CH_2_Cl_2_–MeOH (100:1 to 5:1) to obtain seven fractions (Fr.IIA7A–G). Fr.IIA7G (2094 mg) was subjected to ODC CC (MeOH–H_2_O, 20:80 to 100:0) to afford twelve fractions (Fr.IIA7G-1–12). Fr.IIA7G-9 (33 mg) was separated by repeated semi-prep-HPLC (CH_3_CN–H_2_O, 17:83, 3.0 mL/min) to yield compounds **11** (4 mg, *t*_R_ = 18.22 min).

Fr.IIB (69 g) was chromatographically separated using a silica gel (55 cm × 10 cm) and produced nine fractions (Fr.IIB1–9) by eluting using a CH_2_Cl_2_–MeOH gradient system (500:1 to 5:1, v/v). Fr.IIB1 (3.527 g) and Fr.IIB3 (0.835 g) were isolated utilizing Sephadex LH-20 CC using PE–CH_2_Cl_2_–MeOH isocratic system (5:5:1) to obtain the six sub-fractions Fr.IIB1A–C and Fr.IIB3A–C. Fr.IIB1A (3314 mg) was isolated on a silica gel CC using step CYH–EA mixed-solvent system (100:1 to 1:1, v/v) to give ten fractions (Fr.IIB1A-1–10). Fr.IIB1A–7 (150 mg) was conducted on a ODS CC using step MeOH–H_2_O mixed-solvent system (20:80 to 70:30, v/v) to obtain three fractions (Fr.IIB1A-7a–c). Fr.IIB1A-7b (82 mg) was separated by MCI CC eluted with MeOH–H_2_O (20:80 to 75:25, v/v) yield Fr.IIB1A-7b-1 (38 mg) and Fr.IIB1A-7b-2 (21 mg). Fr.IIB1A-7b-2 (21 mg) was chromatographed on semi-preparative HPLC (CH_3_CN–H_2_O, 45:55, 3.0 mL/min) to yield compound **12** (12 mg, *t*_R_ = 19.47 min). Fr.IIB1A–9 (122 mg) was separated by semi-prep-HPLC (CH_3_CN–H_2_O, 30:70, 3.0 mL/min) to obtain compounds **9** (2 mg, *t*_R_ = 43.93 min) and **10** (2 mg, *t*_R_ = 64.05 min). Fr.IIB3B (387 mg) was fractionated on MCI CC using MeOH–H_2_O step mixed-solvent system (40:60 to 100:0) to give Fr.IIB3B-1 and Fr.IIB3B-2. Fr.IIB3B-1 (96 mg) was fractionated on silica gel CC eluted with CYH–EA (50:1 to 4:1) and purified by ODS CC (MeOH–H_2_O, 70:30) to afford compound **5** (25 mg). Fr.IIB5 (1007 mg) was separated into twelve subfractions (Fr.IIB5A–L) after an ODS CC using a stepwise gradient of MeOH–H_2_O from 20:80 to 100:0. Fr.IIB5C (50 mg) was conducted on a prep-TLC using CH_2_Cl_2_–MeOH (20:1) as the eluent, followed by ODS CC (MeOH–H_2_O, 70:30) yielded compound **3** (26 mg). Fr.IIB5D (63 mg) was chromatographed over preparative TLC (CH_2_Cl_2_–MeOH, 20:1), subsequently refined via ODS CC (MeOH–H_2_O, 70:30) to isolate **4** (7 mg). Fr.IIB5E (53 mg) was conducted on semi-prep-HPLC separation (CH_3_CN–H_2_O, 20:80, 3.0 mL/min) gain compounds **7** (4 mg, *t*_R_ = 33.00 min) and **8** (5 mg, *t*_R_ = 34.50 min). Fr.IIB7 (2.006 g) was conducted on an ODS CC using MeOH–H_2_O from 20:80 to 100:0, giving nineteen sub-fractions (Fr.IIB7-1–19). Fr.IIB7-5 (37 mg) was subjected to prep-TLC (CYH–EA, 1:5) and purified via ODS CC (MeOH–H_2_O, 70:30) to afford **6** (5 mg). Fr.IIB7-9 (182 mg) was conducted on a silica gel CC (CYH–EA, 15:1 to 1:3) to gain three sub-fractions (Fr.IIB7-9A–C). Fr.IIB7-9A (88 mg) was further purified on a Sephadex LH-20 (MeOH–H_2_O, 70:30) CC, producing compound **1** (85 mg). Compounds **2** (5 mg) were obtained by repeating the same procedure with Fr.IIB7-14 (70 mg).

### Spectroscopic data of new compounds

Sanguisorbaol A (**1**): colorless needles crystals (MeOH); $[\alpha]_{\mathrm{D}}^{20}$−64.17 (*c* 0.10, MeOH); ECD (*c* 3.94, MeOH) *λ*_max_ (Δ*ε*) 191 (2.42), 198 (0.82), 202 (1.23), 239 (− 0.24), 257 (0.36), 317 (− 0.45) nm; UV (MeOH)* λ*_max_ 237 nm; ^1^H and ^13^C NMR data (Tables 1 and 2); HRESIMS *m/z* 277.1775 [M + Na]^+^ (calcd for C_15_H_26_O_3_Na, 277.1774).

Sanguisorbaol B (**2**): colorless oil; $[\alpha]_{\mathrm{D}}^{20}$−43.67 (*c* 0.10, MeOH); ECD (*c* 1.57, MeOH) *λ*_max_ (Δ*ε*) 190 (− 10.32), 232 (0.27) nm; UV (MeOH)* λ*_max_ 210 nm; ^1^H and ^13^C NMR data (Tables 1 and 2); HRESIMS *m/z* 277.1775 [M + Na]^+^ (calcd for C_15_H_26_O_3_Na, 277.1774).

Sanguisorbaol C (**3**): colorless needles crystals (MeOH); $[\alpha]_{\mathrm{D}}^{20}$−29.50 (*c* 0.10, MeOH); ECD (*c* 3.94, MeOH) *λ*_max_ (Δ*ε*) 190 (− 5.84), 191 (1.54), 216 (− 2.35), 245 (0.45) nm; UV (MeOH)* λ*_max_ 210, 280 nm; ^1^H and ^13^C NMR data (Tables 1 and 2); HRESIMS *m/z* 277.1773 [M + Na]^+^ (calcd for C_15_H_26_O_3_Na, 277.1774).

Sanguisorbaol D (**4**): colorless oil; $[\alpha]_{\mathrm{D}}^{20}$−7.00 (*c* 0.10, MeOH); ECD (*c* 3.94, MeOH) *λ*_max_ (Δ*ε*) 191 (− 10.67), 242 (2.27) nm; UV (MeOH)* λ*_max_ 210, 257 nm; ^1^H and ^13^C NMR data (Tables 1 and 2); HRESIMS *m/z* 237.1833 [M − H_2_O + H]^+^ (calcd for C_15_H_25_O_2_, 237.1849).

Sanguisorbaol E (**5**): colorless needles crystals (EtOH); $[\alpha]_{\mathrm{D}}^{20}$−92.50 (*c* 0.10, MeOH); ECD (*c* 4.46, MeOH) *λ*_max_ (Δ*ε*) 191 (− 18.08), 211 (0.26) nm; UV (MeOH)* λ*_max_ 210, 278 nm; ^1^H and ^13^C NMR data (Tables 1 and 2); HRESIMS *m/z* 207.1742 [M − H_2_O + H]^+^ (calcd for C_14_H_23_O, 207.1743).

Sanguisorbaol F (**6**): colorless oil; $[\alpha]_{\mathrm{D}}^{20}$−39.00 (*c* 0.10, MeOH); ECD (*c* 1.38, MeOH) *λ*_max_ (Δ*ε*) 190 (12.56), 205 (− 1.80) nm; UV (MeOH)* λ*_max_ 210, 229, 280 nm; ^1^H and ^13^C NMR data (Tables 1 and 2); HRESIMS *m/z* 263.1617 [M + Na]^+^ (calcd for C_14_H_24_O_3_Na, 263.1618).

Sanguisorbaol G (**7**): colorless oil; $[\alpha]_{\mathrm{D}}^{20}$ 10.33 (*c* 0.10, MeOH); ECD (*c* 3.97, MeOH) *λ*_max_ (Δ*ε*) 190 (− 8.69), 209 (4.78), 237 (1.42), 262 (3.11), 338 (− 1.55) nm; UV (MeOH)* λ*_max_ 260 nm; ^1^H and ^13^C NMR data (Tables 1 and 2); HRESIMS *m/z* 253.1798 [M + H]^+^ (calcd for C_15_H_25_O_3_, 253.1798).

Sanguisorbaol H (**8**): colorless oil; $[\alpha]_{\mathrm{D}}^{20}$ 2.00 (*c* 0.10, MeOH); ECD (*c* 3.97, MeOH) *λ*_max_ (Δ*ε*) 190 (− 13.28), 207 (2.22), 218 (− 0.08), 246 (4.75), 330 (− 2.63) nm; UV (MeOH)* λ*_max_ 260 nm; ^1^H and ^13^C NMR data (Tables 1 and 2); HRESIMS *m/z* 253.1800 [M + H]^+^ (calcd for C_15_H_25_O_3_, 253.1798).

Sanguisorbaol I (**9**): colorless oil; $[\alpha]_{\mathrm{D}}^{20}$−11.33 (*c* 0.10, MeOH); ECD (*c* 4.27, MeOH) *λ*_max_ (Δ*ε*) 191 (8.45), 208 (− 10.06), 231 (− 0.58), 247 (− 1.70) nm; UV (MeOH)* λ*_max_ 238 nm; ^1^H and ^13^C NMR data (Tables 1 and 2); HRESIMS *m/z* 235.1696 [M + H]^+^ (calcd for C_15_H_23_O_2_, 235.1693).

Sanguisorbaol J (**10**): colorless oil; $[\alpha]_{\mathrm{D}}^{20}$ 22.17 (*c* 0.10, MeOH); ECD (*c* 4.27, MeOH) *λ*_max_ (Δ*ε*) 210 (19.30), 239 (− 46.39), 319 (11.70) nm; UV (MeOH)* λ*_max_ 210, 252 nm; ^1^H and ^13^C NMR data (Tables 1 and 2); HRESIMS *m/z* 235.1692 [M + H]^+^ (calcd for C_15_H_23_O_2_, 235.1693).

Sanguisorbaol K (**11**): colorless oil; $[\alpha]_{\mathrm{D}}^{20}$−148.83 (*c* 0.10, MeOH); ECD (*c* 0.75, MeOH) *λ*_max_ (Δ*ε*) 190 (21.93), 215 (− 10.18), 241 (27.68), 318 (− 12.48) nm; UV (MeOH)* λ*_max_ 240 nm; ^1^H and ^13^C NMR data (Tables 1 and 2); HRESIMS *m/z* 269.1746 [M + H]^+^ (calcd for C_15_H_25_O_4_, 269.1747).

Sanguisorbaol L (**12**): colorless needles crystals (MeOH); $[\alpha]_{\mathrm{D}}^{20}$−58.50 (*c* 0.10, MeOH); ECD (*c* 4.24, MeOH) *λ*_max_ (Δ*ε*) 191 (− 12.30), 195 (21.54), 209 (− 2.89), 239 (− 0.15), 297 (− 12.87) nm; UV (MeOH)* λ*_max_ 210, 290 nm; ^1^H and ^13^C NMR data (Tables 1 and 2); HRESIMS *m/z* 237.1847 [M + H]^+^ (calcd for C_15_H_25_O_2_, 237.1849).

### X-ray crystallographic analysis

Single crystals of compounds **1**, **3**, **5**, and **12** were grown at 4 °C. Crystals of **1**, **3**, and **12** were obtained from MeOH, while those of **5** were obtained from EtOH. Cu–K*α* (*λ* = 1.54178 Å) was employed to acquire the crystallographic data of **1**, **3**, **5**, and **12** at 150 K, 100 K, 100 K, and 170 K, respectively, utilizing Bruker Apex II CCD diffractometer. X-ray crystallographic structures of these compounds were solved through the direct method and subjected to full-matrix least-squares refinement with SHELX-2014 software. The crystallography info of compounds **1**, **3**, **5** and **12** were deposited at the Cambridge Crystallographic Data Centre (deposition numbers CCDC: 2,333,284, 2,333,293, 2,333,300 and 2,333,292).

Crystallographic data of sanguisorbaol A (**1**): C_15_H_26_O_3_, *M* = 254.36 g/mol, trigonal, size = 0.150 × 0.080 × 0.050 mm^3^, *a* = 7.7907(6) Å, *b* = 7.7907(6) Å, *c* = 19.532(2) Å, *α* = 90°, *β* = 90°, *γ* = 120°, *V* = 1026.7(2) Å3, *T* = 150 K, space group *P*3_1_, *Z* = 3, *μ*(Cu K*α*) = 0.666 mm^−1^, *ρ*_calcd_ = 1.234 g/m^3^, *F* (000) = 420.0, 13,376 reflections measured (13.886° ≤ 2θ ≤ 148.842°), 2764 independent reflections (*R*_int_ = 0.0738, *R*_sigma_ = 0.0485). Final *R*_1_ values were 0.0384 (*I* ≥ 2*σ*(*I*)) and 0.0392 (all data), with corresponding *wR*_2_ values of 0.0992 (*I* ≥ 2*σ* (*I*)) and 0.1006 (all data). The goodness-of-fit on *F*^2^ was 1.113. Flack parameter = 0.05(11). CCDC 2,333,284.

Crystallographic data of sanguisorbaol C (**3**): C_15_H_26_O_3_, *M* = 254.36 g/mol, hexagonal, size = 0.120 × 0.060 × 0.040 mm^3^, *a* = 7.55240(10) Å, *b* = 7.55240(10) Å, *c* = 41.4232(8) Å, *α* = 90°, *β* = 90°, *γ* = 120°, *V* = 2046.18(7) Å3, *T* = 100 K, space group *P*6_5_, *Z* = 6, *μ*(Cu K*α*) = 0.668 mm^−1^, *ρ*_calcd_ = 1.239 g/m^3^, *F* (000) = 840.0, 17,665 reflections measured (13.538° ≤ 2θ ≤ 144.210°), 2686 independent reflections (*R*_int_ = 0.0822, *R*_sigma_ = 0.0447). Final *R*_1_ values were 0.0355 (*I* ≥ 2*σ*(*I*)) and 0.0370 (all data), with corresponding *wR*_2_ values of 0.0883 (*I* ≥ 2*σ* (*I*)) and 0.0897 (all data). The goodness-of-fit on *F*^2^ was 1.057. Flack parameter = 0.10(11). CCDC 2,333,293.

Crystallographic data of sanguisorbaol E (**5**): C_14_H_24_O_2_, *M* = 224.33 g/mol, orthorhombic, size = 0.080 × 0.050 × 0.040 mm^3^, *a* = 14.9889(3) Å, *b* = 21.4839(4) Å, *c* = 7.5409(2) Å, *α* = 90°, *β* = 90°, *γ* = 90°, *V* = 2428.32(9) Å3, *T* = 100 K, space group *P*2_1_2_1_2, *Z* = 8, *μ*(Cu K*α*) = 0.620 mm^−1^, *ρ*_calcd_ = 1.227 g/m^3^, *F* (000) = 992.0, 46,814 reflections measured (7.190° ≤ 2θ ≤ 149.412°), 4961 independent reflections (*R*_int_ = 0.0776, *R*_sigma_ = 0.0368). Final *R*_1_ values were 0.0393 (*I* ≥ 2*σ*(*I*)) and 0.0417 (all data), with corresponding *wR*_2_ values of 0.0974 (*I* ≥ 2*σ* (*I*)) and 0.0999 (all data). The goodness-of-fit on *F*^2^ was 1.038. Flack parameter = 0.00(8). CCDC 2,333,300.

Crystallographic data of sanguisorbaol L (**12**): C_15_H_24_O_2_, *M* = 236.34 g/mol, orthorhombic, size = 0.120 × 0.080 × 0.060 mm^3^, *a* = 6.26610(10) Å, *b* = 12.8195(2) Å, *c* = 35.1835(7) Å, *α* = 90°, *β* = 90°, *γ* = 90°, *V* = 2826.23(8) Å3, *T* = 170 K, space group *P*2_1_2_1_2_1_, *Z* = 8, *μ*(Cu K*α*) = 0.558 mm^−1^, *ρ*_calcd_ = 1.111 g/m^3^, *F* (000) = 1040.0, 44,974 reflections measured (5.024° ≤ 2θ ≤ 149.126°), 5755 independent reflections (*R*_int_ = 0.0850, *R*_sigma_ = 0.0397). Final *R*_1_ values were 0.0983 (*I* ≥ 2*σ*(*I*)) and 0.1026 (all data), with corresponding *wR*_2_ values of 0.0974 (*I* ≥ 2*σ* (*I*)) and 0.0999 (all data). The goodness-of-fit on *F*^2^ was 1.043. Flack parameter = 0.01(10). CCDC 2,333,292.

### Computational methods of ECD

CONFLEX software was utilized to obtain low-energy conformers (energy ≤ 5.0 kcal/mol) for compounds **1–12**, based on the MMFF94. For the purpose of ECD calculation, Gaussian 09 software was applied to perform geometric optimization and frequency analysis of the conformers, adopting the TDDFT method at the basis set level of B3LYP/6-31G(d). Next, stable conformers were selected for ECD calculation via TDDFT method at the M062X/Def2-TZVP level. Ultimately, Boltzmann averaging was implemented in SpecDis V1.70.1 and Origin 2018 to generate ECD spectra, which were then compared against experimental results [46].

### Methods of NMR calculation and DP4 + analysis

The conformational search of compound **11** was carried out using CONFLEX software with the MMFF94 force field. Ninety dominant conformers corresponding to the (4*S*,7*S*,10*S*,11*S*)-1 configuration and seventy to the (4*S*,7*S*,10*S*,11*R*)-2 configuration were initially obtained. All conformers showing a Boltzmann distribution above 0.1% were utilized to perform further geometry optimization and frequency computations at the B3LYP/6-31G(d) level in methanol using the solvation model based on density (SMD) solvent model by Gaussian 09 program package [47]. Optimized conformers were subsequently chosen to undergo GIAO calculations of ^13^C and ^1^H NMR shielding tensor values utilizing Gaussian 09 at the MPW1PW91/6–311 + G(d,p) level with SMD in methanol. Referenced chemical shifts were obtained by deducting the computationally derived tetramethylsilane shielding tensors at the identical theoretical level (^1^H = 31.8816; ^13^C = 183.7262) [48] from each nuclei′s shielding tensor values. Subsequently, the modified DP4 + probability method was utilized to analyze the theoretical and experimental NMR data, facilitating the identification of absolute configurations and hydroxyl positions for all candidate structures [49–51].

### Cell culture

B16F10 murine melanoma cells were maintained at 37 ℃ under 5% CO_2_ in a humidified atmosphere. The culture medium was Dulbecco′s Modified Eagle′s Medium (DMEM) supplemented with 10% fetal bovine serum (FBS), 100 units/mL of penicillin, and 100 µg/mL of streptomycin (RS-485, Thermo Scientific, USA).

### Cell viability assay

Cell viability of B16F10 cells was determined via the CCK-8 assay. Briefly, B16F10 cells were plated into 96-well plates at 1 × 10^6^ cells per well and cultured for 24 h. Compounds were incubated with the cells at varying concentrations for 48 h. Then, each well was supplemented with 10 μL CCK-8 solution and digested continuously at 37℃ for 1 h. According to the CCK-8 kit (Dojindo, Laboratories, Tokyo, Japan) instructions, absorbance was read at 450 nm on a microplate reader (Synergy HT, Bio Tek, USA). All experiments were carried out in triplicate.

### Measurement of cellular melanin contents

A previously established approach was used to determine the intracellular melanin content [20, 52, 53]. B16F10 cells (1 × 10^6^ cells/mL) were seeded on 6-well plates for 24 h. The cells were subjected to various doses of the sample and IBMX (100 μM) for 48 h following adherence growth, with HQ serving as the positive control. The cells were then centrifuged after two PBS (pH 6.8) washes. Subsequently, the cells were resuspended in 1 N NaOH containing 10% DMSO and incubated for two hours at 80 ℃. Finally, a microplate reader (BioTek, USA) was used to measure the absorbance values at 405 nm. All tests were performed in triplicate.

## Supplementary Information

Additional file 1. It contains NMR, HRESIMS, ECD, computational data and methodologies for quantum chemical calculations of new compounds **1–12**, crystal data of new compounds **1**, **3**, **5** and **12**, the anti-melanogenic activity and cells viability of all isolate compounds are available.

Additional file 2. It includes the calculated NMR data of compound **11**.

## Data Availability

All data generated and analyzed during this study are included in this published article and its supplementary information file.
