# Supplementary material for: New patchoulol- and guaiane-type sesquiterpenoids from Sanguisorba officinalis with anti-melanogenic activity
Source: Nat Prod Bioprospect. 2026 May 12;16(1):63. doi: 10.1007/s13659-026-00616-0 (PMC13161421; doi:10.1007/s13659-026-00616-0)
Supplement: Supplementary file 1 — Additional file 1. It contains NMR, HRESIMS, ECD, computational data and methodologies for quantum chemical calculations of new compounds 1–12, crystal data of new compounds 1, 3, 5 and 12, the anti-melanogenic activity and cells viability of all isolate compounds are available. [file 13659_2026_616_MOESM1_ESM.pdf]

Supplementary Material

New patchoulol- and guaiane-type sesquiterpenoids from *Sanguisorba officinalis* with anti-melanogenic activity

Longlong Wu[a,b,1], Jiarui Wu[a,1], Kaixian Chen[a], Liuqiang Zhang[a,*], Yiming Li[a,*]

[a] *School of Pharmacy, Shanghai University of Traditional Chinese Medicine, Shanghai 201203, People's Republic of China*

[b] *Institute of Chinese Medicine Resources, Anhui College of Traditional Chinese Medicine, Wuhu 241000, People's Republic of China*

[1] Both authors contributed equally to this work.

* Corresponding author.

E-mail addresses: 04100217@163.com (L. Zhang), ymlius@163.com (Y. Li).

List of contents

S1. Crystal data for **1** 1

Table S1-1 Crystal data and structure refinement for **1**. 1

Table S1-2 Fractional Atomic Coordinates (×10^4) and Equivalent Isotropic Displacement Parameters (Å^2×10^3) for **1**. U_{eq} is defined as 1/3 of of the trace of the orthogonalised U_{IJ} tensor. 2

Table S1-3 Anisotropic Displacement Parameters (Å^2×10^3) for **1**. The Anisotropic displacement factor exponent takes the form: $-2\pi^2[h^2a^{*2}U_{11}+2hka^*b^*U_{12}+\cdots]$........ 2

Table S1-4 Bond Lengths for **1**........ 3

Table S1-5 Bond Angles for **1**. 4

Table S1-6 Torsion Angles for **1**. 4

Table S1-7 Hydrogen Atom Coordinates (Å×10^4) and Isotropic Displacement Parameters (Å^2×10^3) for **1**. 6

S2. Crystal data for **3** 7

Table S2-1 Crystal data and structure refinement for **3**. 7

Table S2-2 Fractional Atomic Coordinates (×10^4) and Equivalent Isotropic Displacement Parameters (Å^2×10^3) for **3**. U_{eq} is defined as 1/3 of of the trace of the orthogonalised U_{IJ} tensor. 8

Table S2-3 Anisotropic Displacement Parameters (Å^2×10^3) for **3**. The Anisotropic displacement factor exponent takes the form: $-2\pi^2[h^2a^{*2}U_{11}+2hka^*b^*U_{12}+\cdots]$........ 8

Table S2-4 Bond Lengths for **3**........ 9

Table S2-5 Bond Angles for **3**. 10

Table S2-6 Torsion Angles for **3**. 10

Table S2-7 Hydrogen Atom Coordinates (Å×10^4) and Isotropic Displacement Parameters (Å^2×10^3) for **3**. 12

S3. Crystal data for **5** 13

Table S3-1 Crystal data and structure refinement for **5**. 13

Table S3-2 Fractional Atomic Coordinates (×10^4) and Equivalent Isotropic Displacement Parameters (Å^2×10^3) for **5**. U_{eq} is defined as 1/3 of of the trace of the orthogonalised U_{IJ} tensor. 14

Table S3-3 Anisotropic Displacement Parameters (Å^2×10^3) for **5**. The Anisotropic displacement factor exponent takes the form: $-2\pi^2[h^2a^{*2}U_{11}+2hka^*b^*U_{12}+\cdots]$........ 15

Table S3-4 Bond Lengths for **5**........ 16

Table S3-5 Bond Angles for **5**. 17

Table S3-6 Torsion Angles for **5**. 18

Table S3-7 Hydrogen Atom Coordinates (Å×10^4) and Isotropic Displacement Parameters (Å^2×10^3) for **5**. 20

S4. Crystal data for **12** 22

Table S4-1 Crystal data and structure refinement for **12**. 22

Table S4-2 Fractional Atomic Coordinates (×10^4) and Equivalent Isotropic Displacement Parameters (Å^2×10^3) for **12**. U_{eq} is defined as 1/3 of of the trace of the orthogonalised U_{IJ} tensor. 23

Table S4-3 Anisotropic Displacement Parameters (Å^2×10^3) for **12**. The Anisotropic displacement factor exponent takes the form: $-2\pi^2[h^2a^{*2}U_{11}+2hka^*b^*U_{12}+\cdots]$........ 24

Table S4-4 Bond Lengths for **12**........ 26

Table S4-5 Bond Angles for **12**. 26

Table S4-6 Torsion Angles for **12**. 27

Table S4-7 Hydrogen Atom Coordinates (Å×10^4) and Isotropic Displacement Parameters (Å^2×10^3) for **12**. 29

S5. Spectroscopic data........ 31

Figure S5-1. The HRESIMS spectrum of compound **1** 31

Figure S5-2. The ^{1}H NMR spectrum of compound **1** in CD_3OD (600 MHz) 31

Figure S5-3. The ^{13}C NMR spectrum of compound **1** in CD_3OD (150 MHz) 32

Figure S5-4. The DEPT 135 spectrum of compound **1** in CD_3OD (150 MHz)........ 32

Figure S5-5. The HSQC spectrum of compound **1** in CD_3OD (600 MHz)........ 33

Figure S5-6. The HMBC spectrum of compound **1** in CD_3OD (600 MHz)........ 33

Figure S5-7. The ^{1}H-^{1}H COSY spectrum of compound **1** in CD_3OD (600 MHz) 34

Figure S5-8. The NOESY spectrum of compound **1** in CD_3OD (600 MHz) 34

Figure S5-9. The ECD spectrum of compound **1** in MeOH 35

Figure S5-10. The HRESIMS spectrum of compound **2** 35

Figure S5-11. The ^{1}H NMR spectrum of compound **2** in CD_3OD (600 MHz) 36

Figure S5-12. The ^{13}C NMR spectrum of compound **2** in CD_3OD (150 MHz) 36

Figure S5-13. The DEPT 135 spectrum of compound **2** in CD_3OD (150 MHz)........ 37

Figure S5-14. The HSQC spectrum of compound **2** in CD_3OD (600 MHz)........ 37

Figure S5-15. The HMBC spectrum of compound **2** in CD_3OD (600 MHz)........ 38

Figure S5-16. The ^{1}H-^{1}H COSY spectrum of compound **2** in CD_3OD (600 MHz) 38

Figure S5-17. The NOESY spectrum of compound **2** in CD_3OD (600 MHz) 39

Figure S5-18. The ECD spectrum of compound **2** in MeOH 39

Figure S5-19. The HRESIMS spectrum of compound **3** 40

Figure S5-20. The ^{1}H NMR spectrum of compound **3** in CD_3OD (600 MHz) 40

Figure S5-21. The ^{13}C NMR spectrum of compound **3** in CD_3OD (150 MHz) 41

Figure S5-22. The DEPT 135 spectrum of compound **3** in CD_3OD (150 MHz)................ 41

Figure S5-23. The HSQC spectrum of compound **3** in CD_3OD (600 MHz) 42

Figure S5-24. The HMBC spectrum of compound **3** in CD_3OD (600 MHz).................... 42

Figure S5-25. The ^{1}H-^{1}H COSY spectrum of compound **3** in CD_3OD (600 MHz) 43

Figure S5-26. The NOESY spectrum of compound **3** in CD_3OD (600 MHz) 43

Figure S5-27. The ECD spectrum of compound **3** in MeOH 44

Figure S5-28. The HRESIMS spectrum of compound **4** 44

Figure S5-29. The ^{1}H NMR spectrum of compound **4** in CD_3OD (600 MHz) 45

Figure S5-30. The ^{13}C NMR spectrum of compound **4** in CD_3OD (150 MHz) 45

Figure S5-31. The DEPT 135 spectrum of compound **4** in CD_3OD (150 MHz)................ 46

Figure S5-32. The HSQC spectrum of compound **4** in CD_3OD (600 MHz) 46

Figure S5-33. The HMBC spectrum of compound **4** in CD_3OD (600 MHz).................... 47

Figure S5-34. The ^{1}H-^{1}H COSY spectrum of compound **4** in CD_3OD (600 MHz) 47

Figure S5-35. The NOESY spectrum of compound **4** in CD_3OD (600 MHz) 48

Figure S5-36. The ECD spectrum of compound **4** in MeOH 48

Figure S5-37. The HRESIMS spectrum of compound **5** 49

Figure S5-38. The ^{1}H NMR spectrum of compound **5** in CD_3OD (600 MHz) 49

Figure S5-39. The ^{13}C NMR spectrum of compound **5** in CD_3OD (150 MHz) 50

Figure S5-40. The DEPT 135 spectrum of compound **5** in CD_3OD (150 MHz)................ 50

Figure S5-41. The HSQC spectrum of compound **5** in CD_3OD (600 MHz) 51

Figure S5-42. The HMBC spectrum of compound **5** in CD_3OD (600 MHz).................... 51

Figure S5-43. The ^{1}H-^{1}H COSY spectrum of compound **5** in CD_3OD (600 MHz) 52

Figure S5-44. The NOESY spectrum of compound **5** in CD_3OD (600 MHz) 52

Figure S5-45. The ECD spectrum of compound **5** in MeOH 53

Figure S5-46. The HRESIMS spectrum of compound **6** 53

Figure S5-47. The ^{1}H NMR spectrum of compound **6** in CD_3OD (600 MHz) 54

Figure S5-48. The ^{13}C NMR spectrum of compound **6** in CD_3OD (150 MHz) 54

Figure S5-49. The DEPT 135 spectrum of compound **6** in CD_3OD (150 MHz)................ 55

Figure S5-50. The HSQC spectrum of compound **6** in CD_3OD (600 MHz) 55
Figure S5-51. The HMBC spectrum of compound **6** in CD_3OD (600 MHz) 56
Figure S5-52. The ^1H-^1H COSY spectrum of compound **6** in CD_3OD (600 MHz) 56
Figure S5-53 The NOESY spectrum of compound **6** in CD_3OD (600 MHz) 57
Figure S5-54. The ECD spectrum of compound **6** in MeOH 57
Figure S5-55. The HRESIMS spectrum of compound **7** 58
Figure S5-56. The ^1H NMR spectrum of compound **7** in CD_3OD (600 MHz) 58
Figure S5-57. The ^{13}C NMR spectrum of compound **7** in CD_3OD (150 MHz) 59
Figure S5-58. The DEPT 135 spectrum of compound **7** in CD_3OD (150 MHz)................ 59
Figure S5-59. The HSQC spectrum of compound **7** in CD_3OD (600 MHz) 60
Figure S5-60. The HMBC spectrum of compound **7** in CD_3OD (600 MHz) 60
Figure S5-61. The ^1H-^1H COSY spectrum of compound **7** in CD_3OD (600 MHz) 61
Figure S5-62. The NOESY spectrum of compound **7** in CD_3OD (600 MHz) 61
Figure S5-63. The ECD spectrum of compound **7** in MeOH 62
Figure S5-64. The HRESIMS spectrum of compound **8** 62
Figure S5-65. The ^1H NMR spectrum of compound **8** in CD_3OD (600 MHz) 63
Figure S5-66. The ^{13}C NMR spectrum of compound **8** in CD_3OD (150 MHz) 63
Figure S5-67. The DEPT 135 spectrum of compound **8** in CD_3OD (150 MHz)................ 64
Figure S5-68. The HSQC spectrum of compound **8** in CD_3OD (600 MHz) 64
Figure S5-69. The HMBC spectrum of compound **8** in CD_3OD (600 MHz) 65
Figure S5-70. The ^1H-^1H COSY spectrum of compound **8** in CD_3OD (600 MHz) 65
Figure S5-71. The NOESY spectrum of compound **8** in CD_3OD (600 MHz) 66
Figure S5-72. The ECD spectrum of compound **8** in MeOH 66
Figure S5-73. The HRESIMS spectrum of compound **9** 67
Figure S5-74. The ^1H NMR spectrum of compound **9** in CD_3OD (600 MHz) 67
Figure S5-75. The ^{13}C NMR spectrum of compound **9** in CD_3OD (150 MHz) 68
Figure S5-76. The DEPT 135 spectrum of compound **9** in CD_3OD (150 MHz)................ 68
Figure S5-77. The HSQC spectrum of compound **9** in CD_3OD (600 MHz) 69
Figure S5-78. The HMBC spectrum of compound **9** in CD_3OD (600 MHz) 69
Figure S5-79. The ^1H-^1H COSY spectrum of compound **9** in CD_3OD (600 MHz) 70
Figure S5-80. The NOESY spectrum of compound **9** in CD_3OD (600 MHz) 70
Figure S5-81. The ECD spectrum of compound **9** in MeOH 71

Figure S5-82. The HRESIMS spectrum of compound **10** 71
Figure S5-83. The ^{1}H NMR spectrum of compound **10** in CD_3OD (600 MHz) 72
Figure S5-84. The ^{13}C NMR spectrum of compound **10** in CD_3OD (150 MHz) 72
Figure S5-85. The DEPT 135 spectrum of compound **10** in CD_3OD (150 MHz)........ 73
Figure S5-86. The HSQC spectrum of compound **10** in CD_3OD (600 MHz) 73
Figure S5-87. The HMBC spectrum of compound **10** in CD_3OD (600 MHz) 74
Figure S5-88. The ^{1}H-^{1}H COSY spectrum of compound **10** in CD_3OD (600 MHz) 74
Figure S5-89. The NOESY spectrum of compound **10** in CD_3OD (600 MHz) 75
Figure S5-90. The NOESY spectrum of compound **10** in DMSO-d_6 (600 MHz)........ 75
Figure S5-91. The ECD spectrum of compound **10** in MeOH 76
Figure S5-92. The HRESIMS spectrum of compound **11** 76
Figure S5-93. The ^{1}H NMR spectrum of compound **11** in CD_3OD (600 MHz) 77
Figure S5-94. The ^{13}C NMR spectrum of compound **11** in CD_3OD (150 MHz) 77
Figure S5-95. The DEPT 135 spectrum of compound **11** in CD_3OD (150 MHz)........ 78
Figure S5-96. The HSQC spectrum of compound **11** in CD_3OD (600 MHz) 78
Figure S5-97. The HMBC spectrum of compound **11** in CD_3OD (600 MHz) 79
Figure S5-98. The ^{1}H-^{1}H COSY spectrum of compound **11** in CD_3OD (600 MHz) 79
Figure S5-99. The NOESY spectrum of compound **11** in CD_3OD (600 MHz) 80
Figure S5-100. The ECD spectrum of compound **11** in MeOH 80
Figure S5-101. Calculated NMR data and DP4+ probability statistics of compound **11**... 82
Figure S5-102. The HRESIMS spectrum of compound **12** 82
Figure S5-103. The ^{1}H NMR spectrum of compound **12** in CD_3OD (600 MHz)........ 83
Figure S5-104. The ^{13}C NMR spectrum of compound **12** in CD_3OD (150 MHz) 83
Figure S5-105. The DEPT 135 spectrum of compound **12** in CD_3OD (150 MHz)........ 84
Figure S5-106. The HSQC spectrum of compound **12** in CD_3OD (600 MHz) 84
Figure S5-107. The HMBC spectrum of compound **12** in CD_3OD (600 MHz) 85
Figure S5-108. The ^{1}H-^{1}H COSY spectrum of compound **12** in CD_3OD (600 MHz) 85
Figure S5-109. The NOESY spectrum of compound **12** in CD_3OD (600 MHz) 86
Figure S5-110. The ECD spectrum of compound **12** in MeOH 86
Figure S5-111. UV full-wavelength scan spectra of compounds **1** – **12** 88
S6. Calculation of ECD Spectra for **1**. 88
Table S6. Energy Analysis for the Conformers of **1**........ 88

Figure S6. B3lyp/6-31g(d) optimized low-energy conformers of **1**. 90
S7. Calculation of ECD Spectra for **2**. 90
Table S7. Energy Analysis for the Conformers of **2**. 91
Figure S7. B3lyp/6-31g(d) optimized low-energy conformers of **2**. 92
S8. Calculation of ECD Spectra for **3**. 92
Table S8. Energy Analysis for the Conformers of **3**. 92
Figure S8. B3lyp/6-31g(d) optimized low-energy conformers of **3**. 96
S9. Calculation of ECD Spectra for **4**. 97
Table S9. Energy Analysis for the Conformers of **4**. 97
Figure S9. M062x/6-311g(d,p) optimized low-energy conformers of **4**. 97
S10. Calculation of ECD Spectra for **5**. 97
Table S10. Energy Analysis for the Conformers of **5**. 98
Figure S10. B3lyp/6-31g(d) optimized low-energy conformers of **5**. 99
S11. Calculation of ECD Spectra for **6**. 99
Table S11. Energy Analysis for the Conformers of **6**. 99
Figure S11. B3lyp/6-31g(d) optimized low-energy conformers of **6**. 100
S12. Calculation of ECD Spectra for **7**. 100
Table S12. Energy Analysis for the Conformers of **7**. 100
Figure S12. B3lyp/6-31g(d) optimized low-energy conformers of **7**. 103
S13. Calculation of ECD Spectra for **8**. 103
Table S13. Energy Analysis for the Conformers of **8**. 104
Figure S13. B3lyp/6-31g(d) optimized low-energy conformers of **8**. 107
S14. Calculation of ECD Spectra for **9**. 107
Table S14. Energy Analysis for the Conformers of **9**. 108
Figure S14. B3lyp/6-31g(d) optimized low-energy conformers of **9**. 109
S15. Calculation of ECD Spectra for **10**. 110
Table S15. Energy Analysis for the Conformers of **10**. 110
Figure S15. B3lyp/6-31g(d) optimized low-energy conformers of **10**. 111
S16. Calculation of ECD Spectra for **11**. 111
Table S16. Energy Analysis for the Conformers of **11**. 111
Figure S16. B3lyp/6-31g(d) optimized low-energy conformers of **11**. 120
S17. Calculation of ECD Spectra for **12**. 120

Table S17. Energy Analysis for the Conformers of **12**. 120

Figure S17. M062x/6-311g(d,p) optimized low-energy conformers of **12**. 122

S18. UPLC-Q-TOF-MS/MS results of *S. officinalis* 122

Figure S18. UPLC-Q-TOF-MS/MS results of *S. officinalis* 122

Table S18. Elemental constituents of major product ions for patchoulol-type sesquiterpenoids (compounds **3−5**, **14**, **15** and **19**). 122

S19. Inhibition rate of melanin production in B16F10 cells 123

Table S19. Melanogenesis levels of compounds **1−37** in IBMX-stimulated B16F10 melanoma cells. 123

S20. Results of B16F10 cells viability assay 124

Figure S20. The compounds **2−6**, **8−14**, **16**, **17**, **19**, **21−23** and **25−36** were treated at 50 μM for 48 h (except for compound **5** at 6.25 μM and **27** at 25 μM) on B16F10 cells, the cytotoxicity was calculated compared with control group by CCK-8 assay. 124

S21. General experimental procedures 125

S22. Extraction and isolation of the known compounds **13−37** 126

S1. Crystal data for **1**

Table S1-1 Crystal data and structure refinement for **1**.

Identification code	**1**
Empirical formula	$C_{15}H_{26}O_3$
Formula weight	254.36
Temperature/K	150
Crystal system	trigonal
Space group	$P3_1$
a/Å	7.7907(6)
b/Å	7.7907(6)
c/Å	19.532(2)
α/°	90
β/°	90
γ/°	120
Volume/Å^3	1026.7(2)
Z	3
ρ_{calc}g/cm^3	1.234
μ/mm^{-1}	0.666
F(000)	420.0
Crystal size/mm^3	0.15 × 0.08 × 0.05
Radiation	CuKα (λ = 1.54178)
2Θ range for data collection/°	13.886 to 148.842
Index ranges	$-9 \leq h \leq 9, -9 \leq k \leq 9, -23 \leq l \leq 24$
Reflections collected	13376
Independent reflections	2764 [R_{int} = 0.0738, R_{sigma} = 0.0485]
Data/restraints/parameters	2764/1/172
Goodness-of-fit on F^2	1.113
Final R indexes [I>=2σ (I)]	R_1 = 0.0384, wR_2 = 0.0992
Final R indexes [all data]	R_1 = 0.0392, wR_2 = 0.1006

Largest diff. peak/hole / e $Å^{-3}$	0.20/-0.28
Flack parameter	0.05(11)

Table S1-2 Fractional Atomic Coordinates ($\times10^4$) and Equivalent Isotropic Displacement Parameters ($Å^2\times10^3$) for **1**. U_{eq} is defined as 1/3 of of the trace of the orthogonalised U_{IJ} tensor.

**Atom**	***x***	***y***	***z***	**U(eq)**
O1	7060(2)	5620(2)	6297.0(7)	25.8(3)
O3	5033(2)	108(2)	3560.4(8)	27.0(3)
O2	3400(3)	5304(3)	6442.0(8)	32.6(4)
C3	7060(3)	3310(3)	4603.4(11)	24.2(4)
C12	4198(3)	1270(3)	3792.1(11)	26.8(4)
C5	3786(3)	3167(3)	4761.4(11)	21.4(4)
C13	3596(3)	1900(3)	5983.6(11)	28.1(5)
C2	7653(3)	4316(3)	5306.0(10)	23.1(4)
C4	4805(3)	2021(3)	4522.8(11)	22.5(4)
C10	4208(3)	3727(3)	5532.0(10)	20.8(4)
C11	6908(3)	7280(3)	5222.1(12)	24.2(4)
C9	2936(3)	4648(3)	5745.6(12)	27.1(5)
C14	8735(3)	8162(3)	4748.6(13)	32.3(5)
C1	6464(3)	5265(3)	5585.0(10)	20.2(4)
C8	3275(3)	6344(4)	5259.4(12)	32.1(5)
C6	4423(3)	5068(3)	4327.1(11)	25.5(5)
C7	5057(3)	6862(3)	4798.5(12)	27.2(5)
C15	7345(4)	8922(3)	5751.0(14)	32.7(5)

Table S1-3 Anisotropic Displacement Parameters ($Å^2\times10^3$) for **1**. The Anisotropic displacement factor exponent takes the form: $-2\pi^2[h^2a^{*2}U_{11}+2hka^*b^*U_{12}+\ldots]$.

**Atom**	U_{11}	U_{22}	U_{33}	U_{23}	U_{13}	U_{12}

O1	25.8(7)	32.7(8)	20.1(8)	-5.5(6)	-2.5(6)	15.5(6)
O3	28.9(7)	21.6(7)	23.8(8)	-3.2(6)	0.3(6)	7.7(6)
O2	24.9(7)	43.2(10)	29.7(9)	-8.0(7)	4.7(6)	17.1(7)
C3	23.5(10)	28.6(10)	22.0(10)	-5.1(8)	-2.4(7)	14.1(8)
C12	27.7(10)	26.6(10)	22.0(10)	-4.0(8)	-4.5(8)	10.6(8)
C5	17.5(9)	21.8(9)	21.9(9)	0.0(8)	-0.7(7)	7.4(7)
C13	29.6(11)	25.2(10)	22.1(10)	2.0(8)	2.6(8)	8.1(8)
C2	20.3(9)	28.4(10)	23.2(10)	-5.4(8)	-3.5(7)	14.1(8)
C4	24.5(10)	21.1(9)	19.8(10)	-0.7(7)	-0.5(7)	9.8(8)
C10	16.6(9)	21.3(9)	21.6(10)	0.5(7)	1.4(7)	7.2(7)
C11	22.0(9)	20.6(9)	26.3(10)	-0.8(8)	2.3(8)	8.0(8)
C9	19.5(9)	33.2(11)	27.3(11)	-2.6(8)	1.9(8)	12.2(8)
C14	28.1(11)	25.3(10)	32.1(12)	1.1(9)	7.6(9)	4.9(9)
C1	19.1(9)	22.8(9)	17.9(10)	-2.9(7)	-1.1(7)	10.0(7)
C8	30.3(11)	37.1(12)	36.3(14)	-0.7(10)	-0.5(10)	22.3(10)
C6	26.1(10)	27.0(10)	24.2(11)	0.3(8)	-4.3(8)	13.9(8)
C7	30.5(11)	26.2(10)	28.7(11)	2.3(9)	0.2(9)	17.0(9)
C15	33.3(12)	23.0(10)	37.6(13)	-5.2(9)	2.0(9)	10.9(9)

Table S1-4 Bond Lengths for **1**.

**Atom**	**Atom**	**Length/Å**	**Atom**	**Atom**	**Length/Å**
O1	C1	1.448(2)	C2	C1	1.545(3)
O3	C12	1.428(3)	C10	C9	1.543(3)
O2	C9	1.434(3)	C10	C1	1.559(3)
C3	C2	1.533(3)	C11	C14	1.542(3)
C3	C4	1.535(3)	C11	C1	1.595(3)
C12	C4	1.525(3)	C11	C7	1.549(3)
C5	C4	1.534(3)	C11	C15	1.544(3)
C5	C10	1.556(3)	C9	C8	1.539(3)

C5	C6	1.557(3)	C8	C7	1.530(3)
C13	C10	1.534(3)	C6	C7	1.535(3)

Table S1-5 Bond Angles for **1**.

Atom	Atom	Atom	Angle/°	Atom	Atom	Atom	Angle/°
C2	C3	C4	112.36(16)	C7	C11	C1	108.05(15)
O3	C12	C4	112.80(17)	C15	C11	C1	111.57(18)
C4	C5	C10	111.05(16)	C15	C11	C7	109.14(18)
C4	C5	C6	112.12(17)	O2	C9	C10	108.43(17)
C10	C5	C6	109.75(16)	O2	C9	C8	111.99(19)
C3	C2	C1	117.45(16)	C8	C9	C10	110.97(17)
C12	C4	C3	112.41(17)	O1	C1	C2	102.65(15)
C12	C4	C5	110.86(17)	O1	C1	C10	109.94(15)
C5	C4	C3	110.13(16)	O1	C1	C11	110.34(15)
C5	C10	C1	106.19(15)	C2	C1	C10	108.92(15)
C13	C10	C5	111.83(17)	C2	C1	C11	115.31(16)
C13	C10	C9	107.56(17)	C10	C1	C11	109.45(16)
C13	C10	C1	112.51(17)	C7	C8	C9	109.45(17)
C9	C10	C5	107.29(16)	C7	C6	C5	110.11(17)
C9	C10	C1	111.37(16)	C8	C7	C11	111.57(18)
C14	C11	C1	113.77(18)	C8	C7	C6	106.06(17)
C14	C11	C7	109.16(18)	C6	C7	C11	110.70(17)
C14	C11	C15	105.05(17)				

Table S1-6 Torsion Angles for **1**.

A	B	C	D	Angle/°	A	B	C	D	Angle/°
O3	C12	C4	C3	59.3(2)	C10	C9	C8	C7	-10.9(2)
O3	C12	C4	C5	-177.01(16)	C9	C10	C1	O1	73.1(2)
O2	C9	C8	C7	110.5(2)	C9	C10	C1	C2	-175.11(16)

C3	C2	C1	O1	166.66(17)	C9	C10	C1	C11	-48.2(2)
C3	C2	C1	C10	50.1(2)	C9	C8	C7	C11	-52.9(2)
C3	C2	C1	C11	-73.3(2)	C9	C8	C7	C6	67.8(2)
C5	C10	C9	O2	-176.05(16)	C14	C11	C1	O1	104.5(2)
C5	C10	C9	C8	-52.7(2)	C14	C11	C1	C2	-11.2(2)
C5	C10	C1	O1	-170.38(15)	C14	C11	C1	C10	-134.40(18)
C5	C10	C1	C2	-58.6(2)	C14	C11	C7	C8	-169.58(18)
C5	C10	C1	C11	68.27(19)	C14	C11	C7	C6	72.6(2)
C5	C6	C7	C11	63.5(2)	C1	C10	C9	O2	-60.2(2)
C5	C6	C7	C8	-57.7(2)	C1	C10	C9	C8	63.2(2)
C13	C10	C9	O2	63.5(2)	C1	C11	C7	C8	66.2(2)
C13	C10	C9	C8	-173.13(17)	C1	C11	C7	C6	-51.6(2)
C13	C10	C1	O1	-47.7(2)	C6	C5	C4	C3	61.7(2)
C13	C10	C1	C2	64.0(2)	C6	C5	C4	C12	-63.3(2)
C13	C10	C1	C11	-169.07(17)	C6	C5	C10	C13	179.71(17)
C2	C3	C4	C12	171.42(18)	C6	C5	C10	C9	62.0(2)
C2	C3	C4	C5	47.3(2)	C6	C5	C10	C1	-57.2(2)
C4	C3	C2	C1	-44.0(3)	C7	C11	C1	O1	-134.13(16)
C4	C5	C10	C13	-55.8(2)	C7	C11	C1	C2	110.17(19)
C4	C5	C10	C9	-173.48(16)	C7	C11	C1	C10	-13.0(2)
C4	C5	C10	C1	67.3(2)	C15	C11	C1	O1	-14.1(2)
C4	C5	C6	C7	-129.94(18)	C15	C11	C1	C2	-129.84(19)
C10	C5	C4	C3	-61.5(2)	C15	C11	C1	C10	106.97(19)
C10	C5	C4	C12	173.51(16)	C15	C11	C7	C8	-55.3(2)
C10	C5	C6	C7	-6.0(2)	C15	C11	C7	C6	-173.14(18)

Table S1-7 Hydrogen Atom Coordinates (Å×10^4) and Isotropic Displacement Parameters (Å^2×10^3) for **1**.

**Atom**	***x***	***y***	***z***	**U(eq)**
H1	6177.89	5673.35	6527.9	39
H3	6163.58	855.86	3395.95	40
H3A	7673.11	2473.17	4539.8	29
H3B	7579.9	4338.7	4242.04	29
H12A	2734.78	461.54	3768.12	32
H12B	4624.38	2418.71	3482.13	32
H5	2323.99	2281.12	4705.3	26
H13A	2260.95	854.66	5855.91	42
H13B	3603.47	2257.5	6465.09	42
H13C	4532.74	1421.06	5918.32	42
H2A	9065.86	5361.11	5283.61	28
H2B	7549.24	3318.42	5642.74	28
H4	4348.78	840.45	4827.6	27
H9	1507.85	3596.5	5720.94	32
H14A	9911.22	8433.63	5014.63	48
H14B	8928.66	9398.76	4547.02	48
H14C	8527.75	7215.89	4383.03	48
H8A	2079.46	5933.57	4975.34	39
H8B	3522.13	7522.55	5528.97	39
H6A	5539.18	5305.87	4023.88	31
H6B	3302.77	4883.1	4035.96	31
H7	5364.17	8053.49	4516.49	33
H15A	8599.61	9299.94	5983.79	49
H15B	6271.48	8428.6	6088.35	49
H15C	7443.1	10079.61	5516.51	49
H2	2420(60)	5240(50)	6604(19)	40(8)

S2. Crystal data for **3**

Table S2-1 Crystal data and structure refinement for **3**.

Identification code	**3**
Empirical formula	$C_{15}H_{26}O_3$
Formula weight	254.36
Temperature/K	100
Crystal system	hexagonal
Space group	$P6_5$
a/Å	7.55240(10)
b/Å	7.55240(10)
c/Å	41.4232(8)
α/°	90
β/°	90
γ/°	120
Volume/Å^3	2046.18(7)
Z	6
ρ_{calc}g/cm^3	1.239
μ/mm^{-1}	0.668
F(000)	840.0
Crystal size/mm^3	0.12 × 0.06 × 0.04
Radiation	CuKα (λ = 1.54178)
2Θ range for data collection/°	13.538 to 144.21
Index ranges	$-9 \leq h \leq 9$, $-9 \leq k \leq 9$, $-49 \leq l \leq 49$
Reflections collected	17665
Independent reflections	2686 [R_{int} = 0.0822, R_{sigma} = 0.0447]
Data/restraints/parameters	2686/1/168
Goodness-of-fit on F^2	1.057
Final R indexes [I>=2σ (I)]	R_1 = 0.0355, wR_2 = 0.0883

Final R indexes [all data]	R_1 = 0.0370, wR_2 = 0.0897
Largest diff. peak/hole / e Å^{-3}	0.18/-0.22
Flack parameter	0.10(11)

Table S2-2 Fractional Atomic Coordinates (×10^4) and Equivalent Isotropic Displacement Parameters (Å^2×10^3) for **3**. U_{eq} is defined as 1/3 of of the trace of the orthogonalised U_{IJ} tensor.

**Atom**	***x***	***y***	***z***	**U(eq)**
O2	11191(2)	7359(3)	5577.3(4)	20.5(4)
O1	2949(2)	7137(3)	5008.6(4)	19.5(4)
O3	3144(3)	8611(3)	4419.9(4)	27.5(4)
C12	10820(3)	8987(3)	5501.5(6)	18.3(5)
C1	5070(3)	7664(3)	5020.3(5)	14.0(4)
C4	8566(3)	8283(3)	5441.0(5)	14.5(4)
C3	8353(3)	10124(3)	5345.4(5)	16.1(4)
C10	5253(3)	5837(3)	5154.6(5)	14.9(4)
C7	6970(3)	6868(3)	4588.8(5)	16.5(4)
C15	4051(4)	7342(4)	4420.2(6)	21.1(5)
C2	6158(3)	9506(3)	5251.9(5)	16.8(5)
C14	7312(4)	10357(4)	4575.3(6)	20.0(5)
C9	4286(4)	4030(3)	4914.5(6)	20.8(5)
C5	7567(3)	6585(3)	5184.6(5)	14.0(4)
C11	5870(3)	8094(3)	4656.9(5)	15.3(4)
C6	8587(3)	7275(3)	4847.0(5)	16.3(5)
C8	5473(4)	4565(4)	4591.1(6)	22.4(5)
C13	4128(3)	5039(4)	5478.5(6)	20.2(5)

Table S2-3 Anisotropic Displacement Parameters (Å^2×10^3) for **3**. The Anisotropic displacement factor exponent takes the form: $-2\pi^2[h^2a^{*2}U_{11}+2hka^*b^*U_{12}+\ldots]$.

Atom	U_{11}	U_{22}	U_{33}	U_{23}	U_{13}	U_{12}
O2	19.2(8)	30.5(9)	16.4(8)	5.6(7)	3.0(6)	15.9(7)
O1	14.6(8)	33.1(9)	14.7(8)	4.0(7)	2.4(6)	15.0(7)
O3	28.8(9)	46.4(11)	19.5(9)	11.5(8)	4.8(7)	27.8(9)
C12	15.5(10)	21.2(11)	17.7(11)	3.2(9)	0.0(8)	8.9(9)
C1	11.6(10)	19.0(11)	14.1(10)	2.2(8)	1.3(8)	9.7(9)
C4	12.3(10)	16.7(10)	13.6(10)	1.7(8)	1.1(8)	6.4(9)
C3	18.1(11)	16.0(11)	14.4(11)	-1.0(8)	0.7(8)	8.6(9)
C10	14.0(10)	15.0(10)	14.8(11)	2.0(8)	-0.4(8)	6.6(9)
C7	19.4(11)	20.1(11)	12.5(11)	-0.9(8)	0.8(8)	11.8(9)
C15	21.5(12)	31.0(13)	15.4(11)	3.5(9)	-0.5(9)	16.6(10)
C2	19.5(11)	18.1(11)	16.2(11)	1.7(8)	2.5(8)	12.2(9)
C14	25.9(12)	19.4(11)	16.4(11)	6.1(9)	4.8(9)	12.6(10)
C9	21.7(12)	15.5(11)	21.9(12)	0.9(9)	-2.6(9)	6.8(9)
C5	15.0(10)	14.5(10)	15.2(11)	1.4(8)	-0.2(8)	9.5(9)
C11	16.7(10)	19.2(11)	11.5(11)	3.1(8)	2.9(8)	10.1(9)
C6	16.1(11)	20.1(11)	16.0(11)	1.2(8)	1.6(8)	11.4(9)
C8	27.5(12)	20.6(12)	19.8(12)	-3.8(9)	-3.1(9)	12.6(10)
C13	15.3(10)	22.0(11)	18.4(12)	7.4(9)	2.0(9)	5.6(9)

Table S2-4 Bond Lengths for **3**.

Atom	Atom	Length/Å	Atom	Atom	Length/Å
O2	C12	1.426(3)	C10	C9	1.546(3)
O1	C1	1.446(3)	C10	C5	1.549(3)
O3	C15	1.430(3)	C10	C13	1.540(3)
C12	C4	1.529(3)	C7	C11	1.548(3)
C1	C10	1.557(3)	C7	C6	1.534(3)
C1	C2	1.545(3)	C7	C8	1.529(3)
C1	C11	1.593(3)	C15	C11	1.546(3)

C4	C3	1.529(3)	C14	C11	1.537(3)
C4	C5	1.541(3)	C9	C8	1.549(4)
C3	C2	1.531(3)	C5	C6	1.555(3)

Table S2-5 Bond Angles for **3**.

Atom	Atom	Atom	Angle/°	Atom	Atom	Atom	Angle/°
O2	C12	C4	113.64(18)	C6	C7	C11	111.36(18)
O1	C1	C10	110.19(17)	C8	C7	C11	111.50(19)
O1	C1	C2	106.60(17)	C8	C7	C6	106.61(19)
O1	C1	C11	106.24(16)	O3	C15	C11	113.0(2)
C10	C1	C11	108.99(16)	C3	C2	C1	116.00(18)
C2	C1	C10	109.47(17)	C10	C9	C8	111.72(19)
C2	C1	C11	115.24(18)	C4	C5	C10	110.64(18)
C12	C4	C5	113.52(18)	C4	C5	C6	112.74(18)
C3	C4	C12	109.33(18)	C10	C5	C6	109.25(17)
C3	C4	C5	110.34(18)	C7	C11	C1	108.22(17)
C4	C3	C2	112.25(18)	C15	C11	C1	110.33(17)
C9	C10	C1	109.90(18)	C15	C11	C7	107.99(18)
C9	C10	C5	108.82(18)	C14	C11	C1	114.83(18)
C5	C10	C1	106.80(17)	C14	C11	C7	109.36(18)
C13	C10	C1	112.47(18)	C14	C11	C15	105.91(18)
C13	C10	C9	106.88(18)	C7	C6	C5	110.38(17)
C13	C10	C5	111.95(17)	C7	C8	C9	107.72(19)

Table S2-6 Torsion Angles for **3**.

A	B	C	D	Angle/°	A	B	C	D	Angle/°
O2	C12	C4	C3	-177.13(18)	C2	C1	C10	C9	-176.56(17)
O2	C12	C4	C5	-53.5(3)	C2	C1	C10	C5	-58.7(2)
O1	C1	C10	C9	66.5(2)	C2	C1	C10	C13	64.5(2)

O1	C1	C10	C5	-175.60(17)	C2	C1	C11	C7	111.6(2)
O1	C1	C10	C13	-52.4(2)	C2	C1	C11	C15	-130.5(2)
O1	C1	C2	C3	170.51(18)	C2	C1	C11	C14	-10.9(3)
O1	C1	C11	C7	-130.64(18)	C9	C10	C5	C4	-175.77(17)
O1	C1	C11	C15	-12.7(2)	C9	C10	C5	C6	59.5(2)
O1	C1	C11	C14	106.88(19)	C5	C4	C3	C2	49.4(2)
O3	C15	C11	C1	72.6(2)	C5	C10	C9	C8	-51.7(2)
O3	C15	C11	C7	-169.32(18)	C11	C1	C10	C9	-49.7(2)
O3	C15	C11	C14	-52.2(2)	C11	C1	C10	C5	68.2(2)
C12	C4	C3	C2	174.92(18)	C11	C1	C10	C13	-168.62(18)
C12	C4	C5	C10	175.74(17)	C11	C1	C2	C3	-71.9(2)
C12	C4	C5	C6	-61.6(2)	C11	C7	C6	C5	61.7(2)
C1	C10	C9	C8	64.9(2)	C11	C7	C8	C9	-53.5(2)
C1	C10	C5	C4	65.6(2)	C6	C7	C11	C1	-51.7(2)
C1	C10	C5	C6	-59.0(2)	C6	C7	C11	C15	-171.10(18)
C4	C3	C2	C1	-46.4(3)	C6	C7	C11	C14	74.1(2)
C4	C5	C6	C7	-127.2(2)	C6	C7	C8	C9	68.2(2)
C3	C4	C5	C10	-61.1(2)	C8	C7	C11	C1	67.3(2)
C3	C4	C5	C6	61.5(2)	C8	C7	C11	C15	-52.2(2)
C10	C1	C2	C3	51.3(2)	C8	C7	C11	C14	-166.97(19)
C10	C1	C11	C7	-11.9(2)	C8	C7	C6	C5	-60.2(2)
C10	C1	C11	C15	106.0(2)	C13	C10	C9	C8	-172.74(19)
C10	C1	C11	C14	-134.4(2)	C13	C10	C5	C4	-57.9(2)
C10	C9	C8	C7	-11.4(3)	C13	C10	C5	C6	177.45(18)
C10	C5	C6	C7	-3.7(2)					

Table S2-7 Hydrogen Atom Coordinates (Å×10^4) and Isotropic Displacement Parameters (Å^2×10^3) for **3**.

**Atom**	***x***	***y***	***z***	**U(eq)**
H2	10558.89	6772.49	5746.74	31
H1	2502.97	7040.01	5197.38	29
H3	2674.74	8599.86	4604.22	41
H12A	11616.04	9703.12	5306.63	22
H12B	11319.29	9980.73	5681.78	22
H4	7813.18	7730.92	5648.7	17
H3A	9271.13	10832.43	5160.87	19
H3B	8790.13	11095.74	5528.42	19
H7	7648.82	7271.4	4372.84	20
H15A	4531.37	7302.05	4199.42	25
H15B	2999.06	5931.99	4479.53	25
H2A	6171.24	10699.19	5150.04	20
H2B	5335.93	9186.35	5451.91	20
H14A	6678.47	11159.86	4640.95	30
H14B	7569.33	10505.01	4342.24	30
H14C	8608.21	10850.75	4690.59	30
H9A	2850.99	3660.02	4872.36	25
H9B	4276.45	2828.71	5012.08	25
H5	7717.53	5404.14	5256.76	17
H6A	9609.3	8752.66	4852.06	20
H6B	9304.73	6519.96	4792.27	20
H8A	6224.39	3807.98	4571.44	27
H8B	4511.49	4182.13	4407.32	27
H13A	4541.78	6187.24	5627.61	30
H13B	4478.35	4059.81	5571.42	30
H13C	2649.22	4362.02	5442.47	30

S3. Crystal data for **5**

Table S3-1 Crystal data and structure refinement for **5**.

Identification code	**5**
Empirical formula	$C_{14}H_{24}O_2$
Formula weight	224.33
Temperature/K	100
Crystal system	orthorhombic
Space group	$P2_12_12$
a/Å	14.9889(3)
b/Å	21.4839(4)
c/Å	7.5409(2)
α/°	90
β/°	90
γ/°	90
Volume/Å^3	2428.32(9)
Z	8
ρ_{calc}g/cm^3	1.227
μ/mm^{-1}	0.620
F(000)	992.0
Crystal size/mm^3	0.08 × 0.05 × 0.04
Radiation	CuKα (λ = 1.54178)
2Θ range for data collection/°	7.19 to 149.412
Index ranges	$-18 \leq h \leq 18$, $-26 \leq k \leq 26$, $-9 \leq l \leq 9$
Reflections collected	46814
Independent reflections	4961 [R_{int} = 0.0776, R_{sigma} = 0.0368]
Data/restraints/parameters	4961/1/302
Goodness-of-fit on F^2	1.038
Final R indexes [I>=2σ (I)]	R_1 = 0.0393, wR_2 = 0.0974

Final R indexes [all data]	$R_1 = 0.0417$, $wR_2 = 0.0999$
Largest diff. peak/hole / e Å^{-3}	0.23/-0.22
Flack parameter	0.00(8)

Table S3-2 Fractional Atomic Coordinates (×10^4) and Equivalent Isotropic Displacement Parameters (Å^2×10^3) for **5**. U_{eq} is defined as 1/3 of of the trace of the orthogonalised U_{IJ} tensor.

**Atom**	***x***	***y***	***z***	**U(eq)**
O001	5237.6(10)	5846.7(7)	9828(2)	22.9(3)
O1	3049.9(10)	4587.6(7)	8563.3(19)	21.4(3)
O2	3593.9(10)	5323.7(7)	1453.0(19)	21.7(3)
O004	4977.3(13)	8329.6(8)	12048(3)	32.9(4)
C4	3592.5(14)	4992.7(10)	3114(3)	18.2(4)
C12	3342.0(15)	3846.4(10)	5411(3)	22.1(4)
C1	2921.7(14)	4889.5(10)	6860(3)	17.7(4)
C10	2686.3(14)	4396.5(10)	5415(3)	17.8(4)
C9	1756.0(14)	4123.7(10)	5781(3)	22.3(4)
C2	3823.2(13)	5196.2(10)	6405(3)	18.9(4)
C3	3931.9(14)	5444.9(10)	4506(3)	19.4(4)
C00C	5112.0(14)	6426.1(9)	10756(3)	19.6(4)
C00D	5973.6(14)	6794.8(10)	10677(3)	21.4(4)
C7	1468.7(13)	5258.5(10)	5488(3)	18.9(4)
C00F	4341.1(14)	6798.6(10)	9941(3)	18.5(4)
C13	2365.9(15)	6044.7(10)	7186(3)	25.2(5)
C00H	4528.9(14)	6973.2(10)	7970(3)	20.7(4)
C8	1015.7(14)	4620.7(10)	5601(3)	22.4(4)
C00J	5001.9(15)	7813.7(10)	10822(3)	21.6(4)
C11	2103.6(13)	5352.3(10)	7082(3)	19.5(4)
C00L	4380.2(15)	7674.2(10)	7707(3)	23.1(5)

C00M	3324.6(15)	7716.5(11)	10238(3)	25.2(5)
C6	1959.3(14)	5279.4(10)	3708(3)	19.3(4)
C00O	4156.3(14)	7394.6(10)	11032(3)	20.8(4)
C5	2663.5(13)	4746.6(10)	3609(3)	17.3(4)
C00Q	3409.0(15)	7801.2(11)	8216(3)	25.9(5)
C00R	3936.3(16)	7249.8(12)	12974(3)	27.8(5)
C14	1585.0(15)	5223.7(12)	8805(3)	27.6(5)
C00T	5840.0(15)	7450.0(10)	11432(3)	22.7(5)
C00U	5033.0(16)	8069.2(10)	8839(3)	24.1(5)
C00V	5968.3(17)	8056.8(12)	8013(4)	32.5(5)
C00W	4729.8(19)	8756.3(11)	8755(4)	35.9(6)

Table S3-3 Anisotropic Displacement Parameters (Å^2×10^3) for **5**. The Anisotropic displacement factor exponent takes the form: $-2\pi^2[h^2a^{*2}U_{11}+2hka^*b^*U_{12}+\ldots]$.

**Atom**	**U_{11}**	**U_{22}**	**U_{33}**	**U_{23}**	**U_{13}**	**U_{12}**
O001	22.1(7)	16.9(7)	29.8(8)	-2.7(6)	0.6(6)	1.3(6)
O1	18.0(7)	29.4(8)	16.9(7)	3.3(6)	0.0(6)	2.8(6)
O2	23.4(7)	25.4(8)	16.2(7)	2.4(6)	1.3(6)	-1.6(6)
O004	30.8(9)	26.4(8)	41.5(10)	-17.5(8)	-5.3(8)	1.4(7)
C4	16.9(9)	21.3(9)	16.6(9)	2.2(8)	0.5(7)	-0.8(8)
C12	24.0(10)	19.5(10)	22.7(10)	0.6(8)	1.4(9)	3.0(8)
C1	16.4(10)	22.9(11)	13.8(9)	2.1(8)	0.0(7)	1.6(8)
C10	16.4(9)	18.6(9)	18.5(9)	-0.3(8)	0.3(8)	-0.1(8)
C9	20.5(10)	21.1(10)	25.4(10)	1.0(9)	1.2(8)	-3.1(8)
C2	13.4(9)	24.1(10)	19.1(9)	-0.6(8)	-0.6(8)	0.7(7)
C3	14.1(9)	22.7(10)	21.5(10)	-0.2(8)	0.0(8)	-1.3(8)
C00C	19.1(10)	18.6(9)	20.9(9)	-1.2(8)	-0.4(8)	0.4(8)
C00D	16.6(10)	23.1(10)	24.4(10)	0.2(9)	-3.1(8)	1.9(8)
C7	14.0(9)	23.1(10)	19.6(10)	-0.5(8)	0.3(8)	2.8(8)

C00F	17.6(9)	20.0(10)	18.0(9)	-0.8(8)	-0.9(8)	-0.7(8)
C13	21.7(11)	24.4(11)	29.5(11)	-7.6(9)	-2.3(9)	3.0(8)
C00H	19.3(10)	22.8(10)	20.1(10)	-1.5(9)	-1.6(8)	-1.0(8)
C8	14.3(9)	27.6(11)	25.2(10)	-0.9(9)	-0.9(8)	-2.9(8)
C00J	21.0(10)	19.2(10)	24.5(10)	-3.8(8)	-3.8(9)	0.4(8)
C11	15.5(9)	23.9(10)	19.2(9)	-2.9(9)	1.0(8)	2.7(8)
C00L	23.5(11)	23.3(11)	22.7(10)	2.9(9)	-5.0(9)	-1.3(8)
C00M	18.7(10)	25.4(11)	31.7(11)	-4.0(9)	-3.2(9)	5.6(8)
C6	16.9(9)	21.7(10)	19.2(9)	-0.3(8)	-1.9(8)	2.2(8)
C00O	19.1(10)	21.5(10)	21.7(10)	-3.4(9)	-0.5(8)	2.4(8)
C5	16.0(9)	19.8(9)	16.1(9)	-0.7(8)	-0.9(7)	0.1(8)
C00Q	23.7(11)	22.9(11)	31.1(12)	0.3(9)	-8.4(9)	1.6(9)
C00R	25.5(11)	33.6(12)	24.5(11)	-4.6(10)	1.4(9)	3.9(10)
C14	21.0(11)	41.0(13)	20.8(11)	-2.3(10)	2.5(8)	6.9(9)
C00T	18.5(10)	22.5(10)	27.1(11)	-3.4(9)	-3.3(9)	0.1(8)
C00U	22.7(11)	20.9(10)	28.8(11)	3.0(9)	-6.9(9)	-2.9(9)
C00V	28.1(12)	34.9(13)	34.4(13)	7.3(11)	-4.1(11)	-10.3(10)
C00W	41.9(14)	20.8(11)	45.1(15)	5.6(11)	-17.9(12)	-1.7(10)

Table S3-4 Bond Lengths for **5**.

**Atom**	**Atom**	**Length/Å**	**Atom**	**Atom**	**Length/Å**
O001	C00C	1.440(2)	C7	C11	1.546(3)
O1	C1	1.452(2)	C7	C6	1.531(3)
O2	C4	1.440(2)	C00F	C00H	1.558(3)
O004	C00J	1.444(3)	C00F	C00O	1.547(3)
C4	C3	1.518(3)	C13	C11	1.541(3)
C4	C5	1.536(3)	C00H	C00L	1.535(3)
C12	C10	1.537(3)	C00J	C00O	1.563(3)
C1	C10	1.560(3)	C00J	C00T	1.549(3)

C1	C2	1.542(3)	C00J	C00U	1.594(3)
C1	C11	1.588(3)	C11	C14	1.539(3)
C10	C9	1.537(3)	C00L	C00Q	1.530(3)
C10	C5	1.556(3)	C00L	C00U	1.551(3)
C9	C8	1.546(3)	C00M	C00O	1.546(3)
C2	C3	1.536(3)	C00M	C00Q	1.541(3)
C00C	C00D	1.516(3)	C6	C5	1.559(3)
C00C	C00F	1.534(3)	C00O	C00R	1.534(3)
C00D	C00T	1.532(3)	C00U	C00V	1.534(4)
C7	C8	1.532(3)	C00U	C00W	1.546(3)

Table S3-5 Bond Angles for **5**.

Atom	Atom	Atom	Angle/°	Atom	Atom	Atom	Angle/°
O2	C4	C3	106.57(17)	O004	C00J	C00U	109.71(17)
O2	C4	C5	112.51(16)	C00O	C00J	C00U	108.49(17)
C3	C4	C5	110.85(17)	C00T	C00J	C00O	109.69(17)
O1	C1	C10	110.16(16)	C00T	C00J	C00U	115.37(19)
O1	C1	C2	105.79(16)	C7	C11	C1	108.18(16)
O1	C1	C11	106.80(16)	C13	C11	C1	114.39(17)
C10	C1	C11	108.90(16)	C13	C11	C7	108.83(18)
C2	C1	C10	109.43(16)	C14	C11	C1	111.49(18)
C2	C1	C11	115.64(17)	C14	C11	C7	108.79(17)
C12	C10	C1	112.26(17)	C14	C11	C13	105.01(18)
C12	C10	C5	112.57(17)	C00H	C00L	C00U	111.95(18)
C9	C10	C12	106.69(17)	C00Q	C00L	C00H	106.29(18)
C9	C10	C1	109.80(17)	C00Q	C00L	C00U	111.37(19)
C9	C10	C5	108.74(16)	C00Q	C00M	C00O	111.69(18)
C5	C10	C1	106.74(16)	C7	C6	C5	110.24(17)
C10	C9	C8	111.84(17)	C00F	C00O	C00J	106.13(17)

C3	C2	C1	116.68(17)	C00M	C00O	C00F	107.99(18)
C4	C3	C2	112.72(17)	C00M	C00O	C00J	110.92(18)
O001	C00C	C00D	108.72(17)	C00R	C00O	C00F	112.25(18)
O001	C00C	C00F	110.76(17)	C00R	C00O	C00J	112.83(18)
C00D	C00C	C00F	110.70(17)	C00R	C00O	C00M	106.68(19)
C00C	C00D	C00T	110.74(18)	C4	C5	C10	111.06(16)
C8	C7	C11	110.25(17)	C4	C5	C6	111.89(17)
C6	C7	C8	106.73(17)	C10	C5	C6	109.13(16)
C6	C7	C11	112.45(17)	C00L	C00Q	C00M	107.78(18)
C00C	C00F	C00H	111.82(17)	C00D	C00T	C00J	117.34(18)
C00C	C00F	C00O	110.70(17)	C00L	C00U	C00J	108.03(17)
C00O	C00F	C00H	109.89(17)	C00V	C00U	C00J	113.68(19)
C00L	C00H	C00F	109.48(18)	C00V	C00U	C00L	110.1(2)
C7	C8	C9	107.73(17)	C00V	C00U	C00W	105.6(2)
O004	C00J	C00O	110.90(18)	C00W	C00U	C00J	111.0(2)
O004	C00J	C00T	102.58(17)	C00W	C00U	C00L	108.32(19)

Table S3-6 Torsion Angles for **5**.

**A**	**B**	**C**	**D**	**Angle/°**	**A**	**B**	**C**	**D**	**Angle/°**
O001	C00C	C00D	C00T	172.58(18)	C7	C6	C5	C10	-4.2(2)
O001	C00C	C00F	C00H	-61.8(2)	C00F	C00C	C00D	C00T	50.7(2)
O001	C00C	C00F	C00O	175.34(17)	C00F	C00H	C00L	C00Q	-59.3(2)
O1	C1	C10	C12	-50.1(2)	C00F	C00H	C00L	C00U	62.5(2)
O1	C1	C10	C9	68.4(2)	C00H	C00F	C00O	C00J	-58.2(2)
O1	C1	C10	C5	-173.94(15)	C00H	C00F	C00O	C00M	60.8(2)
O1	C1	C2	C3	168.80(17)	C00H	C00F	C00O	C00R	178.13(18)
O1	C1	C11	C7	-133.05(17)	C00H	C00L	C00Q	C00M	69.7(2)
O1	C1	C11	C13	105.5(2)	C00H	C00L	C00U	C00J	-49.7(2)
O1	C1	C11	C14	-13.5(2)	C00H	C00L	C00U	C00V	75.0(2)

O2	C4	C3	C2	170.45(16)	C00H	C00L	C00U	C00W	-170.0(2)
O2	C4	C5	C10	-179.58(16)	C8	C7	C11	C1	69.0(2)
O2	C4	C5	C6	-57.4(2)	C8	C7	C11	C13	-166.21(17)
O004	C00J	C00O	C00F	-169.15(17)	C8	C7	C11	C14	-52.3(2)
O004	C00J	C00O	C00M	73.8(2)	C8	C7	C6	C5	-59.7(2)
O004	C00J	C00O	C00R	-45.8(2)	C11	C1	C10	C12	-166.95(17)
O004	C00J	C00T	C00D	167.63(19)	C11	C1	C10	C9	-48.5(2)
O004	C00J	C00U	C00L	-136.74(19)	C11	C1	C10	C5	69.3(2)
O004	C00J	C00U	C00V	100.8(2)	C11	C1	C2	C3	-73.2(2)
O004	C00J	C00U	C00W	-18.1(3)	C11	C7	C8	C9	-54.0(2)
C12	C10	C9	C8	-173.28(18)	C11	C7	C6	C5	61.3(2)
C12	C10	C5	C4	-58.2(2)	C6	C7	C8	C9	68.4(2)
C12	C10	C5	C6	177.95(18)	C6	C7	C11	C1	-50.0(2)
C1	C10	C9	C8	64.8(2)	C6	C7	C11	C13	74.8(2)
C1	C10	C5	C4	65.4(2)	C6	C7	C11	C14	-171.30(18)
C1	C10	C5	C6	-58.4(2)	C00O	C00F	C00H	C00L	-5.5(2)
C1	C2	C3	C4	-44.6(2)	C00O	C00J	C00T	C00D	49.7(3)
C10	C1	C2	C3	50.1(2)	C00O	C00J	C00U	C00L	-15.4(2)
C10	C1	C11	C7	-14.1(2)	C00O	C00J	C00U	C00V	-137.9(2)
C10	C1	C11	C13	-135.56(19)	C00O	C00J	C00U	C00W	103.2(2)
C10	C1	C11	C14	105.5(2)	C00O	C00M	C00Q	C00L	-12.8(3)
C10	C9	C8	C7	-11.5(2)	C5	C4	C3	C2	47.7(2)
C9	C10	C5	C4	-176.24(17)	C5	C10	C9	C8	-51.6(2)
C9	C10	C5	C6	59.9(2)	C00Q	C00L	C00U	C00J	69.2(2)
C2	C1	C10	C12	65.8(2)	C00Q	C00L	C00U	C00V	-166.16(19)
C2	C1	C10	C9	-175.74(17)	C00Q	C00L	C00U	C00W	-51.2(3)
C2	C1	C10	C5	-58.0(2)	C00Q	C00M	C00O	C00F	-50.9(2)
C2	C1	C11	C7	109.6(2)	C00Q	C00M	C00O	C00J	65.0(2)
C2	C1	C11	C13	-11.9(3)	C00Q	C00M	C00O	C00R	-171.75(19)

C2	C1	C11	C14	-130.85(19)	C00T	C00J	C00O	C00F	-56.6(2)
C3	C4	C5	C10	-60.4(2)	C00T	C00J	C00O	C00M	-173.61(18)
C3	C4	C5	C6	61.9(2)	C00T	C00J	C00O	C00R	66.8(2)
C00C	C00D	C00T	C00J	-45.9(3)	C00T	C00J	C00U	C00L	108.1(2)
C00C	C00F	C00H	C00L	-128.84(19)	C00T	C00J	C00U	C00V	-14.4(3)
C00C	C00F	C00O	C00J	65.8(2)	C00T	C00J	C00U	C00W	-133.3(2)
C00C	C00F	C00O	C00M	-175.18(18)	C00U	C00J	C00O	C00F	70.3(2)
C00C	C00F	C00O	C00R	-57.9(2)	C00U	C00J	C00O	C00M	-46.8(2)
C00D	C00C	C00F	C00H	58.9(2)	C00U	C00J	C00O	C00R	-166.39(18)
C00D	C00C	C00F	C00O	-64.0(2)	C00U	C00J	C00T	C00D	-73.1(3)
C7	C6	C5	C4	-127.48(18)	C00U	C00L	C00Q	C00M	-52.5(2)

Table S3-7 Hydrogen Atom Coordinates (Å×10^4) and Isotropic Displacement Parameters (Å^2×10^3) for **5**.

**Atom**	***x***	***y***	***z***	**U(eq)**
H001	4819.44	5601.98	10080.06	34
H1	3564.78	4434.12	8609.04	32
H2	3404.16	5088.96	645.82	33
H4	4015.28	4633.89	3029.89	22
H12A	3954.97	4004.29	5363.99	33
H12B	3228.58	3583.77	4372.11	33
H12C	3260.63	3600.29	6492.84	33
H9A	1742.57	3947.89	6993.89	27
H9B	1638.4	3780.82	4935.09	27
H2A	3919.6	5546.12	7236.93	23
H2B	4301.3	4887.58	6622.82	23
H3A	3602.77	5842.51	4395.05	23
H3B	4570.86	5531.28	4282.99	23
H00C	4968.34	6334.82	12024.49	23

H00A	6177.41	6824.49	9431.13	26
H00B	6440.55	6576.1	11365.54	26
H7	1001.59	5590.8	5506.14	23
H00F	3793.18	6533.31	9975.68	22
H13A	2781.72	6107.09	8173.39	38
H13B	1830.24	6297.41	7377.81	38
H13C	2652.76	6169.97	6074.35	38
H00D	5151.36	6864.15	7660.51	25
H00E	4124.84	6735.99	7181.8	25
H8A	657.99	4543.18	4519.87	27
H8B	613.19	4605.02	6641.18	27
H00L	4467.22	7778.74	6426.05	28
H00G	2788.98	7463.23	10502.01	30
H00H	3245.42	8128.77	10803.52	30
H6A	2260.36	5686.75	3572.44	23
H6B	1524.93	5233.06	2728.36	23
H5	2470.67	4446.01	2671.77	21
H00I	3008.21	7507.38	7592.81	31
H00J	3240.21	8230.58	7876.93	31
H00K	3494.48	6914.03	13027.04	42
H00M	3693.48	7622.93	13545.06	42
H00N	4480.4	7119.76	13592.6	42
H14A	1478.69	4775.39	8920.37	41
H14B	1012.34	5443.41	8770.69	41
H14C	1933.92	5370.28	9821.82	41
H00O	6371.78	7701.08	11123.96	27
H00P	5820.81	7417	12740.63	27
H00Q	6377.05	8303.36	8745.04	49
H00R	5944.05	8233.02	6815.57	49

H00S	6181.36	7626.07	7951.86	49
H00T	4164.64	8803.76	9394.11	54
H00U	4647.84	8879.2	7513.93	54
H00V	5184.66	9021.5	9303.03	54
H004	4510(30)	8540(30)	12010(110)	170(30)

S4. Crystal data for **12**

Table S4-1 Crystal data and structure refinement for **12**.

Identification code	**12**
Empirical formula	$C_{15}H_{24}O_2$
Formula weight	236.34
Temperature/K	170
Crystal system	orthorhombic
Space group	$P2_12_12_1$
a/Å	6.26610(10)
b/Å	12.8195(2)
c/Å	35.1835(7)
α/°	90
β/°	90
γ/°	90
Volume/Å^3	2826.23(8)
Z	8
ρ_{calc}g/cm^3	1.111
μ/mm^{-1}	0.558
F(000)	1040.0
Crystal size/mm^3	0.12 × 0.08 × 0.06
Radiation	CuKα (λ = 1.54178)
2Θ range for data collection/°	5.024 to 149.126
Index ranges	$-7 \leq h \leq 7, -15 \leq k \leq 16, -43 \leq l \leq 43$

Reflections collected	44974
Independent reflections	5755 [R_{int} = 0.0850, R_{sigma} = 0.0397]
Data/restraints/parameters	5755/0/315
Goodness-of-fit on F^2	1.043
Final R indexes [I>=2σ (I)]	R_1 = 0.0396, wR_2 = 0.0983
Final R indexes [all data]	R_1 = 0.0444, wR_2 = 0.1026
Largest diff. peak/hole / e Å^{-3}	0.16/-0.18
Flack parameter	0.01(10)

Table S4-2 Fractional Atomic Coordinates (×10^4) and Equivalent Isotropic Displacement Parameters (Å^2×10^3) for **12**. U_{eq} is defined as 1/3 of of the trace of the orthogonalised U_{IJ} tensor.

**Atom**	***x***	***y***	***z***	**U(eq)**
O001	7655(2)	6234.1(11)	5163.2(4)	32.5(3)
O002	3611(2)	6815.5(11)	4533.4(4)	34.6(3)
O1	11093(3)	3864.5(15)	6569.3(5)	51.6(4)
O2	9022(3)	5254.2(17)	5832.0(5)	57.3(5)
C005	5018(3)	6174.4(14)	4484.4(5)	26.5(4)
C006	8441(3)	5860.3(14)	4804.1(6)	28.1(4)
C007	7374(3)	6466.7(14)	4471.8(6)	27.8(4)
C008	5084(3)	4576.2(15)	4056.5(5)	30.8(4)
C009	7951(3)	4691.0(15)	4802.7(6)	31.5(4)
C00A	4492(3)	5025.7(14)	4451.1(5)	28.5(4)
C00B	5552(3)	4466.5(15)	4790.7(6)	30.9(4)
C00C	4951(4)	5405.6(16)	3740.8(6)	34.4(4)
C10	9228(3)	4037.5(18)	6503.3(6)	34.8(5)
C00E	7674(4)	7643.6(15)	4518.4(6)	34.9(4)
C4	6982(4)	3431.5(18)	7143.8(6)	38.2(5)
C5	6080(3)	3034.1(16)	6764.4(6)	33.4(4)

C00H	8367(3)	6117.6(17)	4087.0(6)	34.1(4)
C1	8312(4)	5131.4(16)	6494.3(6)	38.2(5)
C6	7674(3)	3159.3(16)	6431.2(6)	34.9(4)
C00K	5072(4)	3297.5(16)	4788.7(7)	41.3(5)
C7	6624(4)	3320(2)	6039.0(6)	43.5(5)
C2	6582(4)	5237.4(17)	6806.7(7)	45.5(6)
C00N	6869(4)	6176.2(18)	3744.2(6)	40.8(5)
C8	5685(4)	4416(2)	6002.2(6)	46.5(6)
C00P	4665(4)	4889.4(18)	3355.8(6)	43.6(5)
C9	7300(4)	5271(2)	6098.4(7)	45.4(5)
C11	5782(4)	2991(2)	7482.4(7)	49.4(6)
C3	7020(5)	4645(2)	7174.0(7)	50.2(6)
C00T	6435(6)	4207(2)	3215.0(8)	60.2(8)
C15	4947(5)	2486(2)	5956.4(8)	60.2(8)
C14	10070(5)	5936(2)	6560.8(9)	60.4(7)
C13	3424(5)	3031(3)	7470.2(8)	63.9(8)
C00X	2891(6)	5011(3)	3158.7(9)	72.5(9)
C12	6859(6)	2625(3)	7780.3(9)	79.9(11)

Table S4-3 Anisotropic Displacement Parameters (Å^2×10^3) for **12**. The Anisotropic displacement factor exponent takes the form: $-2\pi^2[h^2a^{*2}U_{11}+2hka^*b^*U_{12}+\ldots]$.

**Atom**	**U_{11}**	**U_{22}**	**U_{33}**	**U_{23}**	**U_{13}**	**U_{12}**
O001	34.3(7)	31.2(7)	31.9(7)	-6.9(5)	1.8(6)	-4.5(6)
O002	28.3(7)	31.1(7)	44.3(8)	-8.7(6)	0.7(6)	4.8(6)
O1	28.2(8)	67.7(11)	59.0(10)	12.1(9)	-2.5(7)	-1.7(8)
O2	48.1(10)	80.1(13)	43.8(9)	21.9(9)	5.5(8)	-7.5(10)
C005	27.2(9)	27.0(9)	25.1(8)	-2.7(7)	-0.3(7)	0.6(7)
C006	23.7(9)	28.8(9)	31.9(9)	-3.2(8)	0.0(7)	1.7(7)
C007	26.3(9)	24.0(9)	33.0(10)	-1.2(7)	0.4(7)	-0.5(7)

C008	36.1(10)	27.5(9)	28.9(9)	-3.7(7)	-1.3(8)	-0.3(8)
C009	34.6(10)	26.8(9)	33.1(9)	-2.9(8)	-1.2(8)	3.6(8)
C00A	27.1(10)	26.6(9)	31.7(9)	-4.7(8)	2.9(7)	-1.7(7)
C00B	36.9(11)	26.7(9)	29.1(9)	-3.0(8)	1.9(8)	-1.6(8)
C00C	38.1(11)	32.8(10)	32.2(10)	-1.7(8)	-0.1(8)	5.1(9)
C10	29.8(10)	44.5(12)	29.9(10)	2.3(9)	1.0(8)	-2.4(8)
C00E	34.5(11)	27.0(9)	43.3(11)	-1.3(8)	-2.0(9)	-4.4(8)
C4	34.0(11)	46.5(12)	34.2(10)	4.9(9)	-4.7(8)	-7.9(9)
C5	32.8(10)	31.3(10)	36.1(11)	-1.8(8)	-1.5(8)	-3.8(8)
C00H	30.9(10)	35.2(10)	36.1(11)	-0.3(8)	6.3(8)	-2.2(8)
C1	41.0(12)	33.4(10)	40.3(11)	3.3(9)	-0.2(9)	-5.4(9)
C6	30.3(10)	32.9(10)	41.6(11)	-7.4(9)	1.8(8)	1.2(8)
C00K	50.8(13)	31.1(11)	42.0(11)	5.0(9)	0.4(10)	-7.1(10)
C7	36.2(12)	60.7(14)	33.6(11)	-12.4(10)	3.7(9)	-9.0(11)
C2	57.4(15)	31.0(10)	48.3(13)	-6.2(9)	11.8(11)	-0.6(10)
C00N	53.2(14)	35.9(11)	33.4(11)	3.7(9)	1.3(10)	-3.7(10)
C8	37.7(12)	69.9(16)	31.9(10)	8.2(11)	-2.9(9)	-2.4(11)
C00P	55.8(15)	42.8(12)	32.2(10)	1.6(9)	-3.4(10)	-4.5(11)
C9	43.7(13)	49.6(13)	43.0(12)	15.6(10)	1.4(10)	0.9(11)
C11	49.5(15)	61.0(15)	37.9(12)	8.7(11)	-5.3(11)	-18.5(12)
C3	65.9(17)	49.4(13)	35.2(11)	-9.4(10)	1.3(11)	-18.2(13)
C00T	88(2)	51.4(14)	41.6(13)	-9.8(11)	14.9(14)	1.9(14)
C15	54.7(16)	78(2)	47.9(14)	-25.1(13)	2.1(12)	-21.3(15)
C14	68.8(19)	47.6(14)	64.8(17)	4.0(12)	-1.4(14)	-24.4(14)
C13	50.8(17)	96(2)	44.8(14)	-3.2(15)	6.2(12)	-23.6(16)
C00X	79(2)	87(2)	51.3(15)	2.3(15)	-25.4(16)	-8.3(19)
C12	73(2)	110(3)	57.1(18)	40.4(18)	-14.1(16)	-28(2)

Table S4-4 Bond Lengths for **12**.

Atom	Atom	Length/Å	Atom	Atom	Length/Å
O001	C006	1.438(2)	C10	C6	1.510(3)
O002	C005	1.217(2)	C4	C5	1.536(3)
O1	C10	1.212(3)	C4	C11	1.518(3)
O2	C9	1.430(3)	C4	C3	1.559(3)
C005	C007	1.524(3)	C5	C6	1.549(3)
C005	C00A	1.514(3)	C00H	C00N	1.530(3)
C006	C007	1.555(3)	C1	C2	1.550(3)
C006	C009	1.530(3)	C1	C9	1.541(3)
C007	C00E	1.529(3)	C1	C14	1.527(3)
C007	C00H	1.556(3)	C6	C7	1.542(3)
C008	C00A	1.548(3)	C7	C8	1.529(4)
C008	C00C	1.540(3)	C7	C15	1.527(3)
C009	C00B	1.531(3)	C2	C3	1.524(3)
C00A	C00B	1.544(3)	C8	C9	1.529(4)
C00B	C00K	1.528(3)	C00P	C00T	1.497(4)
C00C	C00N	1.556(3)	C00P	C00X	1.319(4)
C00C	C00P	1.518(3)	C11	C13	1.479(4)
C10	C1	1.516(3)	C11	C12	1.332(4)

Table S4-5 Bond Angles for **12**.

Atom	Atom	Atom	Angle/°	Atom	Atom	Atom	Angle/°
O002	C005	C007	122.66(17)	C11	C4	C3	109.0(2)
O002	C005	C00A	120.67(18)	C4	C5	C6	112.70(17)
C00A	C005	C007	116.61(16)	C00N	C00H	C007	115.23(18)
O001	C006	C007	110.28(15)	C10	C1	C2	109.34(17)
O001	C006	C009	105.10(16)	C10	C1	C9	106.38(18)
C009	C006	C007	113.65(16)	C10	C1	C14	110.4(2)

C005	C007	C006	105.76(15)	C9	C1	C2	110.0(2)
C005	C007	C00E	111.00(16)	C14	C1	C2	109.7(2)
C005	C007	C00H	110.00(16)	C14	C1	C9	110.9(2)
C006	C007	C00H	109.79(15)	C10	C6	C5	111.46(17)
C00E	C007	C006	111.10(16)	C10	C6	C7	109.02(18)
C00E	C007	C00H	109.15(17)	C7	C6	C5	114.57(18)
C00C	C008	C00A	112.13(16)	C8	C7	C6	111.27(18)
C00B	C009	C006	112.42(16)	C15	C7	C6	111.7(2)
C005	C00A	C008	112.29(16)	C15	C7	C8	111.2(2)
C005	C00A	C00B	107.36(15)	C3	C2	C1	115.6(2)
C00B	C00A	C008	114.71(16)	C00H	C00N	C00C	116.66(17)
C009	C00B	C00A	110.89(17)	C9	C8	C7	112.6(2)
C009	C00B	C00K	112.20(18)	C00T	C00P	C00C	117.5(2)
C00K	C00B	C00A	111.53(17)	C00X	C00P	C00C	121.1(3)
C008	C00C	C00N	113.03(18)	C00X	C00P	C00T	121.3(3)
C00P	C00C	C008	110.43(17)	O2	C9	C1	106.3(2)
C00P	C00C	C00N	112.00(18)	O2	C9	C8	110.1(2)
O1	C10	C1	122.6(2)	C8	C9	C1	112.93(18)
O1	C10	C6	121.2(2)	C13	C11	C4	117.3(2)
C6	C10	C1	116.26(18)	C12	C11	C4	119.8(3)
C5	C4	C3	113.31(18)	C12	C11	C13	122.8(3)
C11	C4	C5	112.10(19)	C2	C3	C4	115.88(19)

Table S4-6 Torsion Angles for **12**.

**A**	**B**	**C**	**D**	**Angle/°**	**A**	**B**	**C**	**D**	**Angle/°**
O001	C006	C007	C005	-65.83(18)	C00C	C008	C00A	C00B	-152.26(17)
O001	C006	C007	C00E	54.7(2)	C10	C1	C2	C3	35.8(3)
O001	C006	C007	C00H	175.53(15)	C10	C1	C9	O2	-66.7(2)
O001	C006	C009	C00B	67.0(2)	C10	C1	C9	C8	54.2(3)

O002	C005	C007	C006	119.24(19)	C10	C6	C7	C8	-52.1(2)
O002	C005	C007	C00E	-1.4(3)	C10	C6	C7	C15	-177.1(2)
O002	C005	C007	C00H	-122.3(2)	C00E	C007	C00H	C00N	-88.1(2)
O002	C005	C00A	C008	116.7(2)	C4	C5	C6	C10	-26.0(3)
O002	C005	C00A	C00B	-116.28(19)	C4	C5	C6	C7	-150.37(19)
O1	C10	C1	C2	-118.3(2)	C5	C4	C11	C13	-47.7(3)
O1	C10	C1	C9	122.9(2)	C5	C4	C11	C12	134.8(3)
O1	C10	C1	C14	2.5(3)	C5	C4	C3	C2	-11.2(3)
O1	C10	C6	C5	109.7(2)	C5	C6	C7	C8	73.6(2)
O1	C10	C6	C7	-122.8(2)	C5	C6	C7	C15	-51.5(3)
C005	C007	C00H	C00N	33.9(2)	C1	C10	C6	C5	-69.3(2)
C005	C00A	C00B	C009	-55.5(2)	C1	C10	C6	C7	58.1(2)
C005	C00A	C00B	C00K	178.69(18)	C1	C2	C3	C4	-67.3(3)
C006	C007	C00H	C00N	149.92(17)	C6	C10	C1	C2	60.8(2)
C006	C009	C00B	C00A	54.5(2)	C6	C10	C1	C9	-58.0(2)
C006	C009	C00B	C00K	179.90(17)	C6	C10	C1	C14	-178.4(2)
C007	C005	C00A	C008	-66.0(2)	C6	C7	C8	C9	52.4(3)
C007	C005	C00A	C00B	61.0(2)	C7	C8	C9	O2	63.9(2)
C007	C006	C009	C00B	-53.7(2)	C7	C8	C9	C1	-54.7(3)
C007	C00H	C00N	C00C	-67.4(3)	C2	C1	C9	O2	175.00(19)
C008	C00A	C00B	C009	70.0(2)	C2	C1	C9	C8	-64.2(3)
C008	C00A	C00B	C00K	-55.8(2)	C00N	C00C	C00P	C00T	63.1(3)
C008	C00C	C00N	C00H	-9.5(3)	C00N	C00C	C00P	C00X	-119.1(3)
C008	C00C	C00P	C00T	-63.8(3)	C00P	C00C	C00N	C00H	-135.0(2)
C008	C00C	C00P	C00X	114.0(3)	C9	C1	C2	C3	152.3(2)
C009	C006	C007	C005	51.9(2)	C11	C4	C5	C6	-160.8(2)
C009	C006	C007	C00E	172.41(17)	C11	C4	C3	C2	-136.8(2)
C009	C006	C007	C00H	-66.8(2)	C3	C4	C5	C6	75.4(2)
C00A	C005	C007	C006	-58.0(2)	C3	C4	C11	C13	78.6(3)

C00A	C005	C007	C00E	-178.56(16)	C3	C4	C11	C12	-99.0(3)
C00A	C005	C007	C00H	60.5(2)	C15	C7	C8	C9	177.7(2)
C00A	C008	C00C	C00N	76.1(2)	C14	C1	C2	C3	-85.4(3)
C00A	C008	C00C	C00P	-157.60(19)	C14	C1	C9	O2	53.4(3)
C00C	C008	C00A	C005	-29.3(2)	C14	C1	C9	C8	174.3(2)

Table S4-7 Hydrogen Atom Coordinates (Å×10^4) and Isotropic Displacement Parameters (Å^2×10^3) for **12**.

**Atom**	***x***	***y***	***z***	**U(eq)**
H001	8226.64	6810.17	5213.9	49
H2	8616.58	5516.07	5625.39	86
H006	10021.39	5964.34	4792.87	34
H00A	6552.6	4292.73	4066.19	37
H00B	4104.64	3994.27	3994.84	37
H00C	8640.57	4365.78	4578.99	38
H00D	8567.74	4369.04	5033.65	38
H00E	2912.5	4952.22	4481.77	34
H00F	4915.09	4762.72	5027.89	37
H00G	3635.9	5826.57	3790.04	41
H00H	7051.09	8004.97	4299.39	52
H00I	9200.17	7804.36	4534.03	52
H00J	6961.82	7875.61	4751.52	52
H4	8491.17	3183.04	7162.27	46
H5A	4756.88	3423.04	6704.37	40
H5B	5700.63	2287.99	6791.01	40
H00K	9630.82	6558.63	4034.86	41
H00L	8871.85	5389.44	4113.56	41
H6	8524.5	2500.4	6416.72	42
H00M	5726.86	2976.28	4564.43	62

H00N	3524.32	3189.63	4781.17	62
H00O	5657.08	2977.15	5019.12	62
H7	7769.19	3250.33	5842.71	52
H2A	6414.38	5986.25	6868.75	55
H2B	5206.16	4991.02	6701.31	55
H00P	7723.51	6053.84	3511.76	49
H00Q	6297.2	6894.93	3728.63	49
H8A	5171.98	4519.39	5738.72	56
H8B	4441.16	4479.95	6174.23	56
H9	6575.68	5965.21	6086.47	55
H3A	5946.31	4860.13	7365.03	60
H3B	8437.31	4859.36	7270.62	60
H00R	6033.62	3903.3	2969.57	90
H00S	6696.87	3647.14	3398.79	90
H00T	7734.84	4624.13	3184.31	90
H15A	5571.74	1793.45	5994.65	90
H15B	4460.57	2552.97	5692.75	90
H15C	3732.19	2577.48	6128.62	90
H14A	10659.5	5846.45	6816.69	91
H14B	9474.28	6639.69	6535.98	91
H14C	11204.14	5838.65	6372.31	91
H13A	2962.47	3744.55	7413.41	96
H13B	2846.95	2816.74	7717.03	96
H13C	2901.18	2558.31	7272.14	96
H00U	2719.16	4661.37	2922.49	87
H00V	1786.01	5446.95	3252.9	87
H12A	6111.41	2375.13	7996.92	96
H12B	8374.61	2613.31	7776.18	96

S5. Spectroscopic data

Qualitative Analysis Report

**Data Filename**	ESIH202304976.d	**Sample Name**	E0-DY-163
**Sample ID**		**Position**	P1-B1
**Instrument Name**	Agilent 6520 Q-TOF	**Acq Method**	20160322_MS_ESIH_POS_1min.m
**Acquired Time**	8/23/2023 14:28:36	**IRM Calibration Status**	Success
**DA Method**	small molecular data analysis method.m	**Comment**	ESIH by fangsu

User Spectra

Fragmentor Voltage	Collision Energy	Ionization Mode
170	0	ESI

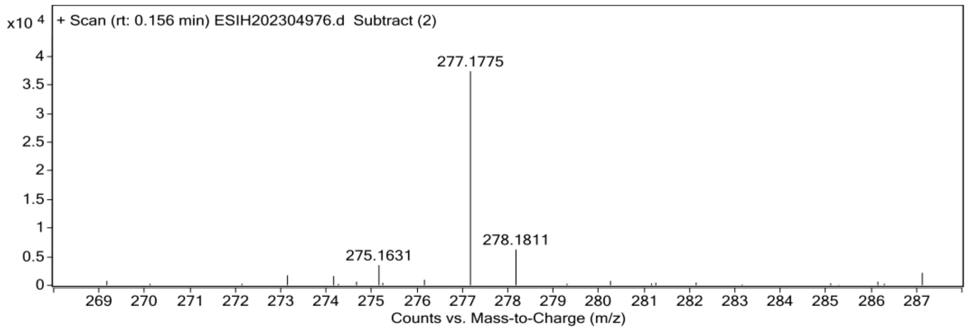

Formula Calculator Results

m/z	Calc m/z	Diff (mDa)	Diff (ppm)	Ion Formula	Ion
277.1775	277.1774	-0.11	-0.41	C15 H26 Na O3	(M+Na)+

--- End Of Report ---

Figure S5-1. The HRESIMS spectrum of compound **1**

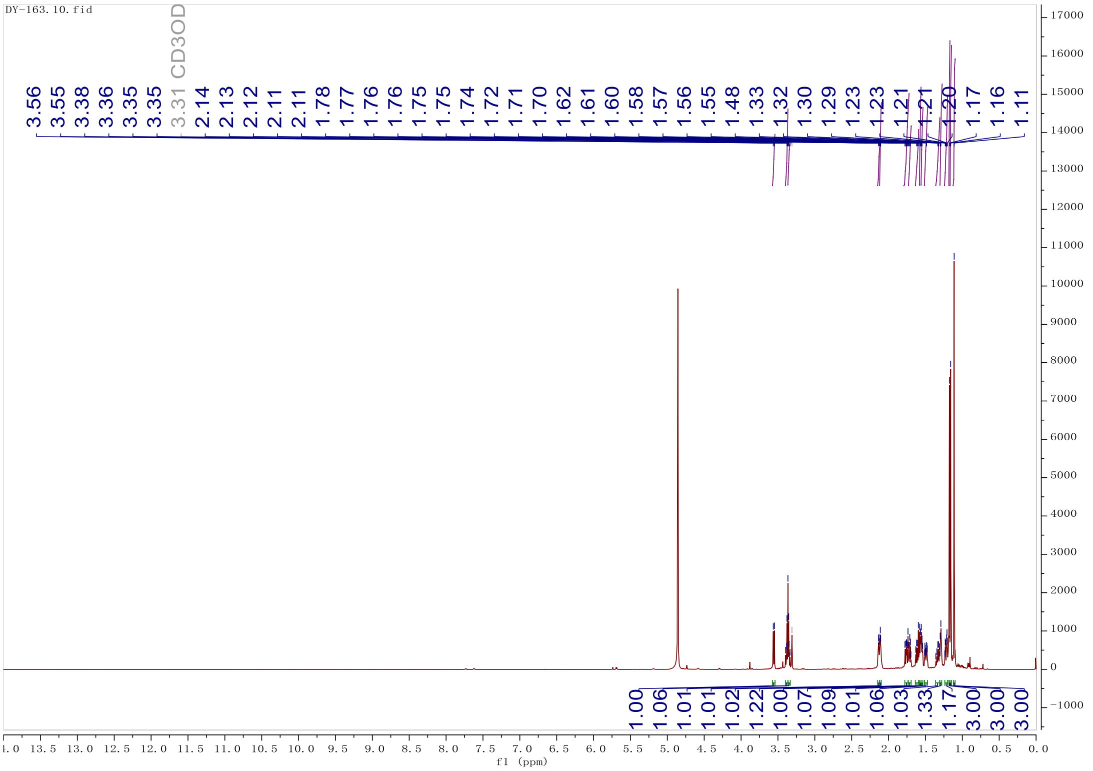

Figure S5-2. The ^{1}H NMR spectrum of compound **1** in CD_3OD (600 MHz)

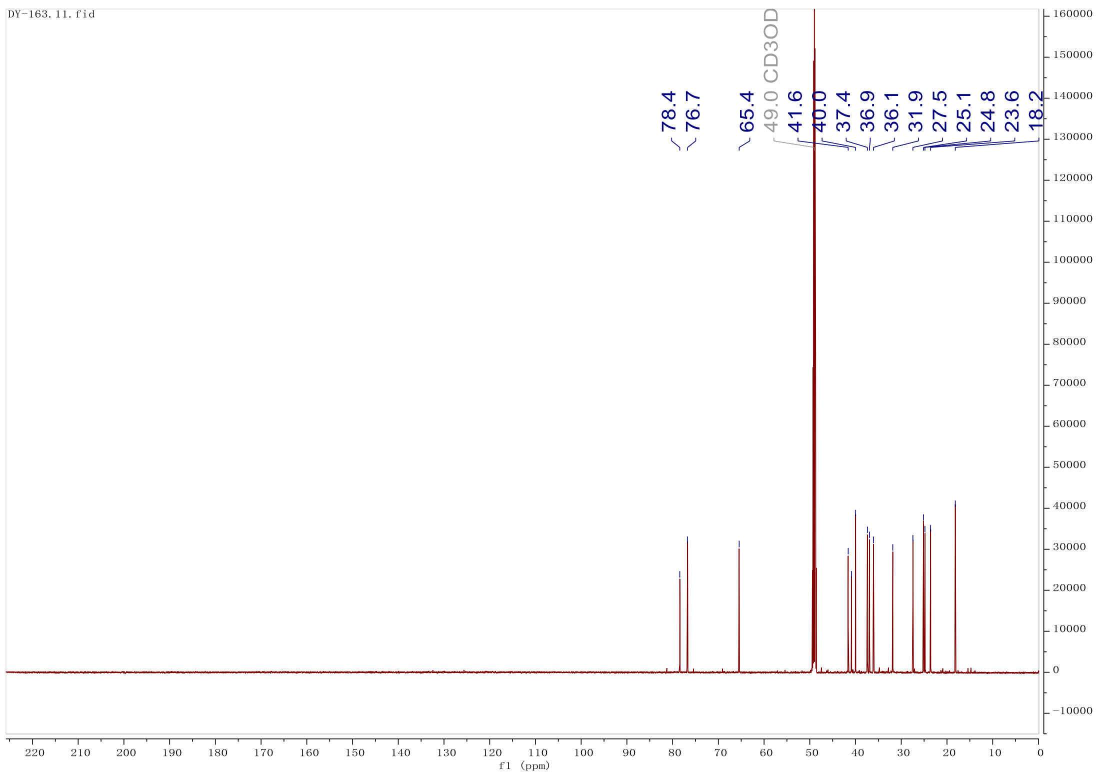

Figure S5-3. The ^{13}C NMR spectrum of compound **1** in CD_3OD (150 MHz)

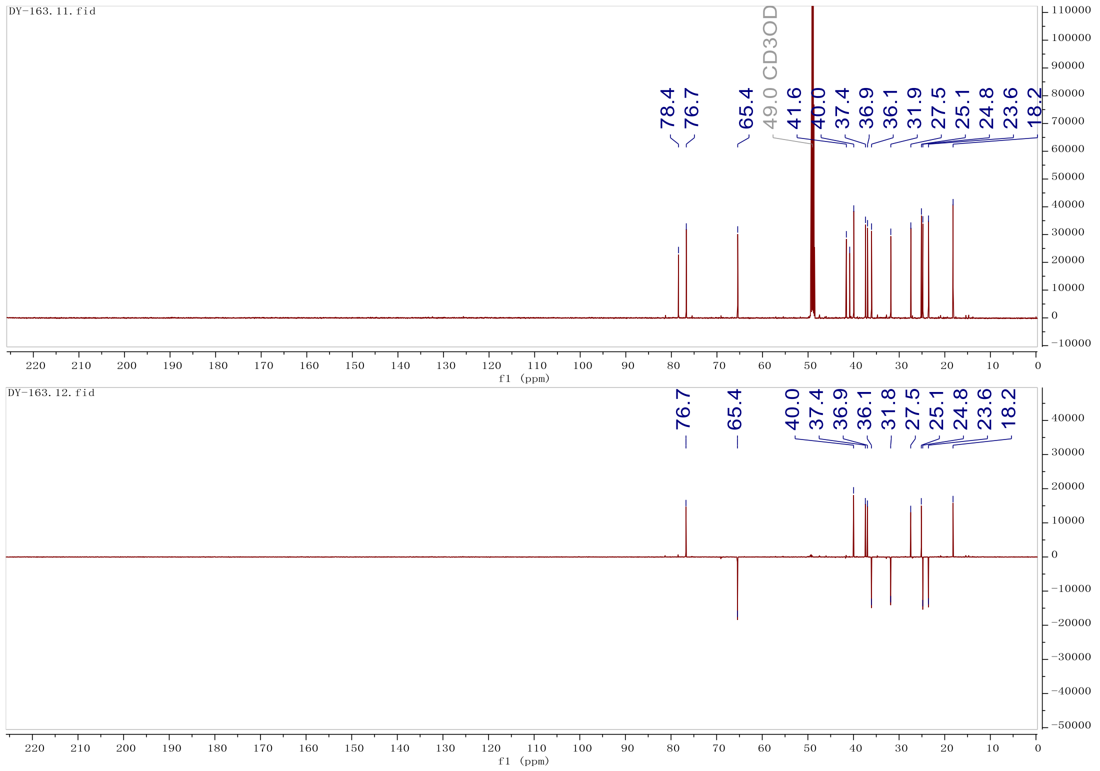

Figure S5-4. The DEPT 135 spectrum of compound **1** in CD_3OD (150 MHz)

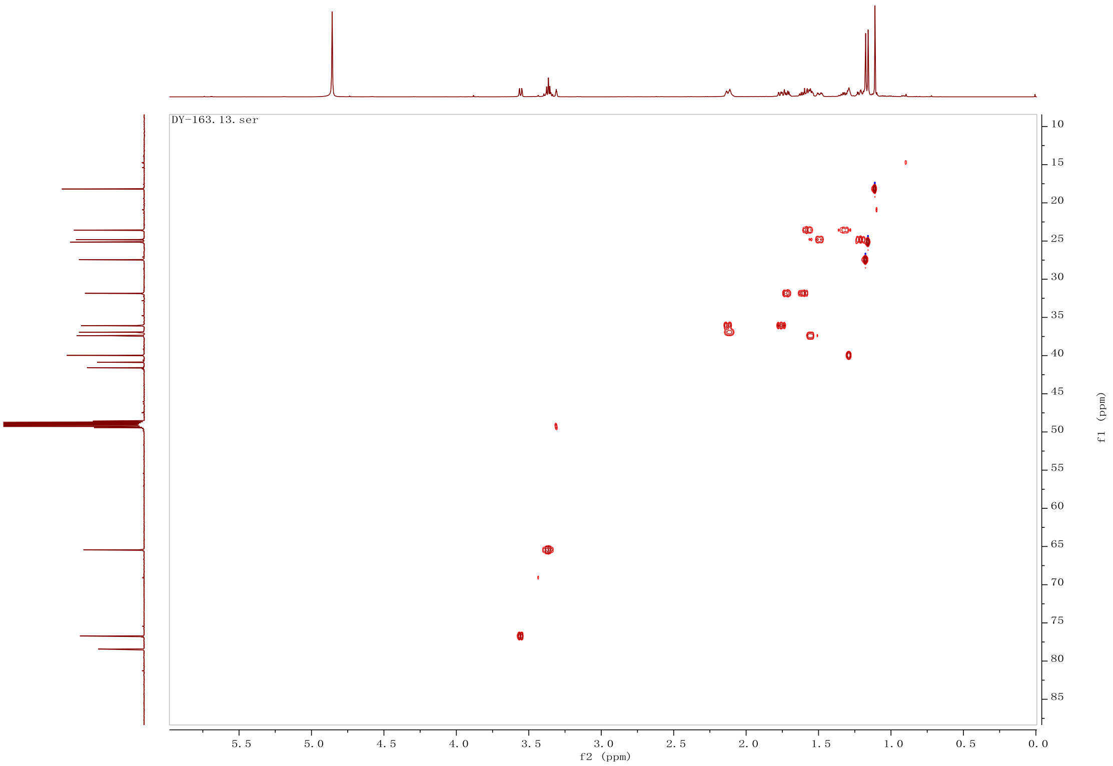

Figure S5-5. The HSQC spectrum of compound **1** in CD_3OD (600 MHz)

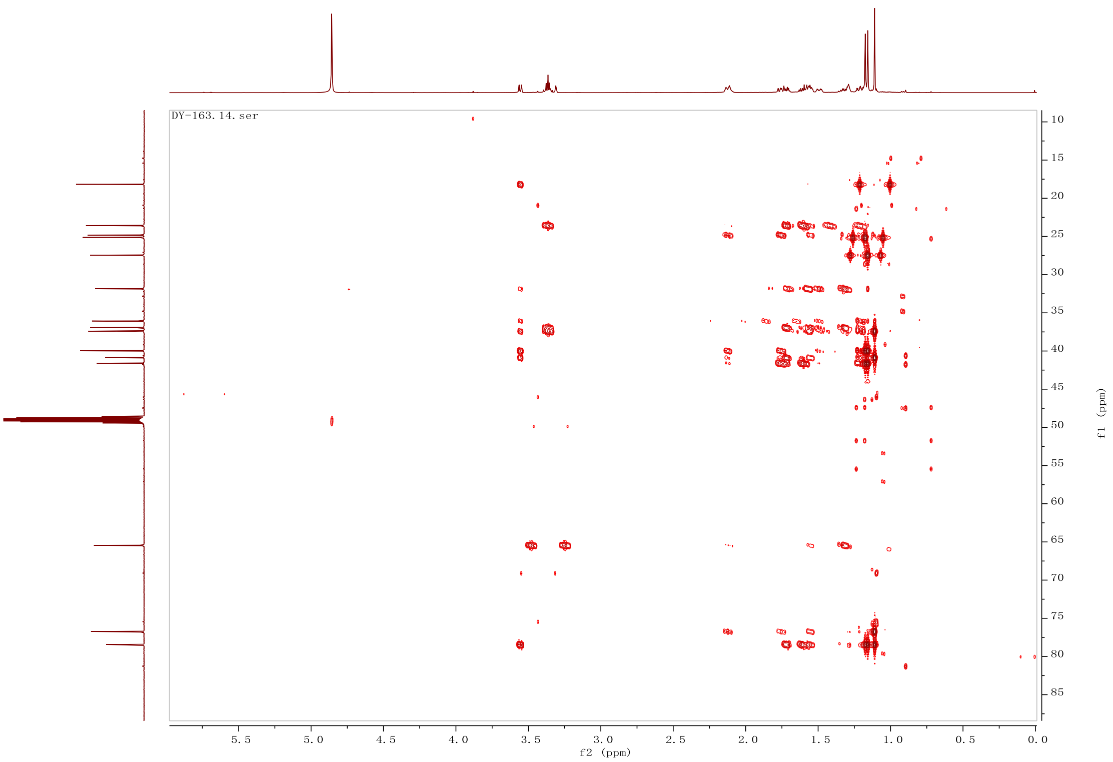

Figure S5-6. The HMBC spectrum of compound **1** in CD_3OD (600 MHz)

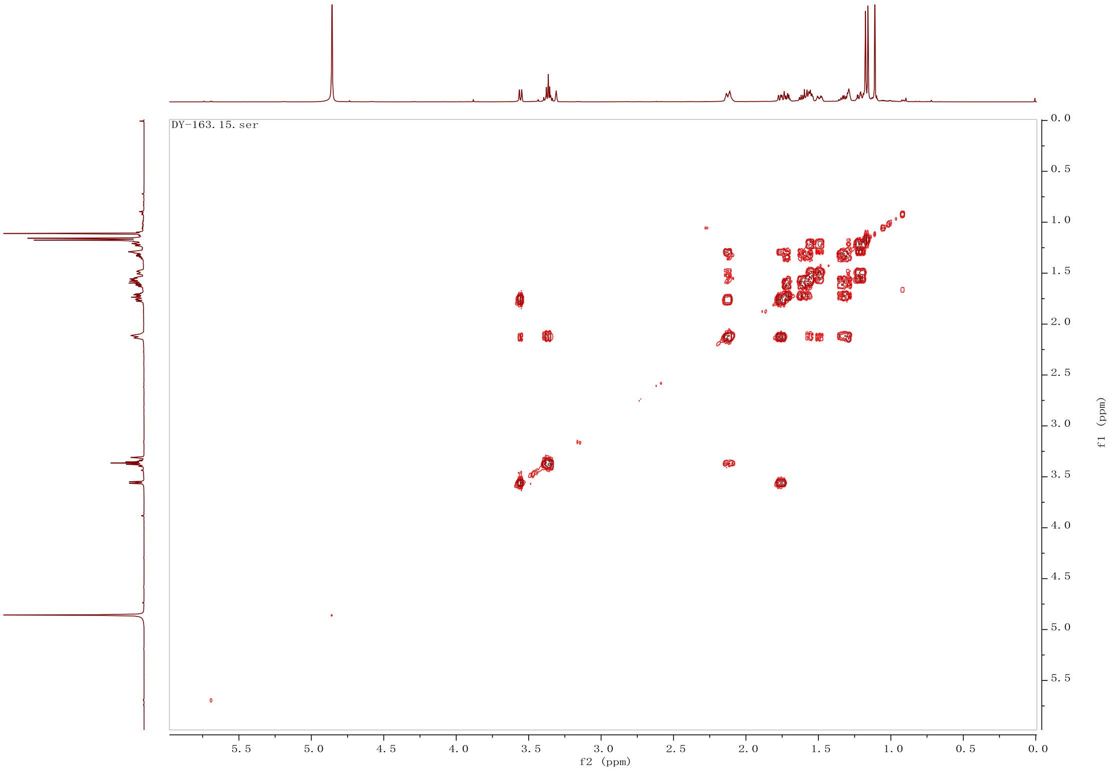

Figure S5-7. The ^{1}H-^{1}H COSY spectrum of compound **1** in CD_3OD (600 MHz)

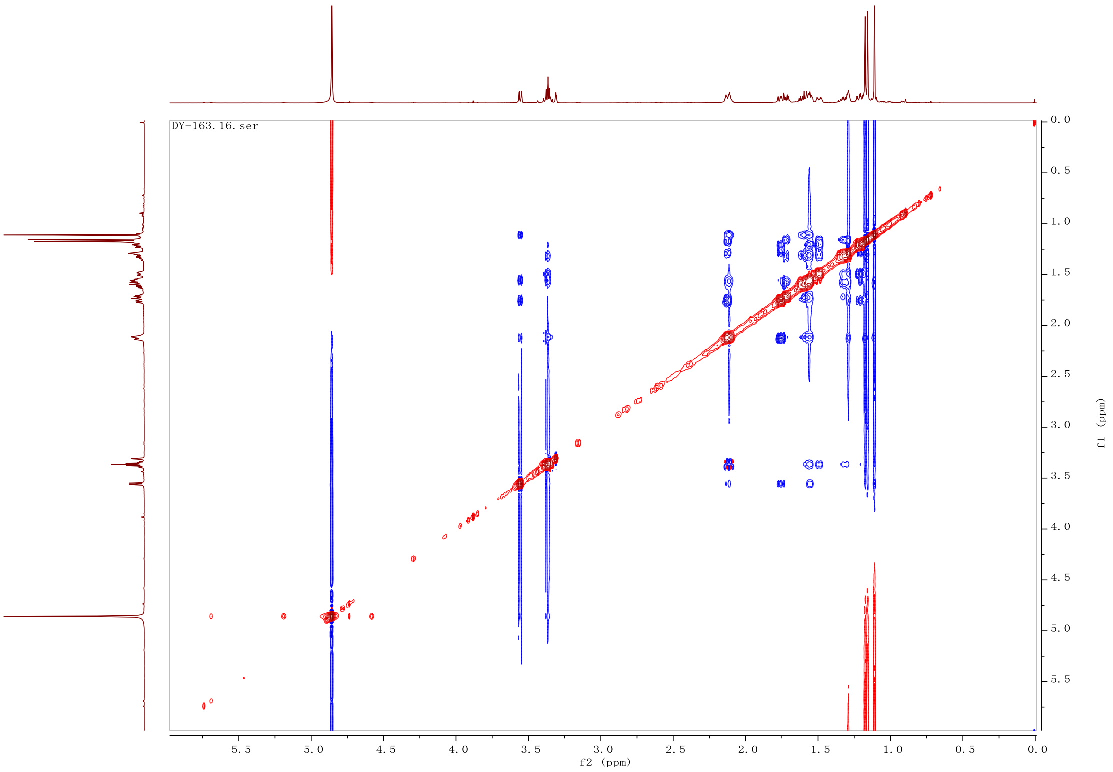

Figure S5-8. The NOESY spectrum of compound **1** in CD_3OD (600 MHz)

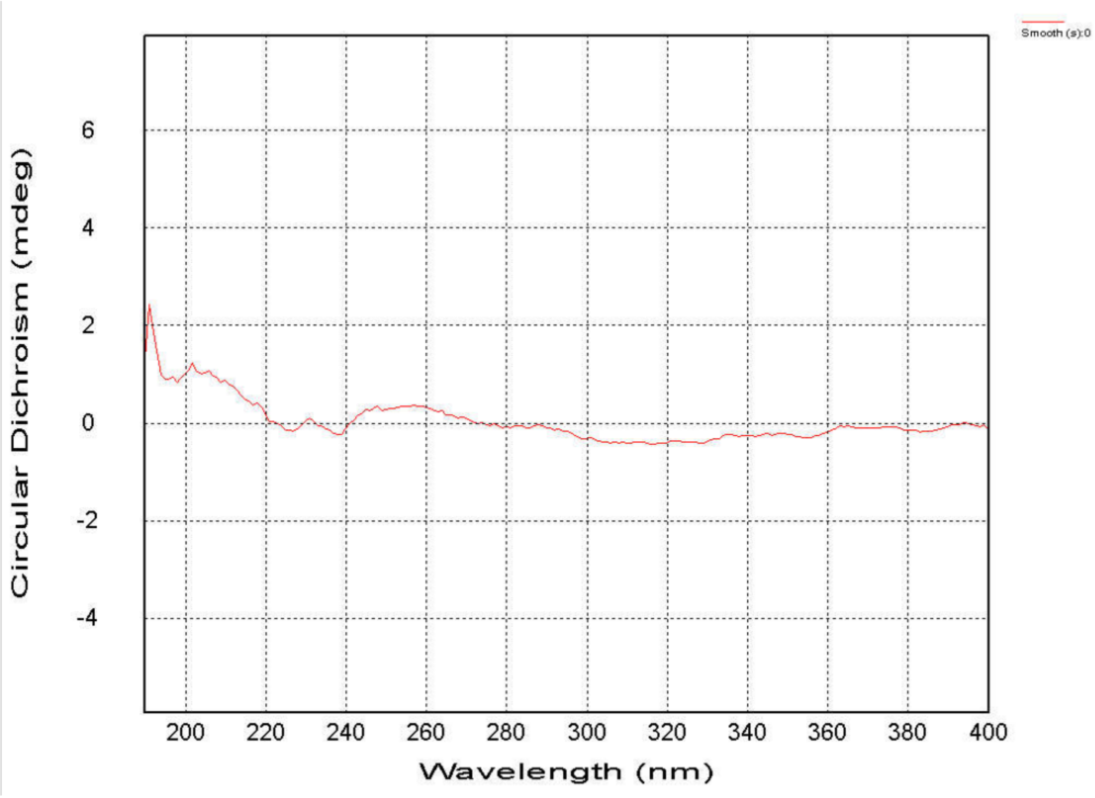

Figure S5-9. The ECD spectrum of compound **1** in MeOH

Qualitative Analysis Report

**Data Filename**	ESIH202306277-1.d	**Sample Name**	E0-DY-188
**Sample ID**		**Position**	P1-A4
**Instrument Name**	Agilent 6520 Q-TOF	**Acq Method**	20160322_MS_ESIH_POS_1min.m
**Acquired Time**	11/3/2023 10:11:22	**IRM Calibration Status**	Success
**DA Method**	small molecular data analysis method.m	**Comment**	ESIH by fangsu

User Spectra

Fragmentor Voltage	Collision Energy	Ionization Mode
170	0	ESI

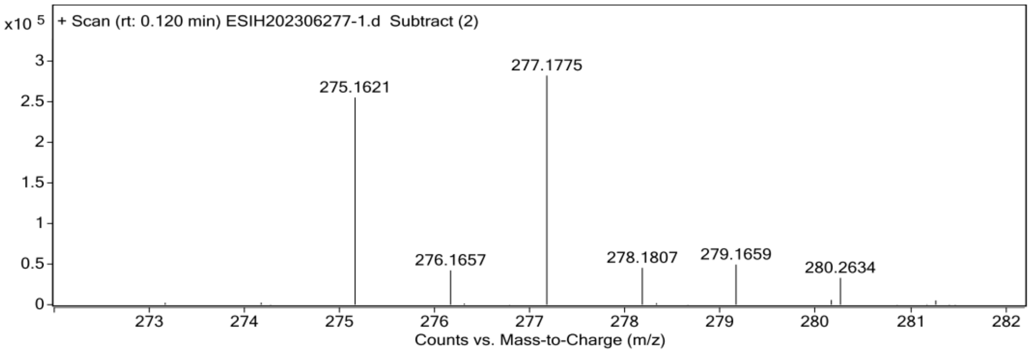

Formula Calculator Results

m/z	Calc m/z	Diff (mDa)	Diff (ppm)	Ion Formula	Ion
277.1775	277.1774	-0.1	-0.37	C15 H26 Na O3	(M+Na)+

--- End Of Report ---

Figure S5-10. The HRESIMS spectrum of compound **2**

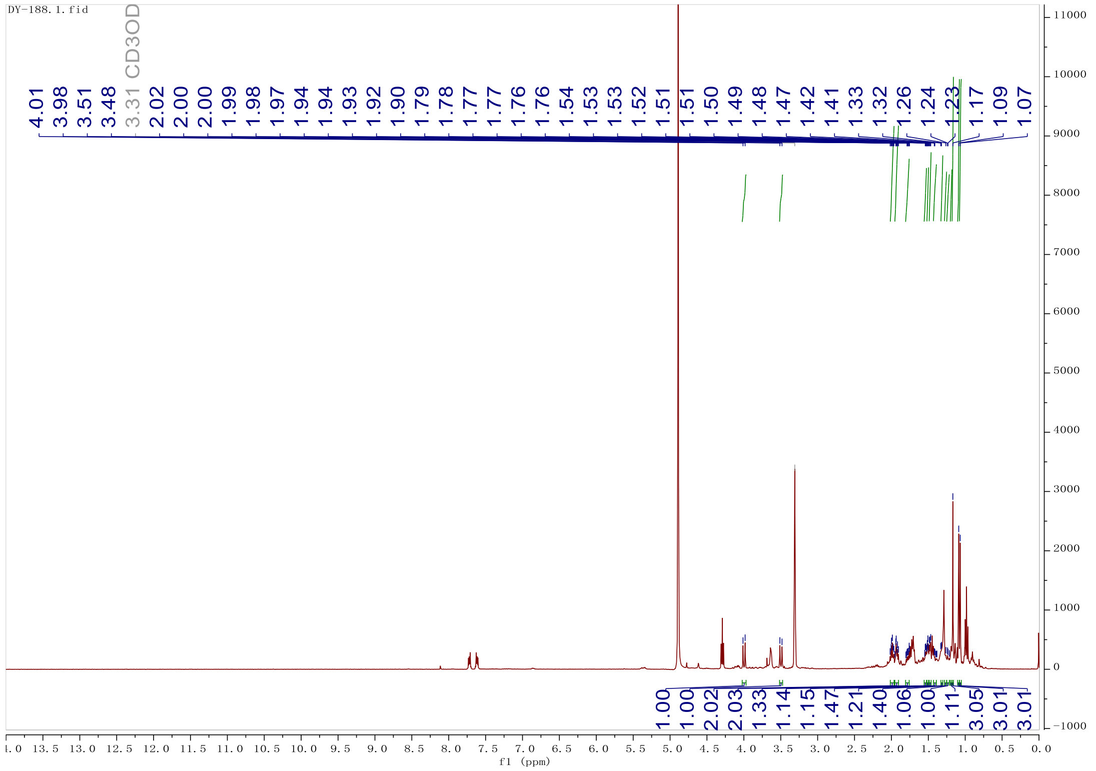

Figure S5-11. The ^{1}H NMR spectrum of compound **2** in CD_3OD (600 MHz)

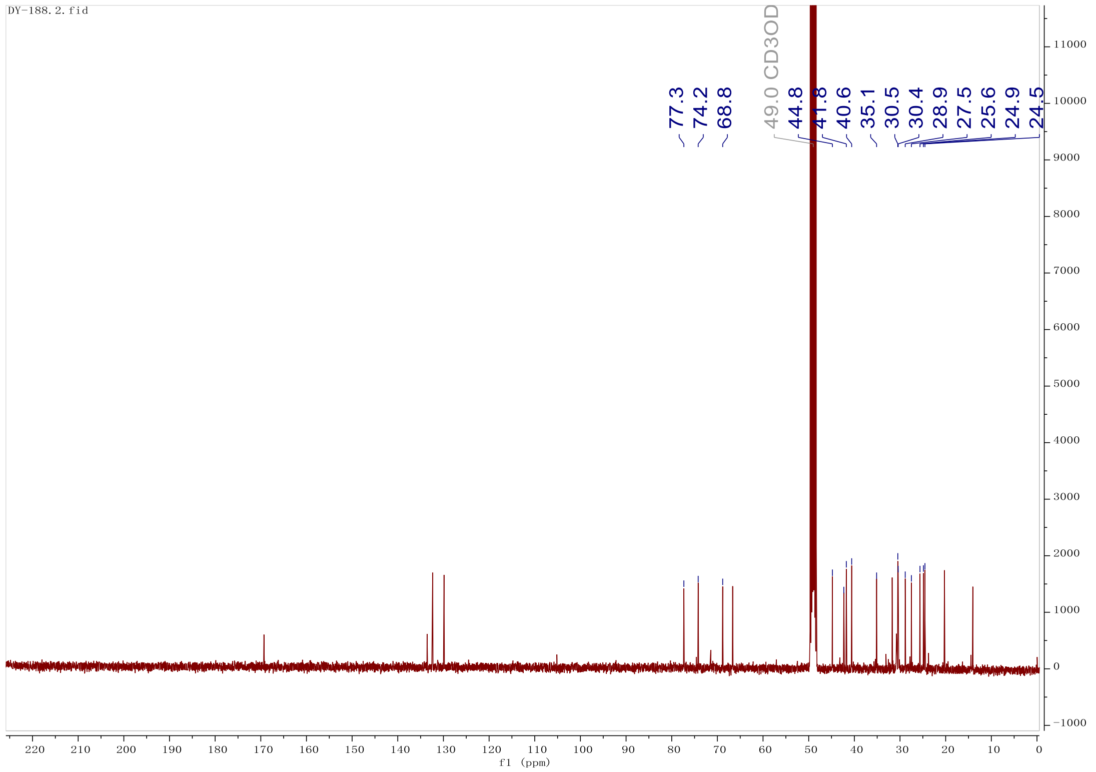

Figure S5-12. The ^{13}C NMR spectrum of compound **2** in CD_3OD (150 MHz)

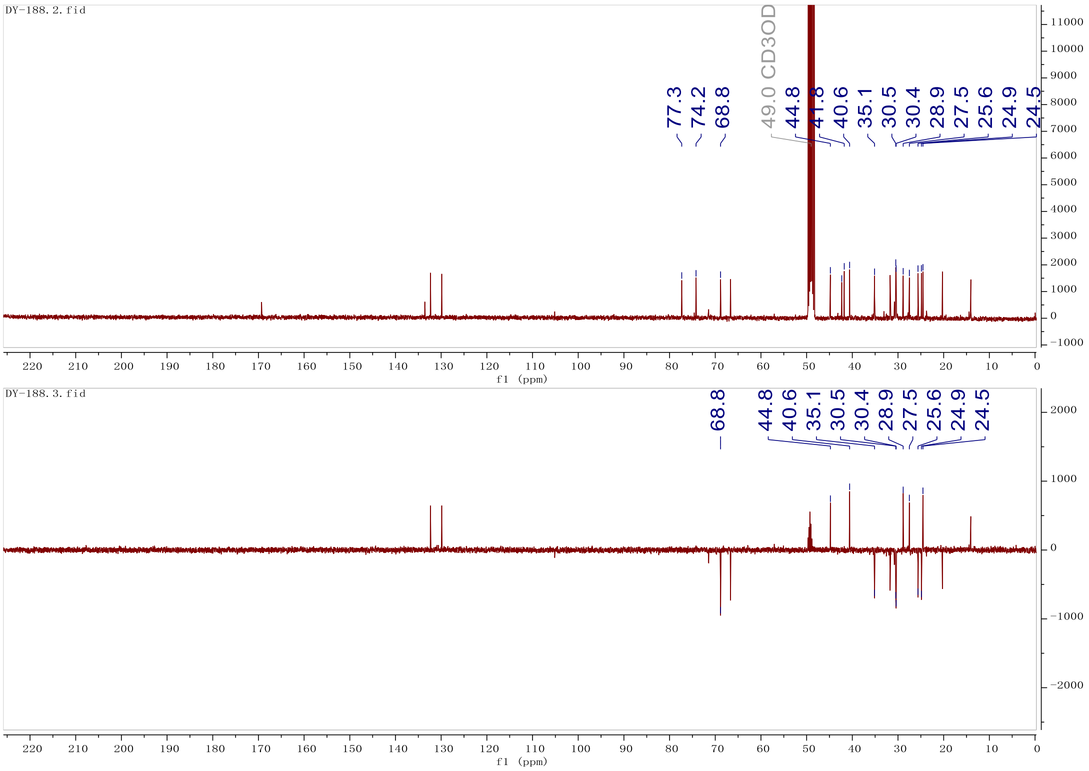

Figure S5-13. The DEPT 135 spectrum of compound **2** in CD_3OD (150 MHz)

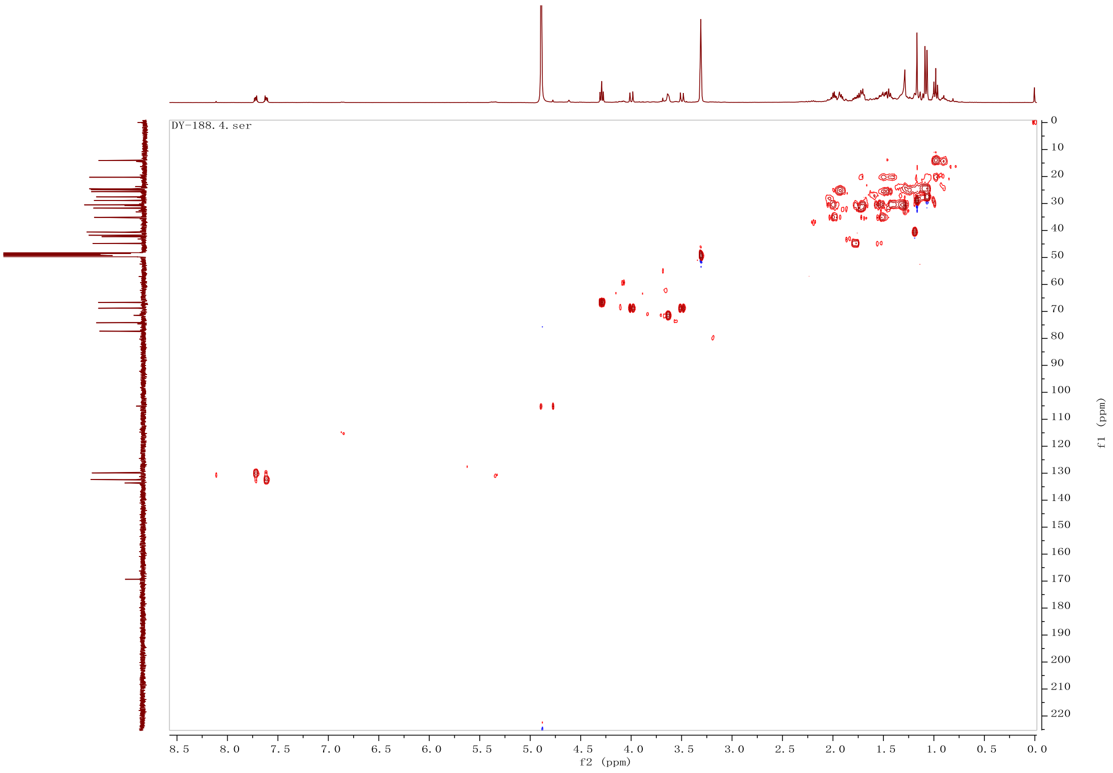

Figure S5-14. The HSQC spectrum of compound **2** in CD_3OD (600 MHz)

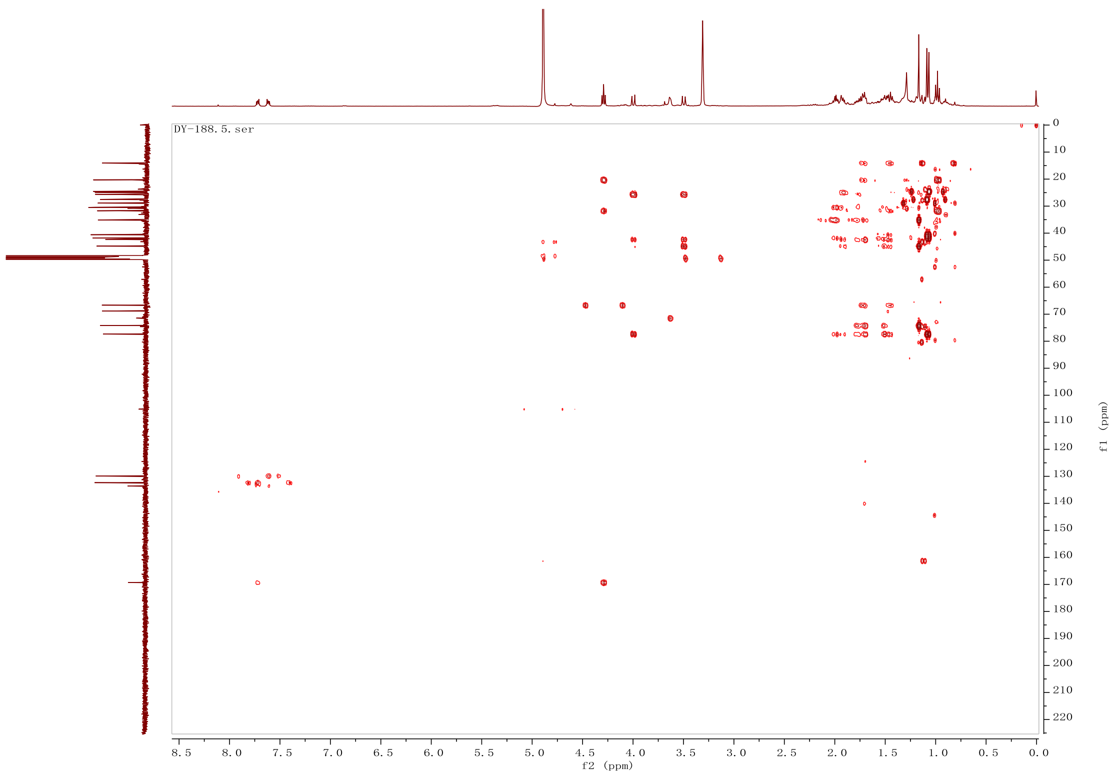

Figure S5-15. The HMBC spectrum of compound **2** in CD_3OD (600 MHz)

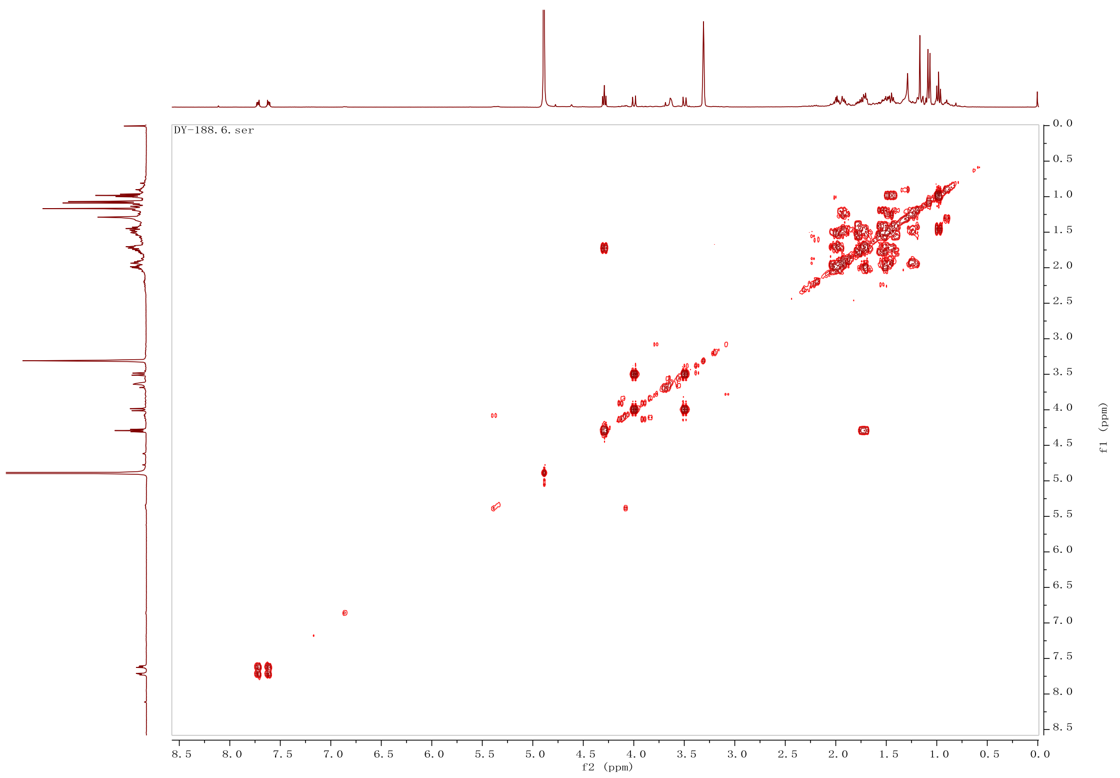

Figure S5-16. The ^1H-^1H COSY spectrum of compound **2** in CD_3OD (600 MHz)

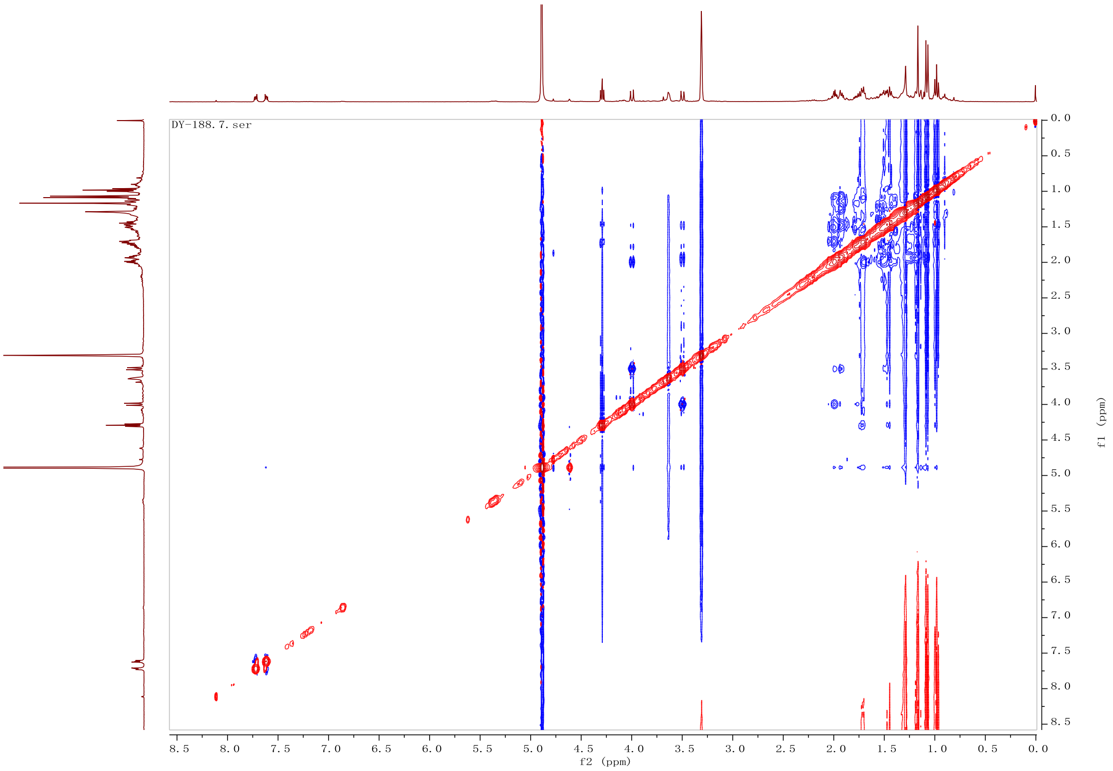

Figure S5-17. The NOESY spectrum of compound **2** in CD_3OD (600 MHz)

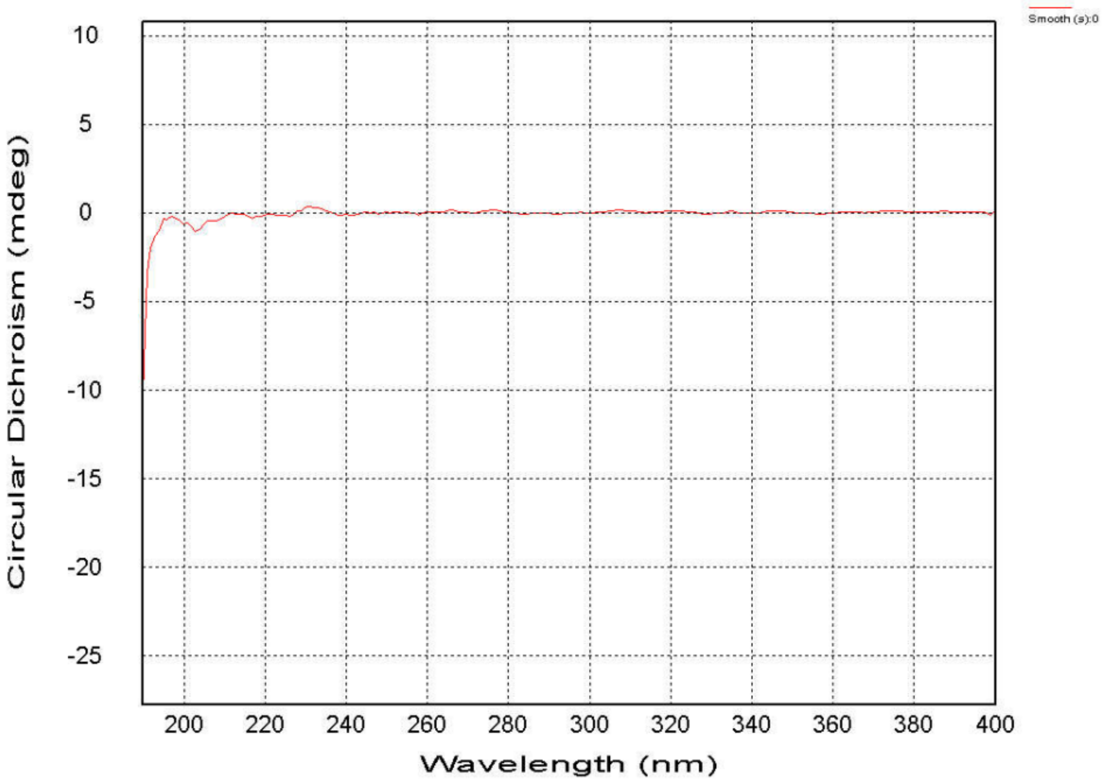

Figure S5-18. The ECD spectrum of compound **2** in MeOH

Qualitative Analysis Report

**Data Filename**	ESIH202304970.d	**Sample Name**	E0-DY-148
**Sample ID**		**Position**	P1-A4
**Instrument Name**	Agilent 6520 Q-TOF	**Acq Method**	20160322_MS_ESIH_POS_1min.m
**Acquired Time**	8/23/2023 14:20:53	**IRM Calibration Status**	Success
**DA Method**	small molecular data analysis method.m	**Comment**	ESIH by fangsu

User Spectra

Fragmentor Voltage	Collision Energy	Ionization Mode
170	0	ESI

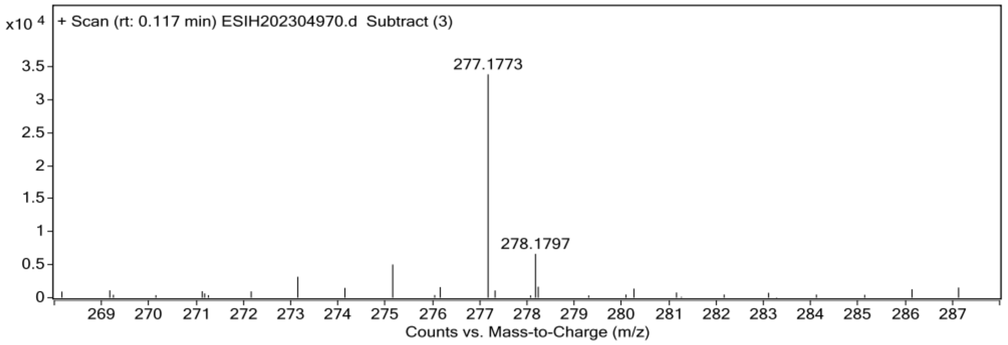

Formula Calculator Results

m/z	Calc m/z	Diff (mDa)	Diff (ppm)	Ion Formula	Ion
277.1773	277.1774	0.09	0.33	C15 H26 Na O3	(M+Na)+

--- End Of Report ---

Figure S5-19. The HRESIMS spectrum of compound **3**

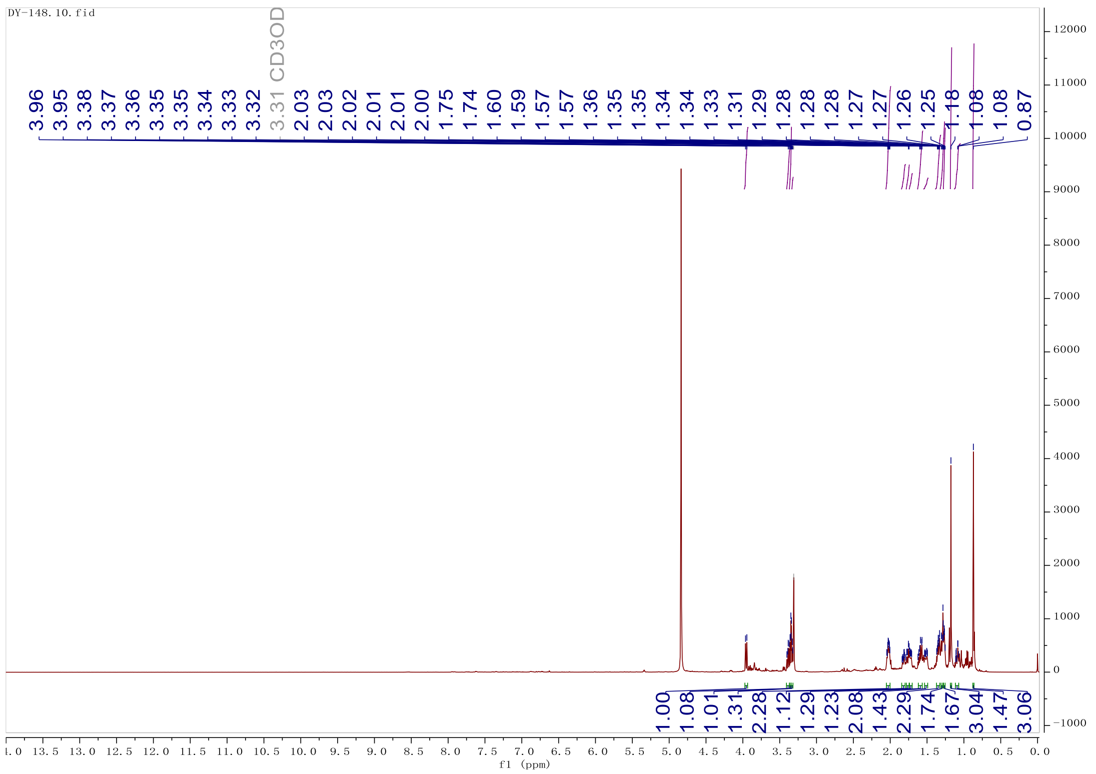

Figure S5-20. The ^{1}H NMR spectrum of compound **3** in CD_3OD (600 MHz)

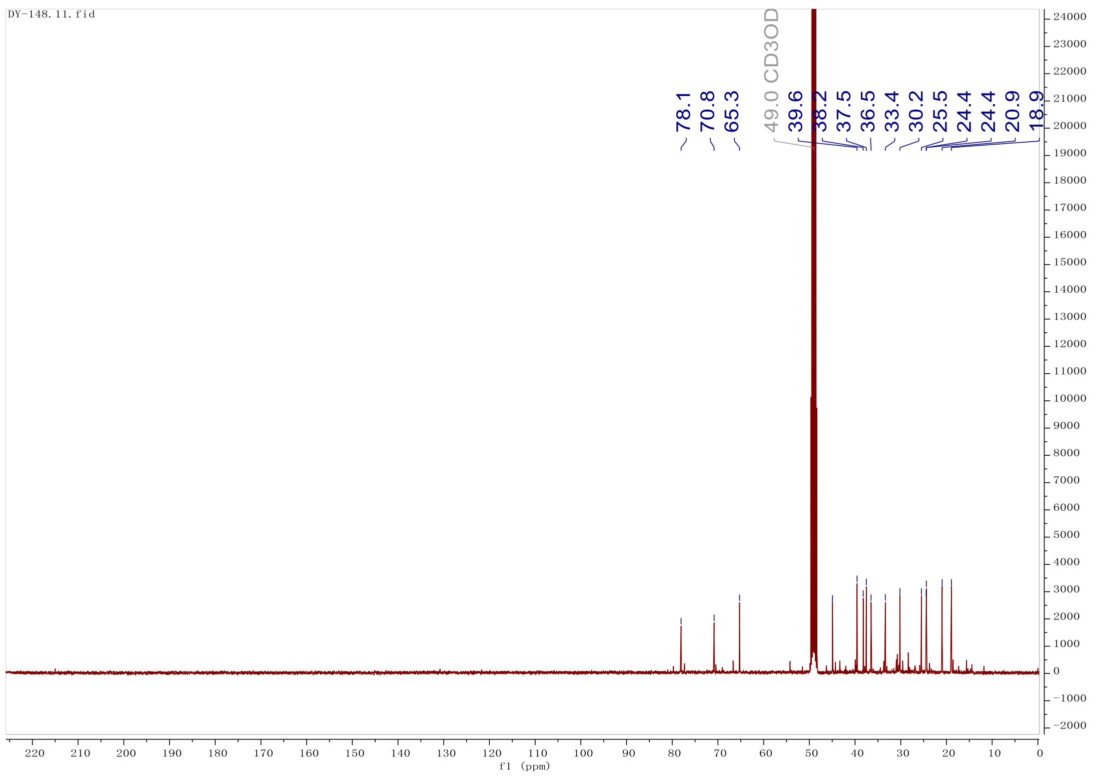

Figure S5-21. The ^{13}C NMR spectrum of compound **3** in CD_3OD (150 MHz)

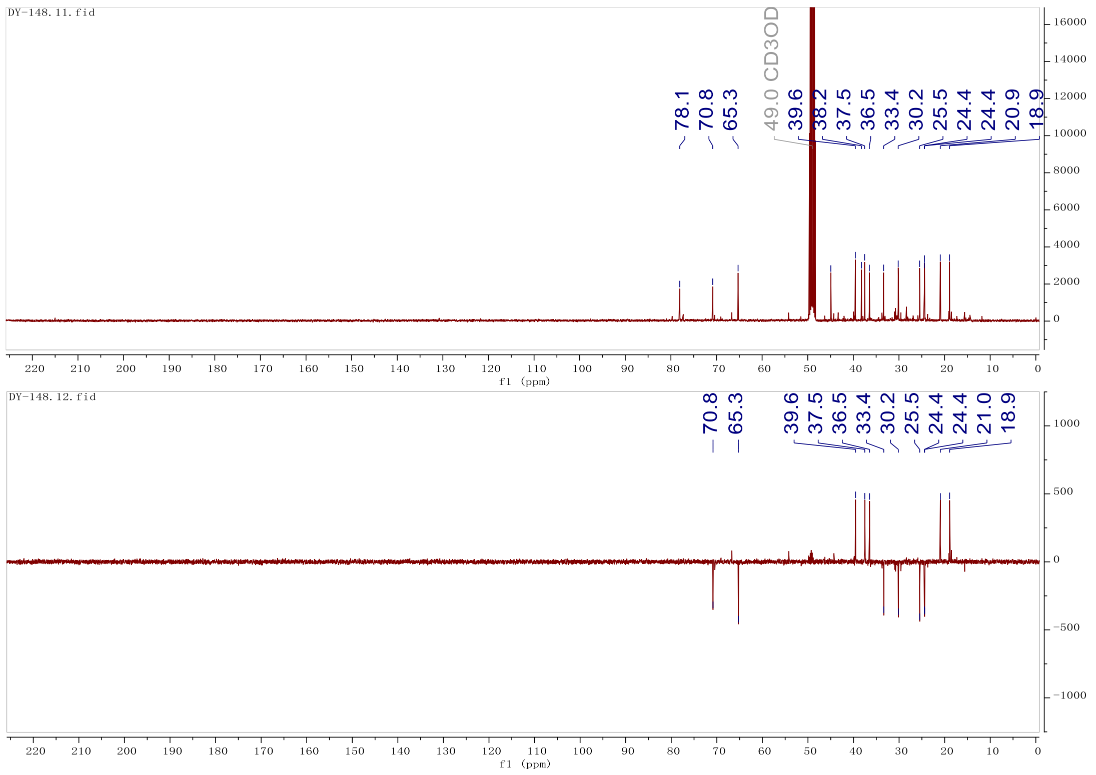

Figure S5-22. The DEPT 135 spectrum of compound **3** in CD_3OD (150 MHz)

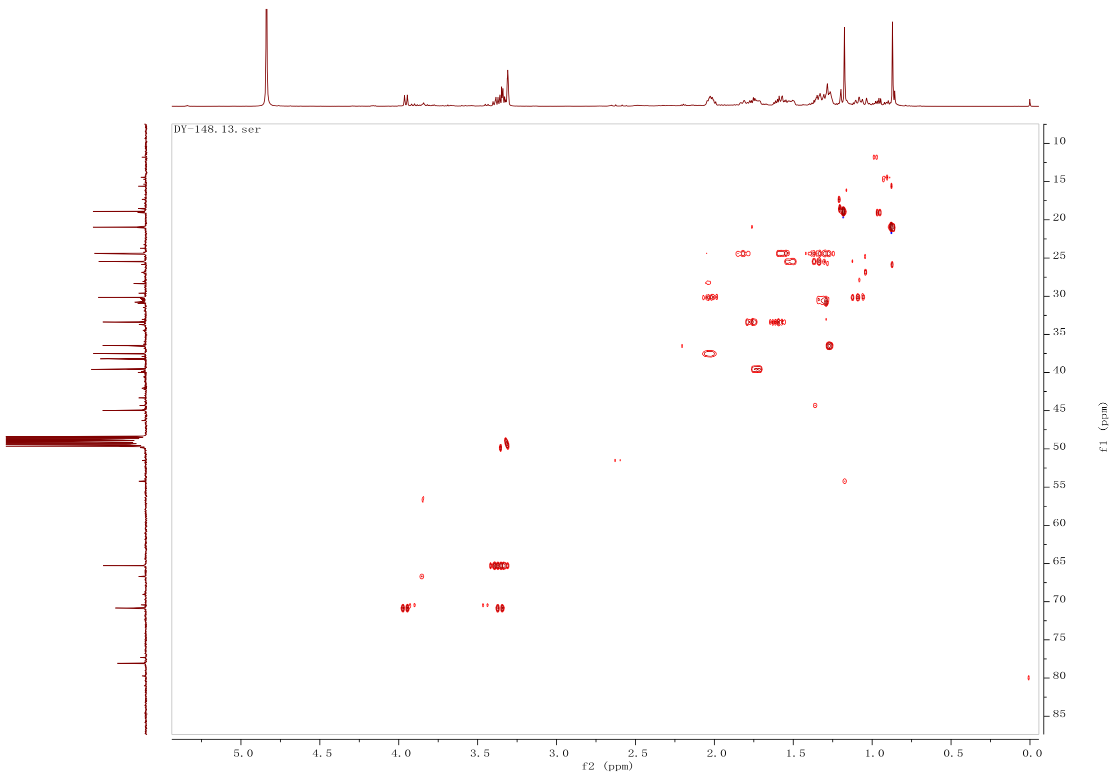

Figure S5-23. The HSQC spectrum of compound **3** in CD_3OD (600 MHz)

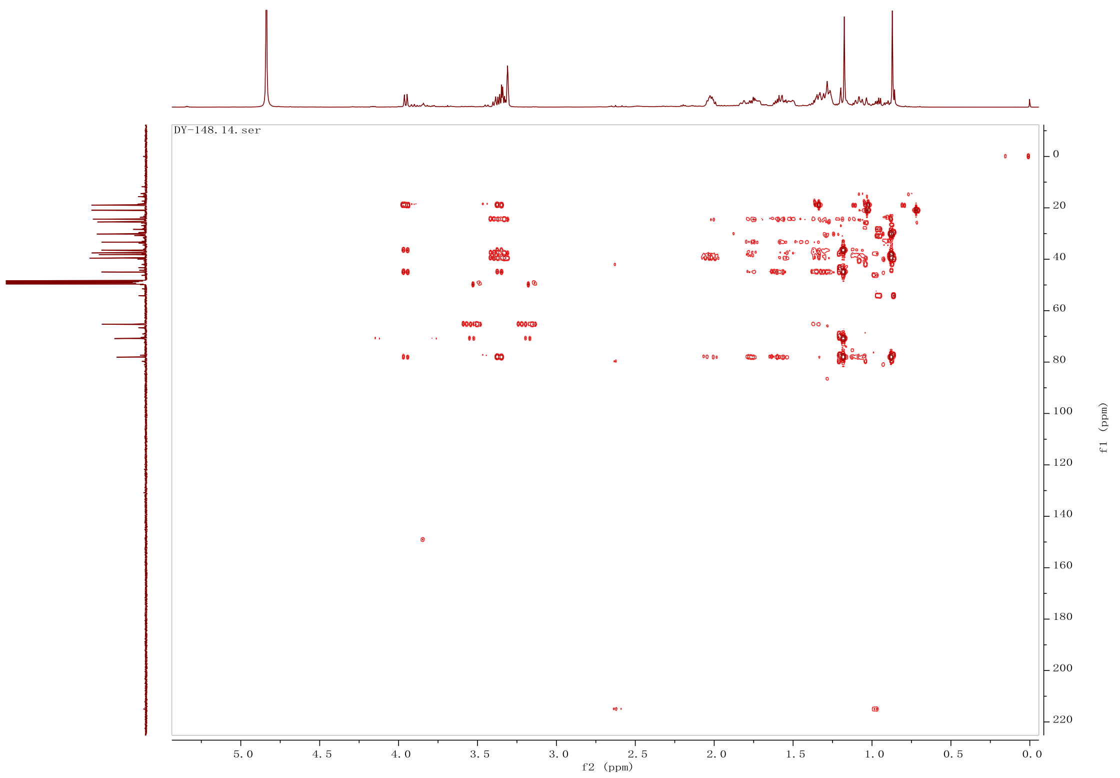

Figure S5-24. The HMBC spectrum of compound **3** in CD_3OD (600 MHz)

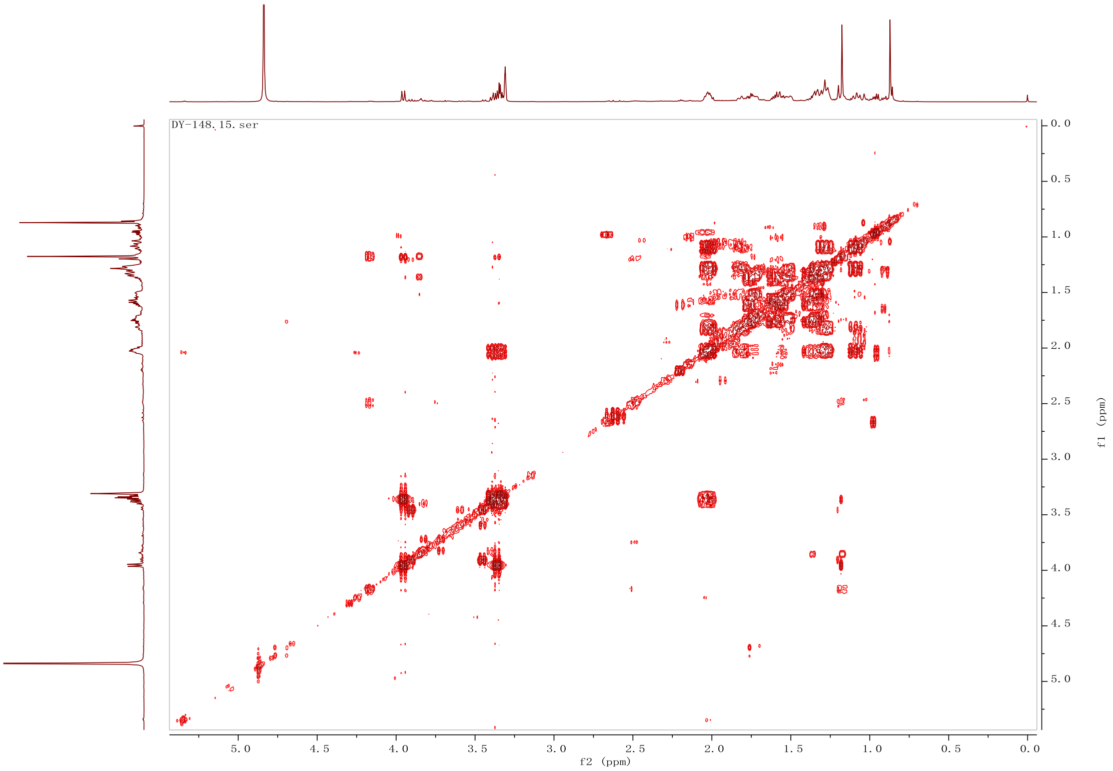

Figure S5-25. The ^1H-^1H COSY spectrum of compound **3** in CD_3OD (600 MHz)

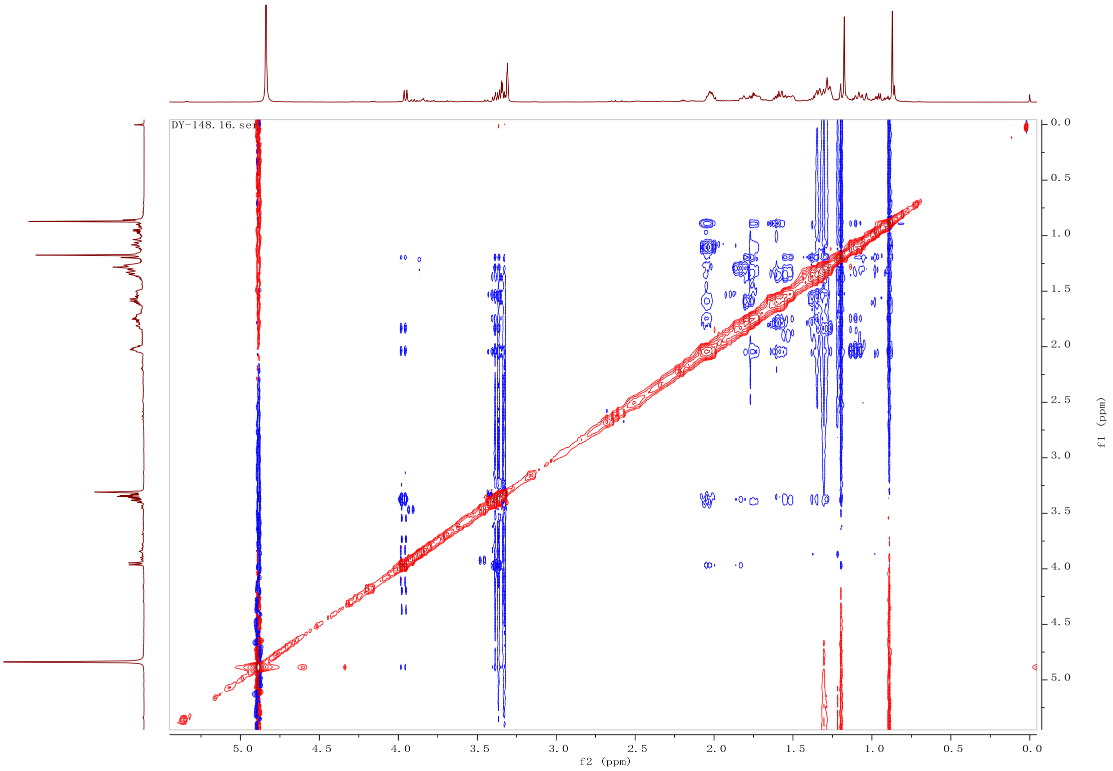

Figure S5-26. The NOESY spectrum of compound **3** in CD_3OD (600 MHz)

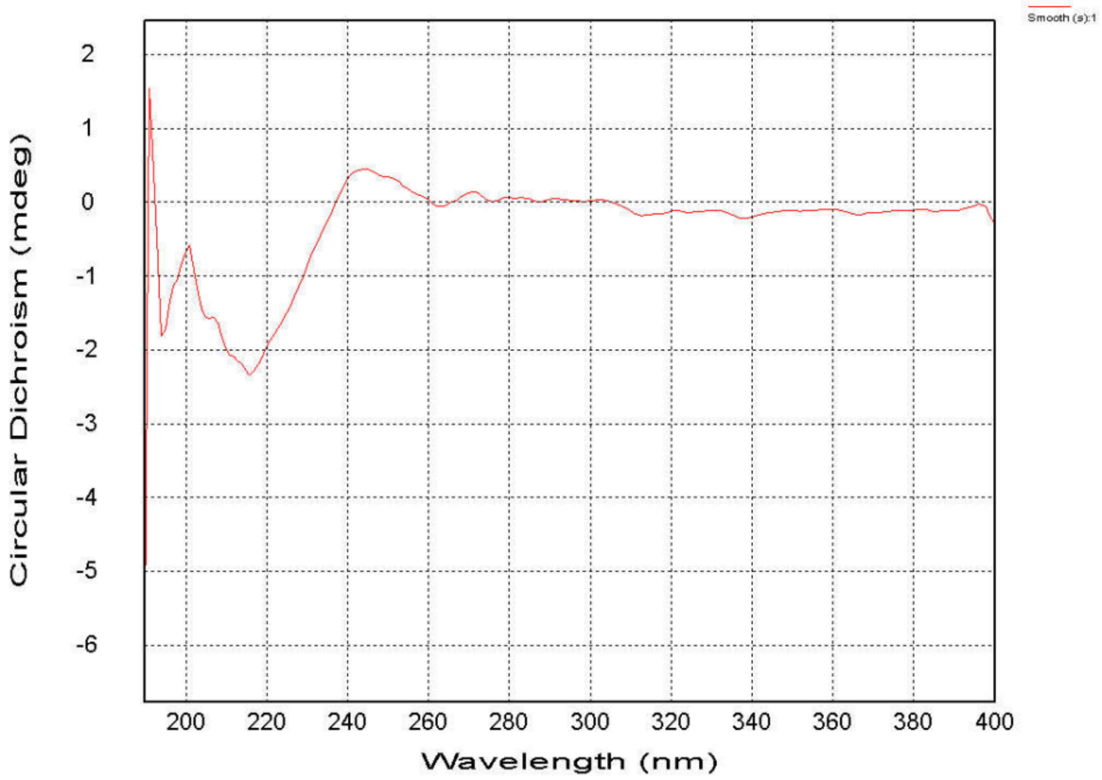

Figure S5-27. The ECD spectrum of compound **3** in MeOH

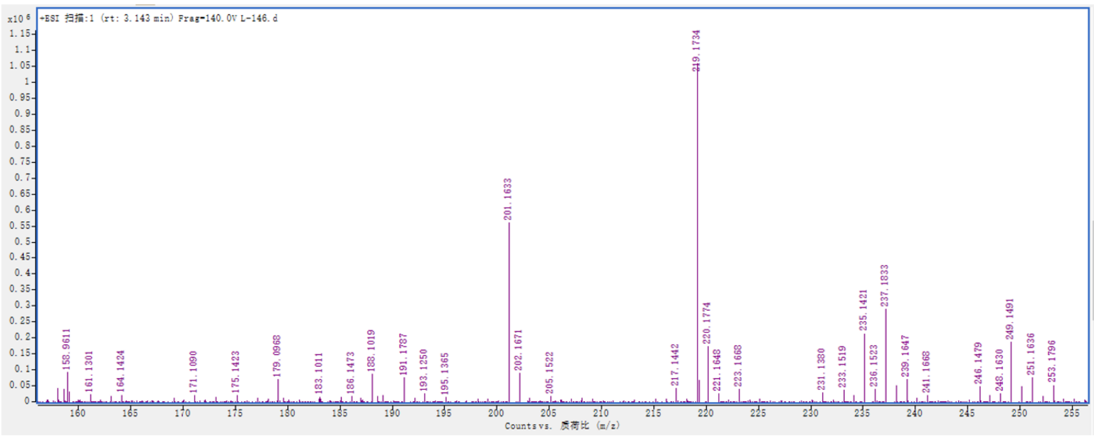

Formula Calculator Results

m/z	Calc m/z	Diff (mDa)	Diff (ppm)	Ion Formula	Ion
237.1833	237.1849	0.03	6.75	C15 H25 O2	$(M-H_2O+H)^+$

Figure S5-28. The HRESIMS spectrum of compound **4**

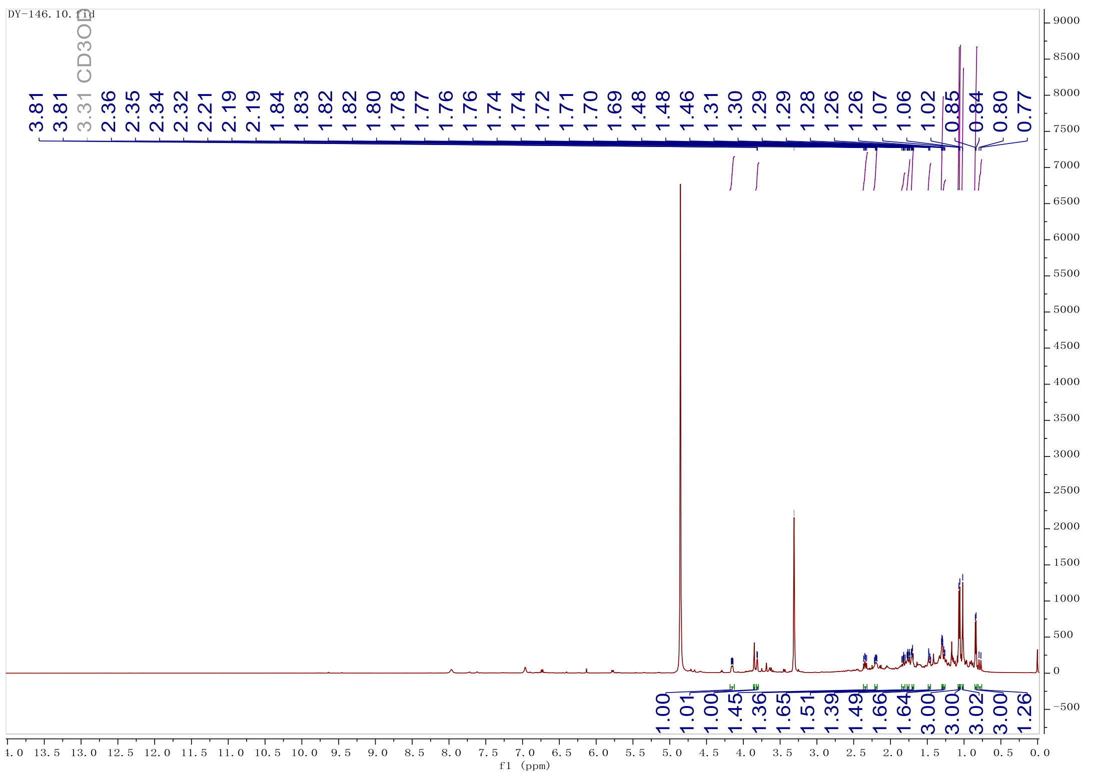

Figure S5-29. The ^{1}H NMR spectrum of compound **4** in CD_3OD (600 MHz)

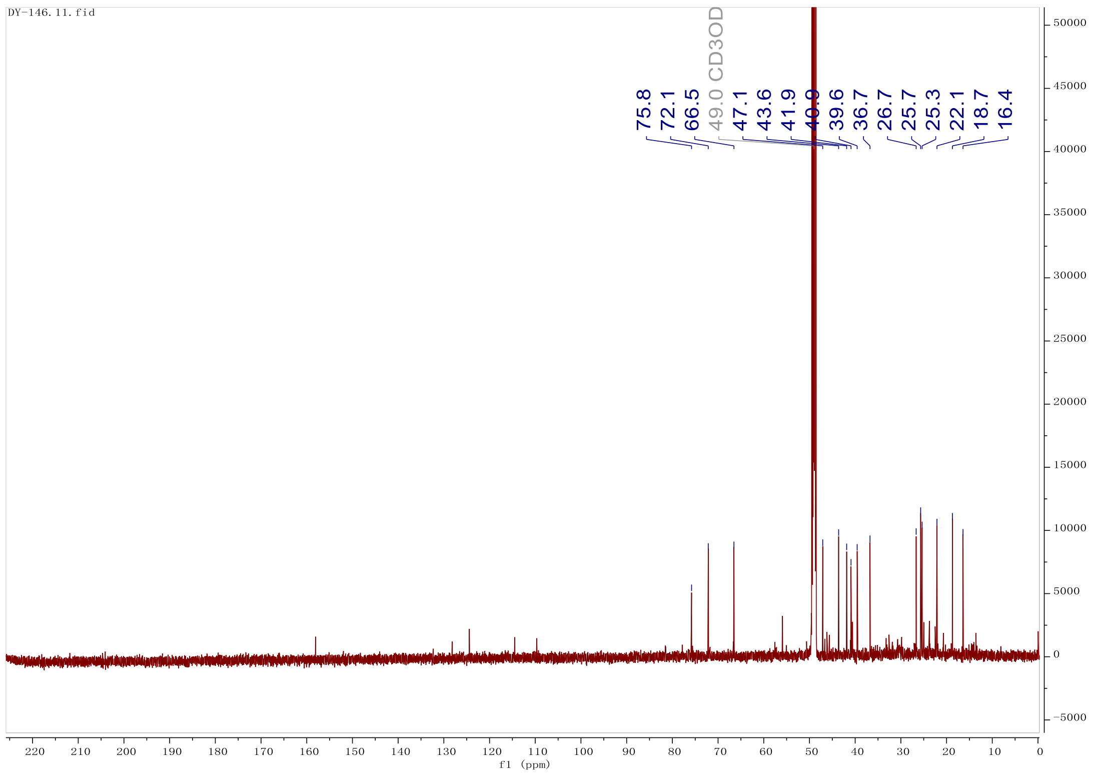

Figure S5-30. The ^{13}C NMR spectrum of compound **4** in CD_3OD (150 MHz)

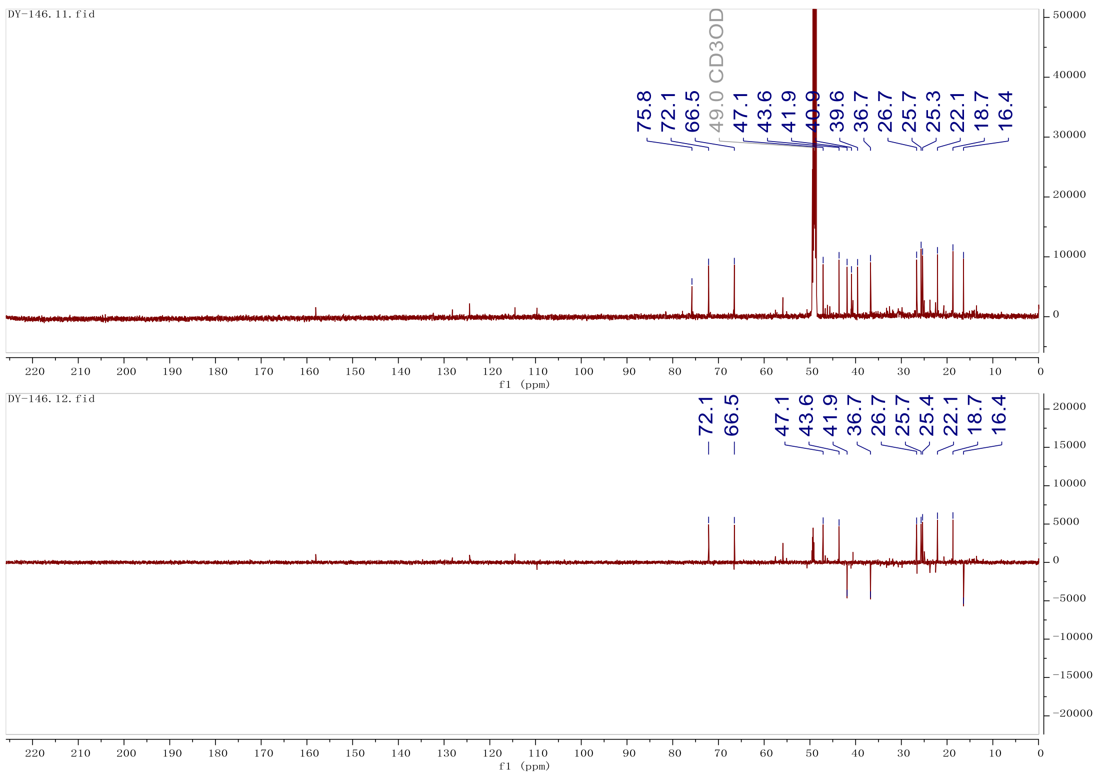

Figure S5-31. The DEPT 135 spectrum of compound **4** in CD_3OD (150 MHz)

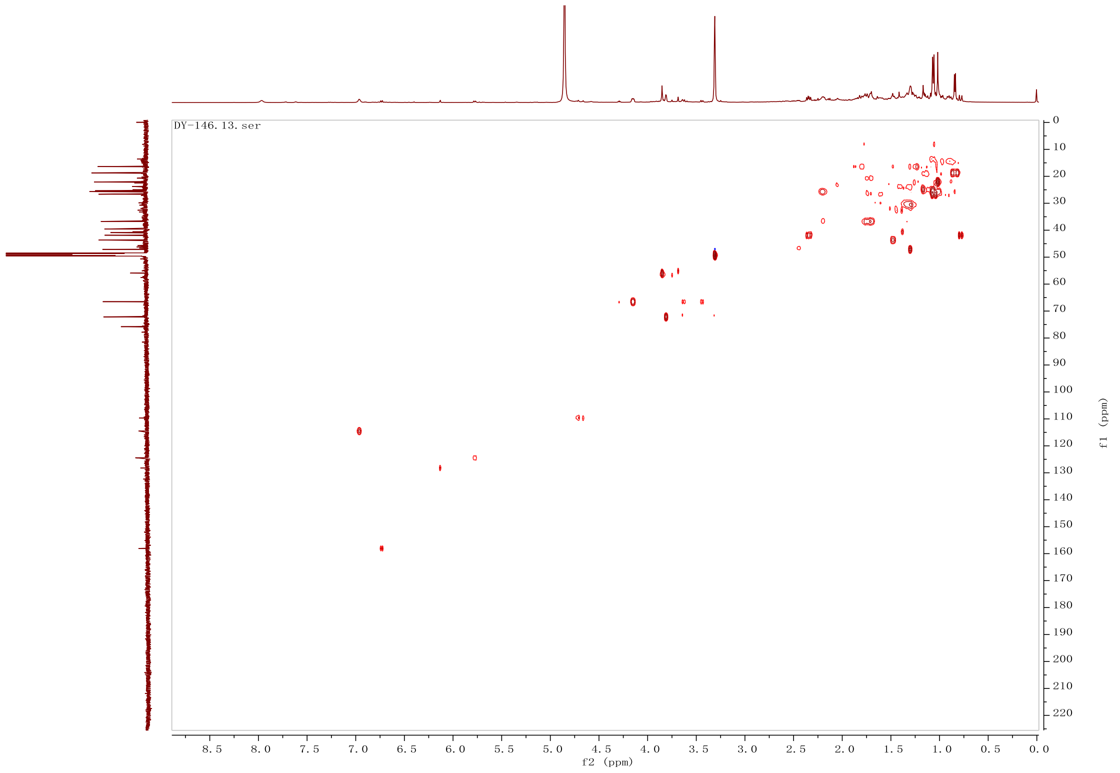

Figure S5-32. The HSQC spectrum of compound **4** in CD_3OD (600 MHz)

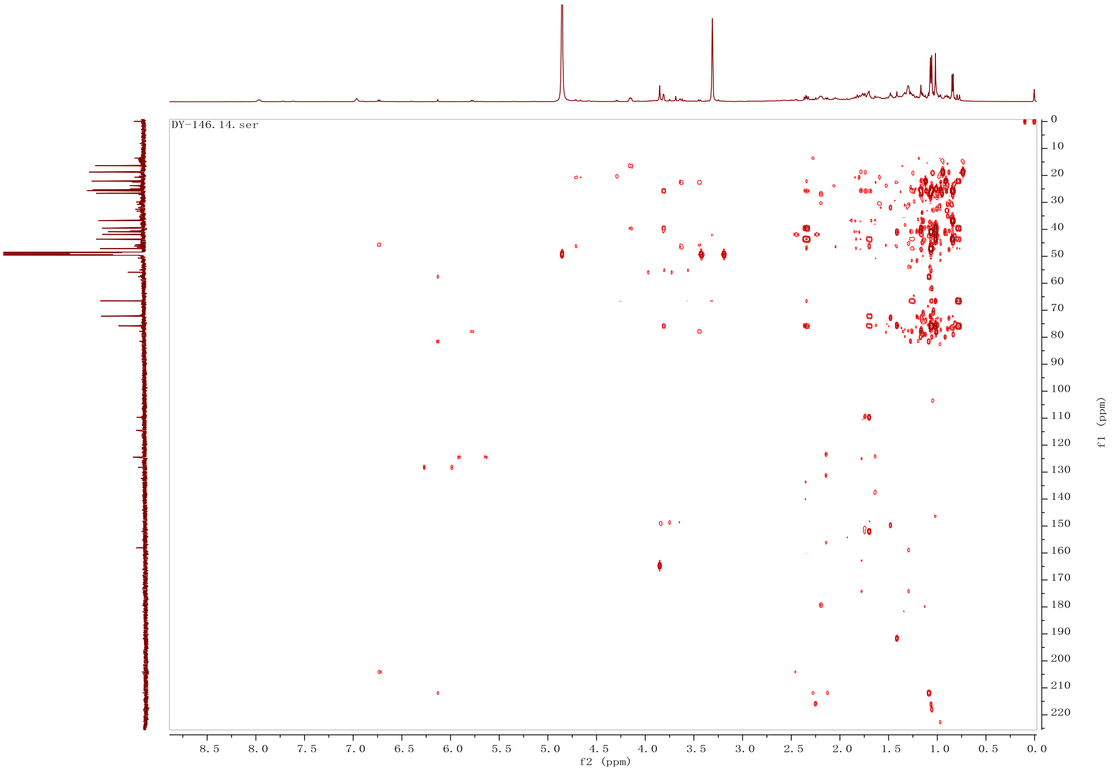

Figure S5-33. The HMBC spectrum of compound **4** in CD_3OD (600 MHz)

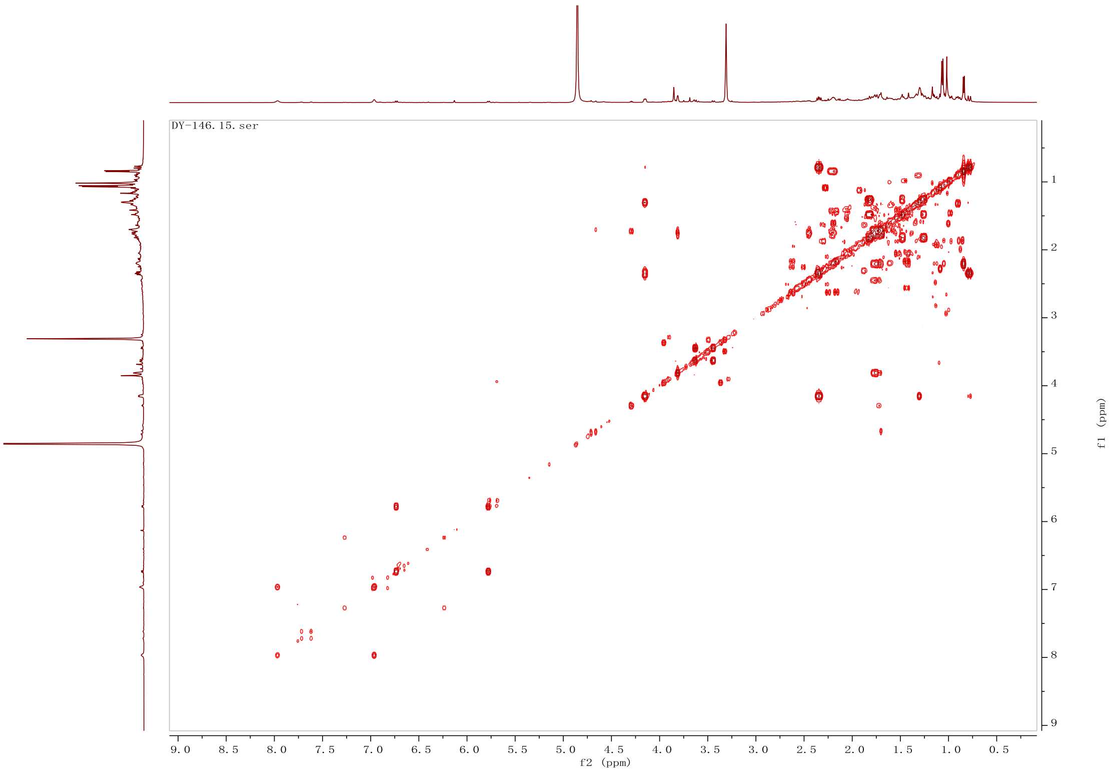

Figure S5-34. The ^1H-^1H COSY spectrum of compound **4** in CD_3OD (600 MHz)

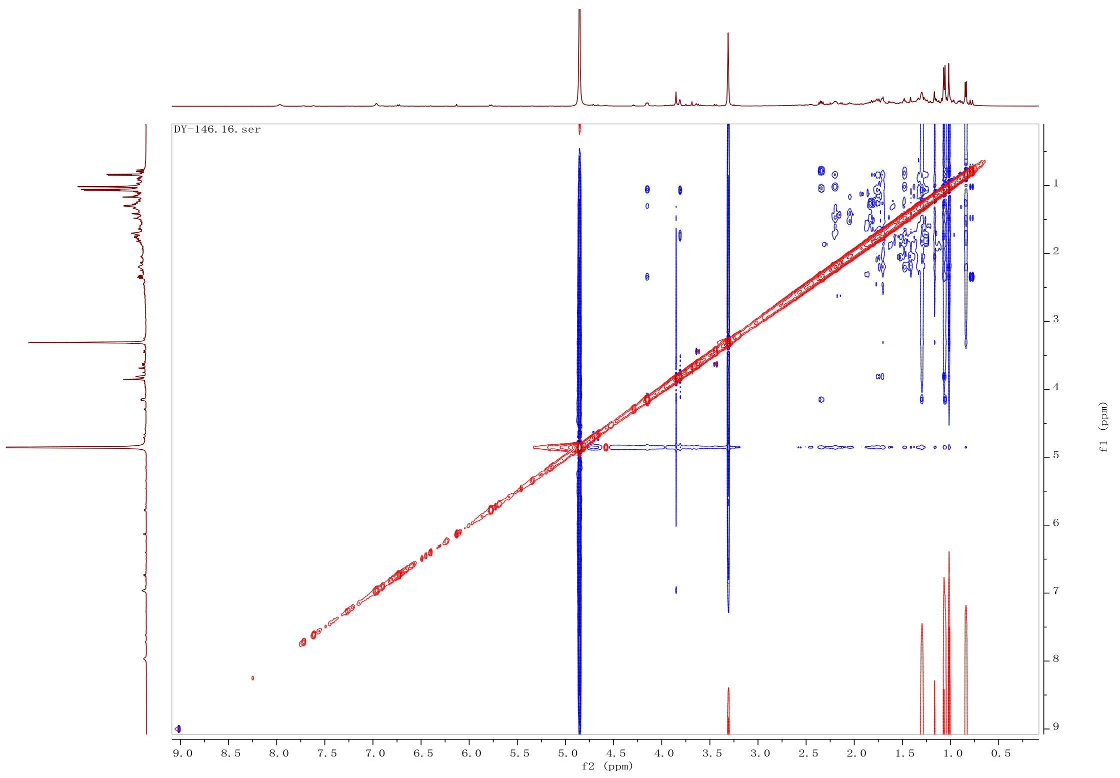

Figure S5-35. The NOESY spectrum of compound **4** in CD_3OD (600 MHz)

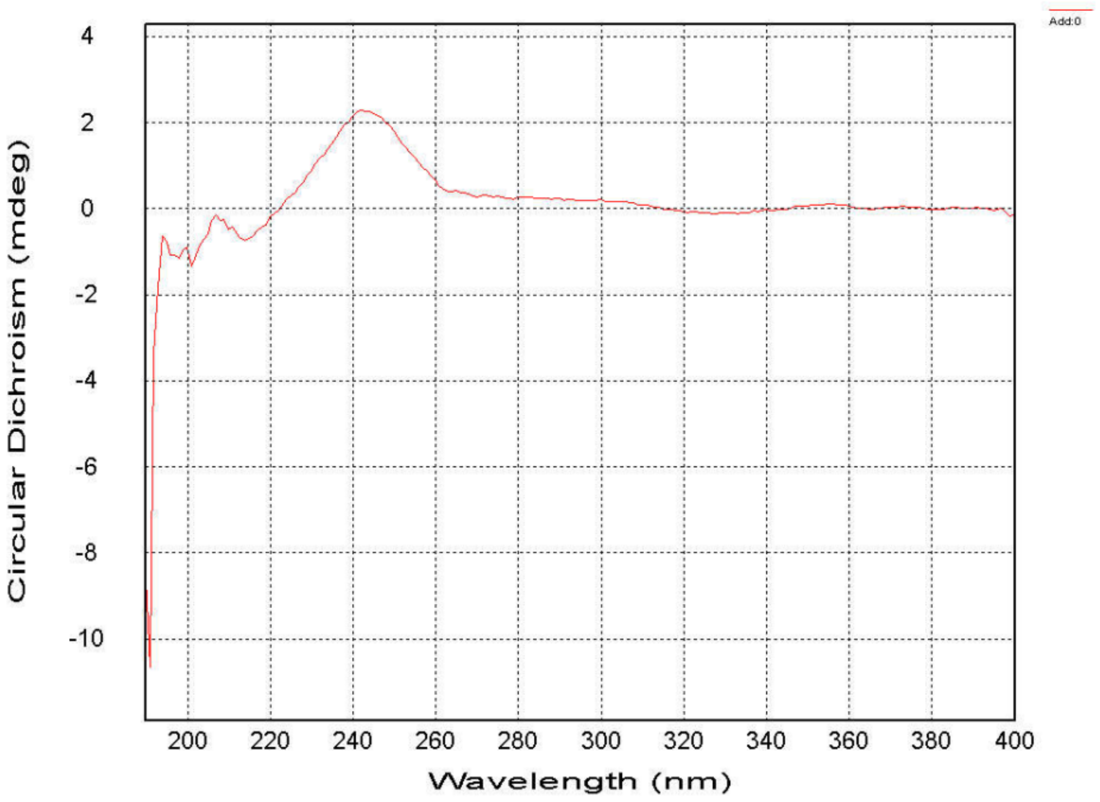

Figure S5-36. The ECD spectrum of compound **4** in MeOH

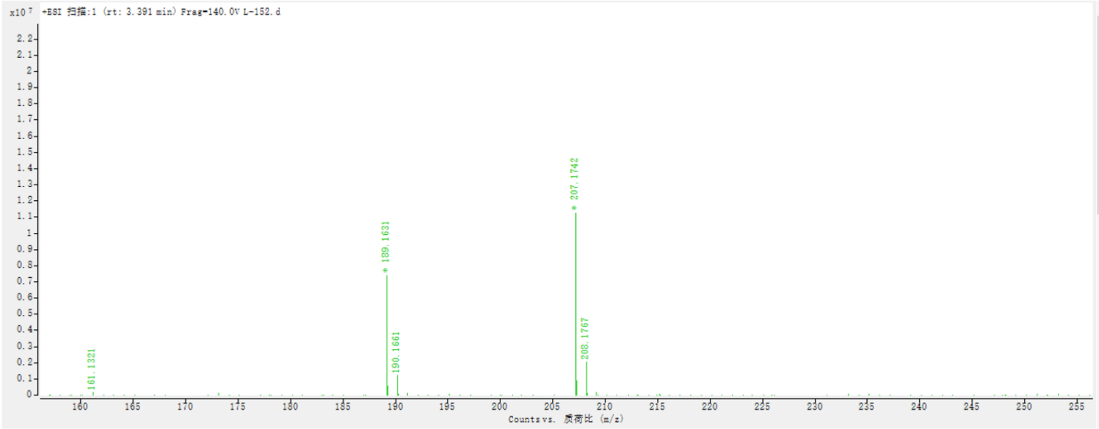

Formula Calculator Results

m/z	Calc m/z	Diff (mDa)	Diff (ppm)	Ion Formula	Ion
207.1742	207.1743	0.17	0.48	C14 H23 O	$(M-H_2O+H)^+$

Figure S5-37. The HRESIMS spectrum of compound **5**

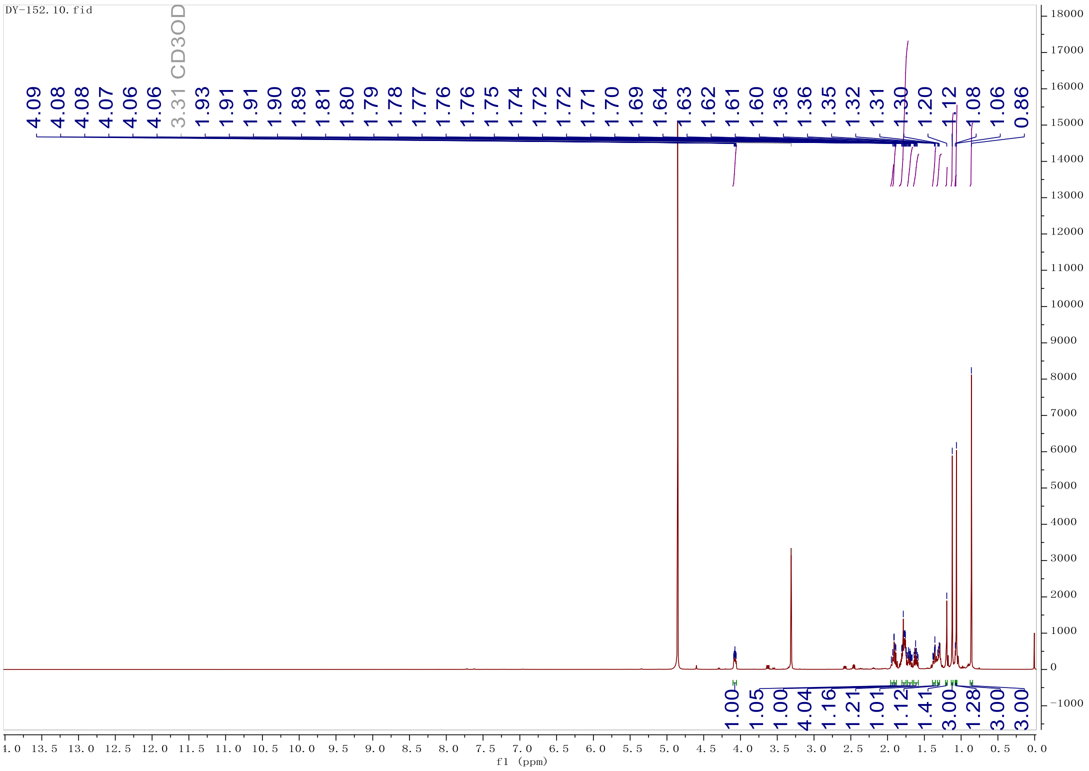

Figure S5-38. The ^{1}H NMR spectrum of compound **5** in CD_3OD (600 MHz)

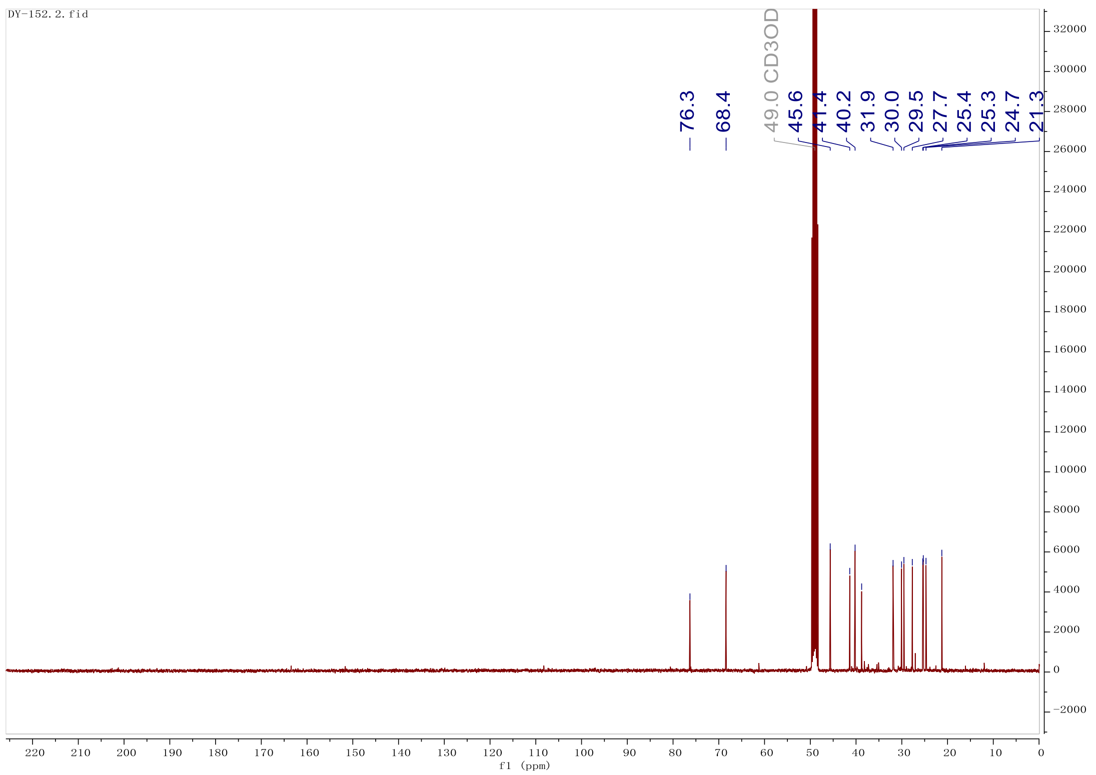

Figure S5-39. The ^{13}C NMR spectrum of compound **5** in CD_3OD (150 MHz)

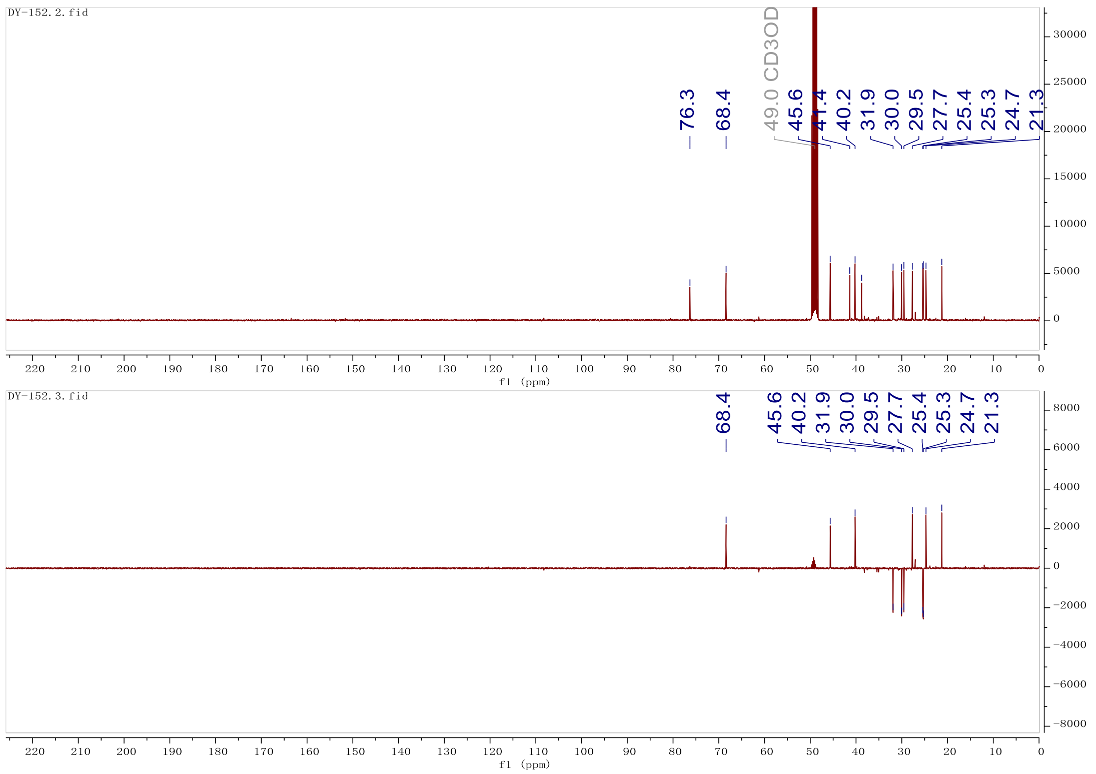

Figure S5-40. The DEPT 135 spectrum of compound **5** in CD_3OD (150 MHz)

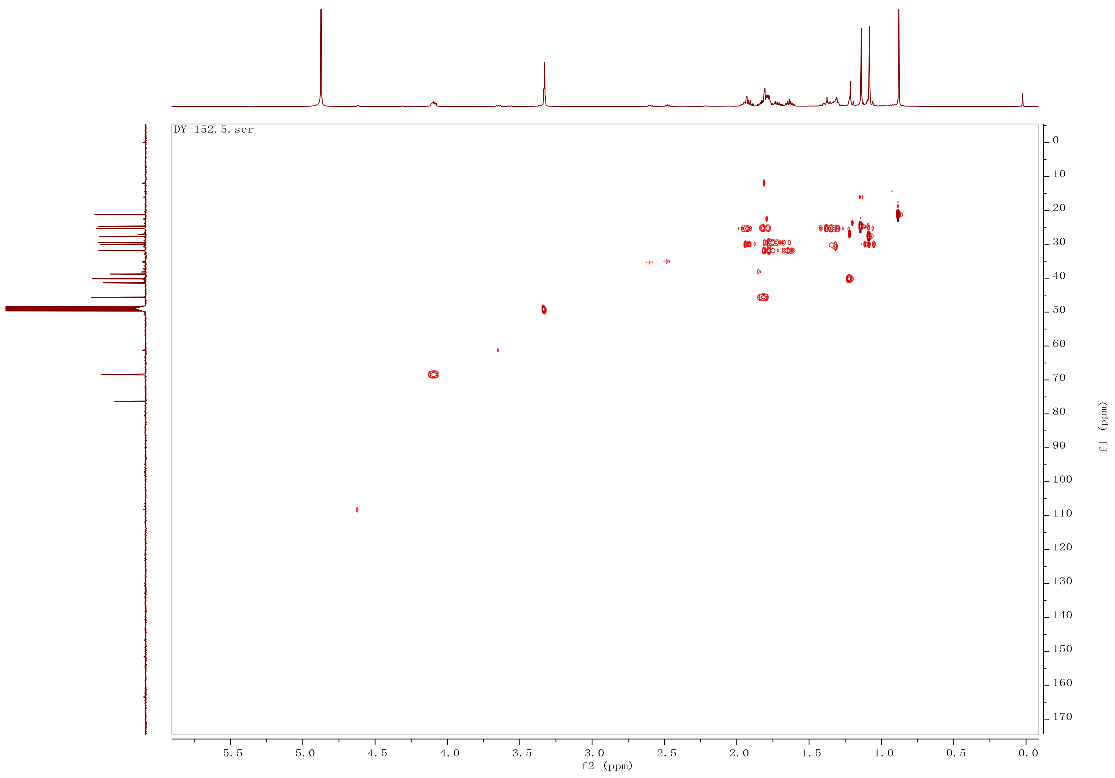

Figure S5-41. The HSQC spectrum of compound **5** in CD_3OD (600 MHz)

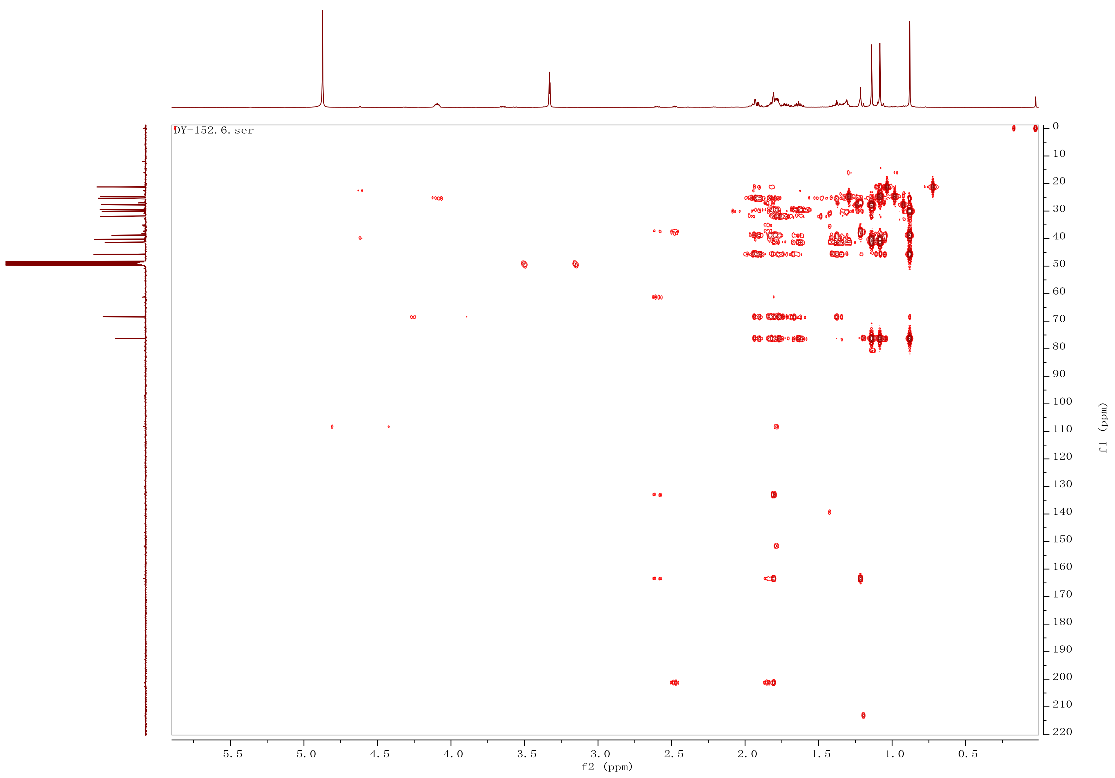

Figure S5-42. The HMBC spectrum of compound **5** in CD_3OD (600 MHz)

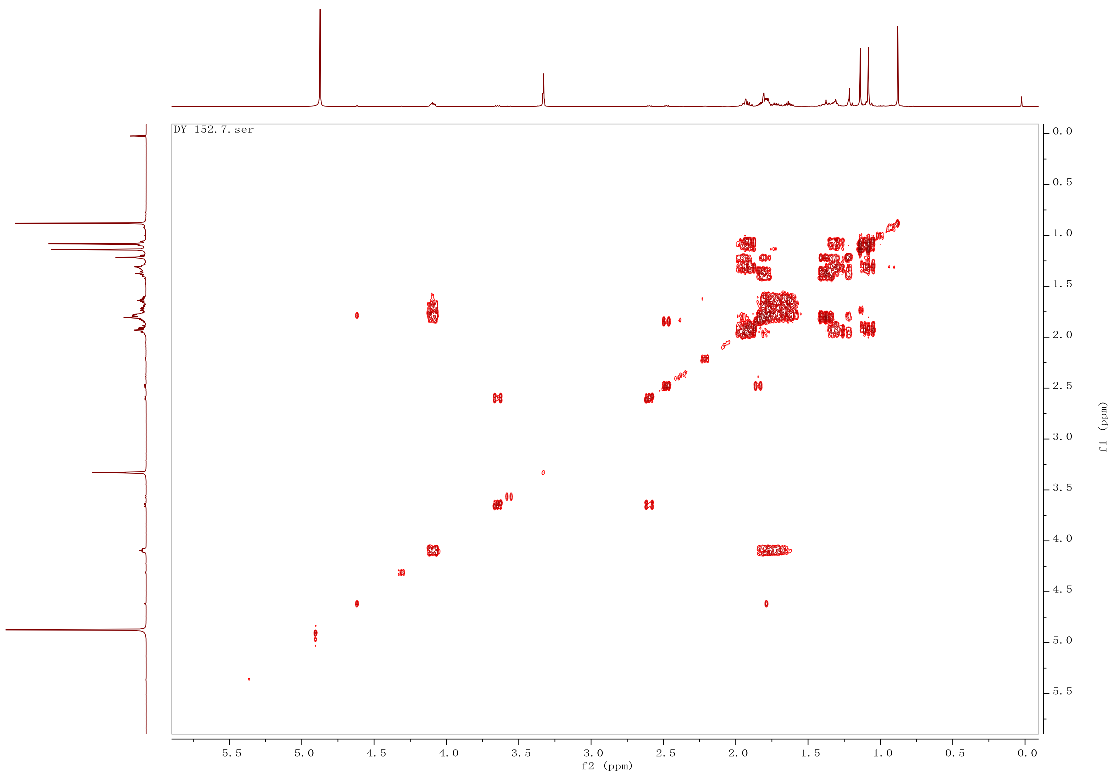

Figure S5-43. The ^{1}H-^{1}H COSY spectrum of compound **5** in CD_3OD (600 MHz)

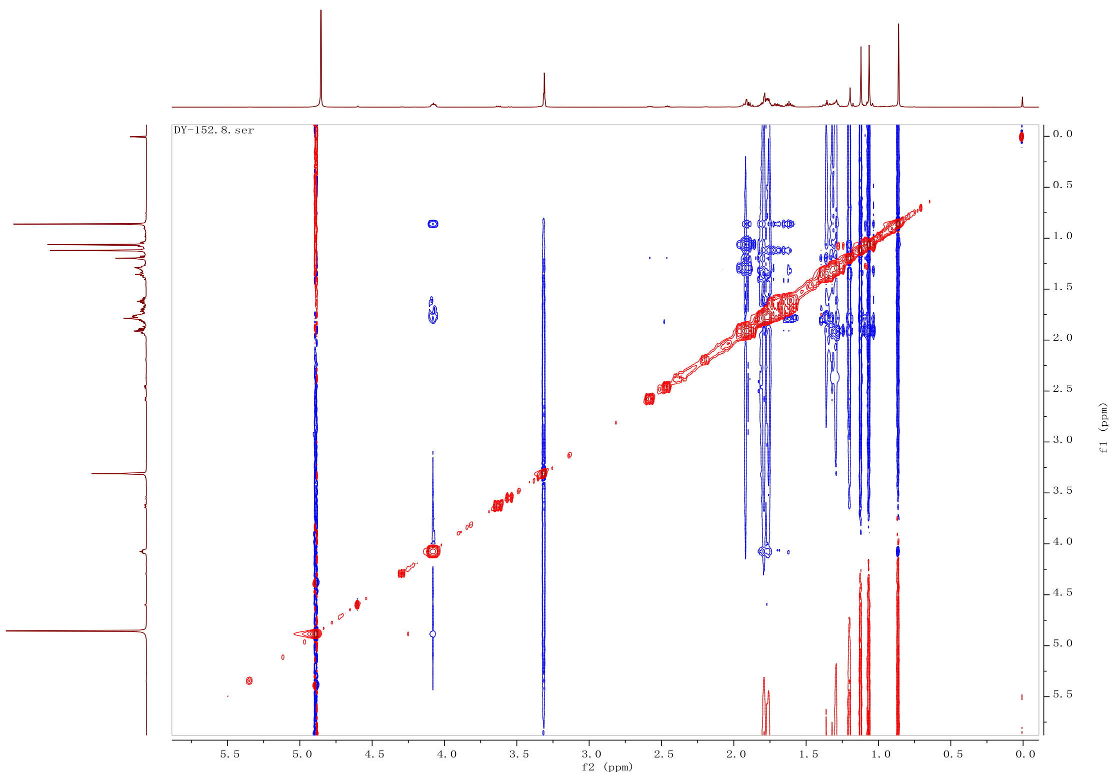

Figure S5-44. The NOESY spectrum of compound **5** in CD_3OD (600 MHz)

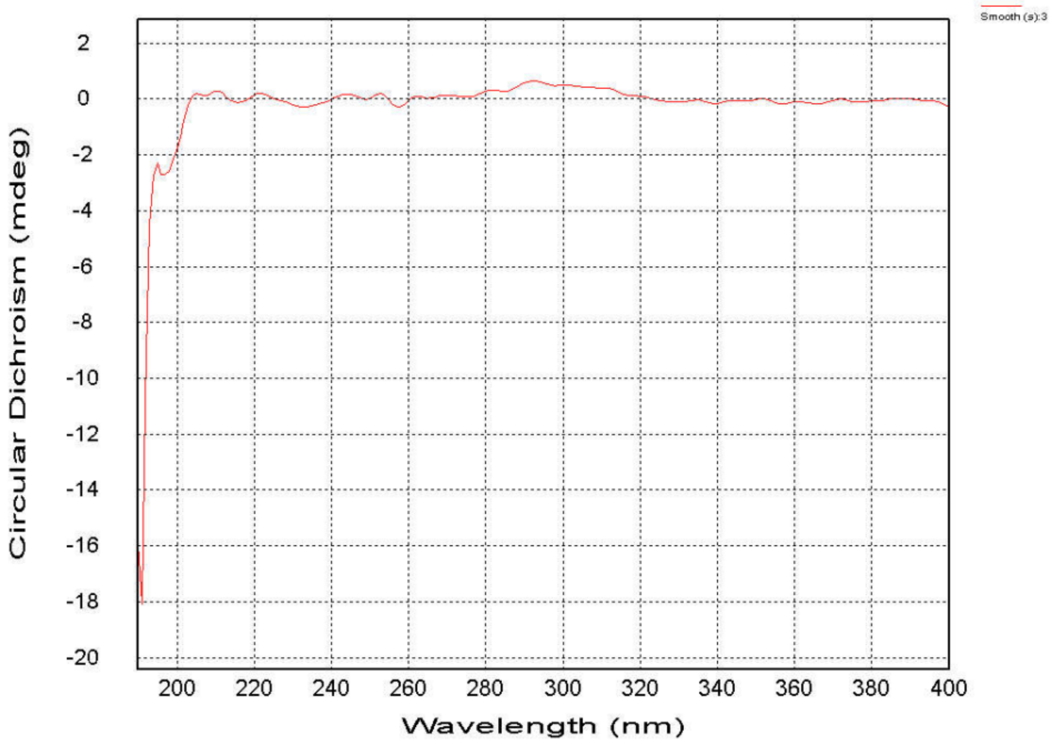

Figure S5-45. The ECD spectrum of compound **5** in MeOH

Qualitative Analysis Report

**Data Filename**	ESIH202306278-1.d	**Sample Name**	E0-DY-197
**Sample ID**		**Position**	P1-A5
**Instrument Name**	Agilent 6520 Q-TOF	**Acq Method**	20160322_MS_ESIH_POS_1min.m
**Acquired Time**	11/3/2023 10:12:44	**IRM Calibration Status**	Success
**DA Method**	small molecular data analysis method.m	**Comment**	ESIH by fangsu

User Spectra

**Fragmentor Voltage**	**Collision Energy**	**Ionization Mode**
170	0	ESI

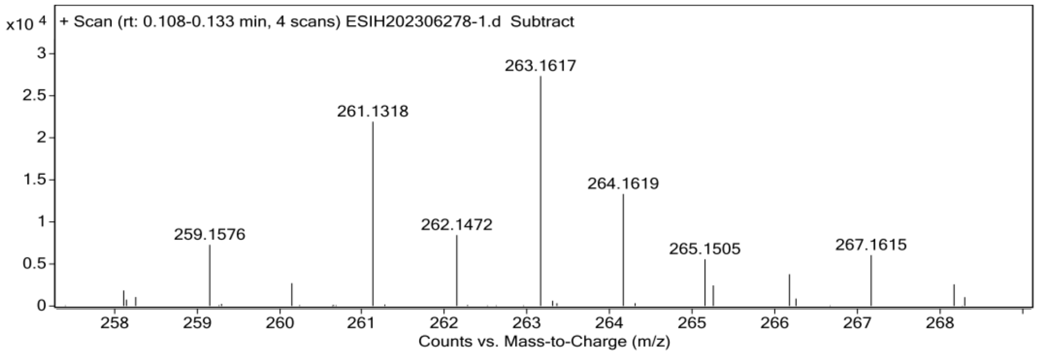

Formula Calculator Results

**m/z**	**Calc m/z**	**Diff (mDa)**	**Diff (ppm)**	**Ion Formula**	**Ion**
263.1617	263.1618	0.07	0.25	C14 H24 Na O3	(M+Na)+

--- End Of Report ---

Figure S5-46. The HRESIMS spectrum of compound **6**

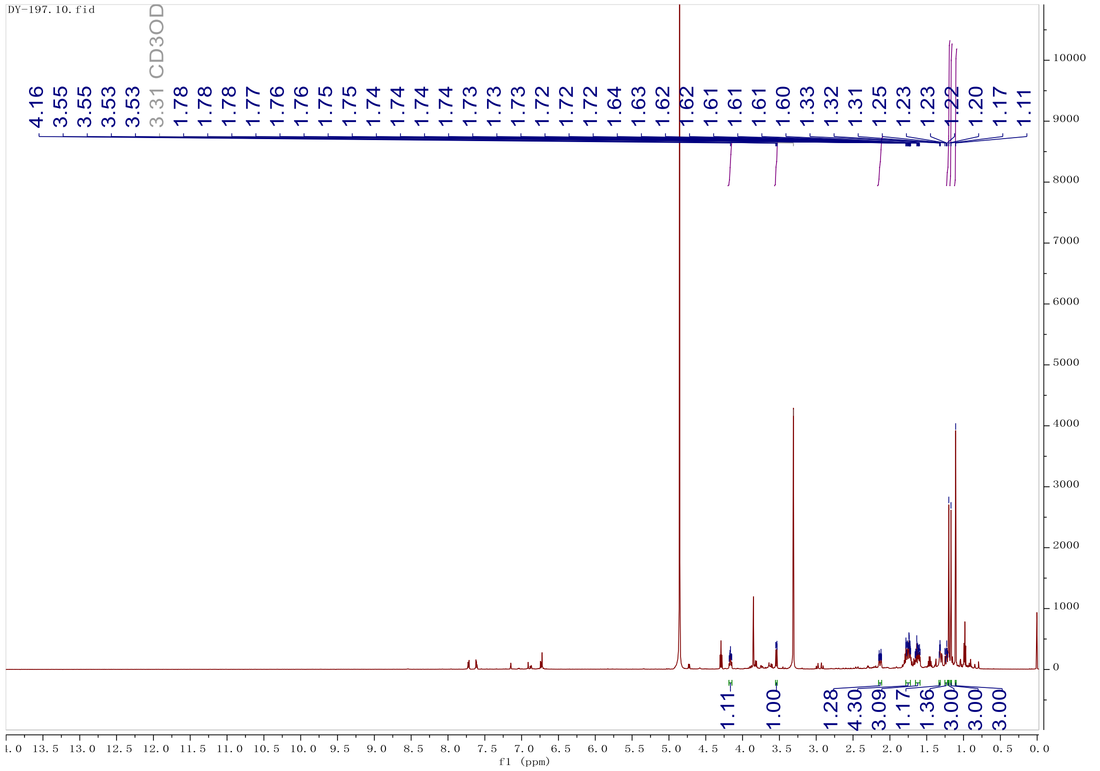

Figure S5-47. The ^{1}H NMR spectrum of compound **6** in CD_3OD (600 MHz)

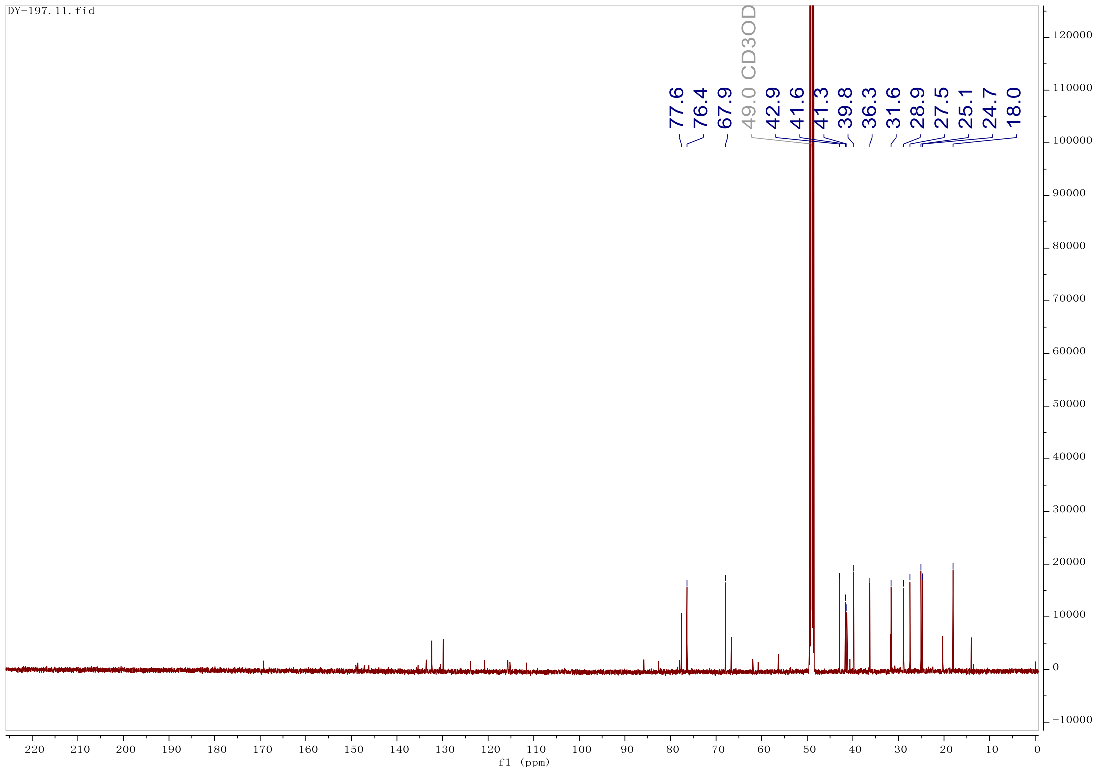

Figure S5-48. The ^{13}C NMR spectrum of compound **6** in CD_3OD (150 MHz)

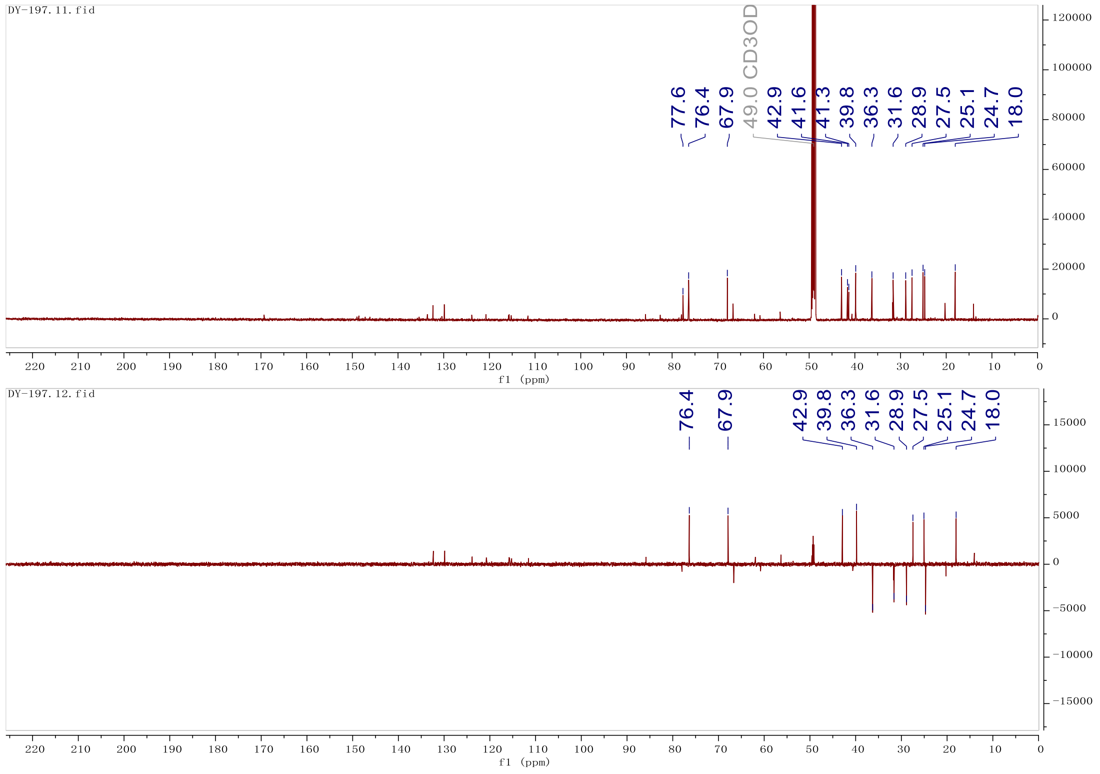

Figure S5-49. The DEPT 135 spectrum of compound **6** in CD_3OD (150 MHz)

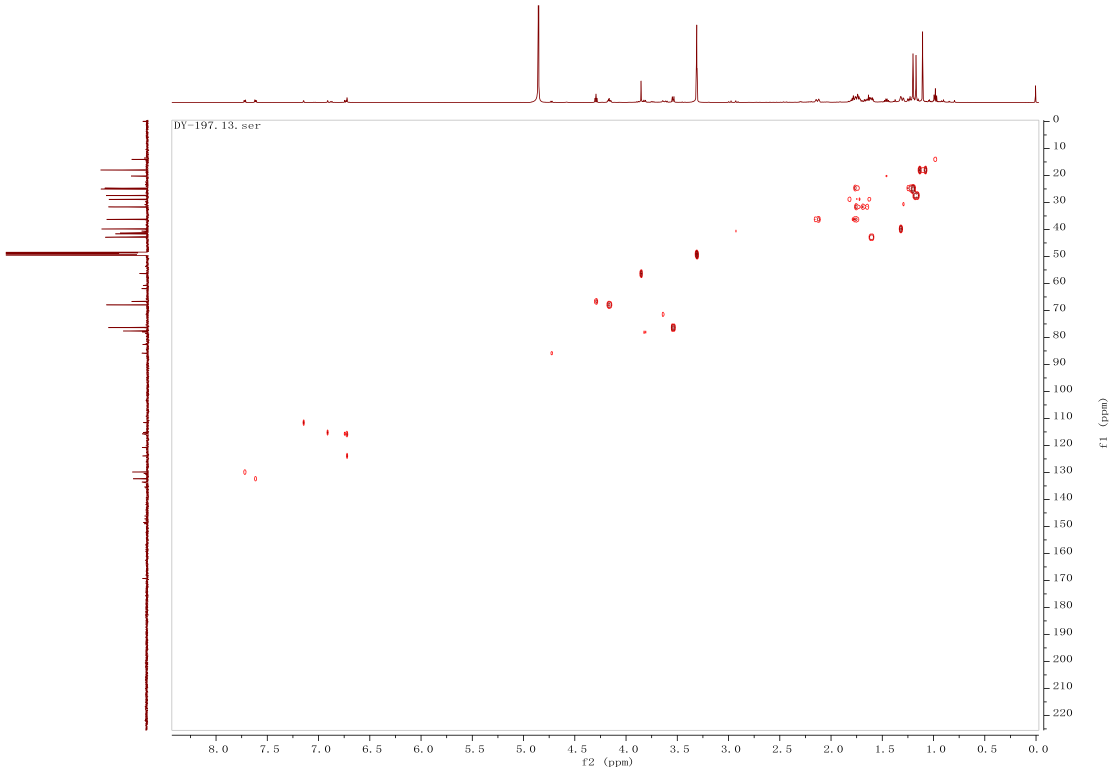

Figure S5-50. The HSQC spectrum of compound **6** in CD_3OD (600 MHz)

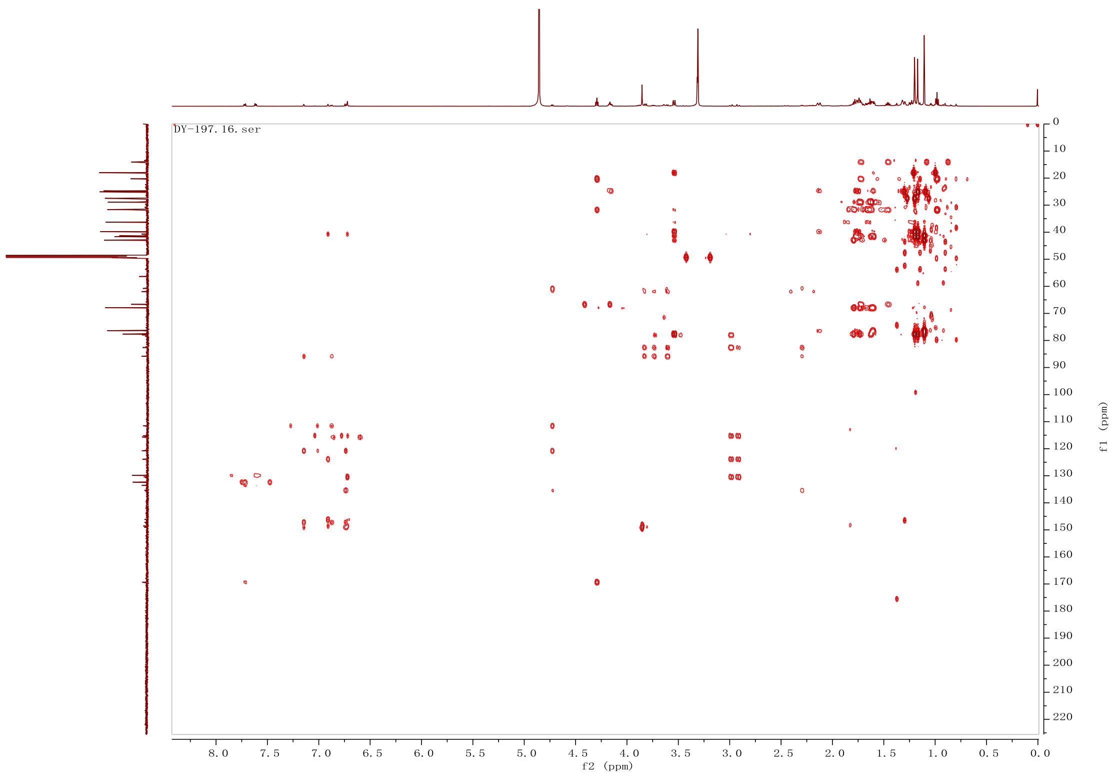

Figure S5-51. The HMBC spectrum of compound **6** in CD_3OD (600 MHz)

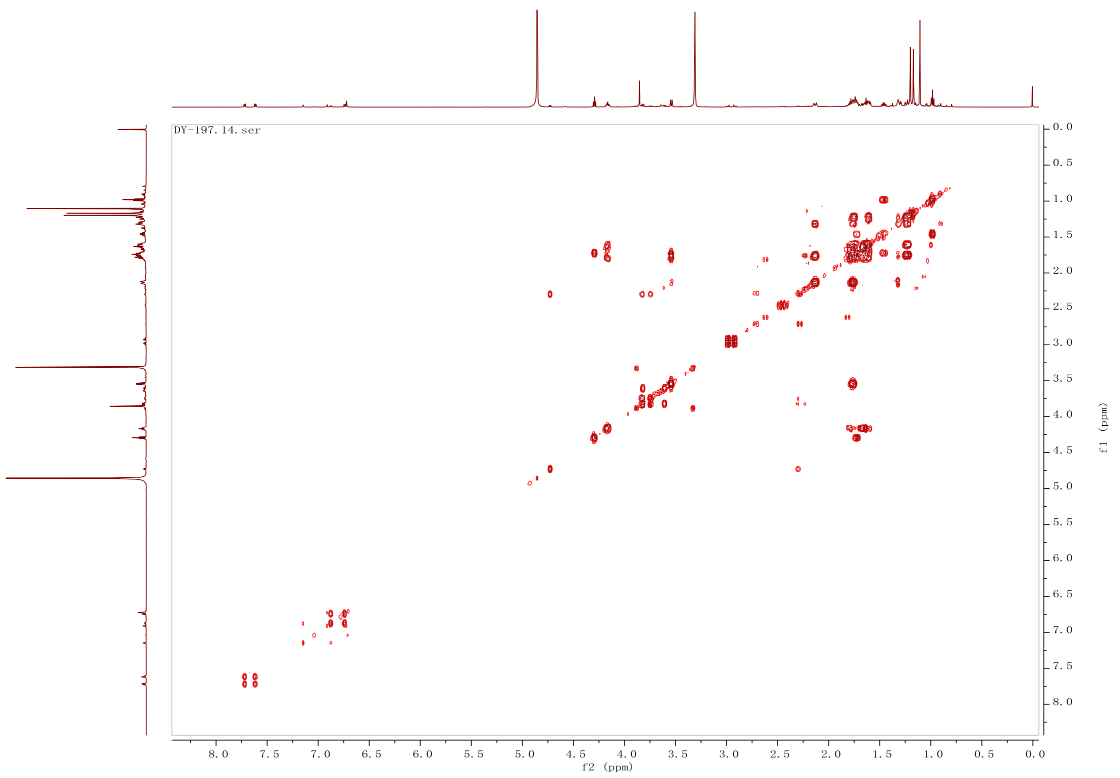

Figure S5-52. The ^1H-^1H COSY spectrum of compound **6** in CD_3OD (600 MHz)

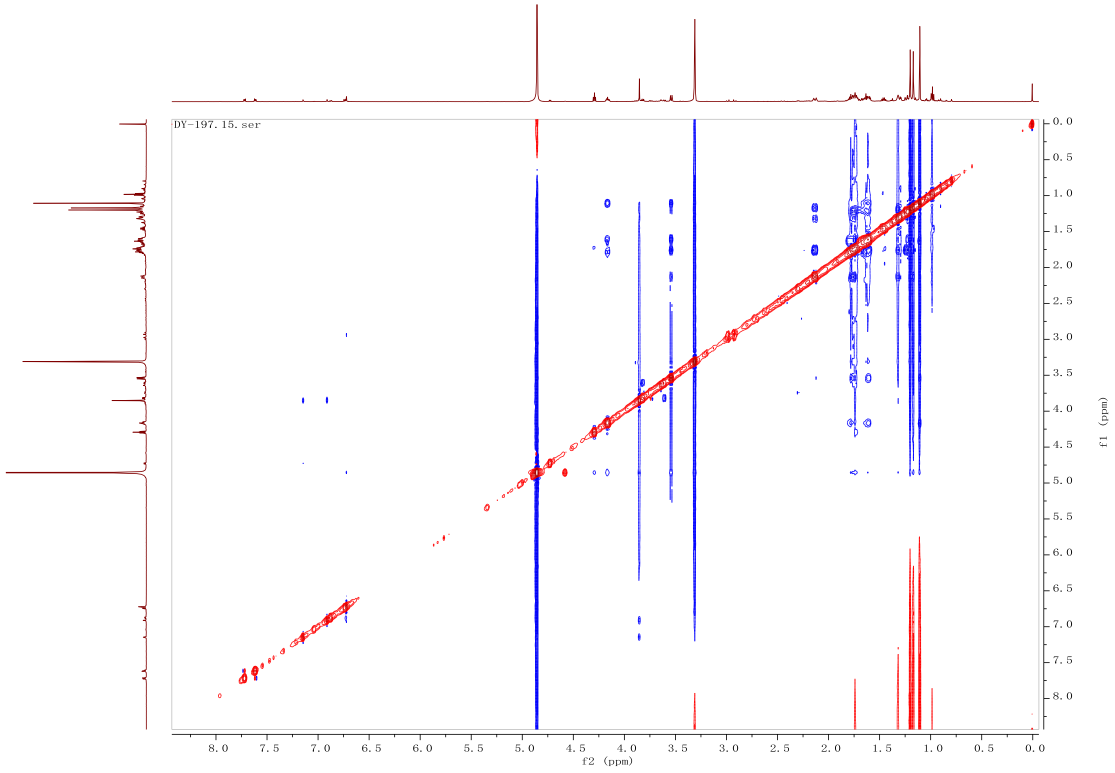

Figure S5-53 The NOESY spectrum of compound **6** in CD_3OD (600 MHz)

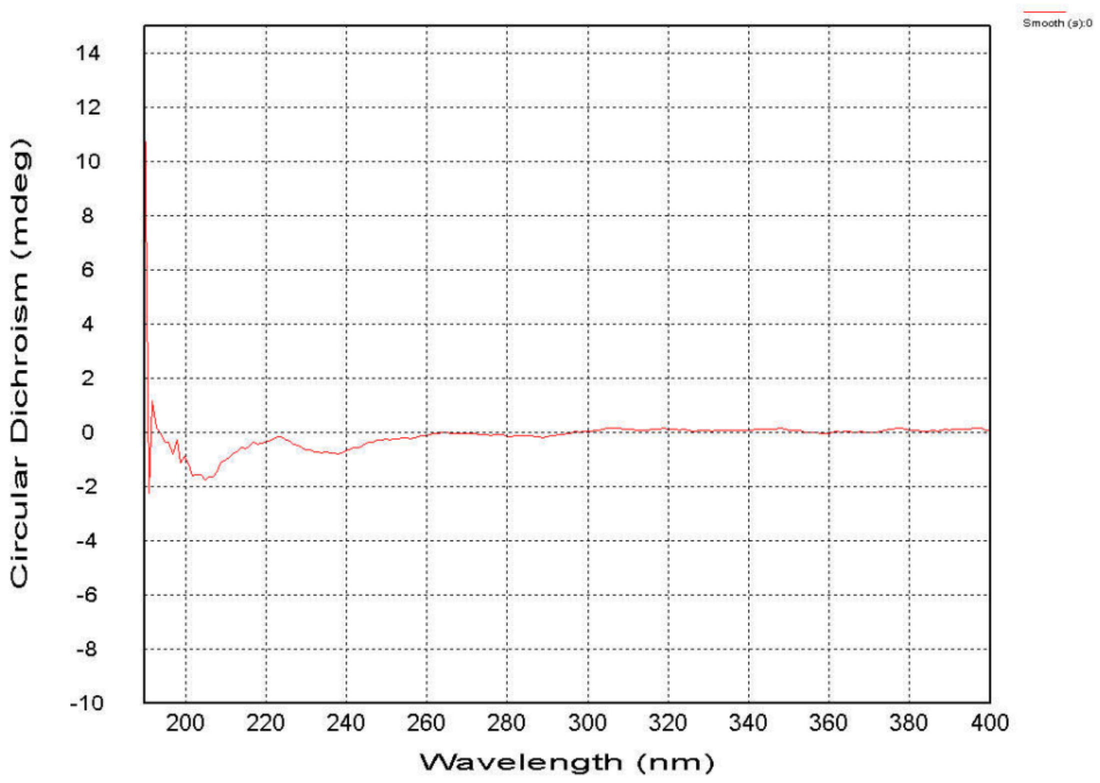

Figure S5-54. The ECD spectrum of compound **6** in MeOH

Qualitative Analysis Report

**Data Filename**	ESIH202304967.d	**Sample Name**	E0-DY-143A
**Sample ID**		**Position**	P1-A1
**Instrument Name**	Agilent 6520 Q-TOF	**Acq Method**	20160322_MS_ESIH_POS_1min.m
**Acquired Time**	8/23/2023 14:16:50	**IRM Calibration Status**	Success
**DA Method**	small molecular data analysis method.m	**Comment**	ESIH by fangsu

User Spectra

Fragmentor Voltage	Collision Energy	Ionization Mode
170	0	ESI

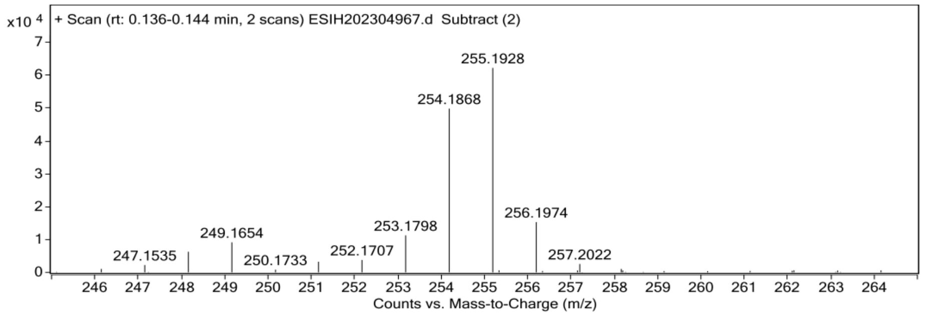

Formula Calculator Results

m/z	Calc m/z	Diff (mDa)	Diff (ppm)	Ion Formula	Ion
253.1798	253.1798	0.06	0.23	C15 H25 O3	(M+H)+

--- End Of Report ---

Figure S5-55. The HRESIMS spectrum of compound **7**

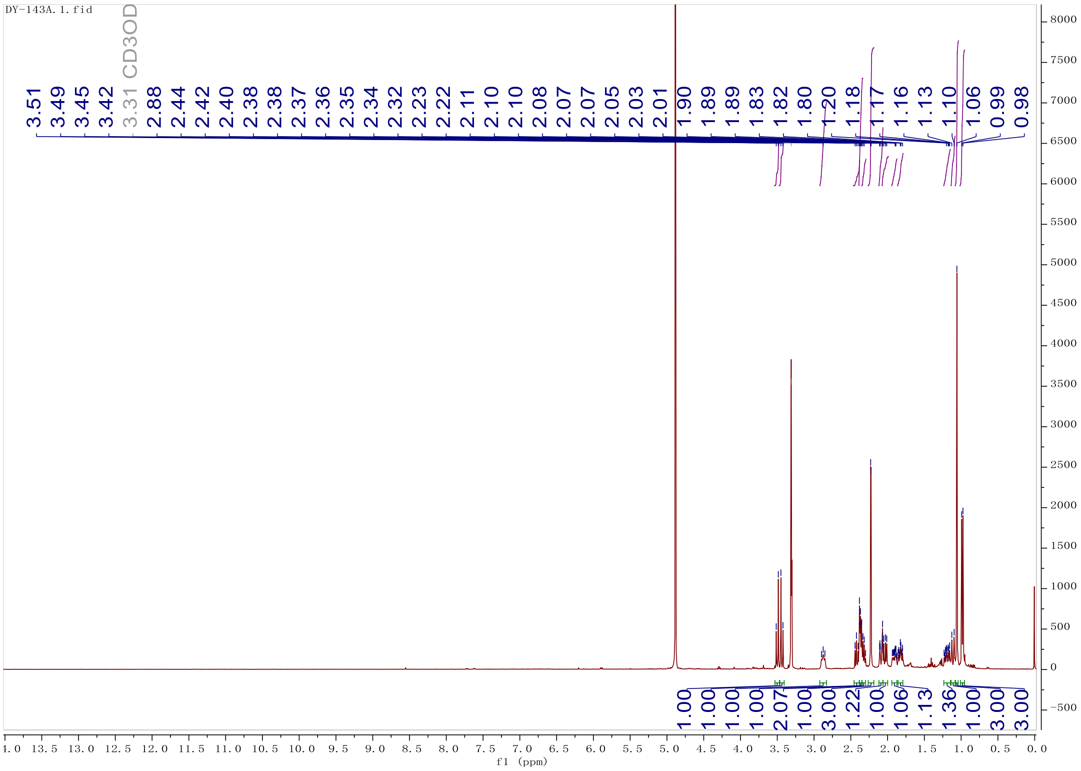

Figure S5-56. The ^{1}H NMR spectrum of compound **7** in CD_3OD (600 MHz)

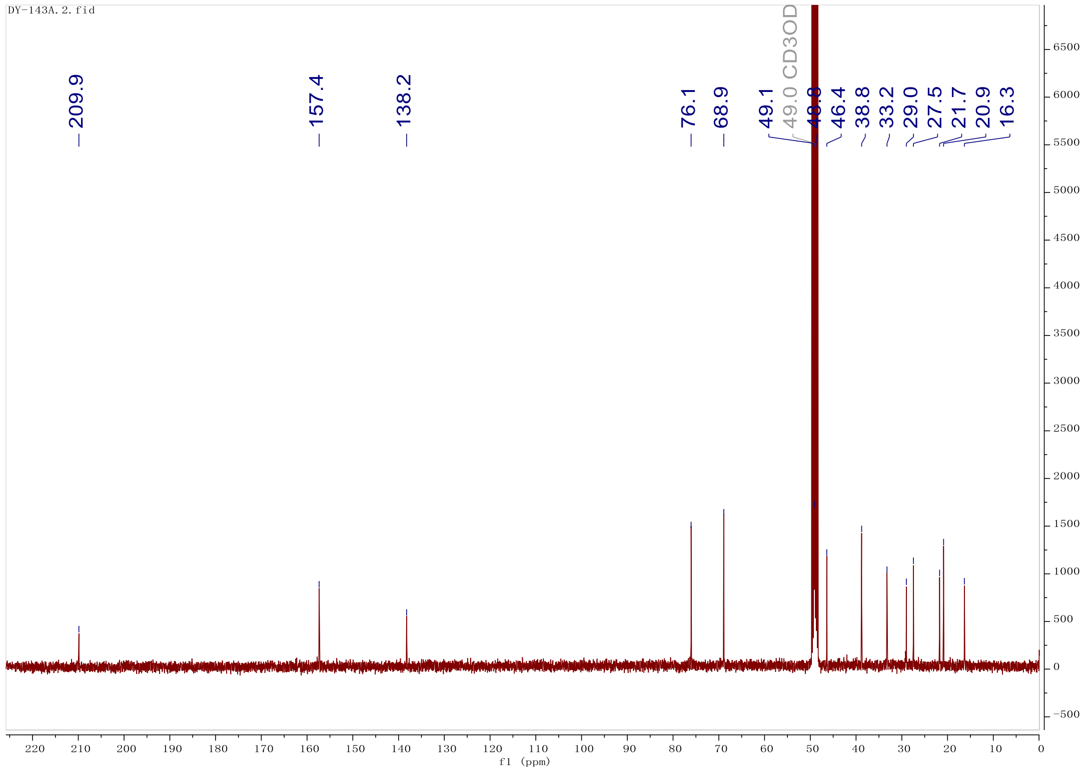

Figure S5-57. The ^{13}C NMR spectrum of compound **7** in CD_3OD (150 MHz)

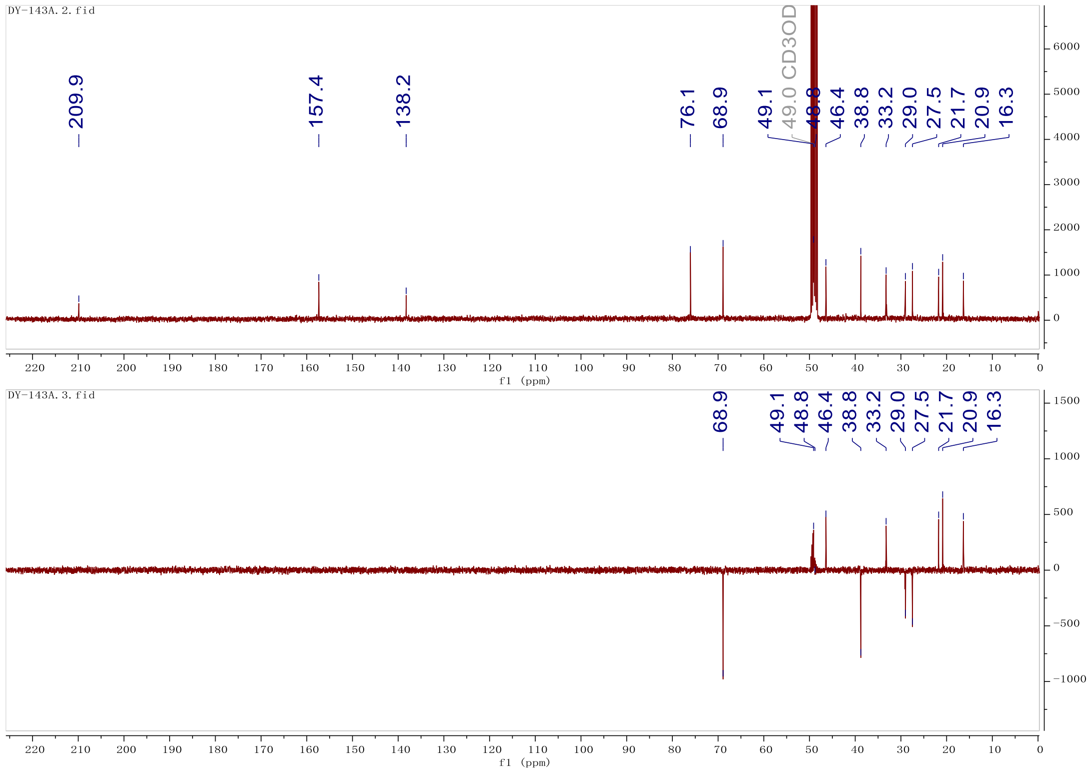

Figure S5-58. The DEPT 135 spectrum of compound **7** in CD_3OD (150 MHz)

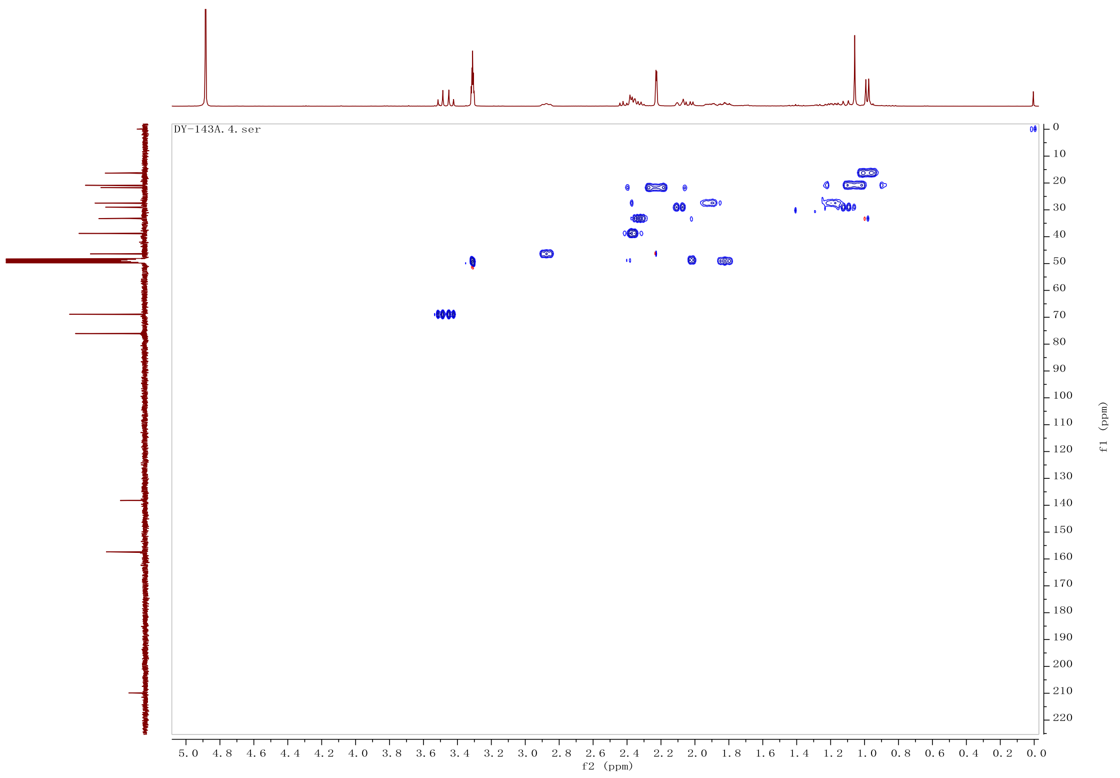

Figure S5-59. The HSQC spectrum of compound **7** in CD_3OD (600 MHz)

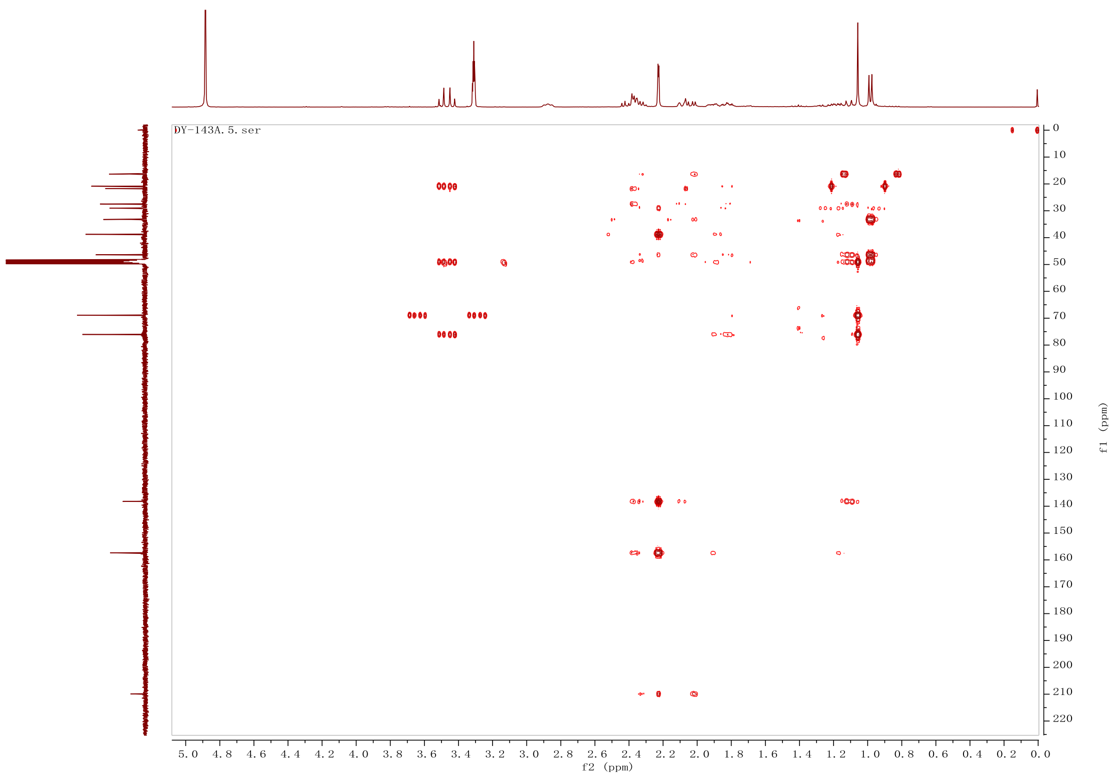

Figure S5-60. The HMBC spectrum of compound **7** in CD_3OD (600 MHz)

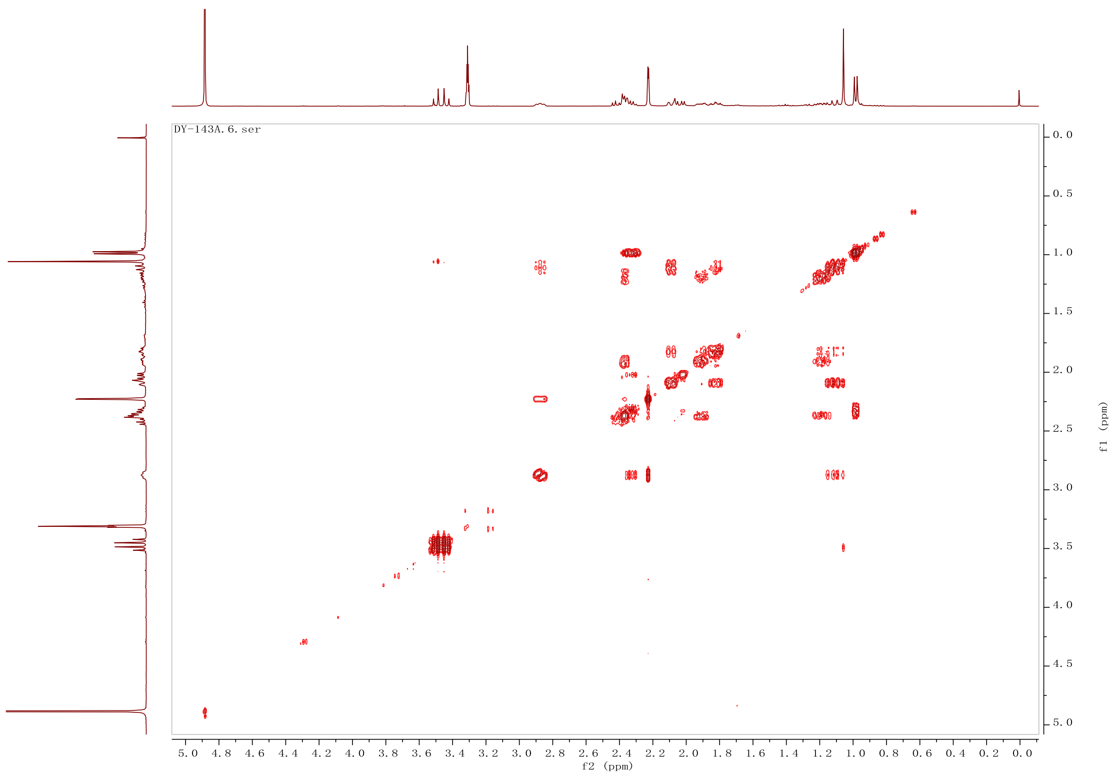

Figure S5-61. The ^{1}H-^{1}H COSY spectrum of compound **7** in CD_3OD (600 MHz)

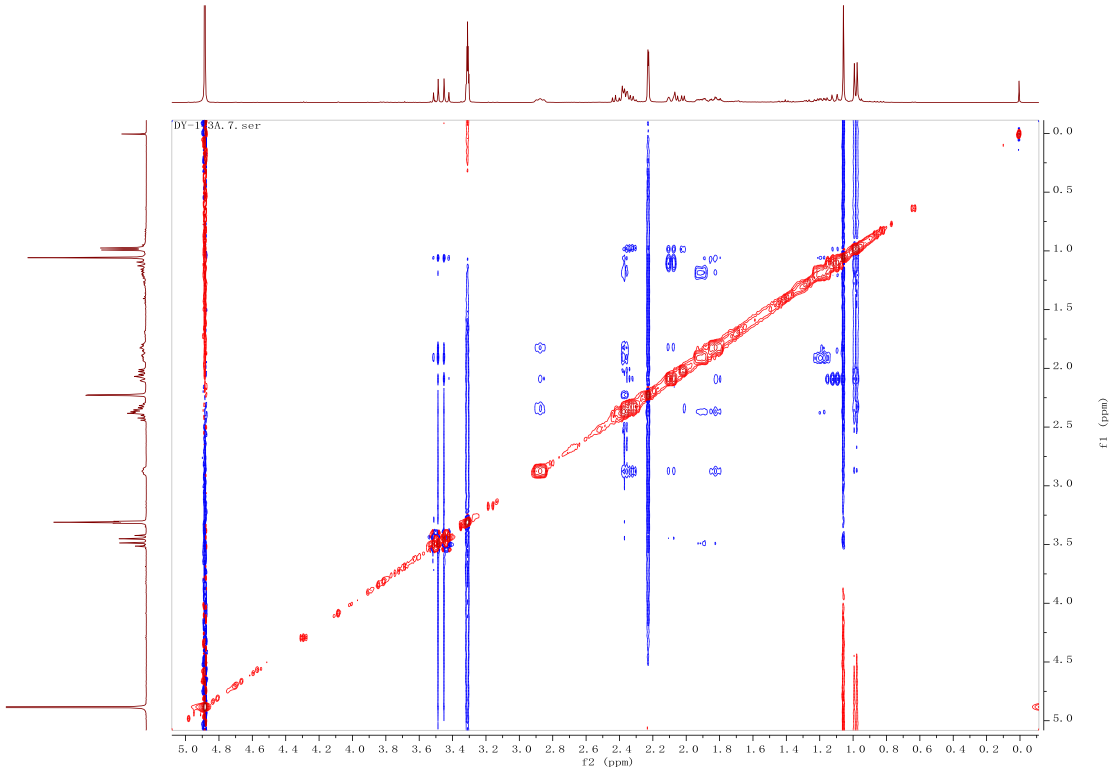

Figure S5-62. The NOESY spectrum of compound **7** in CD_3OD (600 MHz)

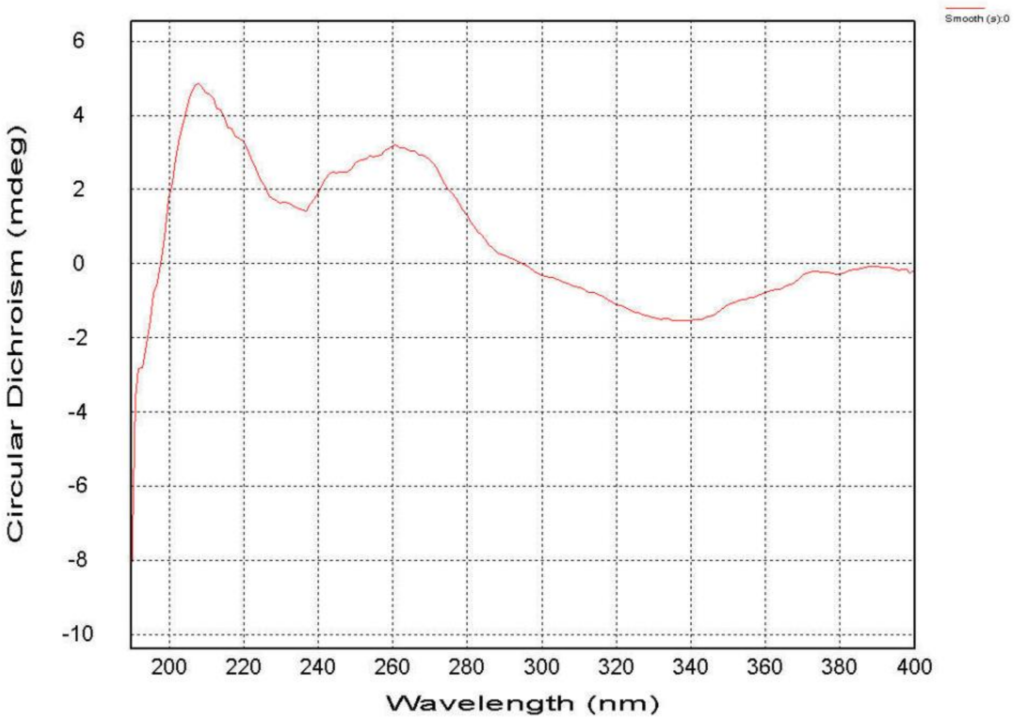

Figure S5-63. The ECD spectrum of compound **7** in MeOH

Qualitative Analysis Report

**Data Filename**	ESIH202304968.d	**Sample Name**	E0-DY-143B
**Sample ID**		**Position**	P1-A2
**Instrument Name**	Agilent 6520 Q-TOF	**Acq Method**	20160322_MS_ESIH_POS_1min.m
**Acquired Time**	8/23/2023 14:18:15	**IRM Calibration Status**	Success
**DA Method**	small molecular data analysis method.m	**Comment**	ESIH by fangsu

User Spectra

Fragmentor Voltage	Collision Energy	Ionization Mode
170	0	ESI

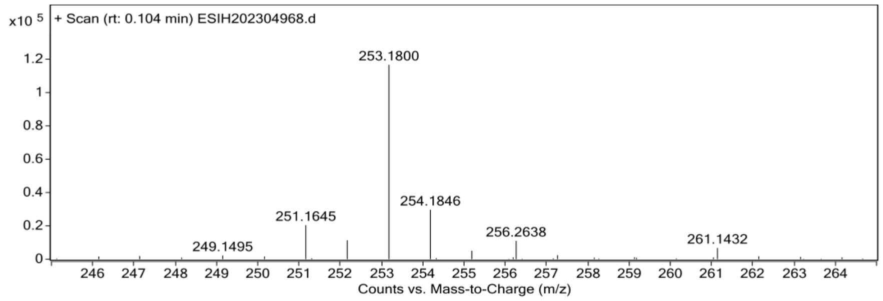

Formula Calculator Results

m/z	Calc m/z	Diff (mDa)	Diff (ppm)	Ion Formula	Ion
253.18	253.1798	-0.21	-0.85	C15 H25 O3	(M+H)+

--- End Of Report ---

Figure S5-64. The HRESIMS spectrum of compound **8**

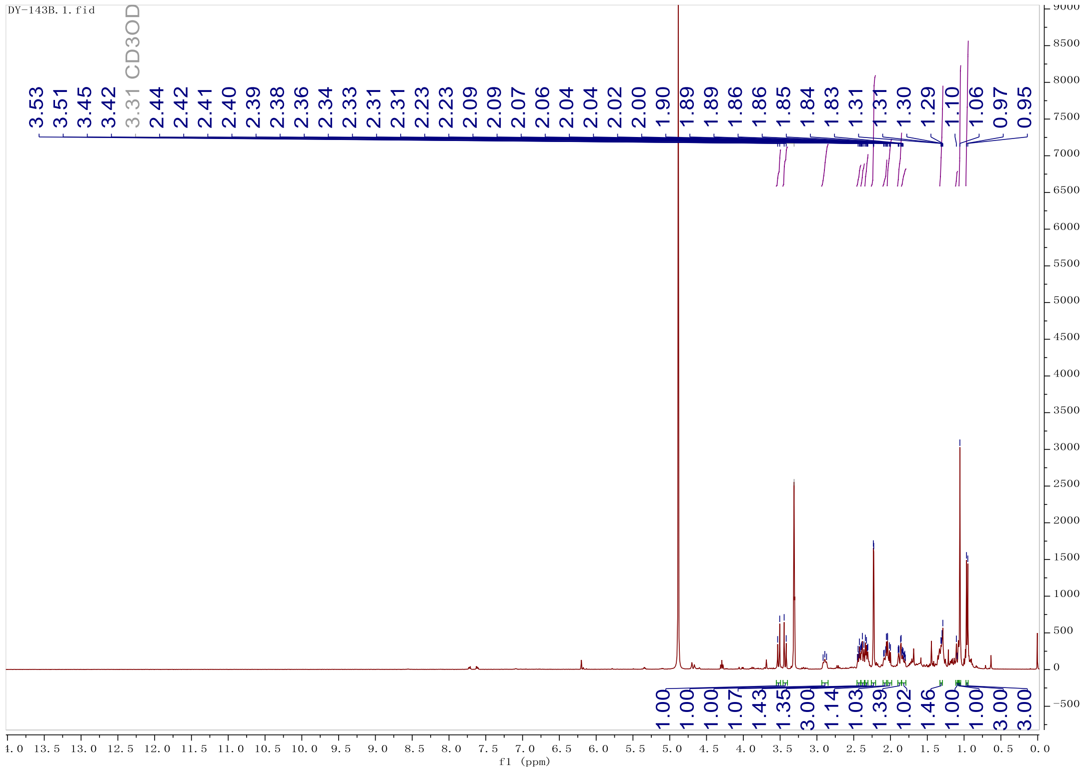

Figure S5-65. The ^{1}H NMR spectrum of compound **8** in CD_3OD (600 MHz)

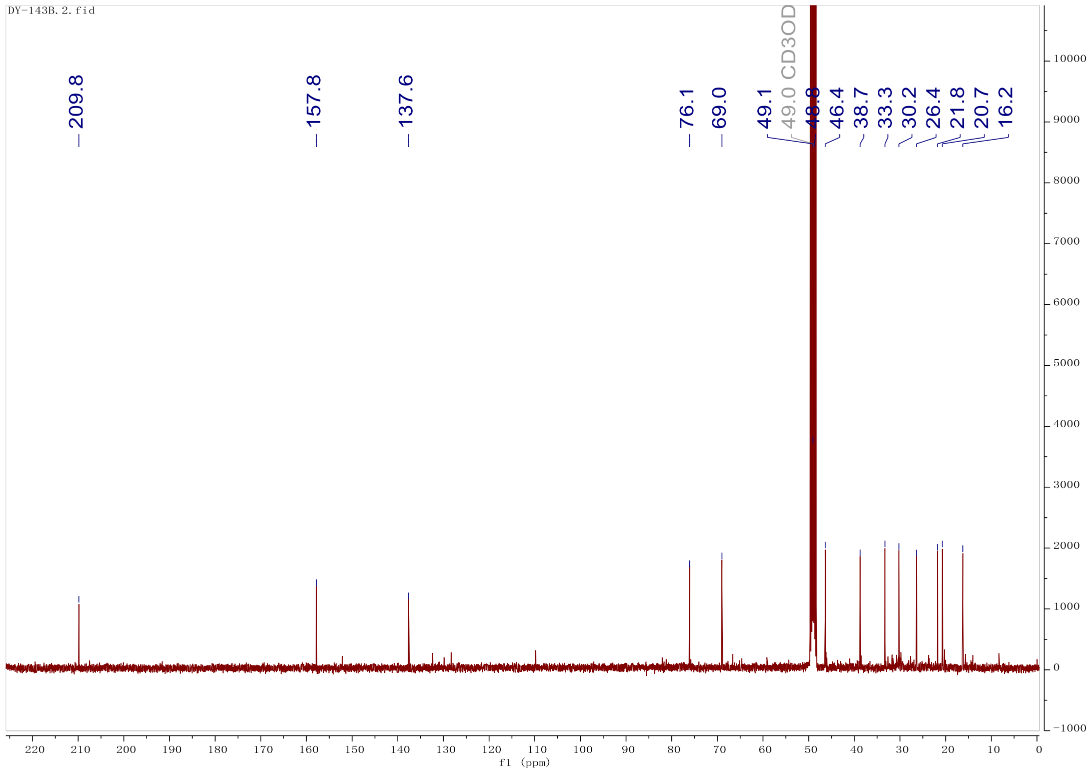

Figure S5-66. The ^{13}C NMR spectrum of compound **8** in CD_3OD (150 MHz)

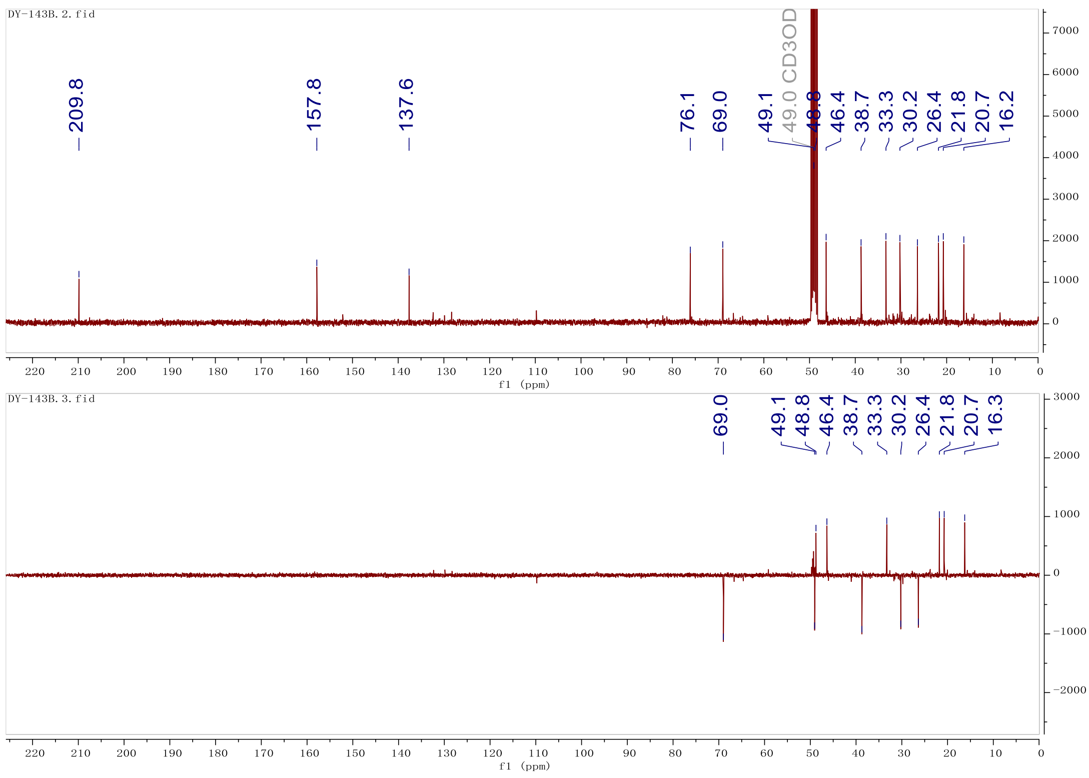

Figure S5-67. The DEPT 135 spectrum of compound **8** in CD_3OD (150 MHz)

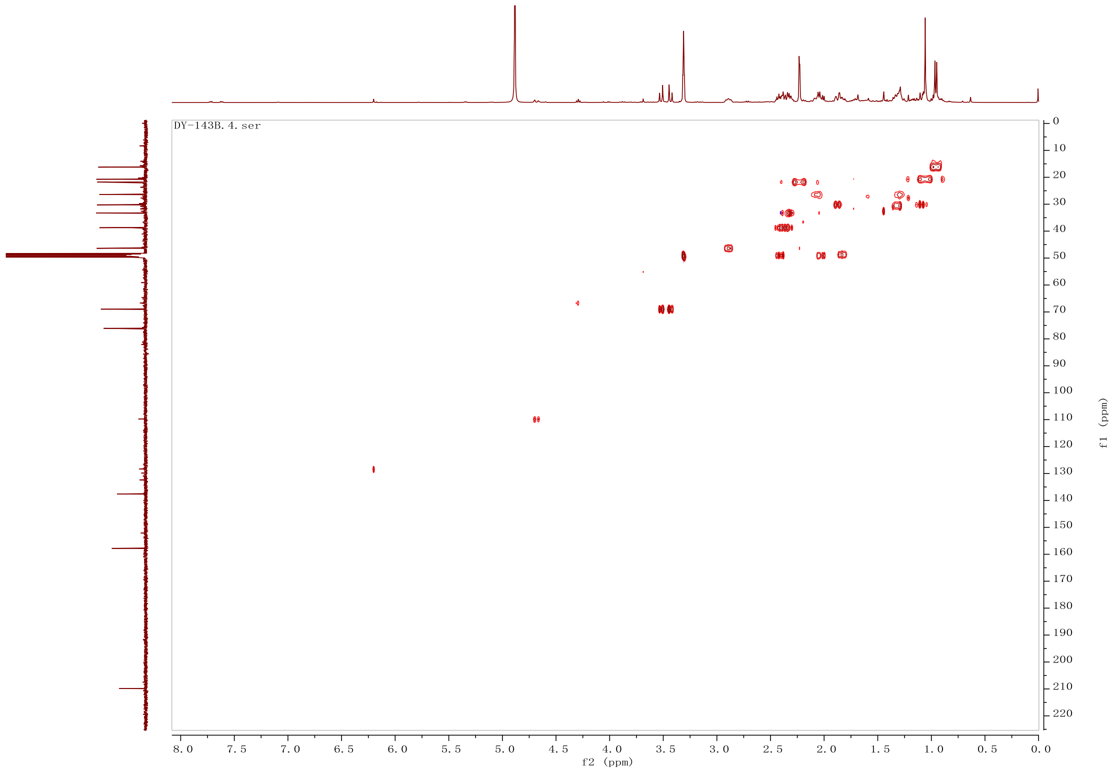

Figure S5-68. The HSQC spectrum of compound **8** in CD_3OD (600 MHz)

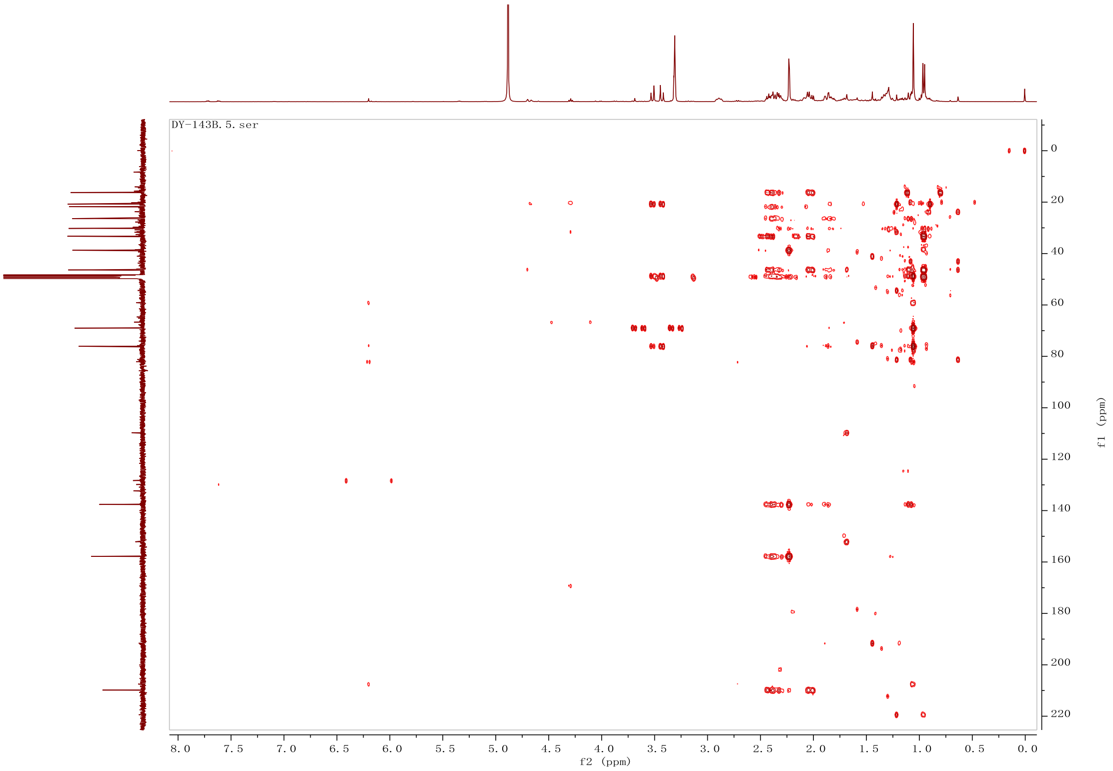

Figure S5-69. The HMBC spectrum of compound **8** in CD_3OD (600 MHz)

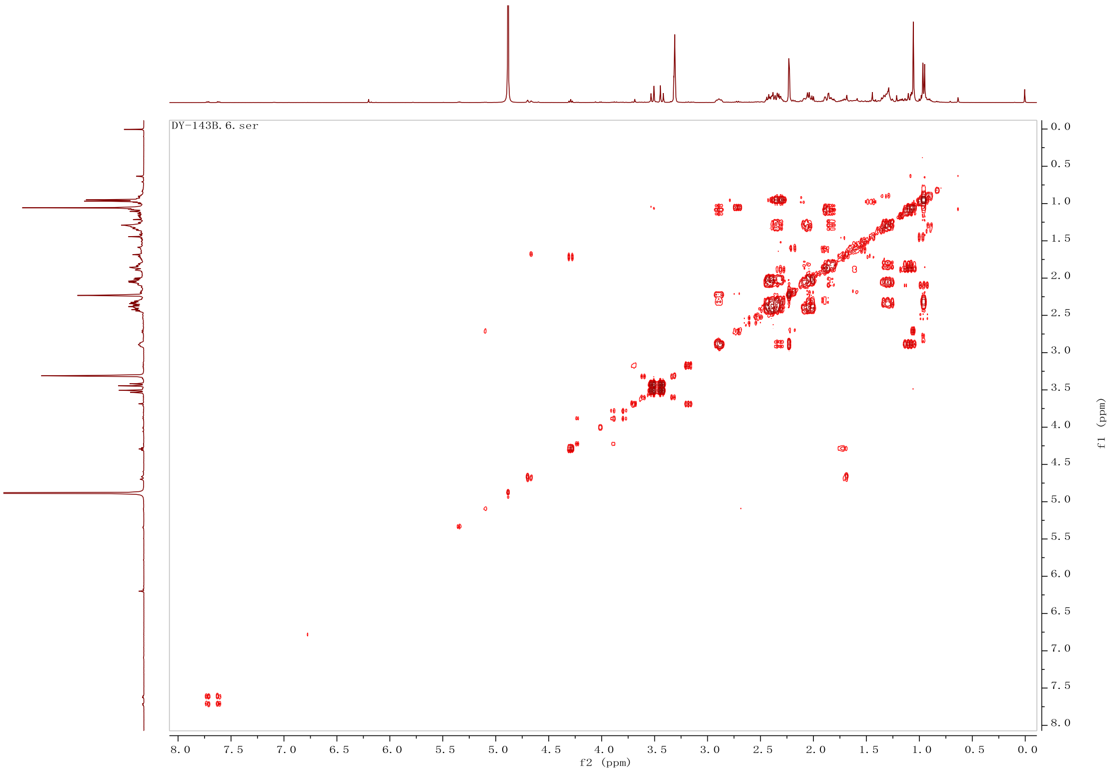

Figure S5-70. The ^1H-^1H COSY spectrum of compound **8** in CD_3OD (600 MHz)

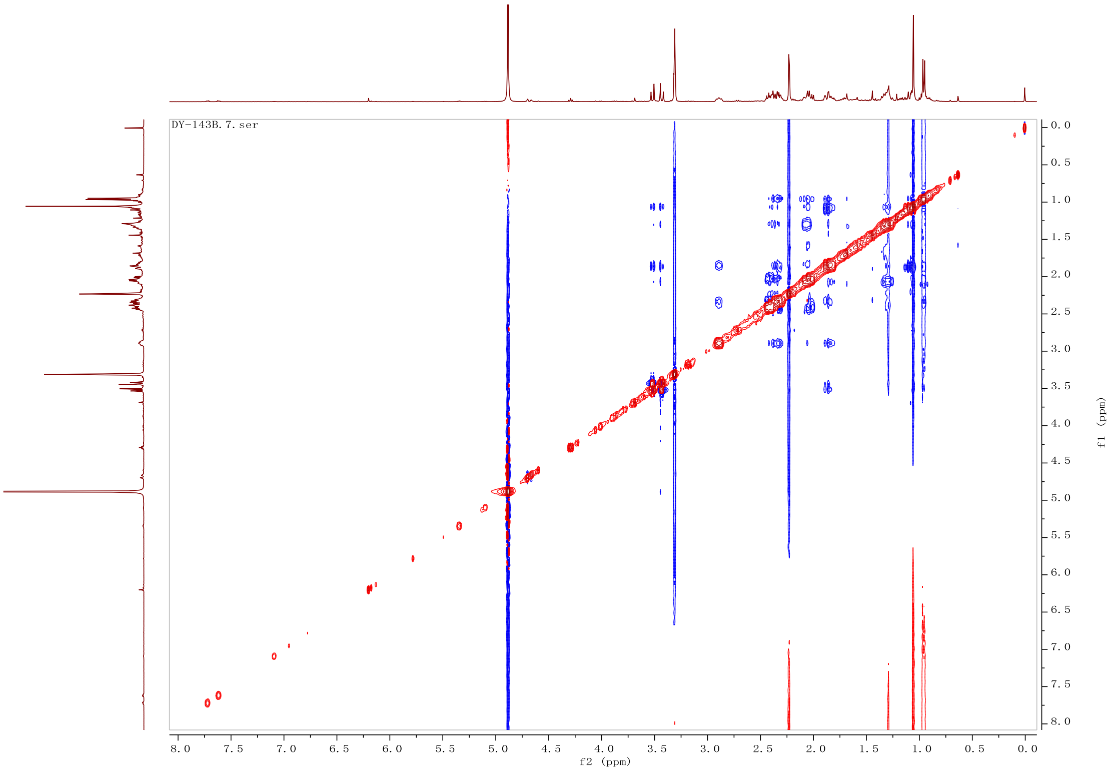

Figure S5-71. The NOESY spectrum of compound **8** in CD_3OD (600 MHz)

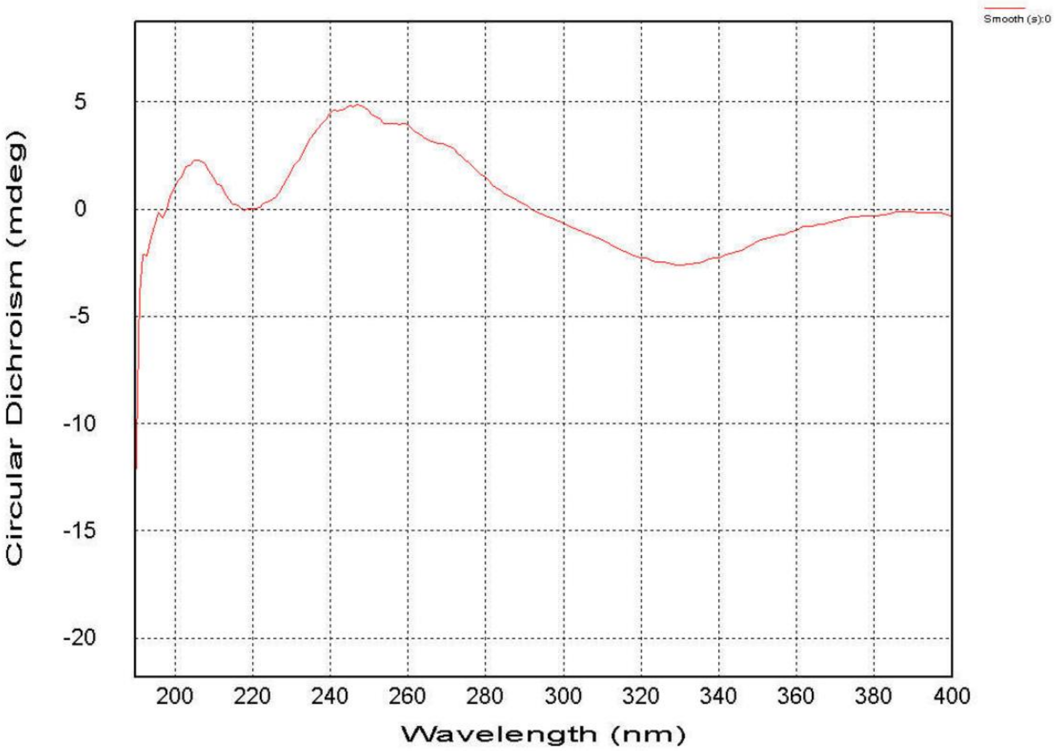

Figure S5-72. The ECD spectrum of compound **8** in MeOH

Qualitative Analysis Report

**Data Filename**	ESIH202306274.d	**Sample Name**	E0-DY-174A
**Sample ID**		**Position**	P1-A1
**Instrument Name**	Agilent 6520 Q-TOF	**Acq Method**	20160322_MS_ESIH_POS_1min.m
**Acquired Time**	11/3/2023 9:40:57	**IRM Calibration Status**	Success
**DA Method**	small molecular data analysis method.m	**Comment**	ESIH by fangsu

User Spectra

**Fragmentor Voltage**	**Collision Energy**	**Ionization Mode**
170	0	ESI

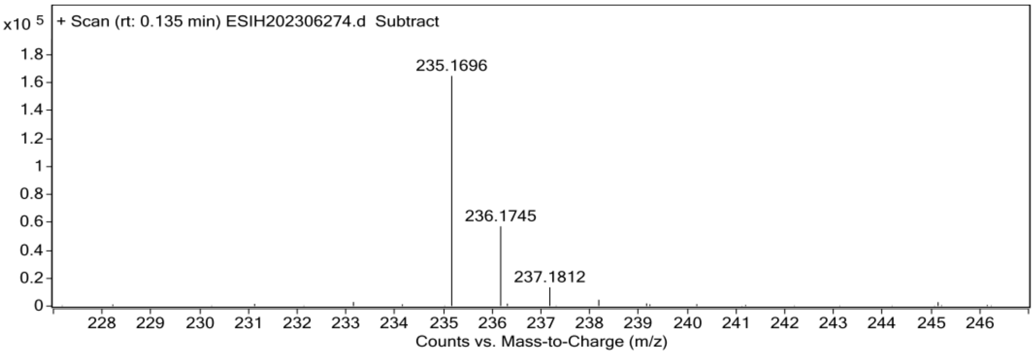

Formula Calculator Results

m/z	Calc m/z	Diff (mDa)	Diff (ppm)	Ion Formula	Ion
235.1696	235.1693	-0.34	-1.45	C15 H23 O2	(M+H)+

--- End Of Report ---

Figure S5-73. The HRESIMS spectrum of compound **9**

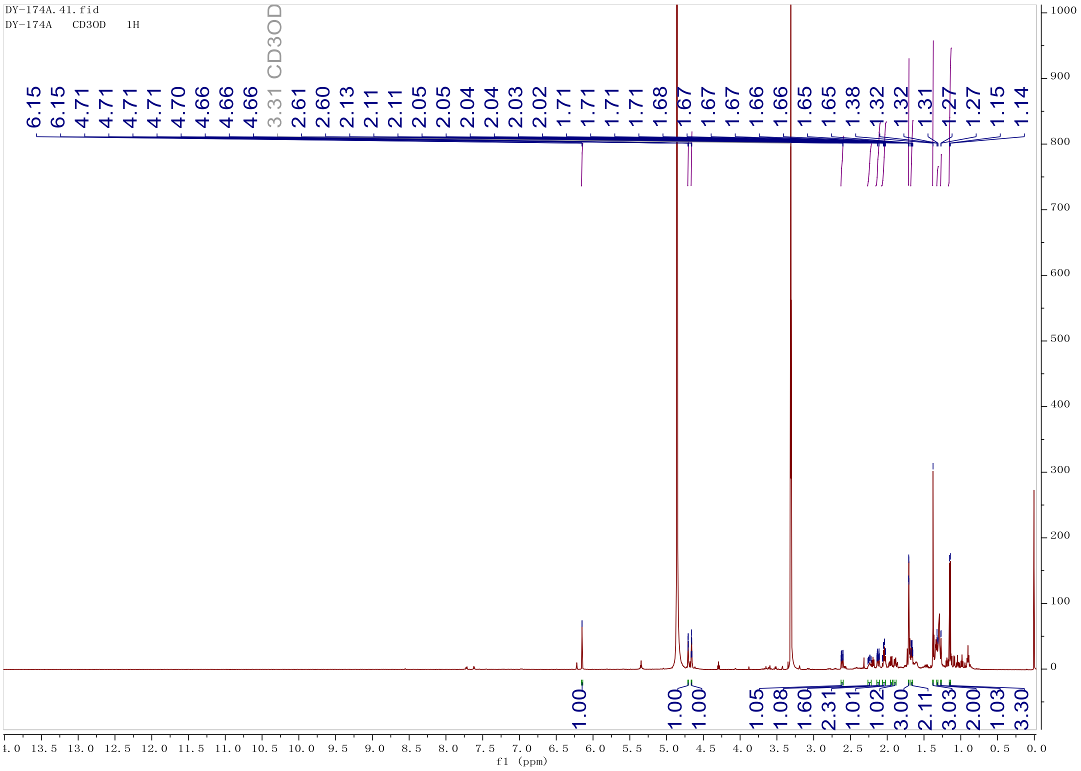

Figure S5-74. The ^{1}H NMR spectrum of compound **9** in CD_3OD (600 MHz)

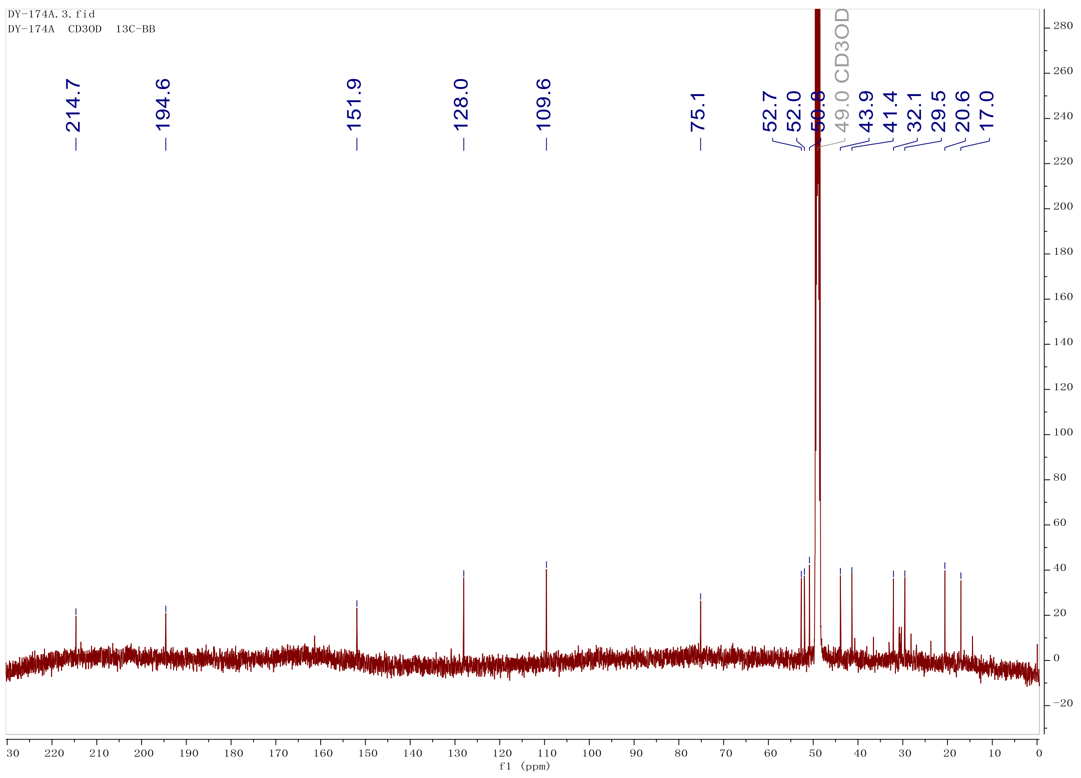

Figure S5-75. The ^{13}C NMR spectrum of compound **9** in CD_3OD (150 MHz)

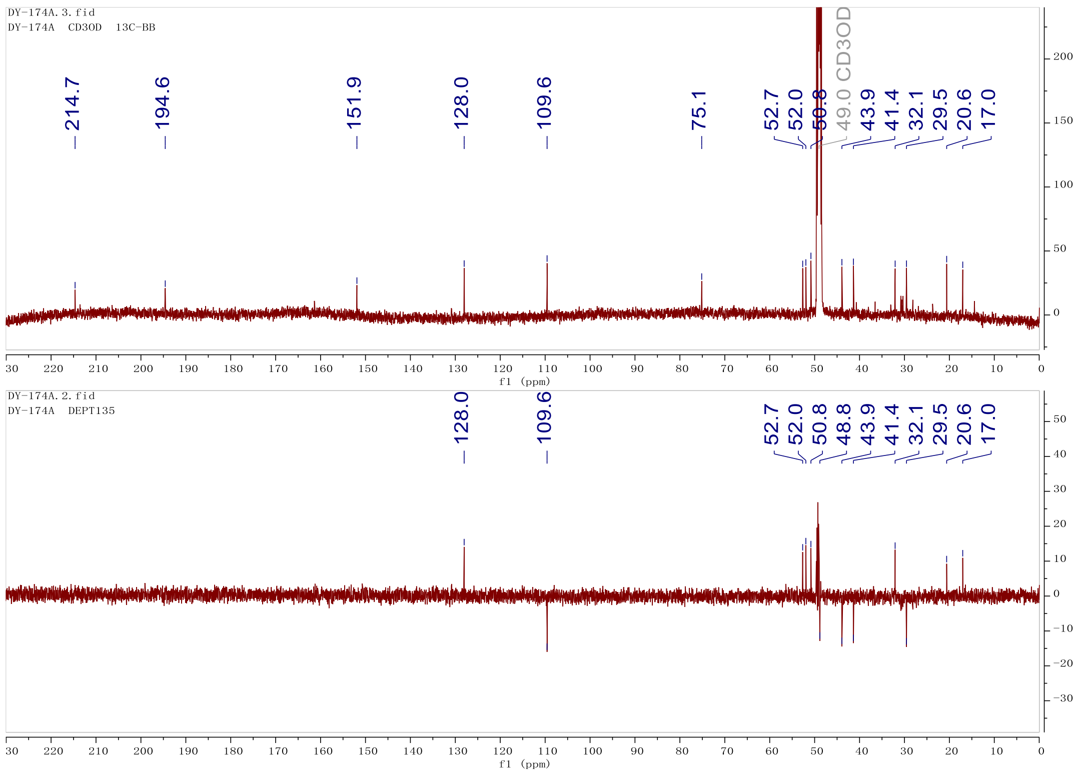

Figure S5-76. The DEPT 135 spectrum of compound **9** in CD_3OD (150 MHz)

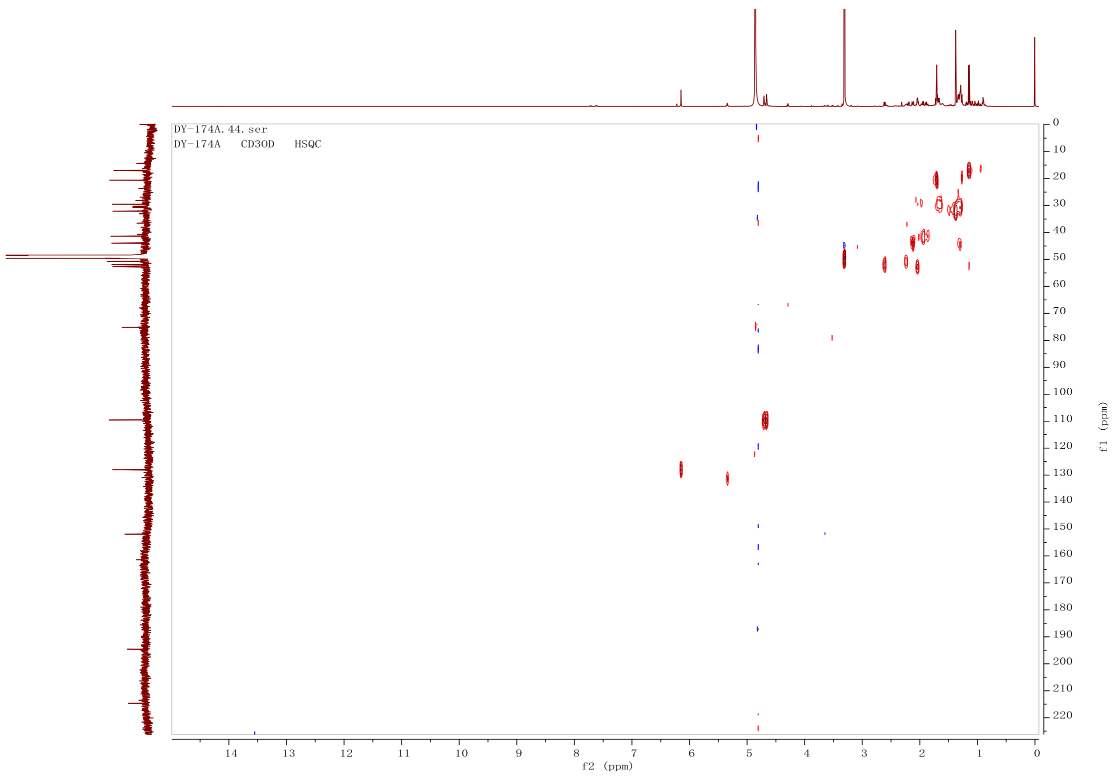

Figure S5-77. The HSQC spectrum of compound **9** in CD_3OD (600 MHz)

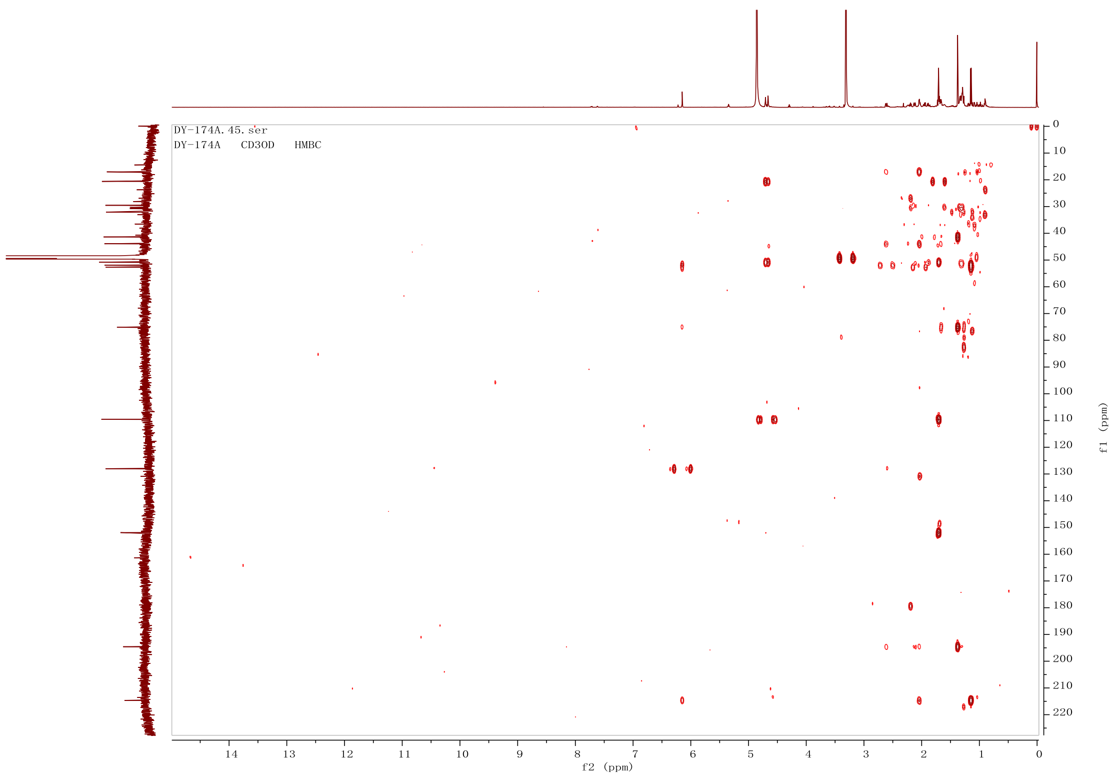

Figure S5-78. The HMBC spectrum of compound **9** in CD_3OD (600 MHz)

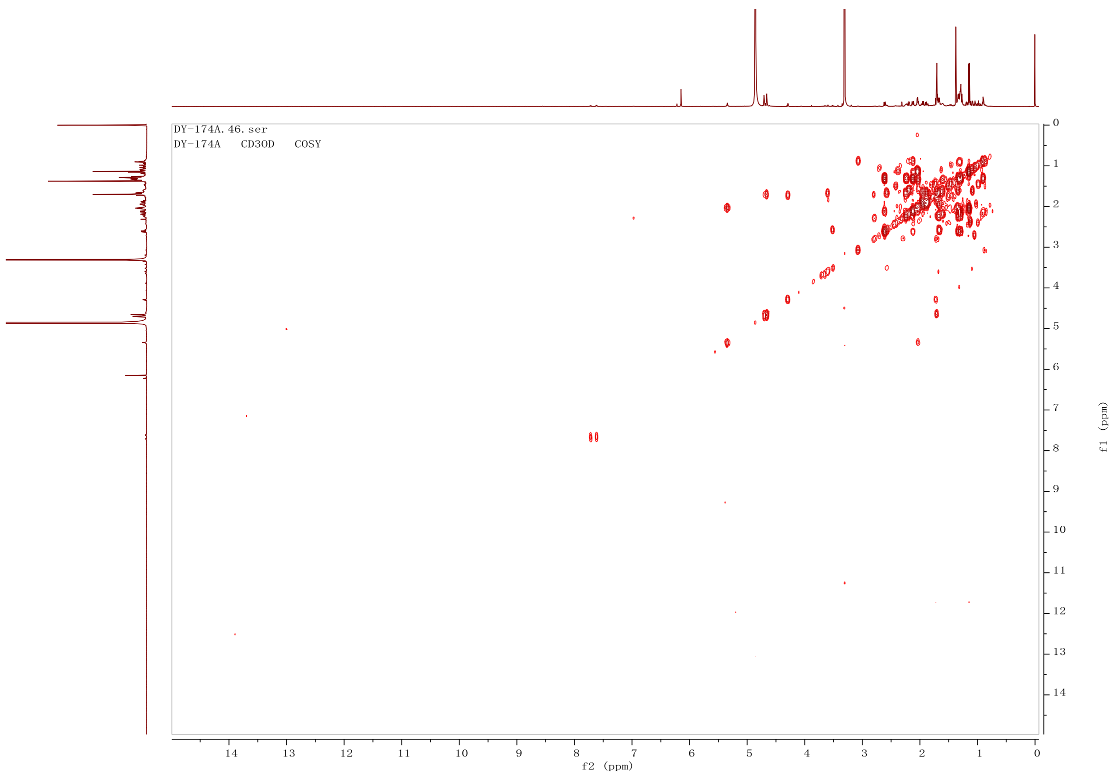

Figure S5-79. The ^{1}H-^{1}H COSY spectrum of compound **9** in CD_3OD (600 MHz)

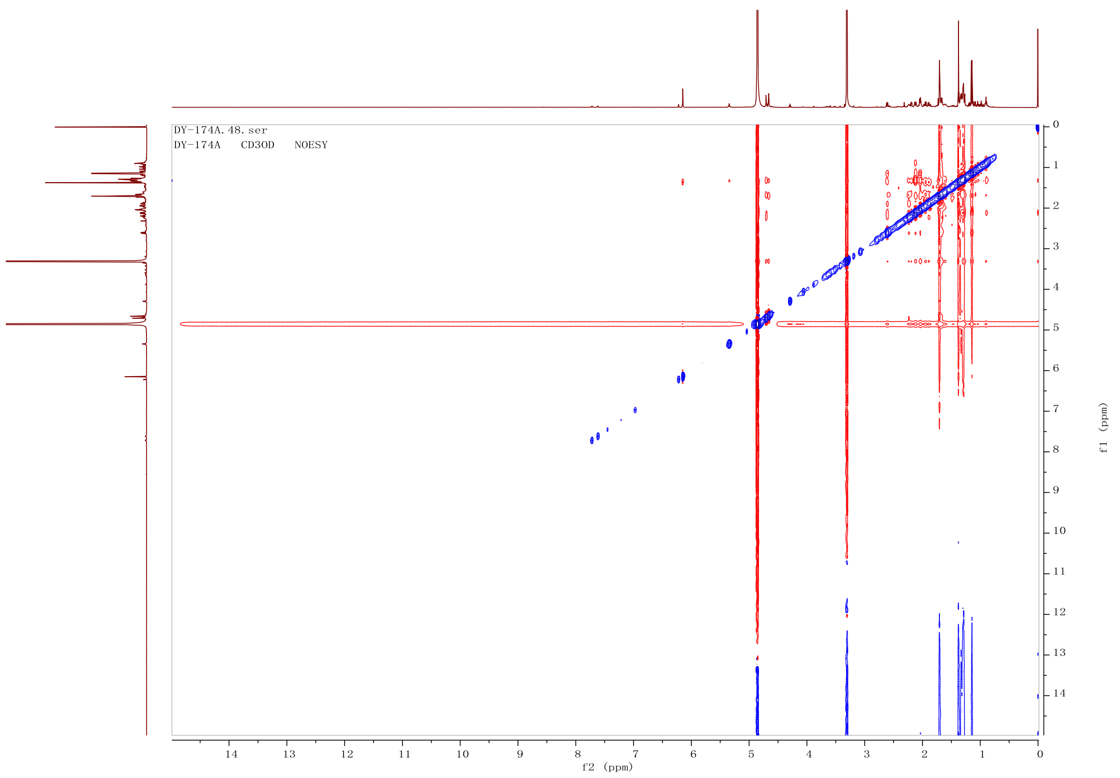

Figure S5-80. The NOESY spectrum of compound **9** in CD_3OD (600 MHz)

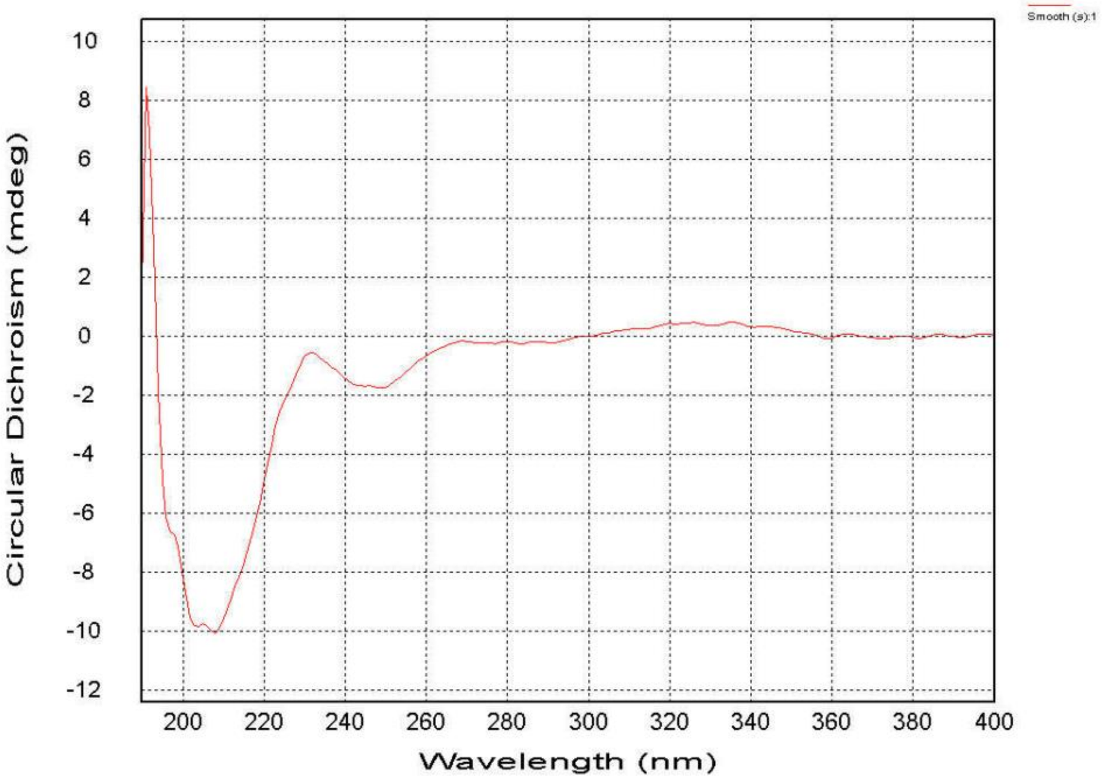

Figure S5-81. The ECD spectrum of compound **9** in MeOH

Qualitative Analysis Report

**Data Filename**	ESIH202306275.d	**Sample Name**	E0-DY-175A
**Sample ID**		**Position**	P1-A2
**Instrument Name**	Agilent 6520 Q-TOF	**Acq Method**	20160322_MS_ESIH_POS_1min.m
**Acquired Time**	11/3/2023 9:42:29	**IRM Calibration Status**	Success
**DA Method**	small molecular data analysis method.m	**Comment**	ESIH by fangsu

User Spectra

Fragmentor Voltage	Collision Energy	Ionization Mode
170	0	ESI

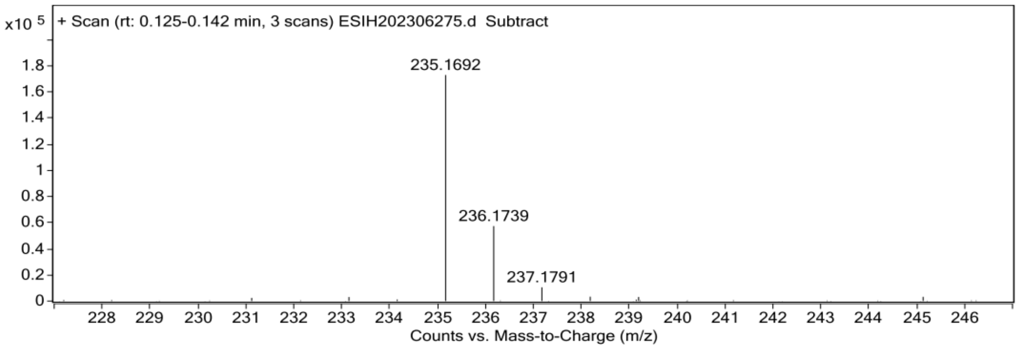

Formula Calculator Results

m/z	Calc m/z	Diff (mDa)	Diff (ppm)	Ion Formula	Ion
235.1692	235.1693	0.08	0.33	C15 H23 O2	(M+H)+

--- End Of Report ---

Figure S5-82. The HRESIMS spectrum of compound **10**

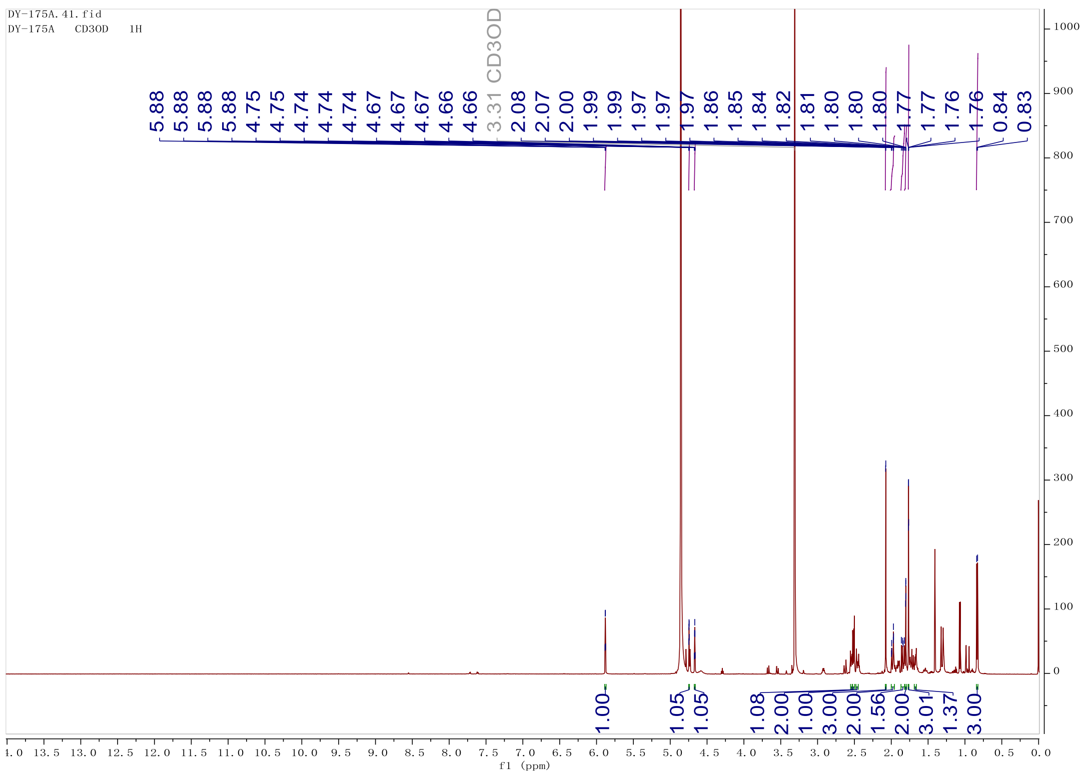

Figure S5-83. The ^{1}H NMR spectrum of compound **10** in CD_3OD (600 MHz)

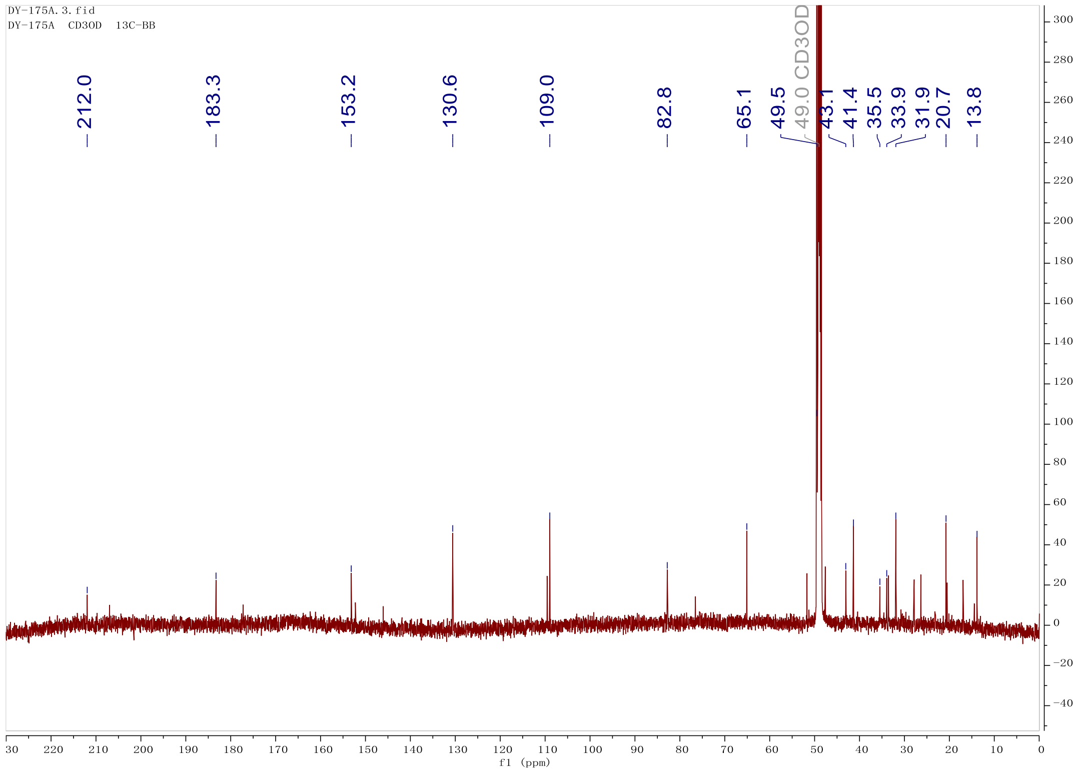

Figure S5-84. The ^{13}C NMR spectrum of compound **10** in CD_3OD (150 MHz)

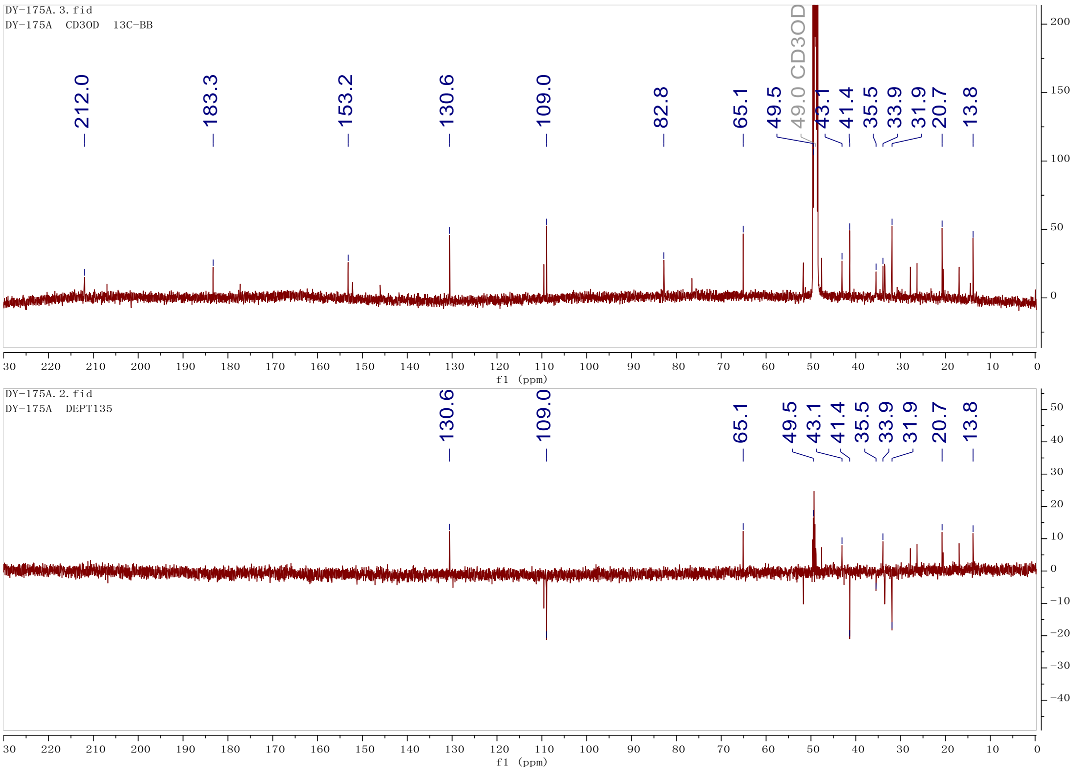

Figure S5-85. The DEPT 135 spectrum of compound **10** in CD_3OD (150 MHz)

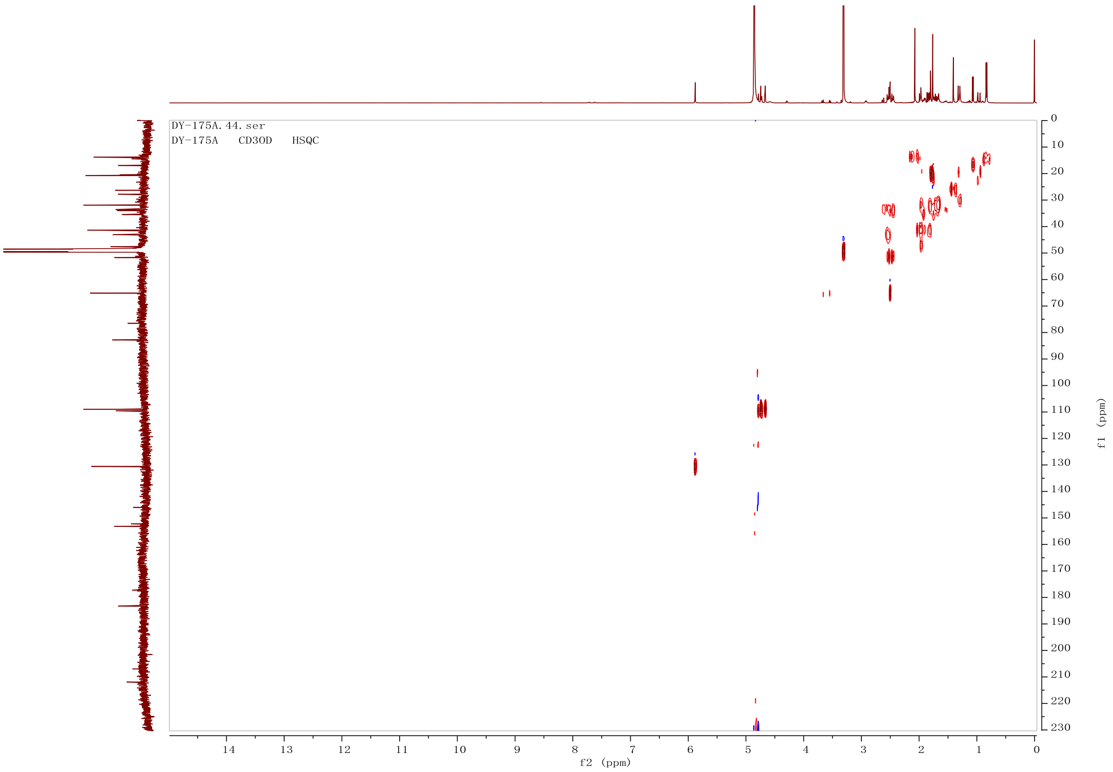

Figure S5-86. The HSQC spectrum of compound **10** in CD_3OD (600 MHz)

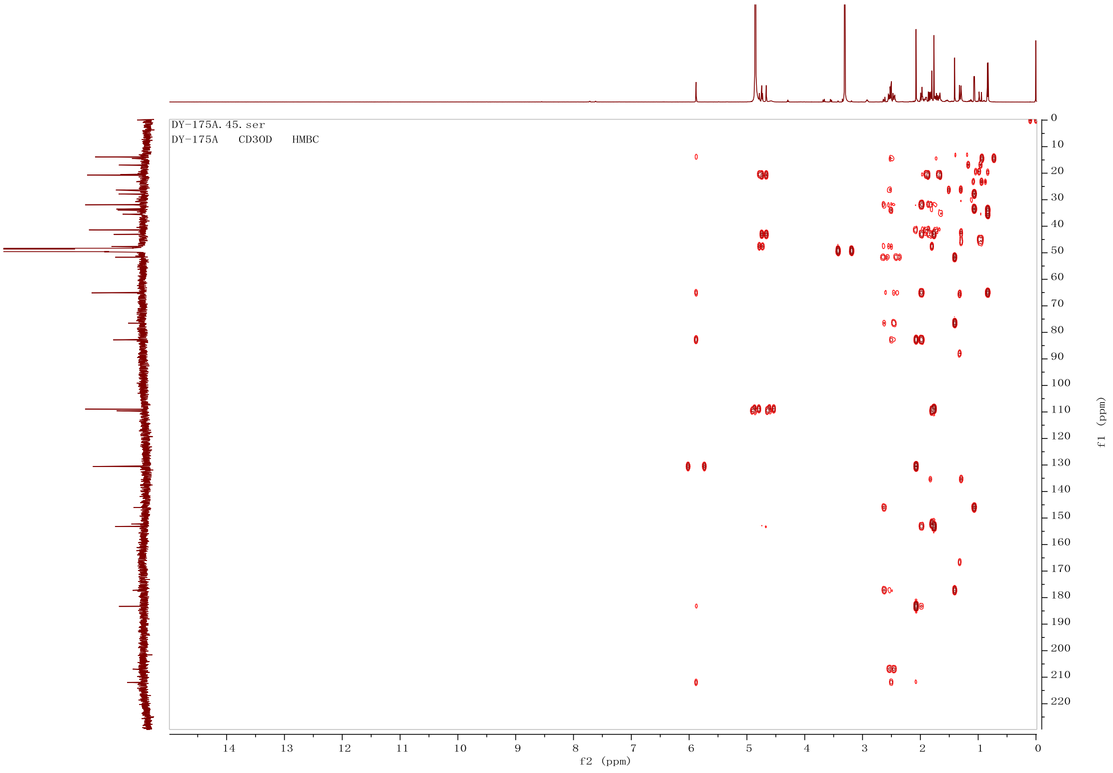

Figure S5-87. The HMBC spectrum of compound **10** in CD_3OD (600 MHz)

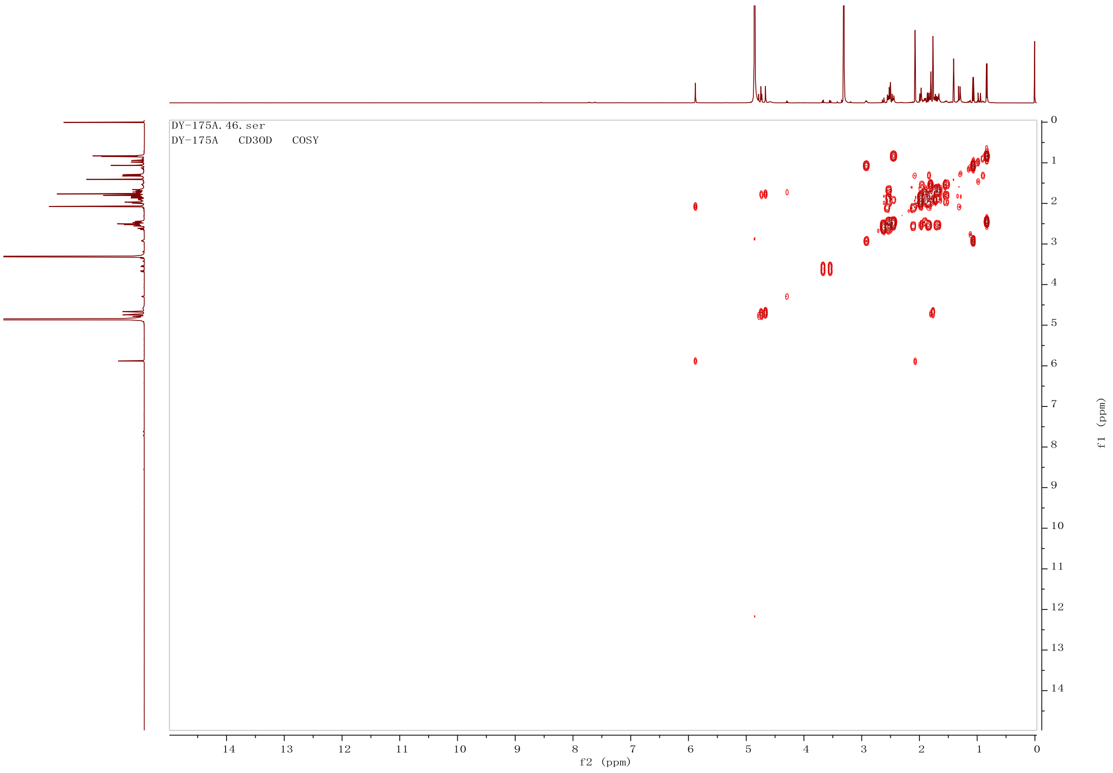

Figure S5-88. The ^{1}H-^{1}H COSY spectrum of compound **10** in CD_3OD (600 MHz)

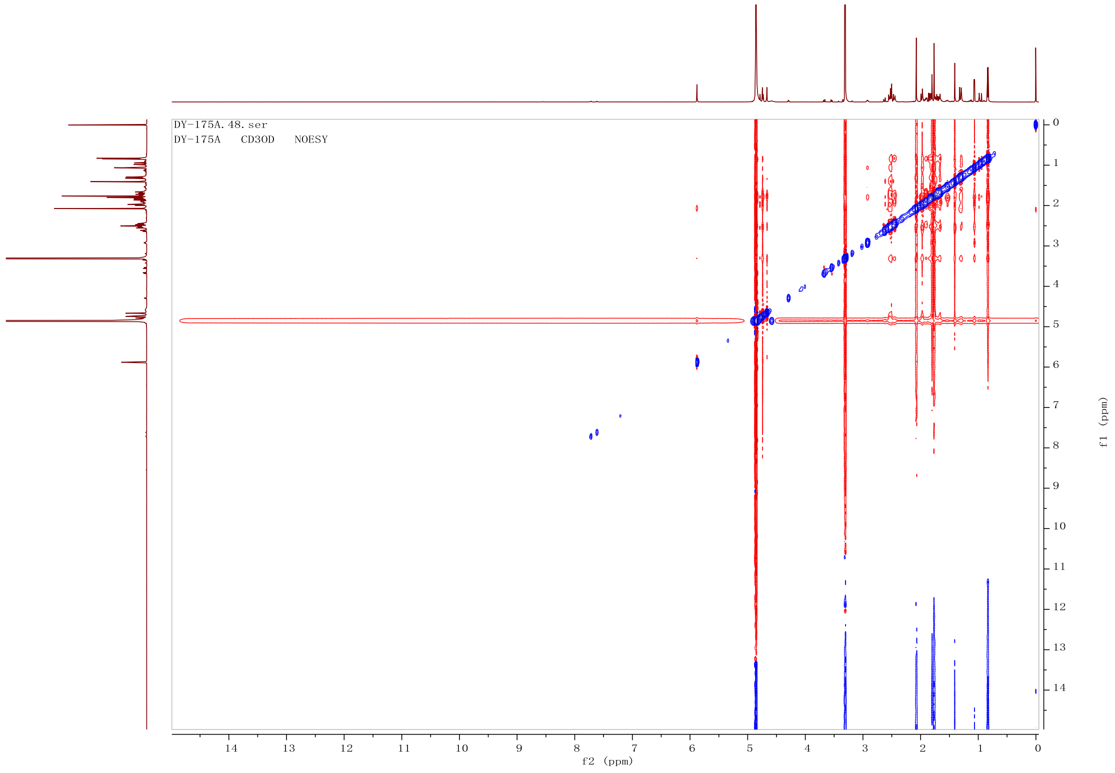

Figure S5-89. The NOESY spectrum of compound **10** in CD_3OD (600 MHz)

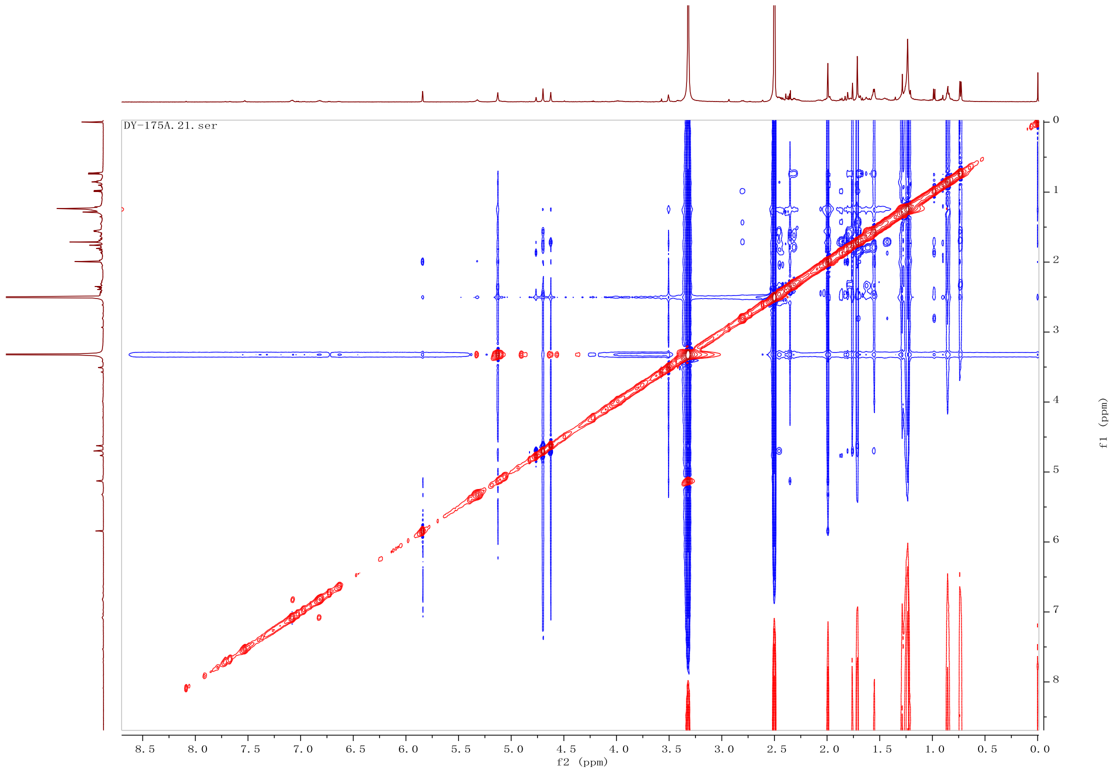

Figure S5-90. The NOESY spectrum of compound **10** in DMSO-d_6 (600 MHz)

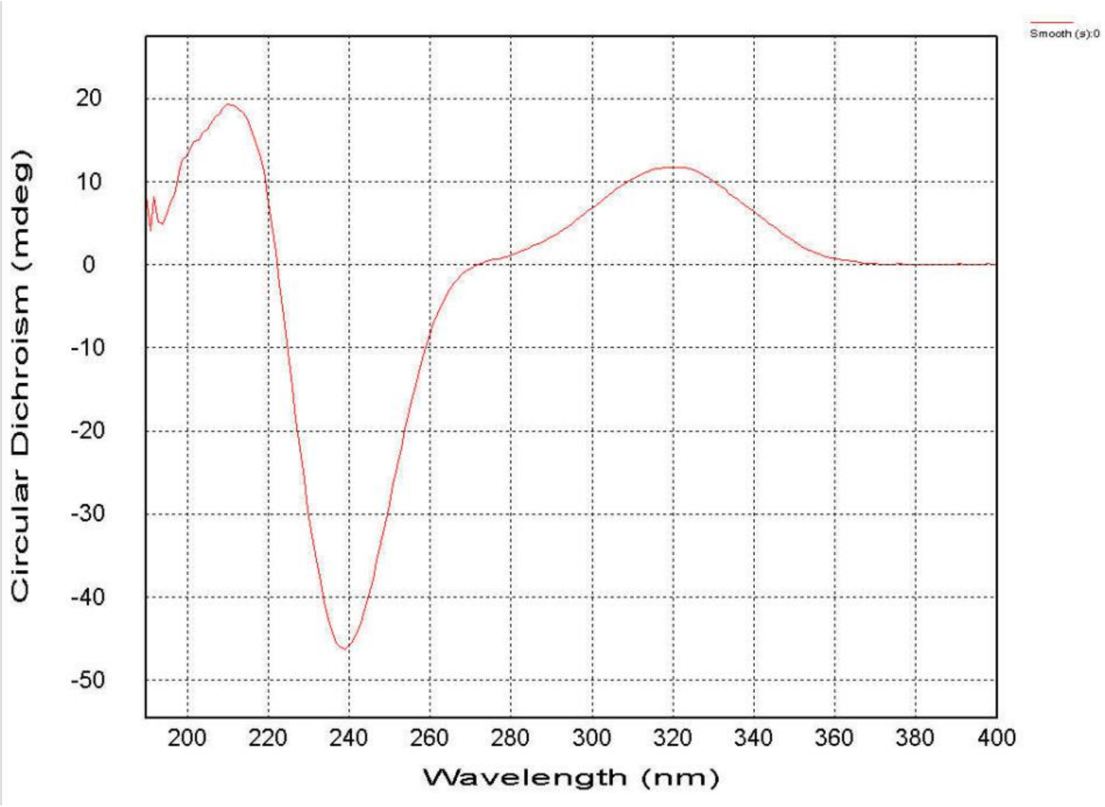

Figure S5-91. The ECD spectrum of compound **10** in MeOH

Qualitative Analysis Report

**Data Filename**	ESIH202400468.d	**Sample Name**	E0-DY-202A
**Sample ID**		**Position**	P1-A1
**Instrument Name**	Agilent 6520 Q-TOF	**Acq Method**	20160322_MS_ESIH_POS_1min.m
**Acquired Time**	1/26/2024 4:32:42 PM	**DA Method**	ESI-HR-20231114.m
**Comment**	ESIH by fangsu		

User Spectra

Fragmentor Voltage	Collision Energy	Ionization Mode
170	0	ESI

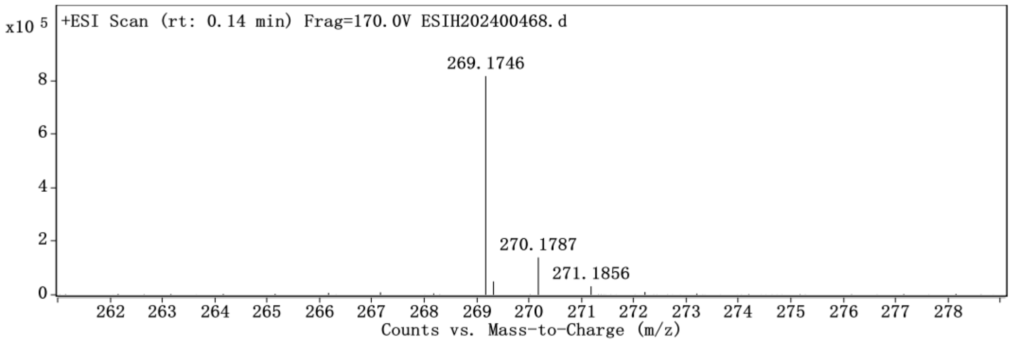

Formula Calculator Results

m/z	Calc m/z	Diff (mDa)	Diff (ppm)	Ion Formula	Ion
269.1746	269.1747	0.13	0.48	C15 H25 O4	(M+H)+

--- End Of Report ---

Figure S5-92. The HRESIMS spectrum of compound **11**

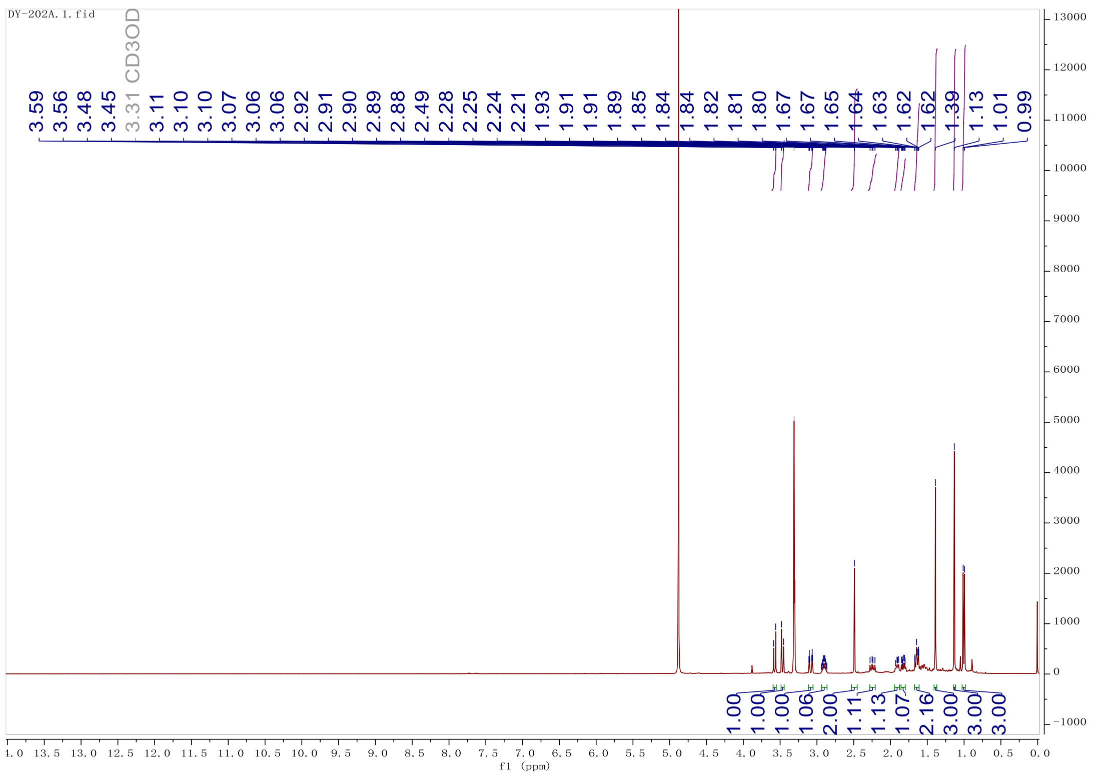

Figure S5-93. The ^{1}H NMR spectrum of compound **11** in CD_3OD (600 MHz)

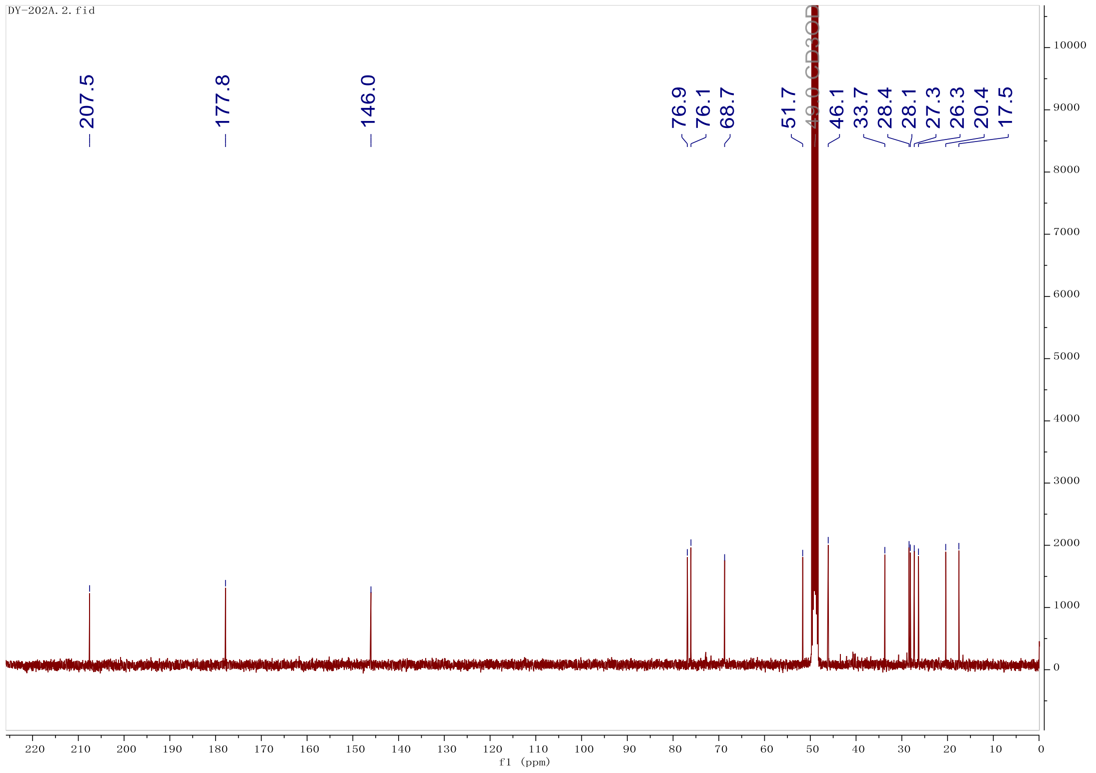

Figure S5-94. The ^{13}C NMR spectrum of compound **11** in CD_3OD (150 MHz)

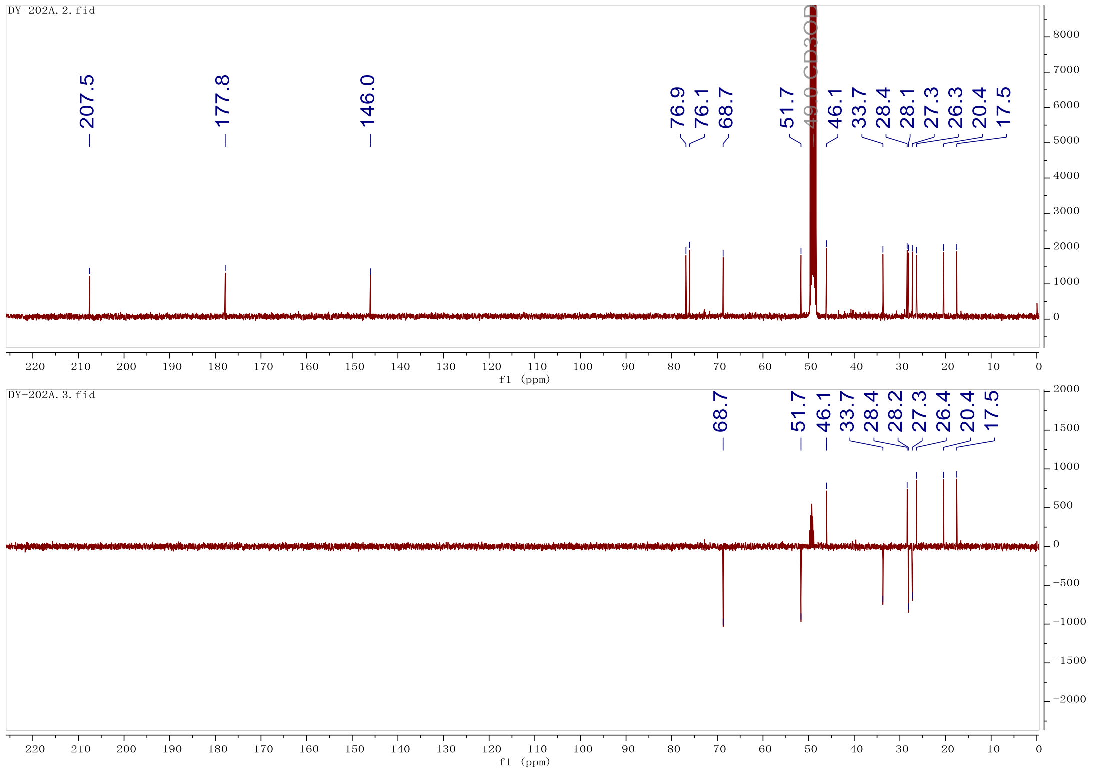

Figure S5-95. The DEPT 135 spectrum of compound **11** in CD_3OD (150 MHz)

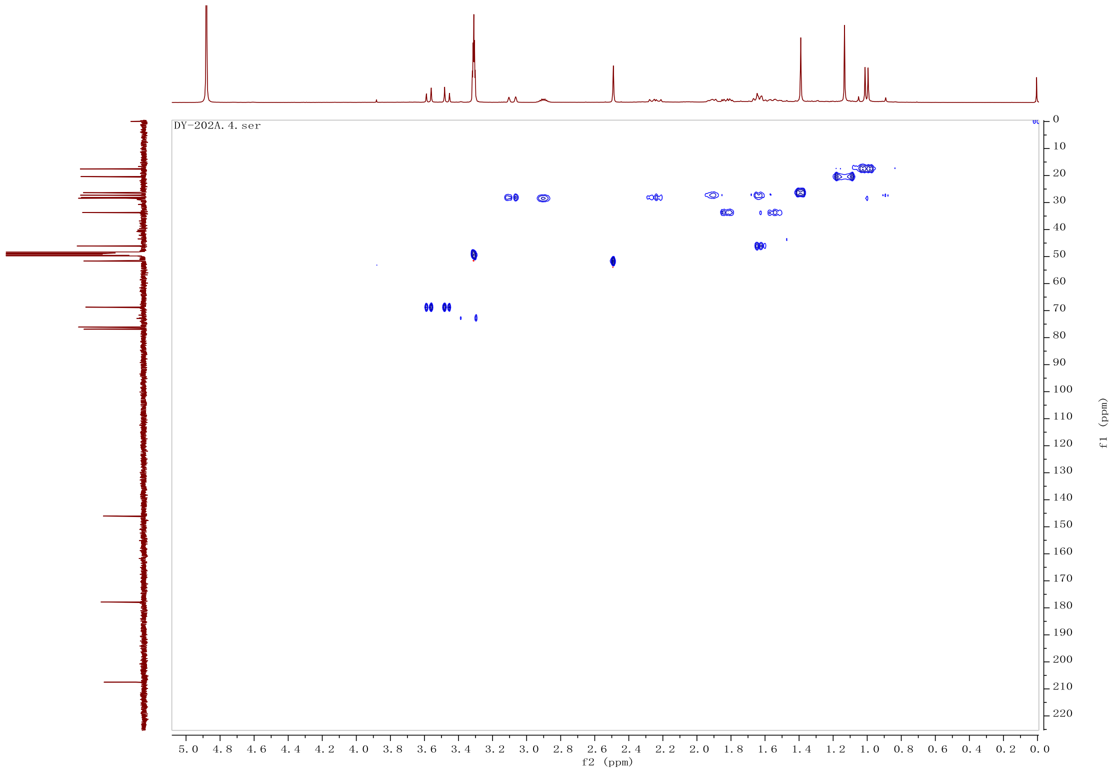

Figure S5-96. The HSQC spectrum of compound **11** in CD_3OD (600 MHz)

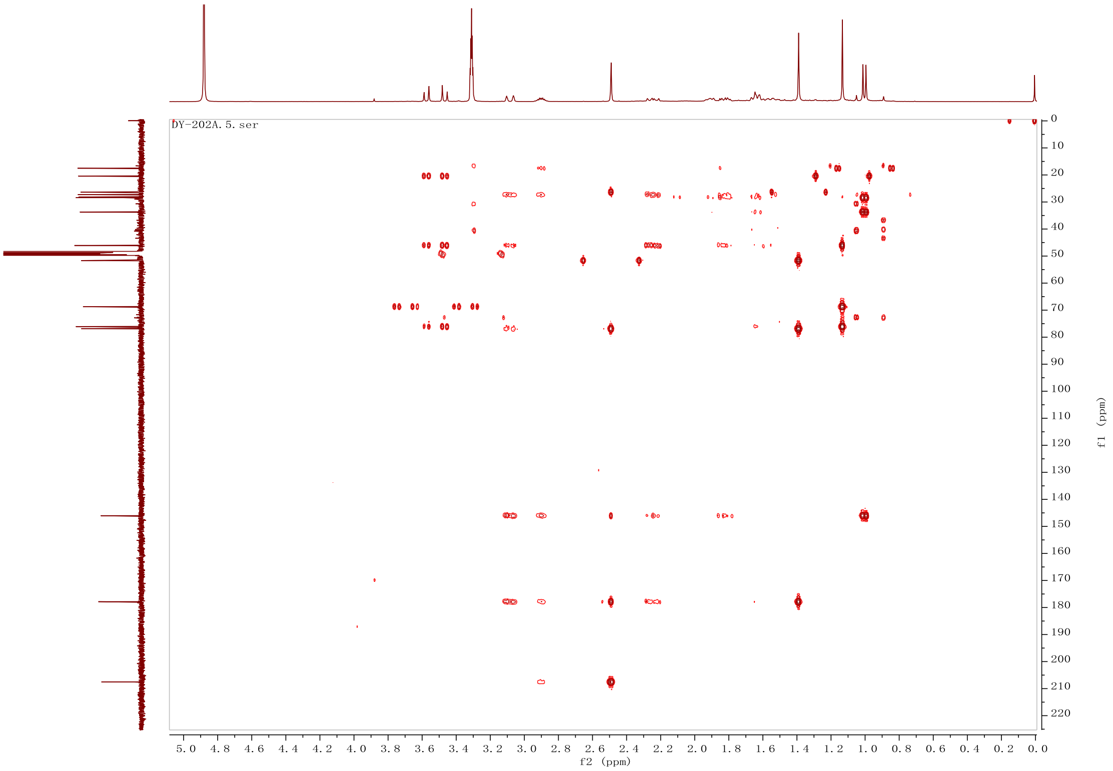

Figure S5-97. The HMBC spectrum of compound **11** in CD_3OD (600 MHz)

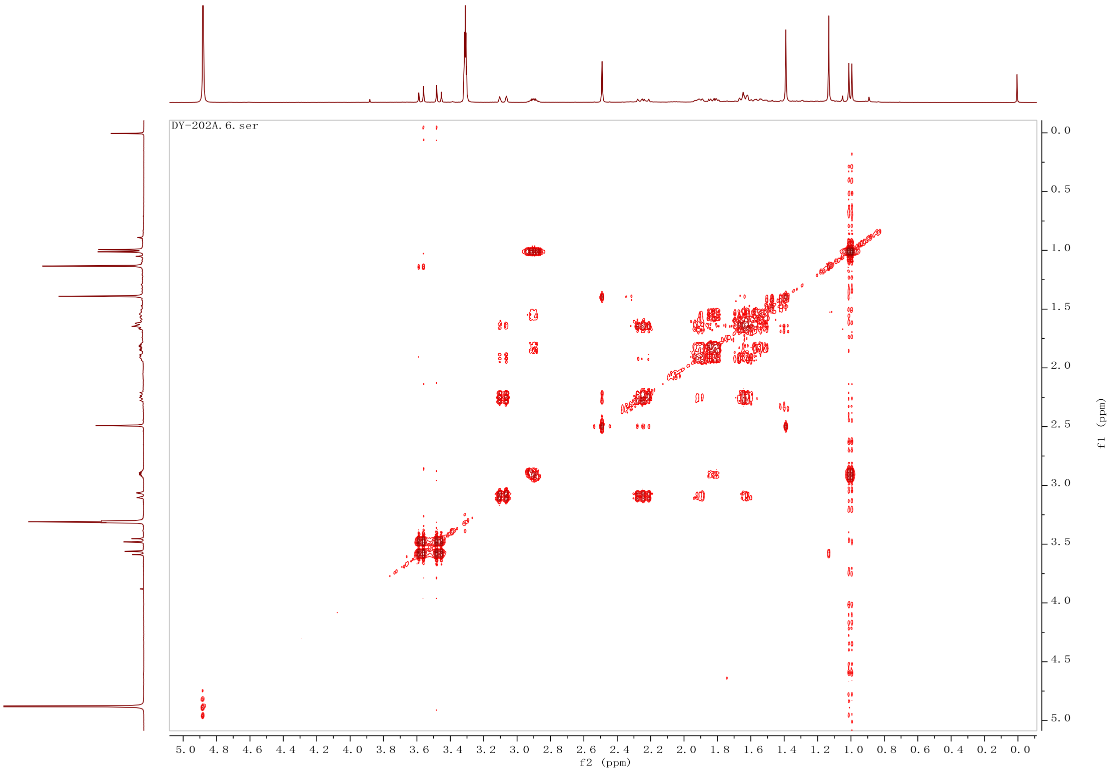

Figure S5-98. The ^1H-^1H COSY spectrum of compound **11** in CD_3OD (600 MHz)

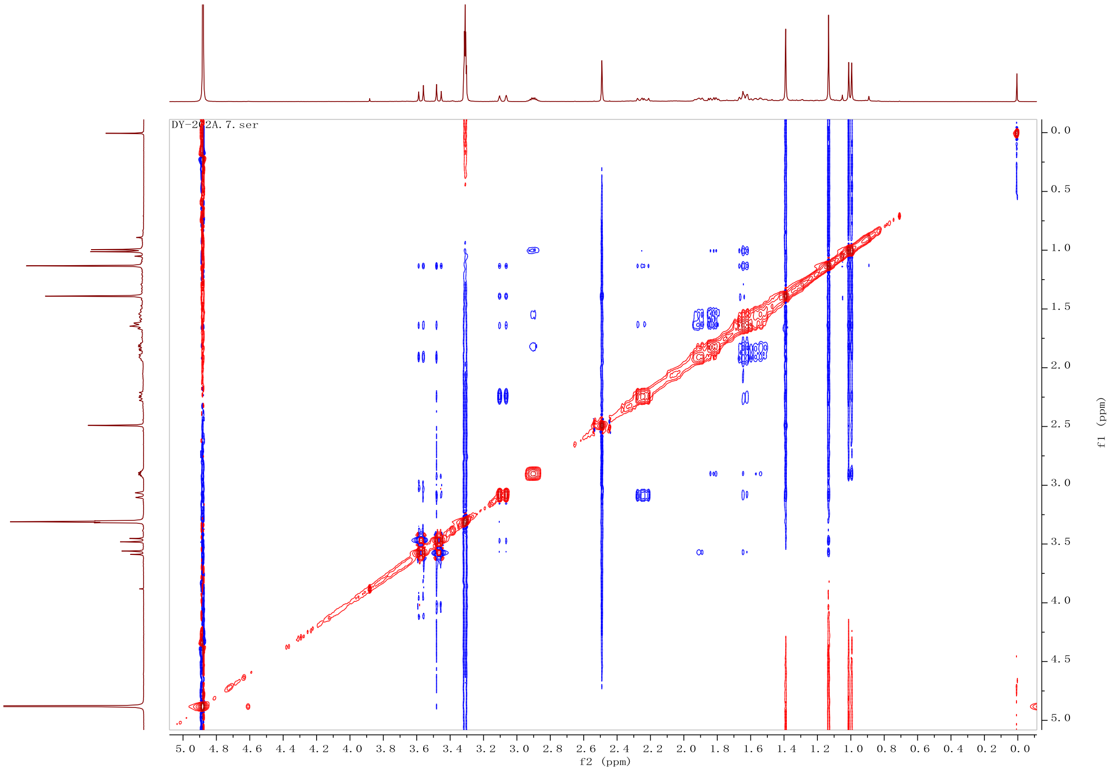

Figure S5-99. The NOESY spectrum of compound **11** in CD_3OD (600 MHz)

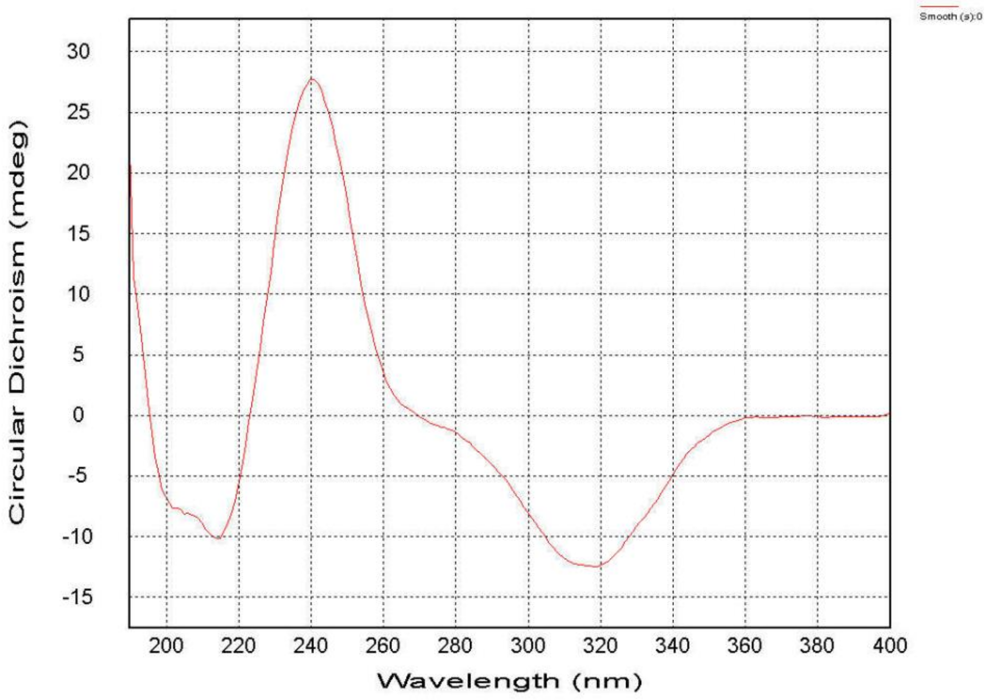

Figure S5-100. The ECD spectrum of compound **11** in MeOH

Isomer Nº			1	2
DP4+ (%)		H data	99.29%	0.71%
		C data	0.00%	100.00%
		All data	0.10%	99.90%
Type	sp2?	Exp	1	2
C	x	146	35.19098657	35.7099333
C	x	207.5	-30.8625874	-24.558331
C		51.7	131.7516108	133.299102
C		76.9	105.4951448	106.736238
C	x	177.8	-7.58680596	-1.854625
C		28.2	159.4317123	160.472175
C		46.1	140.3720437	139.977465
C		27.3	160.811837	160.549973
C		33.7	156.9721571	156.198223
C		28.4	154.574568	154.439103
C		76.1	107.7041474	108.787591
C		68.7	117.7733263	117.621999
C		20.4	165.863285	165.148886
C		26.4	161.2299607	159.956204
C		17.5	169.6931994	169.08603
H		2.49	29.05071906	29.1985848
H		2.49	29.02984557	29.2627012
H		3.08	28.92242194	29.4644231
H		2.24	29.24836284	28.9040865
H		1.63	29.65481544	29.8592205
H		1.91	30.00471146	30.2150961
H		1.63	29.73412889	29.886043
H		1.82	29.68546083	29.8791653
H		1.54	30.02791749	30.0755341
H		2.9	29.03725989	29.0843012
H		3.57	27.95549515	28.0520797
H		3.47	28.21946524	28.3000376
H		1.13	30.73619339	30.864023
H		1.13	30.49366635	30.5515688
H		1.13	30.39279862	30.3339119
H		1.39	30.18330172	30.2167882
H		1.39	30.11108122	30.2320712
H		1.39	30.70235577	30.7977098
H		1	30.74903221	30.7458243
H		1	30.48363497	30.8468316
H		1	30.6475701	30.4317529

Default parameters	1	2
sDP4+ (H data)	99.90%	0.10%
sDP4+ (C data)	4.13%	95.87%
sDP4+ (all data)	97.75%	2.25%
uDP4+ (H data)	12.12%	87.88%
uDP4+ (C data)	0.02%	99.98%
uDP4+ (all data)	0.00%	100.00%
DP4+ (H data)	99.29%	0.71%
DP4+ (C data)	0.00%	100.00%
DP4+ (all data)	0.10%	99.90%

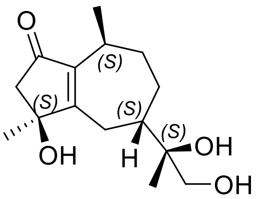

11S (1)

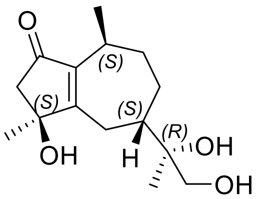

11R (2)

Figure S5-101. Calculated NMR data and DP4+ probability statistics of compound **11**

Qualitative Analysis Report

**Data Filename**	ESIH202304974.d	**Sample Name**	E0-DY-159
**Sample ID**		**Position**	P1-A8
**Instrument Name**	Agilent 6520 Q-TOF	**Acq Method**	20160322_MS_ESIH_POS_1min.m
**Acquired Time**	8/23/2023 14:26:03	**IRM Calibration Status**	Success
**DA Method**	small molecular data analysis method.m	**Comment**	ESIH by fangsu

User Spectra

**Fragmentor Voltage**	**Collision Energy**	**Ionization Mode**
170	0	ESI

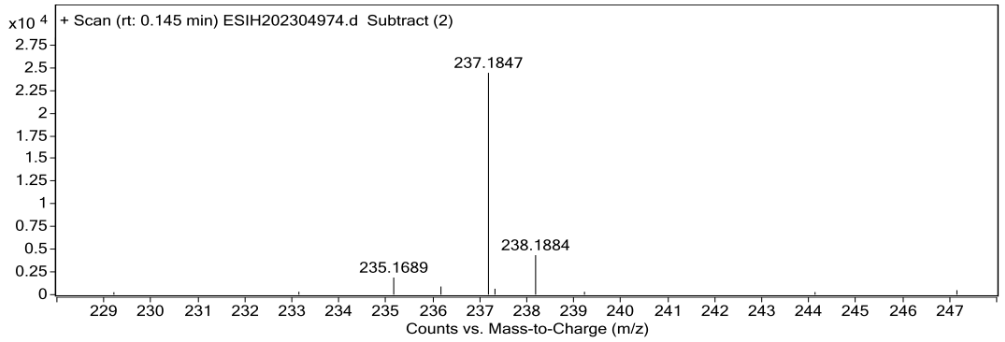

Formula Calculator Results

m/z	Calc m/z	Diff (mDa)	Diff (ppm)	Ion Formula	Ion
237.1847	237.1849	0.26	1.08	C15 H25 O2	(M+H)+

--- End Of Report ---

Figure S5-102. The HRESIMS spectrum of compound **12**

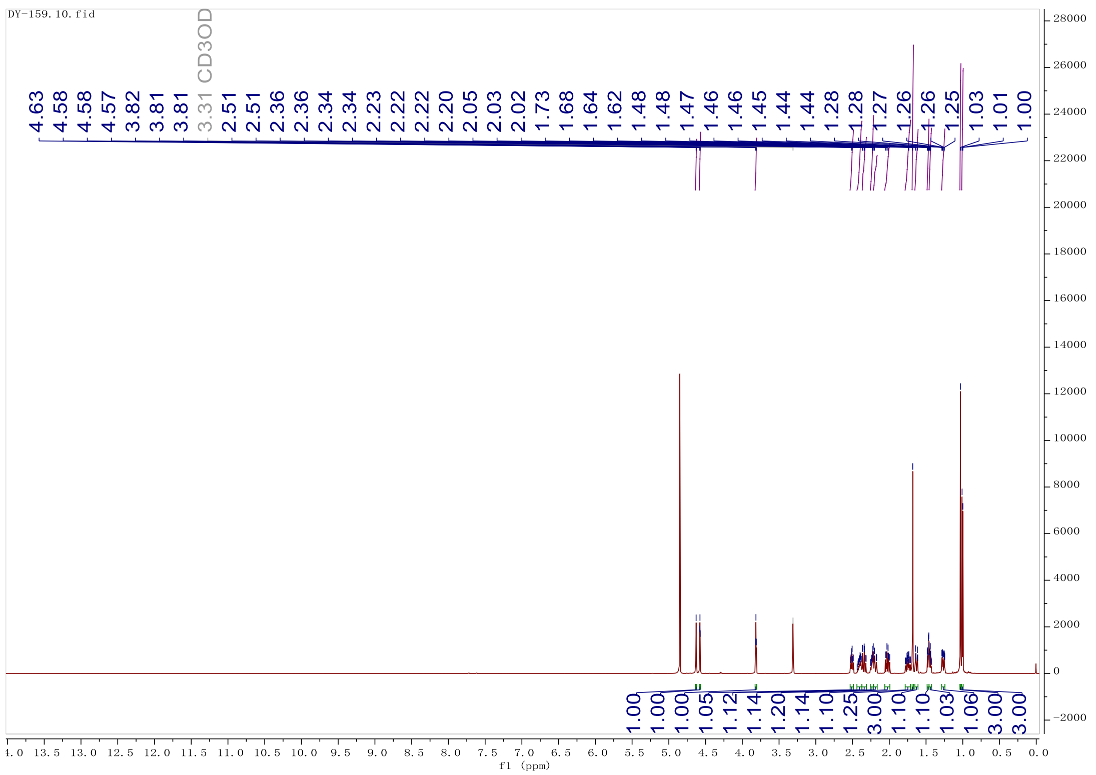

Figure S5-103. The ^{1}H NMR spectrum of compound **12** in CD_3OD (600 MHz)

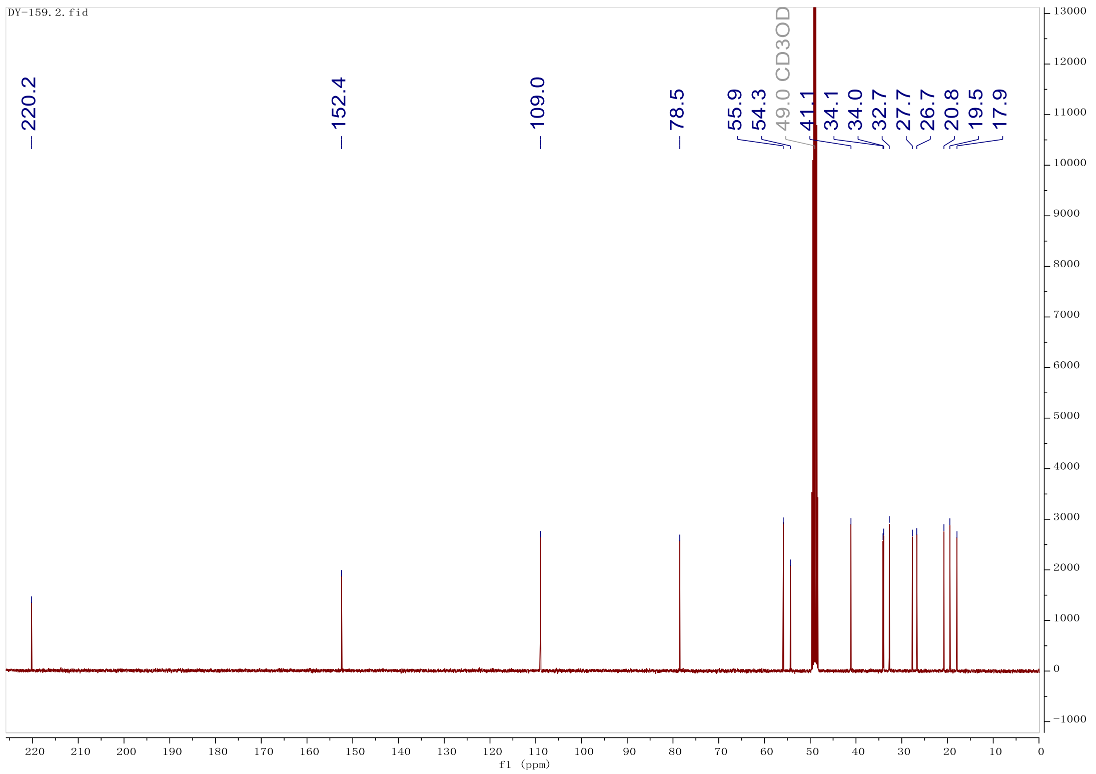

Figure S5-104. The ^{13}C NMR spectrum of compound **12** in CD_3OD (150 MHz)

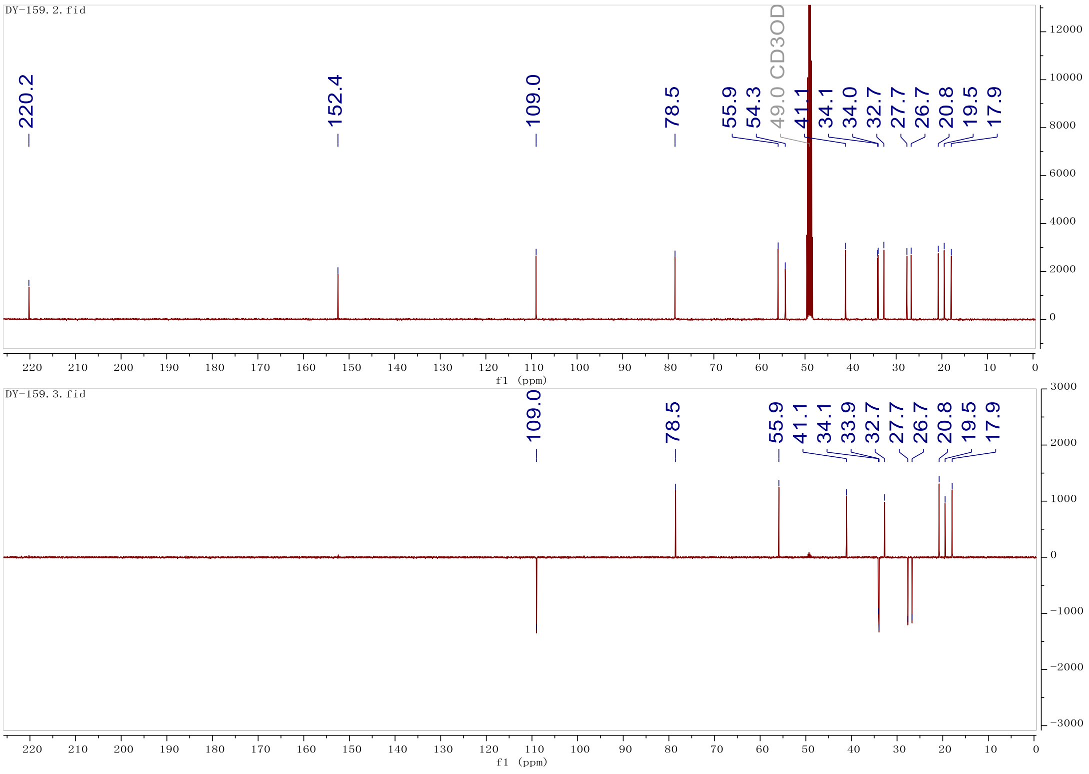

Figure S5-105. The DEPT 135 spectrum of compound **12** in CD_3OD (150 MHz)

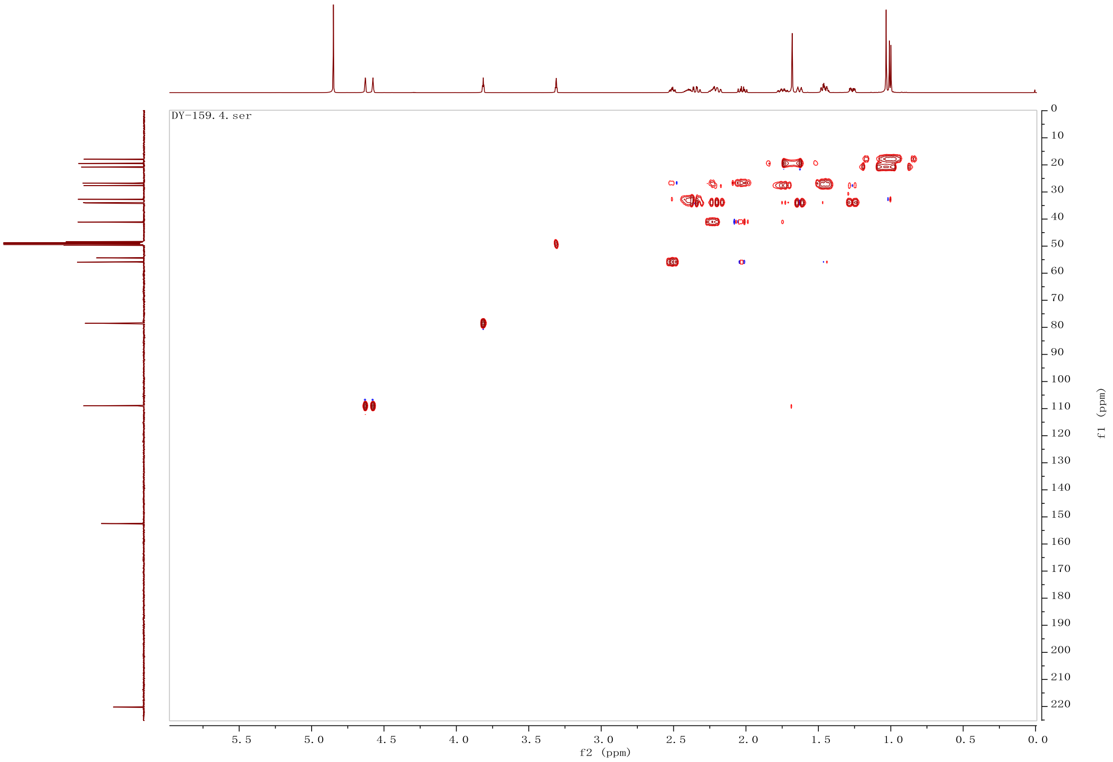

Figure S5-106. The HSQC spectrum of compound **12** in CD_3OD (600 MHz)

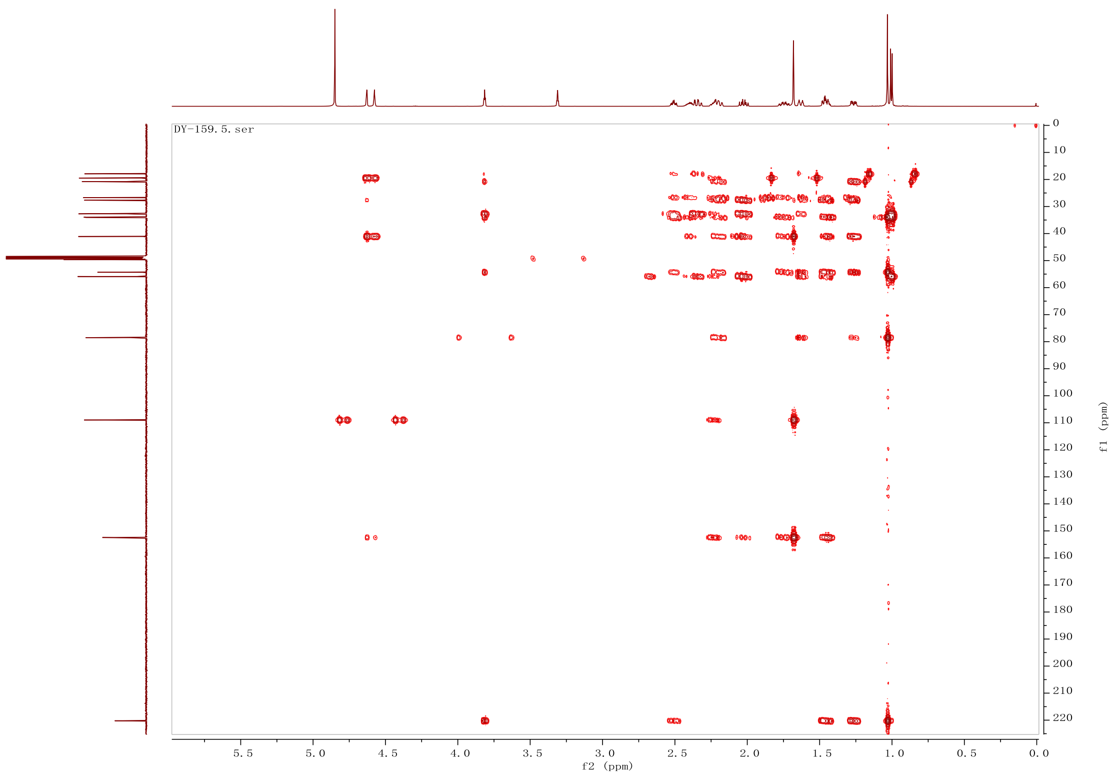

Figure S5-107. The HMBC spectrum of compound **12** in CD_3OD (600 MHz)

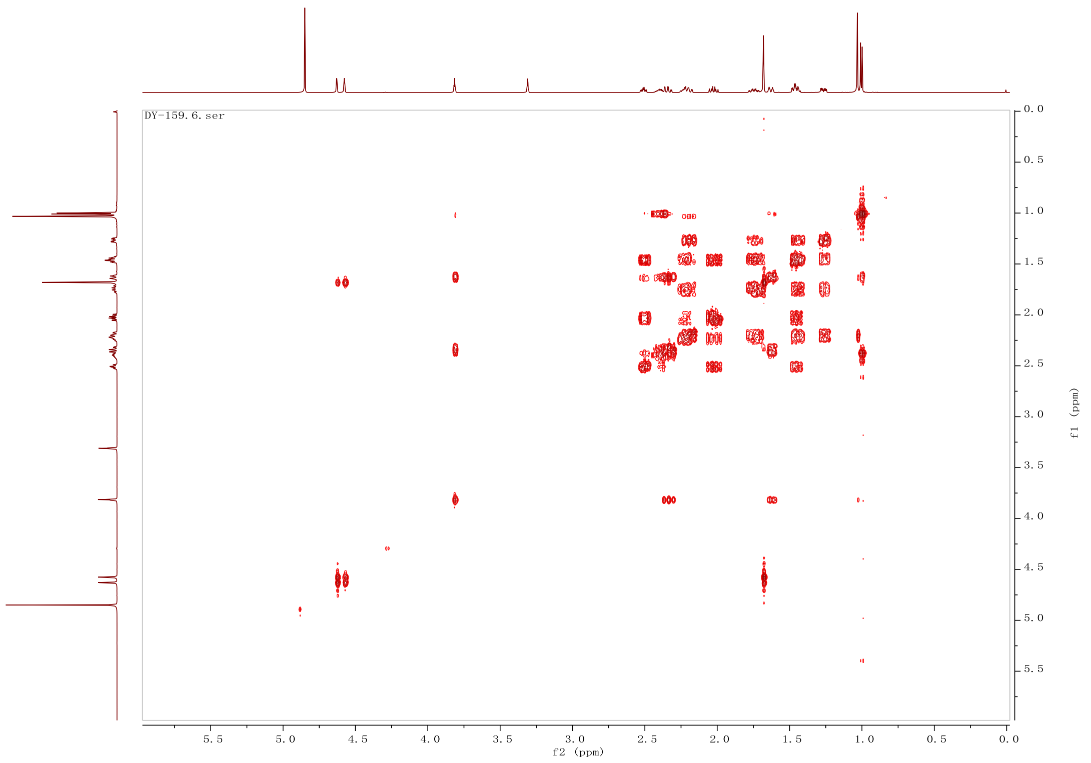

Figure S5-108. The ^{1}H-^{1}H COSY spectrum of compound **12** in CD_3OD (600 MHz)

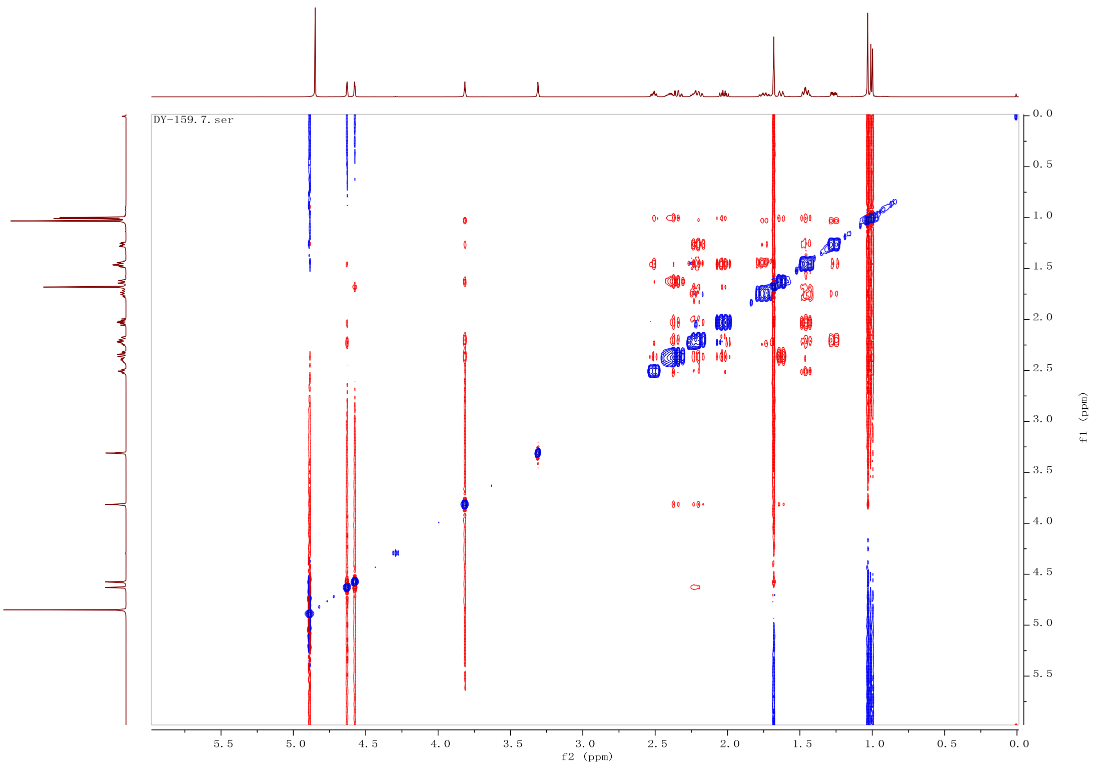

Figure S5-109. The NOESY spectrum of compound **12** in CD_3OD (600 MHz)

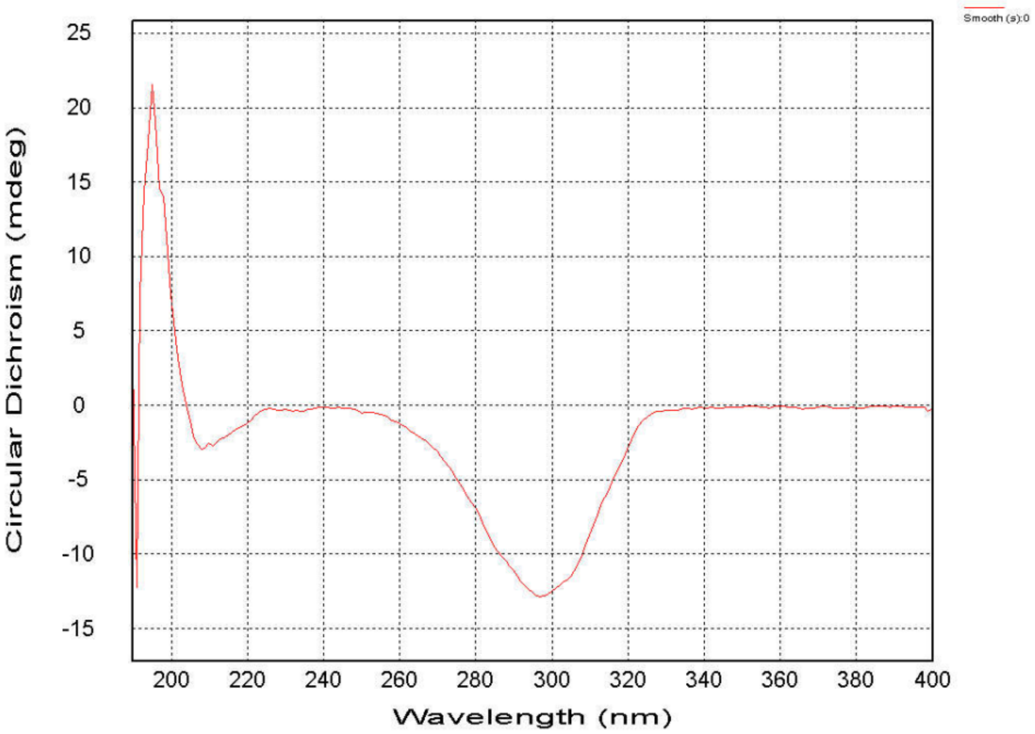

Figure S5-110. The ECD spectrum of compound **12** in MeOH

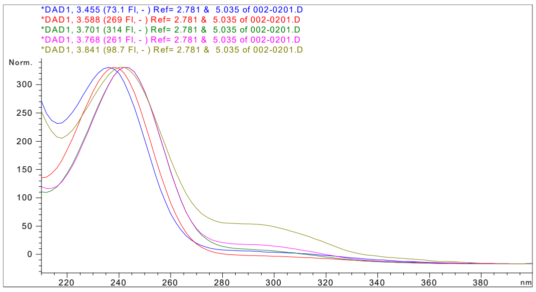

compound **1**

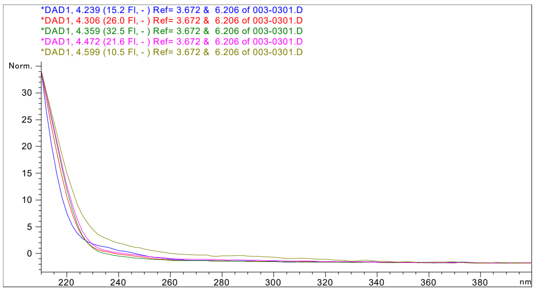

compound **2**

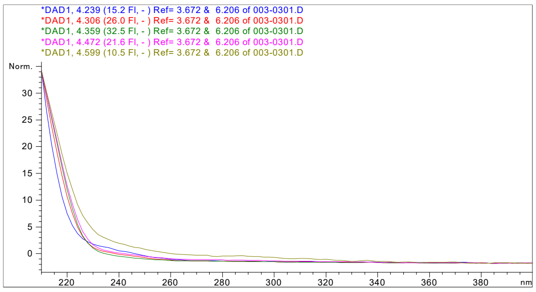

compound **3**

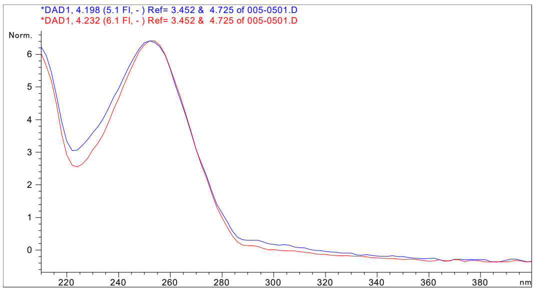

compound **4**

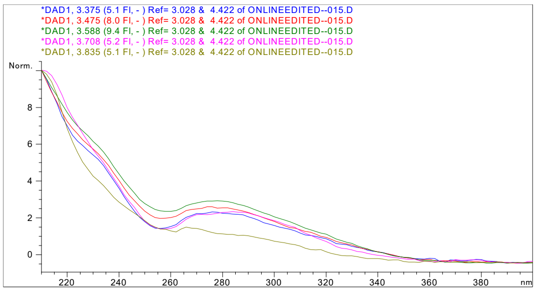

compound **5**

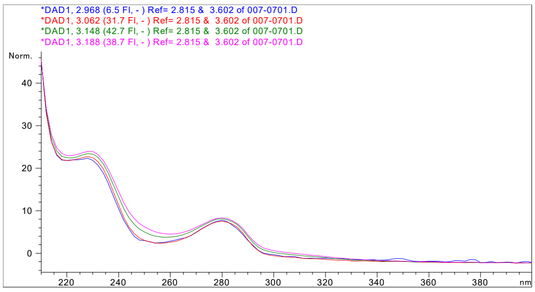

compound **6**

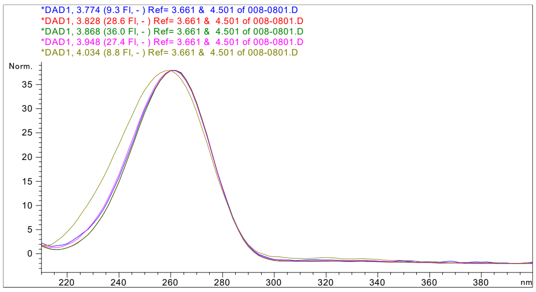

compound **7**

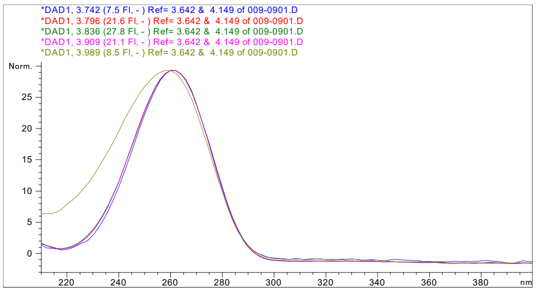

compound **8**

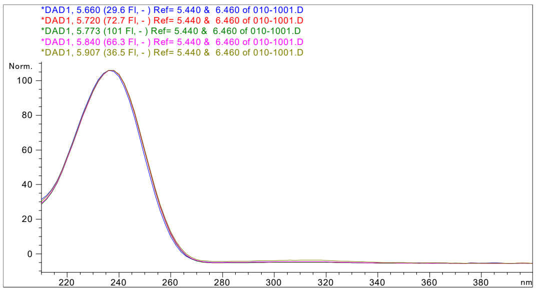

compound **9**

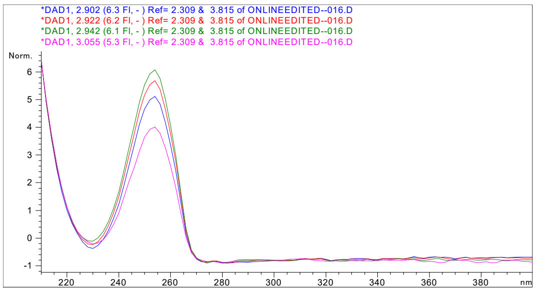

compound **10**

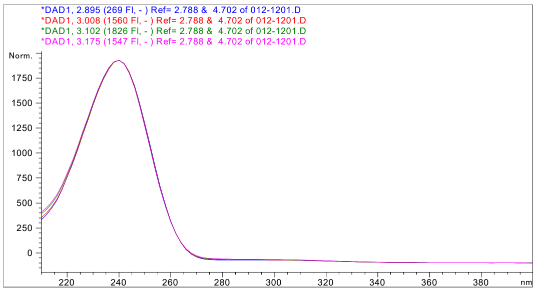

compound **11**

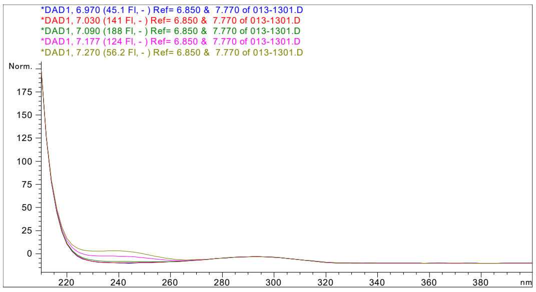

compound **12**

Figure S5-111. UV full-wavelength scan spectra of compounds **1–12**

S6. Calculation of ECD Spectra for **1**.

In general, the conformations of the model compounds were searched through the molecular Merck force field 94 (MMFF94) force field with an energy cutoff of 5.0 kcal/mol using CONFLEX software. The results showed thirty-six lowest energy conformers for both compounds. Subsequently, the searched results were optimized with b3lyp/6-31g(d) methods in methanol using the polarizable conductor calculation model (SMD) by the Computing Center High performance computing server, Shanghai Institute of Ceramics, Chinese Academy of Science. And ECD calculated with m062x/def2tzvp in methanol by the Computing Center High performance computing server. Furthermore, averaged the simulated spectra of the conformers according to the Boltzmann distribution theory and their relative Gibbs free energy (ΔG) to obtain the final spectrum [1,2]. Ultimately, the absolute configuration of the chiral center was determined to be by comparing the experiment spectra with the calculated ECD spectra using SpecDis V1.70.1 and Origin 2018 programs.

Table S6. Energy Analysis for the Conformers of **1**.

Conformers	Relative energy (kcal/mol)	Boltzmann distribution (%)	Single point energy (a.u.)
00000001(1-1)	0.000415	29.71	-812.9129390
00000005(1-2)	0.000017	20.26	-812.9133365
00000003(1-3)	0.001106	6.30	-812.9122482
00000014(1-4)	0.000753	6.02	-812.9126004
00000026(1-5)	0.002037	4.78	-812.9113164
00000007(1-6)	0.000350	4.63	-812.9130043
00000023(1-7)	0.001319	4.52	-812.9120345
00000010(1-8)	0.001031	4.08	-812.9123226
00000030(1-9)	0.001706	3.22	-812.9116477

00000027(1-10)	0.000930	3.11	-812.9124235
00000006(1-11)	0.000264	2.85	-812.9130895
00000012(1-12)	0.000000	2.00	-812.9133539
00000015(1-13)	0.001086	0.96	-812.9122679
00000029(1-14)	0.002000	0.94	-812.9113540
00000008(1-15)	0.001009	0.86	-812.9123449
00000016(1-16)	0.001786	0.86	-812.9115677
00000028(1-17)	0.001154	0.82	-812.9122002
00000002(1-18)	0.003666	0.79	-812.9096880
00000019(1-19)	0.000753	0.65	-812.9126008
00000032(1-20)	0.001015	0.56	-812.9123392
00000024(1-21)	0.000187	0.44	-812.9131665
00000025(1-22)	0.004655	0.42	-812.9086986
00000035(1-23)	0.000652	0.38	-812.9127022
00000017(1-24)	0.003360	0.35	-812.9099936
00000020(1-25)	0.001471	0.34	-812.9118825
00000039(1-26)	0.001763	0.17	-812.9115904
00000041(1-27)	0.000838	0.14	-812.9125160
00000021(1-28)	0.003720	0.08	-812.9096340
00000033(1-29)	0.004392	0.06	-812.9089622
00000009(1-30)	0.003032	0.05	-812.9103215
00000040(1-31)	0.004351	0.04	-812.9090032
00000034(1-32)	0.004013	0.04	-812.9093411
00000043(1-33)	0.003405	0.03	-812.9099490
00000048(1-34)	0.004061	0.01	-812.9092929
00000037(1-35)	0.003056	0.01	-812.9102975
00000046(1-36)	0.003685	0.003	-812.9096692

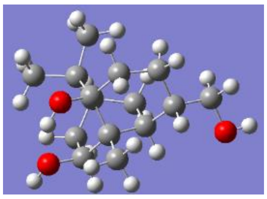

1-1

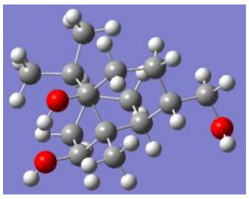

1-2

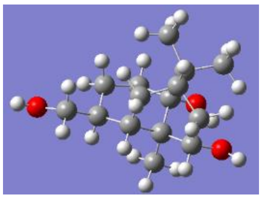

1-3

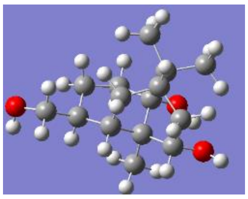

1-4

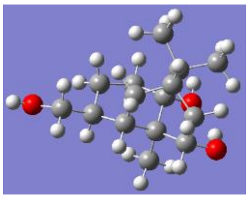

1-5

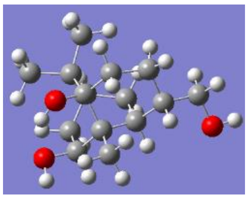

1-6

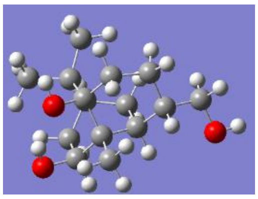

1-7

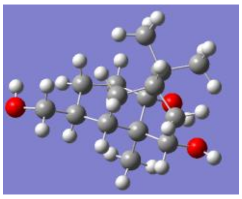

1-8

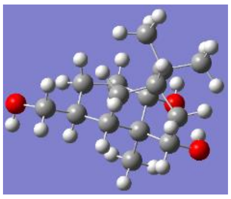

1-9

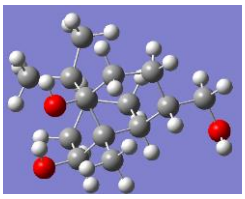

1-10

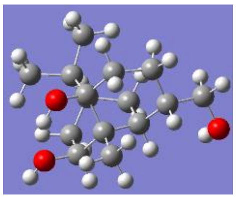

1-11

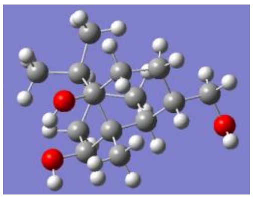

1-12

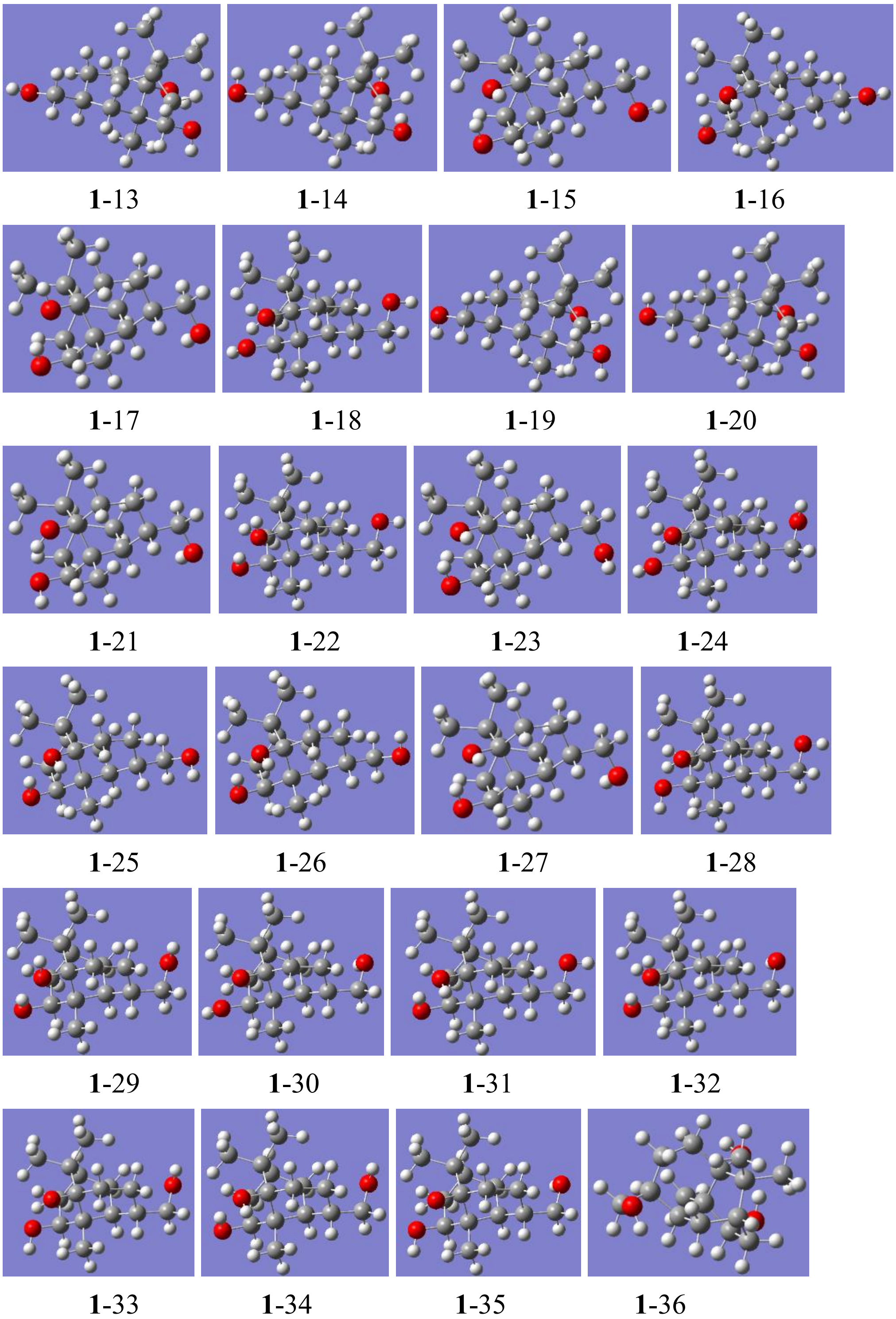

Figure S6. B3lyp/6-31g(d) optimized low-energy conformers of **1**.

S7. Calculation of ECD Spectra for **2**.

In general, the conformations of the model compounds were searched through the molecular Merck force field 94 (MMFF94) force field with an energy cutoff of 5.0 kcal/mol using CONFLEX software. The results showed seventeen lowest energy conformers for both compounds. Subsequently, the searched results were optimized

with b3lyp/6-31g(d) methods in methanol using the polarizable conductor calculation model (SMD) by the Computing Center High performance computing server, Shanghai Institute of Ceramics, Chinese Academy of Science. And ECD calculated with m062x/def2tzvp in methanol by the Computing Center High performance computing server. Furthermore, averaged the simulated spectra of the conformers according to the Boltzmann distribution theory and their relative Gibbs free energy (ΔG) to obtain the final spectrum [1,2]. Ultimately, the absolute configuration of the chiral center was determined to be by comparing the experiment spectra with the calculated ECD spectra using SpecDis V1.70.1 and Origin 2018 programs.

Table S7. Energy Analysis for the Conformers of **2**.

Conformers	Relative energy (kcal/mol)	Boltzmann distribution (%)	Single point energy (a.u.)
00000001(2-1)	0.001888	41.83	-812.9137436
00000002(2-2)	0.000271	17.81	-812.9153610
00000019(2-3)	0.005238	11.54	-812.9103940
00000007(2-4)	0.002120	6.34	-812.9135118
00000011(2-5)	0.000000	5.25	-812.9156318
00000026(2-6)	0.003257	4.58	-812.9123746
00000004(2-7)	0.003117	4.45	-812.9125152
00000021(2-8)	0.005149	1.75	-812.9104832
00000012(2-9)	0.000416	1.48	-812.9152155
00000024(2-10)	0.004876	1.30	-812.9107563
00000005(2-11)	0.001716	0.98	-812.9139158
00000006(2-12)	0.002174	0.95	-812.9134579
00000022(2-13)	0.002234	0.60	-812.9133979
00000032(2-14)	0.002081	0.39	-812.9135505
00000016(2-15)	0.002367	0.26	-812.9132644
00000030(2-16)	0.004012	0.16	-812.9116203
00000033(2-17)	0.004844	0.13	-812.9107882

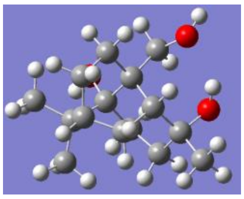
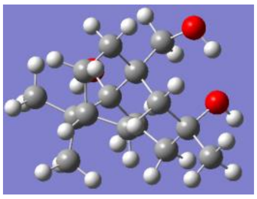
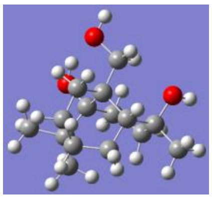
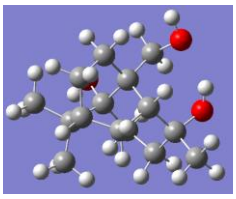

2-1 **2**-2 **2**-3 **2**-4

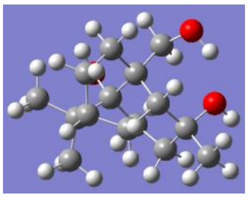
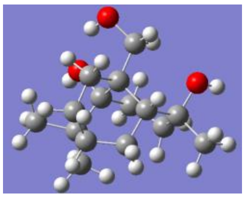
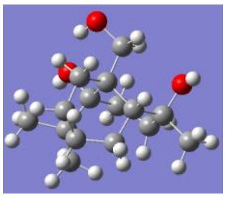
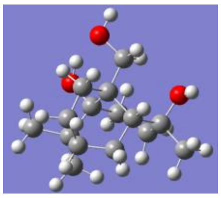

2-5 **2-6** **2-7** **2-8**

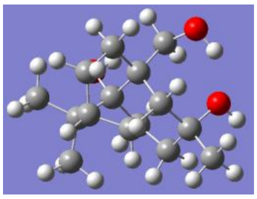 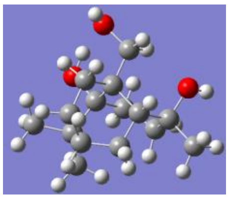 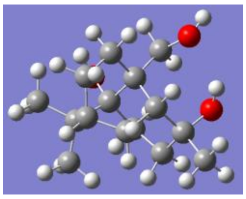 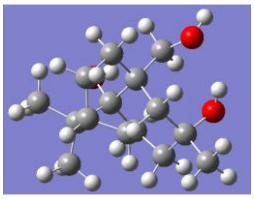

2-9 **2-10** **2-11** **2-12**

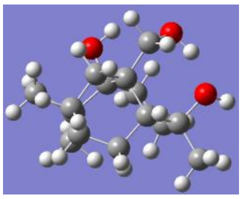 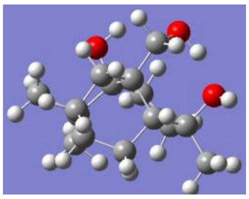 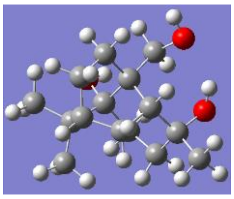 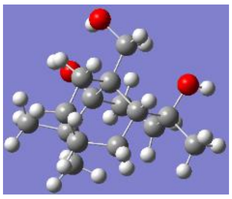

2-13 **2-14** **2-15** **2-16**

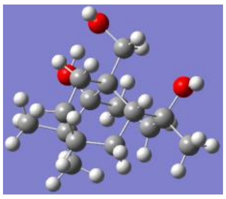

2-17

Figure S7. B3lyp/6-31g(d) optimized low-energy conformers of **2**.

S8. Calculation of ECD Spectra for **3**.

In general, the conformations of the model compounds were searched through the molecular Merck force field 94 (MMFF94) force field with an energy cutoff of 5.0 kcal/mol using CONFLEX software. The results showed seventy lowest energy conformers for both compounds. Subsequently, the searched results were optimized with b3lyp/6-31g(d) methods in methanol using the polarizable conductor calculation model (SMD) by the Computing Center High performance computing server, Shanghai Institute of Ceramics, Chinese Academy of Science. And ECD calculated with m062x/def2tzvp in methanol by the Computing Center High performance computing server. Furthermore, averaged the simulated spectra of the conformers according to the Boltzmann distribution theory and their relative Gibbs free energy (ΔG) to obtain the final spectrum [1,2]. Ultimately, the absolute configuration of the chiral center was determined to be by comparing the experiment spectra with the calculated ECD spectra using SpecDis V1.70.1 and Origin 2018 programs.

Table S8. Energy Analysis for the Conformers of **3**.

Conformers	Relative energy	Boltzmann	Single point energy

	(kcal/mol)	distribution (%)	(a.u.)
00000048(3-1)	0.001681	11.95	-812.9098022
00000001(3-2)	0.000345	11.23	-812.9111379
00000003(3-3)	0.000772	10.39	-812.9107116
00000043(3-4)	0.002088	9.86	-812.9093949
00000057(3-5)	0.002196	9.51	-812.9092875
00000047(3-6)	0.002584	8.21	-812.9088991
00000005(3-7)	0.000000	4.37	-812.9114834
00000013(3-8)	0.000450	3.75	-812.9110332
00000053(3-9)	0.002450	3.73	-812.9090332
00000049(3-10)	0.001895	2.96	-812.9095887
00000076(3-11)	0.003599	2.78	-812.9078845
00000012(3-12)	0.000660	2.75	-812.9108232
00000006(3-13)	0.000161	2.28	-812.9113222
00000079(3-14)	0.003150	1.98	-812.9083336
00000082(3-15)	0.003285	0.99	-812.9081980
00000046(3-16)	0.005347	0.87	-812.9061363
00000058(3-17)	0.004001	0.79	-812.9074822
00000084(3-18)	0.002912	0.76	-812.9085716
00000081(3-19)	0.003534	0.74	-812.9079496
00000087(3-20)	0.002799	0.68	-812.9086848
00000004(3-21)	0.001669	0.67	-812.9098141
00000064(3-22)	0.001813	0.54	-812.9096707
00000062(3-23)	0.003626	0.50	-812.9078577
00000002(3-24)	0.003571	0.43	-812.9079124
00000060(3-25)	0.002195	0.41	-812.9092886
00000050(3-26)	0.003590	0.41	-812.9078933
00000019(3-27)	0.001294	0.35	-812.9101891
00000054(3-28)	0.003367	0.31	-812.9081161
00000011(3-29)	0.002171	0.31	-812.9093119
00000061(3-30)	0.004966	0.27	-812.9065178
00000014(3-31)	0.003413	0.26	-812.9080707
00000088(3-32)	0.002886	0.25	-812.9085974
00000083(3-33)	0.003446	0.23	-812.9080373
00000066(3-34)	0.003915	0.19	-812.9075683
00000015(3-35)	0.004921	0.19	-812.9065619
00000025(3-36)	0.001826	0.18	-812.9096575
00000069(3-37)	0.003292	0.18	-812.9081913
00000116(3-38)	0.005390	0.17	-812.9060929
00000078(3-39)	0.003925	0.17	-812.9075585
00000007(3-40)	0.003138	0.16	-812.9083456
00000135(3-41)	0.005837	0.15	-812.9056467
00000113(3-42)	0.003374	0.14	-812.9081098
00000042(3-43)	0.005734	0.14	-812.9057496
00000030(3-44)	0.002063	0.13	-812.9094199
00000103(3-45)	0.003535	0.12	-812.9079483
00000123(3-46)	0.006257	0.12	-812.9052261
00000120(3-47)	0.003124	0.11	-812.9083590

00000024(3-48)	0.003229	0.11	-812.9082547
00000008(3-49)	0.004287	0.11	-812.9071966
00000026(3-50)	0.003090	0.11	-812.9083938
00000096(3-51)	0.002659	0.10	-812.9088241
00000035(3-52)	0.001450	0.10	-812.9100333
00000052(3-53)	0.004615	0.10	-812.9068688
00000086(3-54)	0.003864	0.09	-812.9076190
00000092(3-55)	0.003122	0.09	-812.9083611
00000027(3-56)	0.004579	0.07	-812.9069045
00000020(3-57)	0.002766	0.07	-812.9087177
00000099(3-58)	0.003374	0.06	-812.9081097
00000075(3-59)	0.004442	0.06	-812.9070413
00000073(3-60)	0.004952	0.06	-812.9065318
00000031(3-61)	0.003344	0.06	-812.9081389
00000140(3-62)	0.006100	0.06	-812.9053832
00000080(3-63)	0.006094	0.05	-812.9053892
00000036(3-64)	0.002801	0.05	-812.9086819
00000101(3-65)	0.002605	0.05	-812.9088787
00000021(3-66)	0.003993	0.05	-812.9074904
00000095(3-67)	0.003504	0.05	-812.9079792
00000018(3-68)	0.002652	0.05	-812.9088315
00000141(3-69)	0.005498	0.05	-812.9059858
00000132(3-70)	0.003828	0.04	-812.9076555

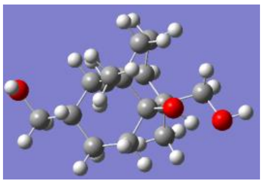
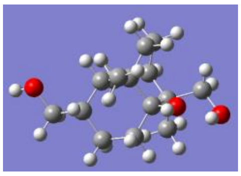
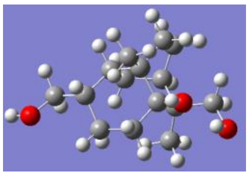
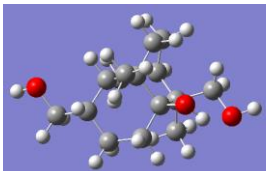

3-1 **3**-2 **3**-3 **3**-4

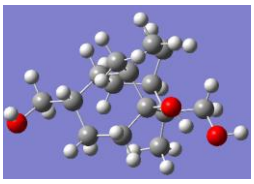
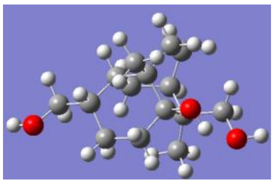
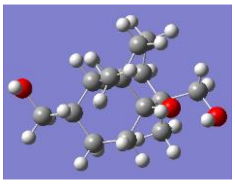
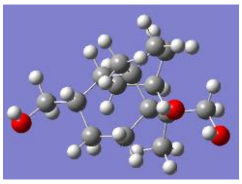

3-5 **3**-6 **3**-7 **3**-8

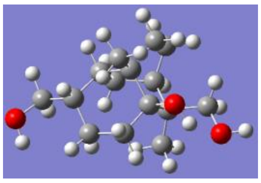
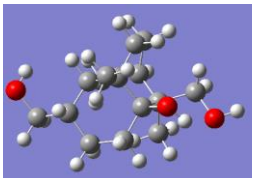
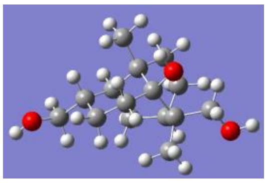
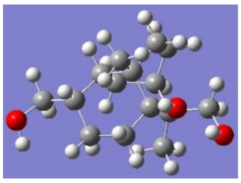

3-9 **3**-10 **3**-11 **3**-12

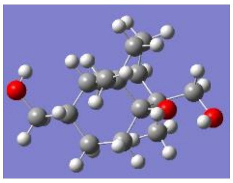
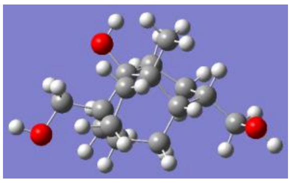
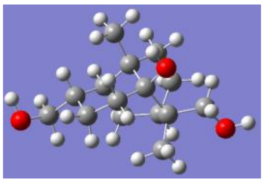
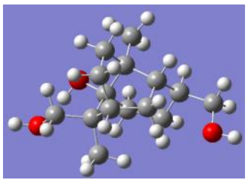

3-13 **3**-14 **3**-15 **3**-16

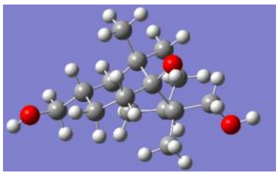 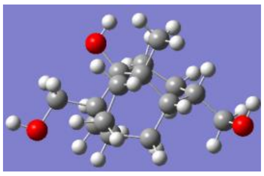 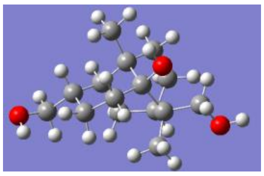 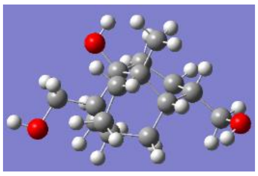

3-17 **3**-18 **3**-19 **3**-20

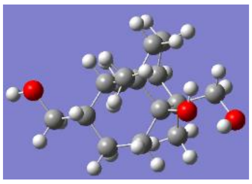 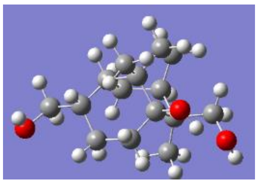 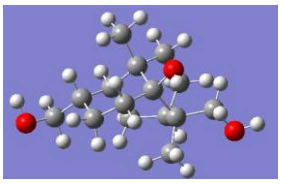 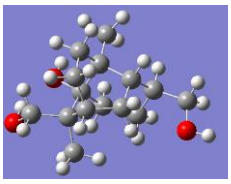

3-21 **3**-22 **3**-23 **3**-24

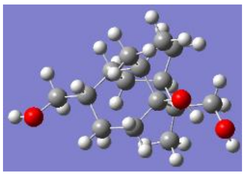 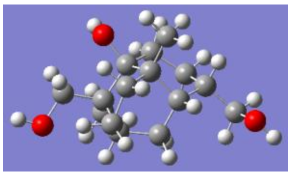 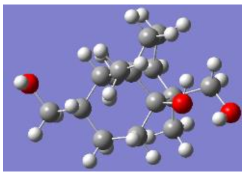 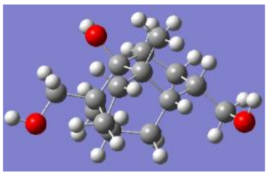

3-25 **3**-26 **3**-27 **3**-28

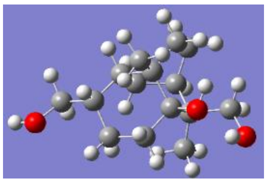 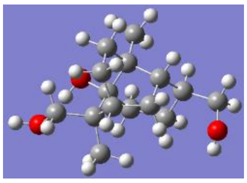 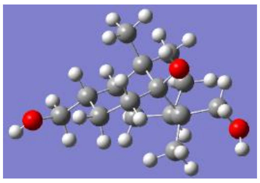 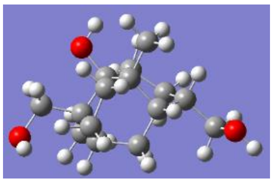

3-29 **3**-30 **3**-31 **3**-32

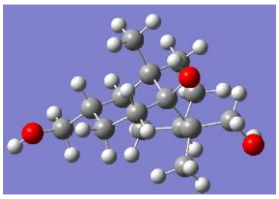 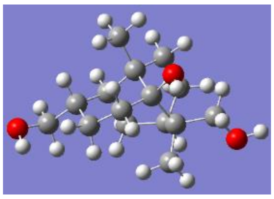 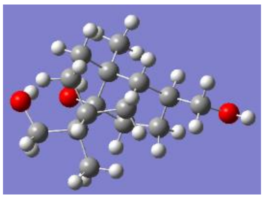 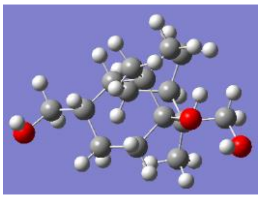

3-33 **3**-34 **3**-35 **3**-36

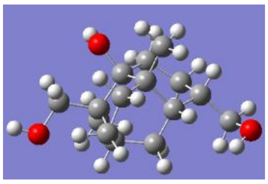 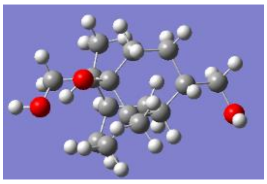 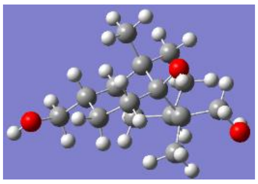 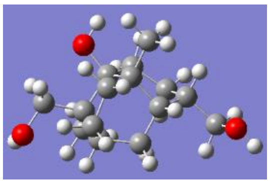

3-37 **3**-38 **3**-39 **3**-40

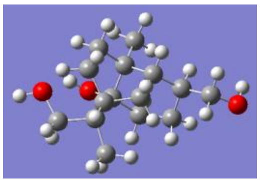 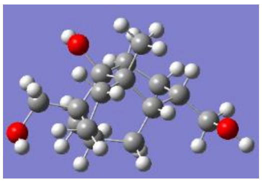 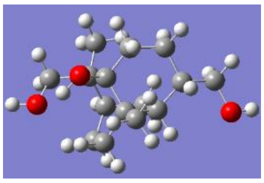 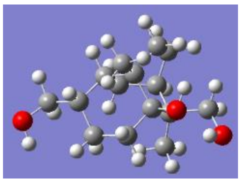

3-41 **3**-42 **3**-43 **3**-44

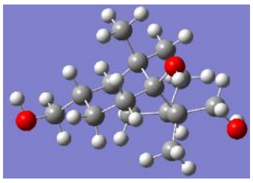
3-45

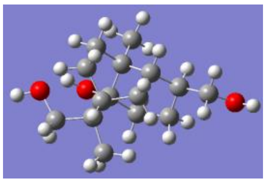
3-46

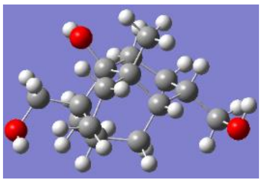
3-47

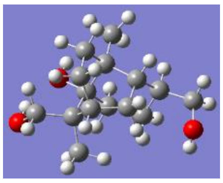
3-48

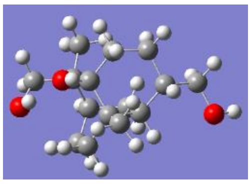
3-49

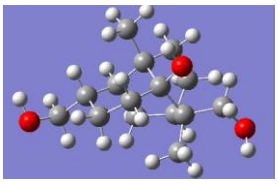
3-50

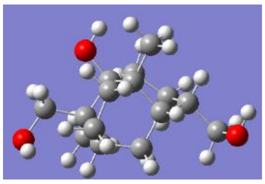
3-51

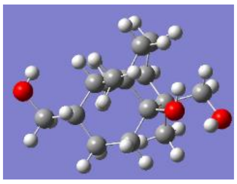
3-52

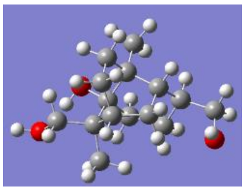
3-53

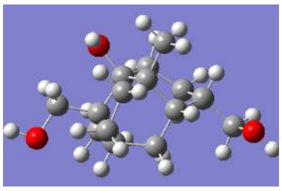
3-54

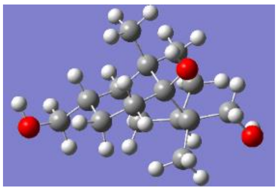
3-55

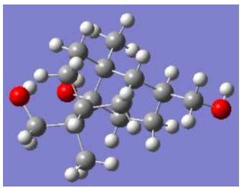
3-56

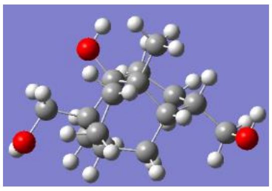
3-57

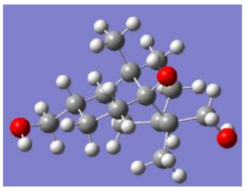
3-58

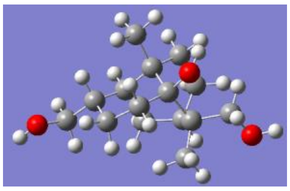
3-59

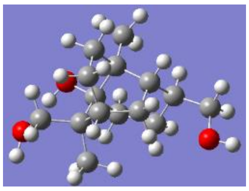
3-60

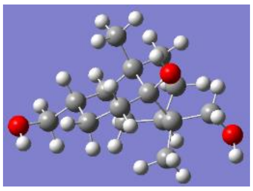
3-61

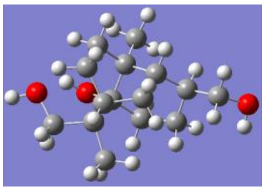
3-62

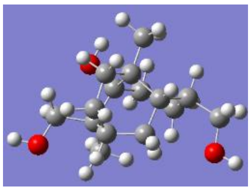
3-63

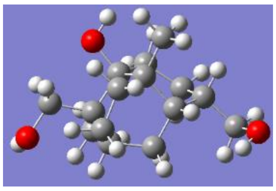
3-64

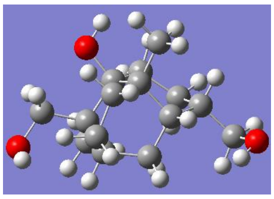
3-65

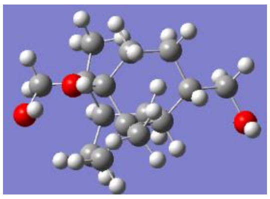
3-66

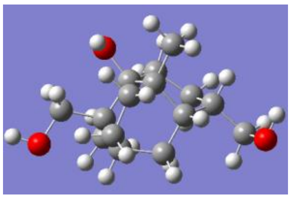
3-67

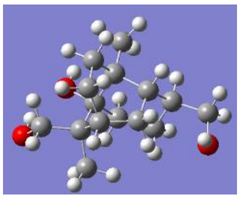
3-68

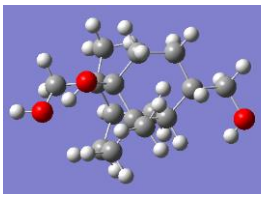
3-69

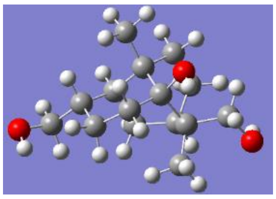
3-70

Figure S8. B3lyp/6-31g(d) optimized low-energy conformers of **3**.

S9. Calculation of ECD Spectra for **4**.

In general, the conformations of the model compounds were searched through the molecular Merck force field 94 (MMFF94) force field with an energy cutoff of 5.0 kcal/mol using CONFLEX software. The results showed three lowest energy conformers for both compounds. Subsequently, the searched results were optimized with m062x/6-311g(d,p) methods in methanol using the polarizable conductor calculation model (SMD) by the Computing Center High performance computing server, Shanghai Institute of Ceramics, Chinese Academy of Science. And ECD calculated with m062x/def2tzvp in methanol by the Computing Center High performance computing server. Furthermore, averaged the simulated spectra of the conformers according to the Boltzmann distribution theory and their relative Gibbs free energy (ΔG) to obtain the final spectrum [1,2]. Ultimately, the absolute configuration of the chiral center was determined to be by comparing the experiment spectra with the calculated ECD spectra using SpecDis V1.70.1 and Origin 2018 programs.

Table S9. Energy Analysis for the Conformers of **4**.

Conformers	Relative energy (kcal/mol)	Boltzmann distribution (%)	Single point energy (a.u.)
00000003(4-1)	0.000270	64.38	-812.9131019
00000001(4-2)	0.000000	31.84	-812.9133718
00000002(4-3)	0.000619	3.78	-812.9127530

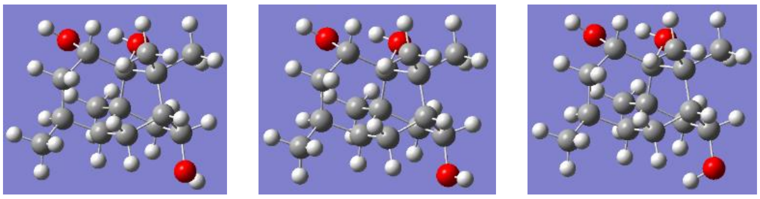

4-1 **4**-2 **4**-3

Figure S9. M062x/6-311g(d,p) optimized low-energy conformers of **4**.

S10. Calculation of ECD Spectra for **5**.

In general, the conformations of the model compounds were searched through the molecular Merck force field 94 (MMFF94) force field with an energy cutoff of 5.0 kcal/mol using CONFLEX software. The results showed nine lowest energy conformers for both compounds. Subsequently, the searched results were optimized

with b3lyp/6-31g(d) methods in methanol using the polarizable conductor calculation model (SMD) by the Computing Center High performance computing server, Shanghai Institute of Ceramics, Chinese Academy of Science. And ECD calculated with m062x/def2tzvp in methanol by the Computing Center High performance computing server. Furthermore, averaged the simulated spectra of the conformers according to the Boltzmann distribution theory and their relative Gibbs free energy (ΔG) to obtain the final spectrum [1,2]. Ultimately, the absolute configuration of the chiral center was determined to be by comparing the experiment spectra with the calculated ECD spectra using SpecDis V1.70.1 and Origin 2018 programs.

Table S10. Energy Analysis for the Conformers of **5**.

Conformers	Relative energy (kcal/mol)	Boltzmann distribution (%)	Single point energy (a.u.)
00000002(5-1)	0.000361	43.05	-698.3740090
00000001(5-2)	0.000702	28.63	-698.3736683
00000004(5-3)	0.000443	6.99	-698.3739270
00000008(5-4)	0.000000	5.95	-698.3743701
00000005(5-5)	0.000338	5.06	-698.3740324
00000007(5-6)	0.000146	4.33	-698.3742236
00000003(5-7)	0.000613	3.92	-698.3737570
00000012(5-8)	0.000176	1.20	-698.3741939
00000010(5-9)	0.000283	0.86	-698.3740873

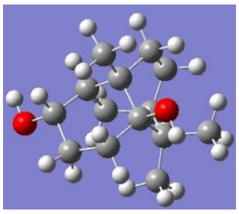
5-1

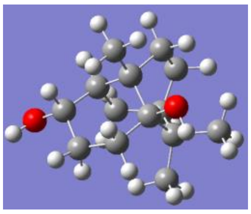
5-2

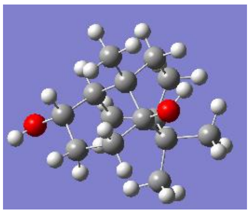
5-3

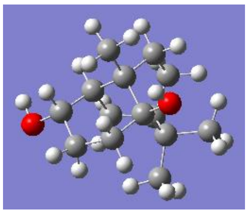
5-4

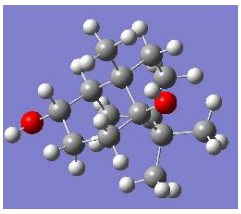
5-5

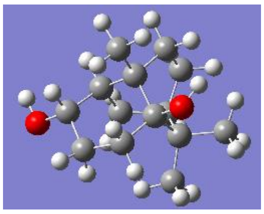
5-6

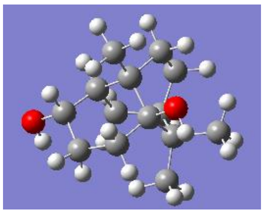
5-7

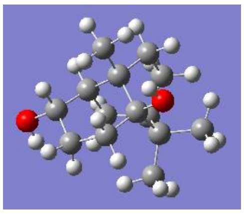
5-8

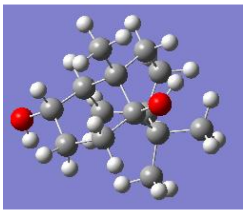
5-9

Figure S10. B3lyp/6-31g(d) optimized low-energy conformers of **5**.

S11. Calculation of ECD Spectra for **6**.

In general, the conformations of the model compounds were searched through the molecular Merck force field 94 (MMFF94) force field with an energy cutoff of 5.0 kcal/mol using CONFLEX software. The results showed twelve lowest energy conformers for both compounds. Subsequently, the searched results were optimized with b3lyp/6-31g(d) methods in methanol using the polarizable conductor calculation model (SMD) by the Computing Center High performance computing server, Shanghai Institute of Ceramics, Chinese Academy of Science. And ECD calculated with m062x/def2tzvp in methanol by the Computing Center High performance computing server. Furthermore, averaged the simulated spectra of the conformers according to the Boltzmann distribution theory and their relative Gibbs free energy (ΔG) to obtain the final spectrum [1,2]. Ultimately, the absolute configuration of the chiral center was determined to be by comparing the experiment spectra with the calculated ECD spectra using SpecDis V1.70.1 and Origin 2018 programs.

Table S11. Energy Analysis for the Conformers of **6**.

Conformers	Relative energy (kcal/mol)	Boltzmann distribution (%)	Single point energy (a.u.)
00000001(6-1)	0.000315	56.79	-773.6089473
00000002(6-2)	0.000011	16.21	-773.6092513
00000011(6-3)	0.001019	7.41	-773.6082429
00000010(6-4)	0.001389	6.48	-773.6078727
00000005(6-5)	0.000305	5.17	-773.6089574
00000003(6-6)	0.000258	4.28	-773.6090037
00000007(6-7)	0.000000	1.46	-773.6092622
00000013(6-8)	0.001208	0.55	-773.6080542
00000012(6-9)	0.001309	0.54	-773.6079528
00000014(6-10)	0.000850	0.53	-773.6084119
00000008(6-11)	0.000212	0.50	-773.6090503
00000016(6-12)	0.001097	0.07	-773.6081648

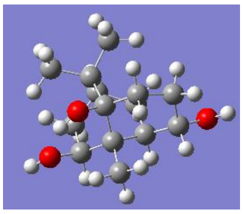

6-1

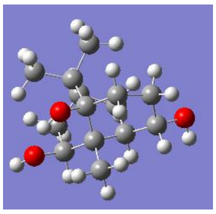

6-2

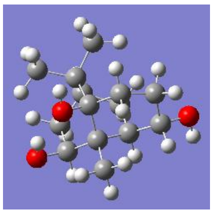

6-3

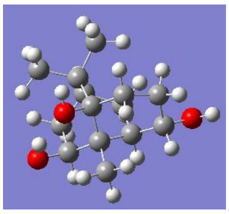

6-4

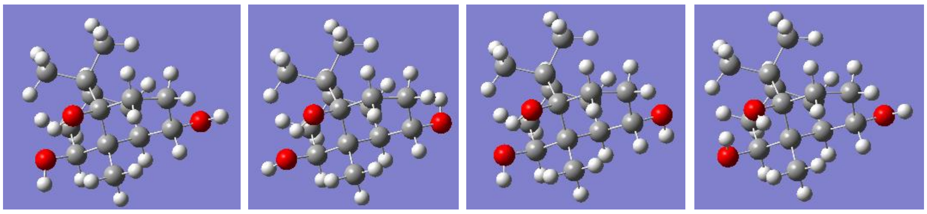

6-5 **6**-6 **6**-7 **6**-8

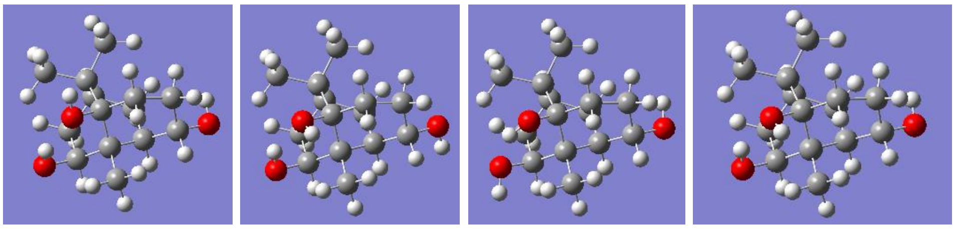

6-9 **6**-10 **6**-11 **6**-12

Figure S11. B3lyp/6-31g(d) optimized low-energy conformers of **6**.

S12. Calculation of ECD Spectra for **7**.

In general, the conformations of the model compounds were searched through the molecular Merck force field 94 (MMFF94) force field with an energy cutoff of 5.0 kcal/mol using CONFLEX software. The results showed fifty lowest energy conformers for both compounds. Subsequently, the searched results were optimized with b3lyp/6-31g(d) methods in methanol using the polarizable conductor calculation model (SMD) by the Computing Center High performance computing server, Shanghai Institute of Ceramics, Chinese Academy of Science. And ECD calculated with m062x/def2tzvp in methanol by the Computing Center High performance computing server. Furthermore, averaged the simulated spectra of the conformers according to the Boltzmann distribution theory and their relative Gibbs free energy (ΔG) to obtain the final spectrum [1,2]. Ultimately, the absolute configuration of the chiral center was determined to be by comparing the experiment spectra with the calculated ECD spectra using SpecDis V1.70.1 and Origin 2018 programs.

Table S12. Energy Analysis for the Conformers of **7**.

Conformers	Relative energy (kcal/mol)	Boltzmann distribution (%)	Single point energy (a.u.)
00000025(7-1)	0.000492	22.61	-811.6985163
00000013(7-2)	0.003209	17.60	-811.6957996
00000014(7-3)	0.001572	11.26	-811.6974363
00000005(7-4)	0.003052	6.52	-811.6959569
00000062(7-5)	0.002636	4.92	-811.6963724
00000053(7-6)	0.003330	4.41	-811.6956782

00000040(7-7)	0.000000	4.34	-811.6990086
00000031(7-8)	0.001021	4.06	-811.6979878
00000023(7-9)	0.003976	2.21	-811.6950330
00000093(7-10)	0.001411	1.51	-811.6975971
00000038(7-11)	0.001107	1.50	-811.6979018
00000022(7-12)	0.002987	1.46	-811.6960215
00000020(7-13)	0.002996	1.44	-811.6960126
00000033(7-14)	0.000618	1.33	-811.6983904
00000061(7-15)	0.002398	1.32	-811.6966102
00000064(7-16)	0.000318	1.13	-811.6986909
00000034(7-17)	0.002620	0.90	-811.6963884
00000027(7-18)	0.001055	0.85	-811.6979538
00000037(7-19)	0.002970	0.80	-811.6960381
00000026(7-20)	0.000964	0.80	-811.6980445
00000054(7-21)	0.002596	0.78	-811.6964124
00000021(7-22)	0.002795	0.73	-811.6962137
00000126(7-23)	0.003081	0.70	-811.6959280
00000032(7-24)	0.000315	0.54	-811.6986937
00000028(7-25)	0.003314	0.49	-811.6956949
00000097(7-26)	0.002385	0.48	-811.6966231
00000070(7-27)	0.003698	0.45	-811.6953107
00000121(7-28)	0.001047	0.41	-811.6979617
00000016(7-29)	0.002437	0.40	-811.6965714
00000060(7-30)	0.002989	0.36	-811.6960195
00000004(7-31)	0.004932	0.33	-811.6940769
00000015(7-32)	0.002469	0.32	-811.6965396
00000057(7-33)	0.004667	0.24	-811.6943411
00000045(7-34)	0.002088	0.23	-811.6969210
00000041(7-35)	0.003066	0.22	-811.6959429
00000044(7-36)	0.002600	0.21	-811.6964088
00000051(7-37)	0.003684	0.20	-811.6953247
00000119(7-38)	0.001821	0.18	-811.6971879
00000069(7-39)	0.002586	0.17	-811.6964225
00000048(7-40)	0.005122	0.14	-811.6938866
00000059(7-41)	0.003346	0.12	-811.6956625
00000118(7-42)	0.004203	0.09	-811.6948060
00000036(7-43)	0.005235	0.02	-811.6937731
00000065(7-44)	0.002616	0.05	-811.6963925
00000103(7-45)	0.004607	0.03	-811.6944019
00000099(7-46)	0.001642	0.02	-811.6973667
00000050(7-47)	0.003884	0.02	-811.6951250
00000111(7-48)	0.001927	0.04	-811.6970818
00000081(7-49)	0.002482	0.07	-811.6965266
00000110(7-50)	0.001820	0.08	-811.6971882

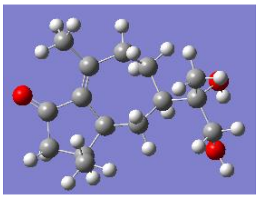

7-1

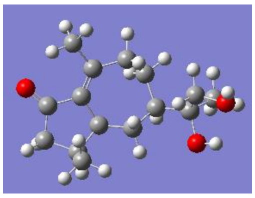

7-2

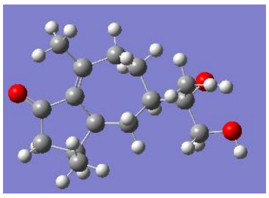

7-3

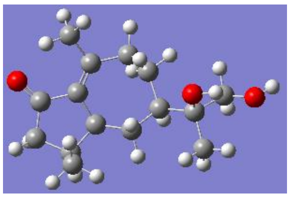

7-4

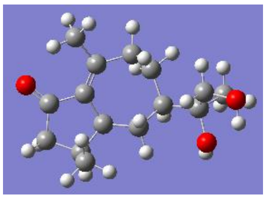

7-5

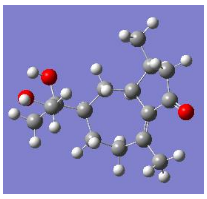

7-6

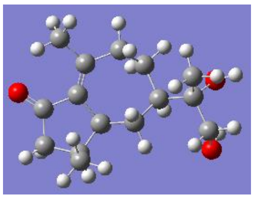

7-7

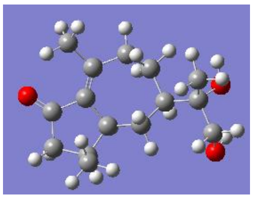

7-8

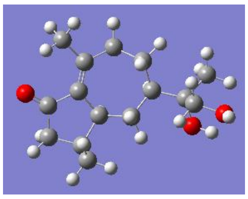

7-9

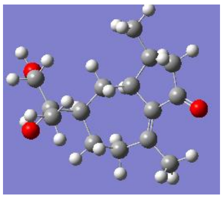

7-10

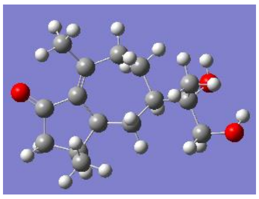

7-11

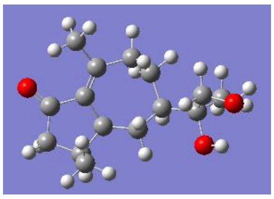

7-12

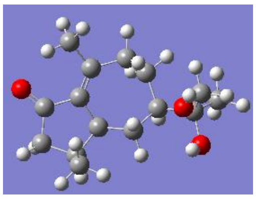

7-13

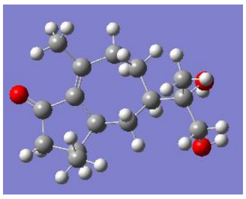

7-14

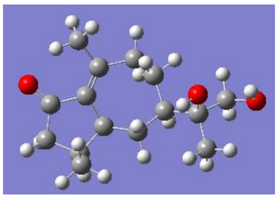

7-15

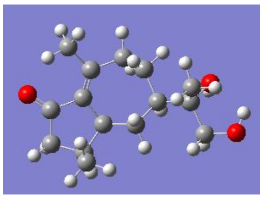

7-16

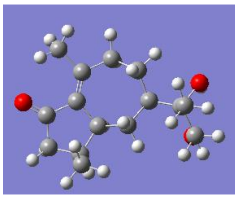

7-17

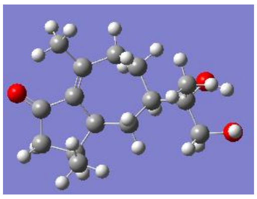

7-18

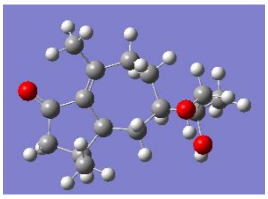

7-19

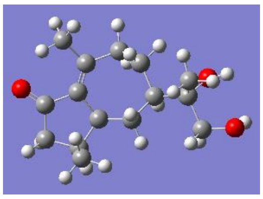

7-20

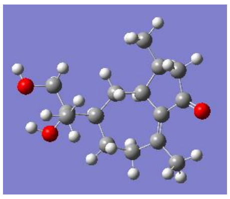

7-21

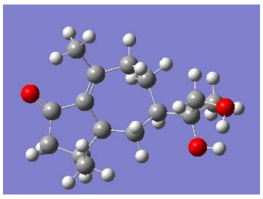

7-22

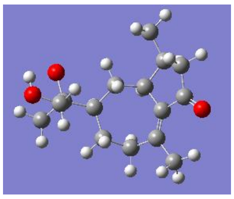

7-23

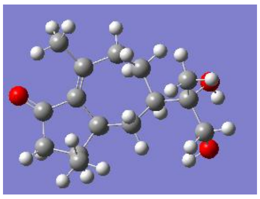

7-24

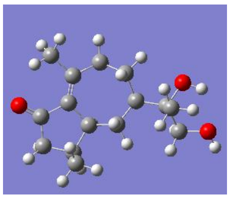

7-25

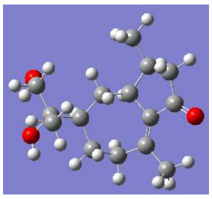

7-26

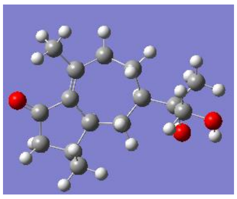

7-27

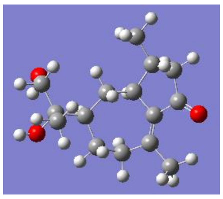

7-28

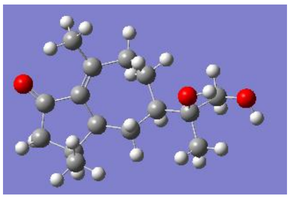 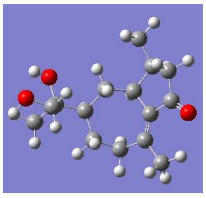 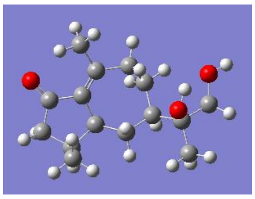 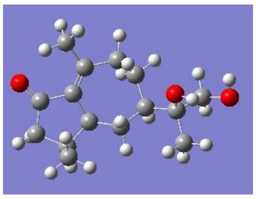

7-29 **7**-30 **7**-31 **7**-32

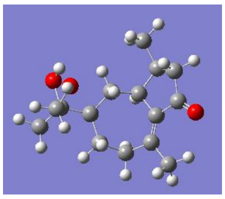 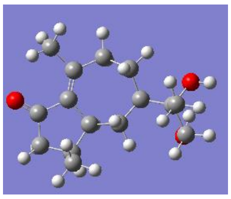 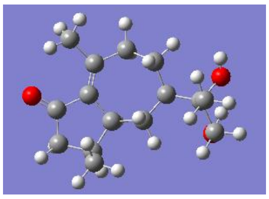 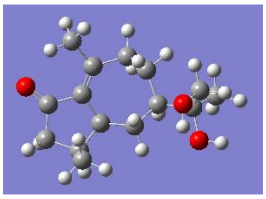

7-33 **7**-34 **7**-35 **7**-36

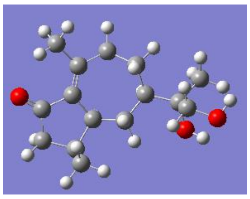 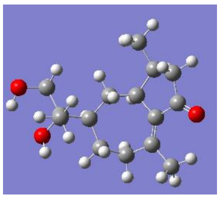 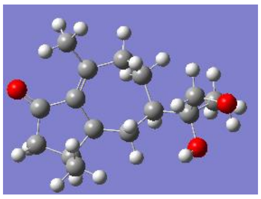 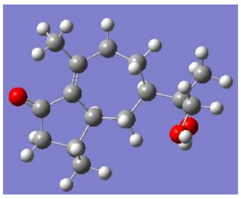

7-37 **7**-38 **7**-39 **7**-40

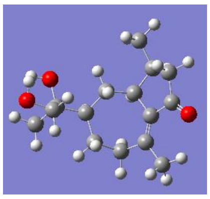 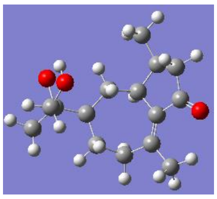 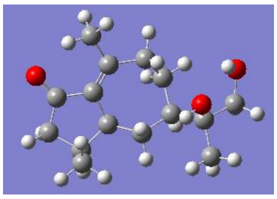 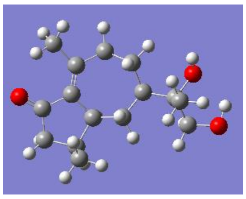

7-41 **7**-42 **7**-43 **7**-44

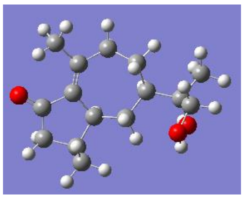 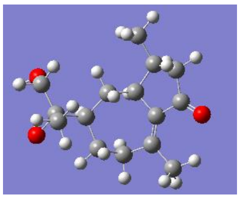 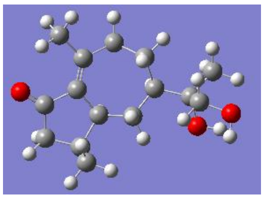 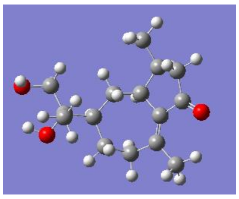

7-45 **7**-46 **7**-47 **7**-48

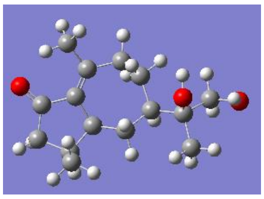 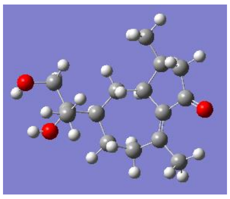

7-49 **7**-50

Figure S12. B3lyp/6-31g(d) optimized low-energy conformers of **7**.

S13. Calculation of ECD Spectra for **8**.

In general, the conformations of the model compounds were searched through the molecular Merck force field 94 (MMFF94) force field with an energy cutoff of 5.0 kcal/mol using CONFLEX software. The results showed sixty lowest energy conformers for both compounds. Subsequently, the searched results were optimized

with b3lyp/6-31g(d) methods in methanol using the polarizable conductor calculation model (SMD) by the Computing Center High performance computing server, Shanghai Institute of Ceramics, Chinese Academy of Science. And ECD calculated with m062x/def2tzvp in methanol by the Computing Center High performance computing server. Furthermore, averaged the simulated spectra of the conformers according to the Boltzmann distribution theory and their relative Gibbs free energy (ΔG) to obtain the final spectrum [1,2]. Ultimately, the absolute configuration of the chiral center was determined to be by comparing the experiment spectra with the calculated ECD spectra using SpecDis V1.70.1 and Origin 2018 programs.

Table S13. Energy Analysis for the Conformers of **8**.

Conformers	Relative energy (kcal/mol)	Boltzmann distribution (%)	Single point energy (a.u.)
00000011(8-1)	0.001467	22.43	-811.6960453
00000002(8-2)	0.000847	16.49	-811.6966650
00000016(8-3)	0.000533	12.13	-811.6969789
00000074(8-4)	0.000098	5.82	-811.6974140
00000005(8-5)	0.001898	5.27	-811.6956139
00000041(8-6)	0.000238	3.82	-811.6972740
00000045(8-7)	0.001315	3.36	-811.6961970
00000071(8-8)	0.000231	2.17	-811.6972817
00000014(8-9)	0.001260	2.12	-811.6962527
00000020(8-10)	0.002108	1.98	-811.6954040
00000048(8-11)	0.001874	1.93	-811.6956379
00000065(8-12)	0.000250	1.91	-811.6972619
00000061(8-13)	0.000093	1.81	-811.6974190
00000018(8-14)	0.000626	1.66	-811.6968859
00000069(8-15)	0.001199	1.40	-811.6963131
00000003(8-16)	0.001793	1.32	-811.6957196
00000057(8-17)	0.002105	1.13	-811.6954070
00000001(8-18)	0.002411	1.10	-811.6951008
00000027(8-19)	0.002427	1.01	-811.6950850
00000019(8-20)	0.001221	1.00	-811.6962912
00000025(8-21)	0.000116	0.99	-811.6973961
00000076(8-22)	0.000454	0.92	-811.6970583
00000013(8-23)	0.000402	0.88	-811.6971106
00000130(8-24)	0.000000	0.56	-811.6975123
00000034(8-25)	0.001396	0.55	-811.6961160
00000119(8-26)	0.001379	0.48	-811.6961337
00000026(8-27)	0.001037	0.45	-811.6964749
00000030(8-28)	0.001579	0.40	-811.6959332
00000082(8-29)	0.002029	0.33	-811.6954835
00000066(8-30)	0.000935	0.30	-811.6965769

00000134(8-31)	0.001021	0.30	-811.6964914
00000031(8-32)	0.002180	0.29	-811.6953320
00000051(8-33)	0.000620	0.28	-811.6968918
00000006(8-34)	0.002534	0.23	-811.6949785
00000120(8-35)	0.002032	0.20	-811.6954806
00000064(8-36)	0.002384	0.20	-811.6951284
00000102(8-37)	0.001367	0.19	-811.6961451
00000123(8-38)	0.000655	0.18	-811.6968574
00000063(8-39)	0.000949	0.18	-811.6965632
00000052(8-40)	0.000659	0.16	-811.6968528
00000054(8-41)	0.001285	0.16	-811.6962270
00000047(8-42)	0.000020	0.15	-811.6974920
00000080(8-43)	0.000628	0.15	-811.6968847
00000073(8-44)	0.001639	0.14	-811.6958733
00000060(8-45)	0.001414	0.12	-811.6960985
00000151(8-46)	0.000083	0.11	-811.6974289
00000055(8-47)	0.001313	0.10	-811.6961988
00000039(8-48)	0.001116	0.09	-811.6963965
00000077(8-49)	0.001305	0.09	-811.6962077
00000092(8-50)	0.001285	0.09	-811.6962272
00000007(8-51)	0.002076	0.08	-811.6954362
00000042(8-52)	0.003108	0.07	-811.6944044
00000097(8-53)	0.002021	0.07	-811.6954914
00000104(8-54)	0.000854	0.07	-811.6966579
00000093(8-55)	0.001312	0.06	-811.6961999
00000156(8-56)	0.001123	0.05	-811.6963895
00000036(8-57)	0.003280	0.05	-811.6942321
00000173(8-58)	0.000853	0.04	-811.6966588
00000098(8-59)	0.002241	0.04	-811.6952711
00000086(8-60)	0.002831	0.04	-811.6946811

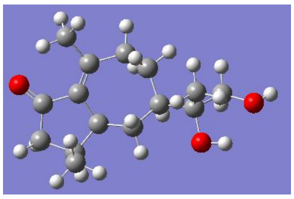

8-1

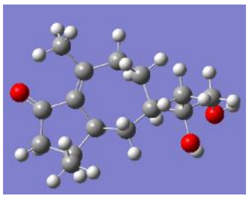

8-2

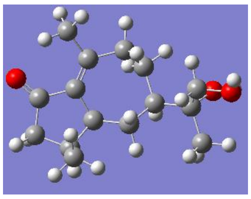

8-3

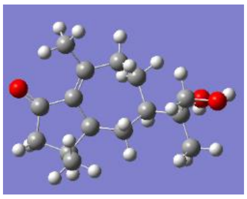

8-4

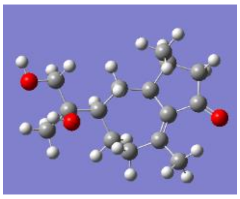

8-5

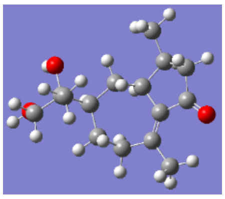

8-6

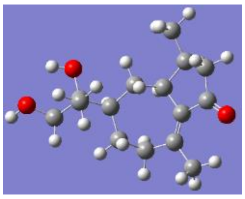

8-7

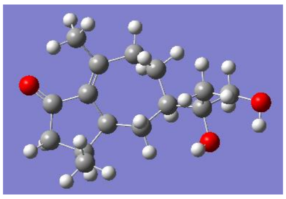

8-8

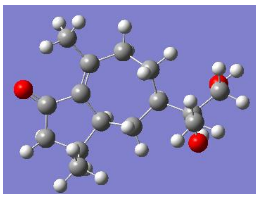
8-9
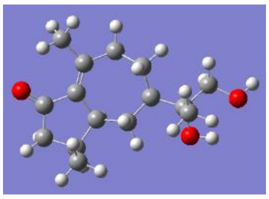
8-10
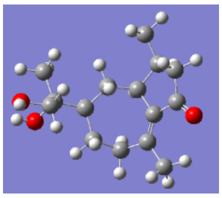
8-11
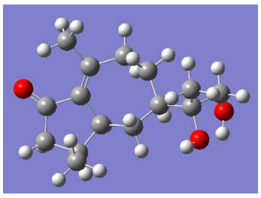
8-12
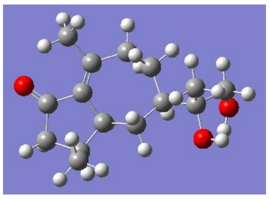
8-13
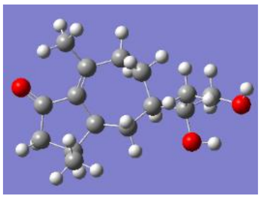
8-14
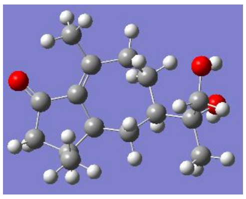
8-15
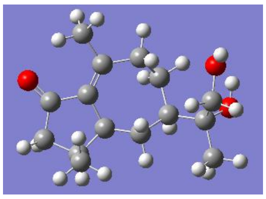
8-16
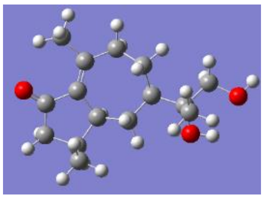
8-17
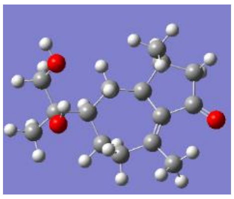
8-18
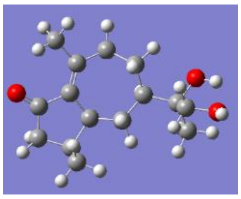
8-19
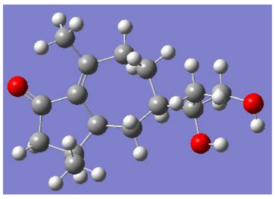
8-20
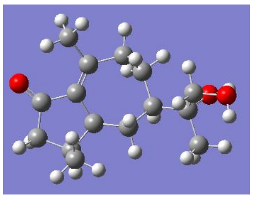
8-21
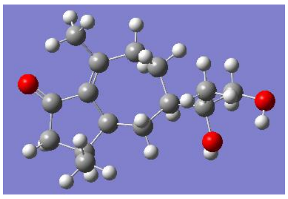
8-22
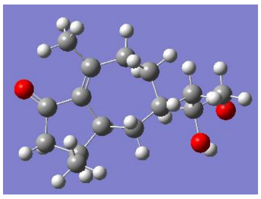
8-23
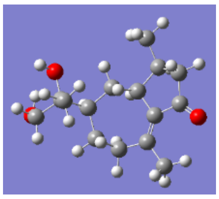
8-24
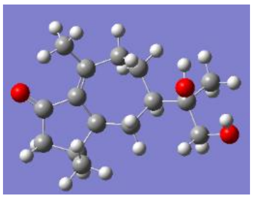
8-25
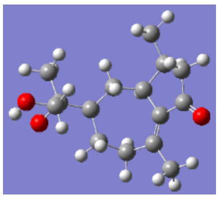
8-26
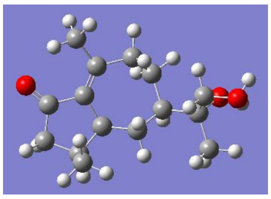
8-27
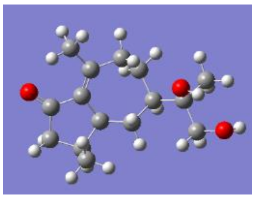
8-28
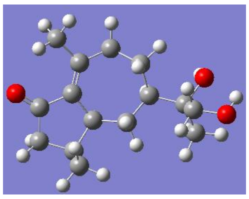
8-29
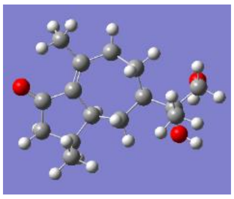
8-30
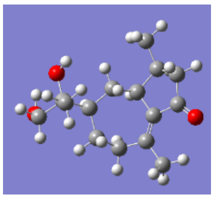
8-31
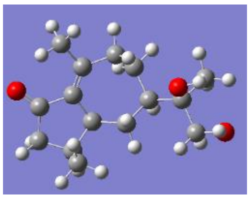
8-32
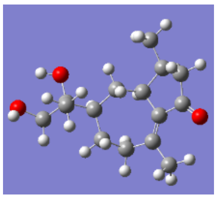
8-33
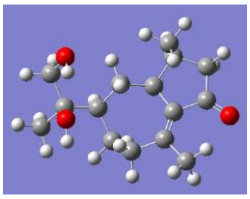
8-34
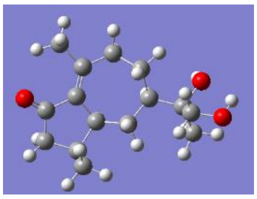
8-35
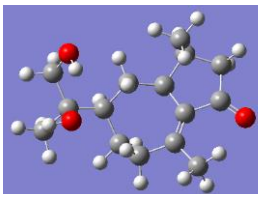
8-36

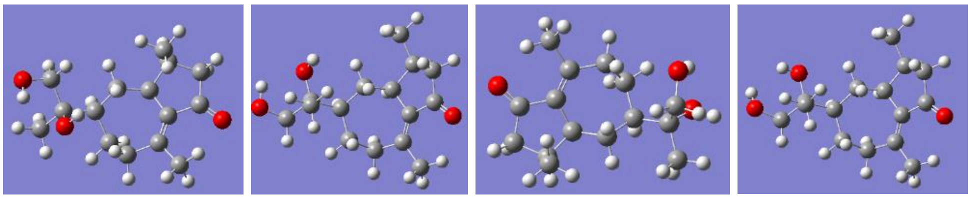

8-37 **8**-38 **8**-39 **8**-40

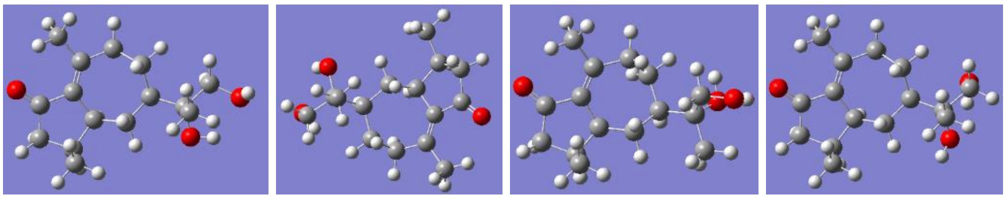

8-41 **8**-42 **8**-43 **8**-44

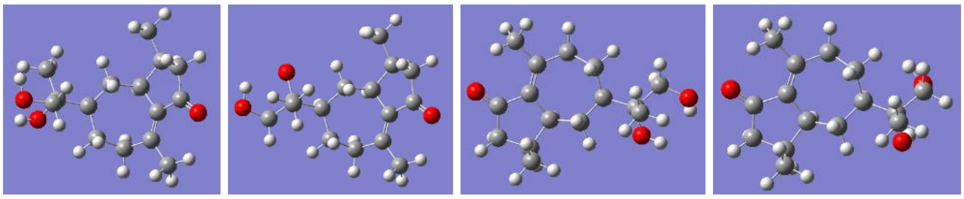

8-45 **8**-46 **8**-47 **8**-48

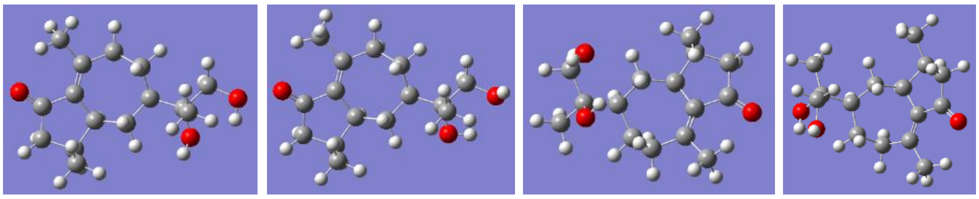

8-49 **8**-50 **8**-51 **8**-52

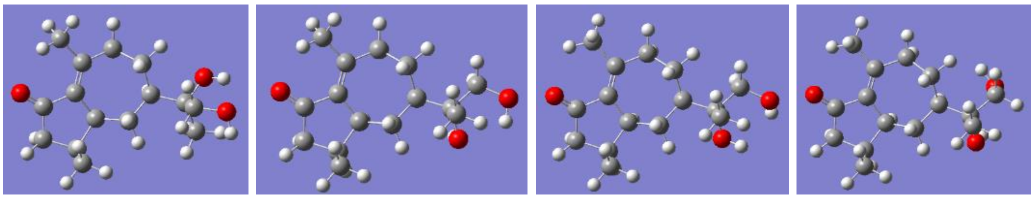

8-53 **8**-54 **8**-55 **8**-56

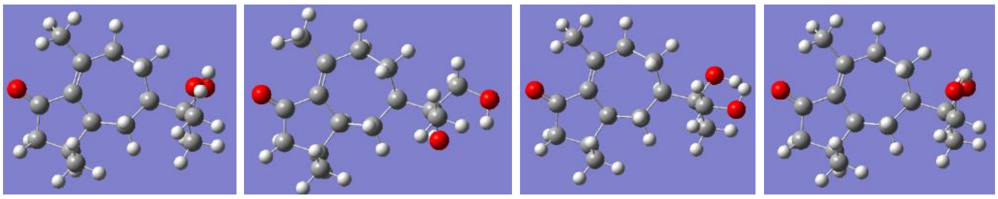

8-57 **8**-58 **8**-59 **8**-60

Figure S13. B3lyp/6-31g(d) optimized low-energy conformers of **8**.

S14. Calculation of ECD Spectra for **9**.

In general, the conformations of the model compounds were searched through the molecular Merck force field 94 (MMFF94) force field with an energy cutoff of 5.0 kcal/mol using CONFLEX software. The results showed twenty-eight lowest energy conformers for both compounds. Subsequently, the searched results were optimized

with b3lyp/6-31g(d) methods in methanol using the polarizable conductor calculation model (SMD) by the Computing Center High performance computing server, Shanghai Institute of Ceramics, Chinese Academy of Science. And ECD calculated with m062x/def2tzvp in methanol by the Computing Center High performance computing server. Furthermore, averaged the simulated spectra of the conformers according to the Boltzmann distribution theory and their relative Gibbs free energy (ΔG) to obtain the final spectrum [1,2]. Ultimately, the absolute configuration of the chiral center was determined to be by comparing the experiment spectra with the calculated ECD spectra using SpecDis V1.70.1 and Origin 2018 programs.

Table S14. Energy Analysis for the Conformers of **9**.

Conformers	Relative energy (kcal/mol)	Boltzmann distribution (%)	Single point energy (a.u.)
00000003(9-1)	0.000000	27.32	-735.2461878
00000010(9-2)	0.000125	24.46	-735.2460624
00000001(9-3)	0.000333	9.05	-735.2458544
00000002(9-4)	0.000432	6.84	-735.2457555
00000004(9-5)	0.000592	6.68	-735.2455960
00000011(9-6)	0.000470	4.42	-735.2457180
00000016(9-7)	0.000623	4.27	-735.2455649
00000015(9-8)	0.004294	3.84	-735.2418935
00000019(9-9)	0.000125	2.63	-735.2460630
00000005(9-10)	0.000966	1.25	-735.2452216
00000009(9-11)	0.004767	1.07	-735.2414210
00000031(9-12)	0.000624	0.89	-735.2455641
00000018(9-13)	0.003362	0.84	-735.2428261
00000029(9-14)	0.004307	0.77	-735.2418810
00000020(9-15)	0.001053	0.76	-735.2451351
00000017(9-16)	0.003605	0.67	-735.2425827
00000012(9-17)	0.003074	0.64	-735.2431138
00000022(9-18)	0.004175	0.52	-735.2420124
00000028(9-19)	0.002951	0.50	-735.2432368
00000014(9-20)	0.003192	0.46	-735.2429955
00000040(9-21)	0.003835	0.45	-735.2423533
00000008(9-22)	0.002622	0.38	-735.2435662
00000026(9-23)	0.003274	0.26	-735.2429137
00000007(9-24)	0.003302	0.20	-735.2428854
00000024(9-25)	0.003923	0.19	-735.2422645
00000027(9-26)	0.003531	0.18	-735.2426569
00000043(9-27)	0.005436	0.14	-735.2407513
00000046(9-28)	0.000469	0.10	-735.2457185

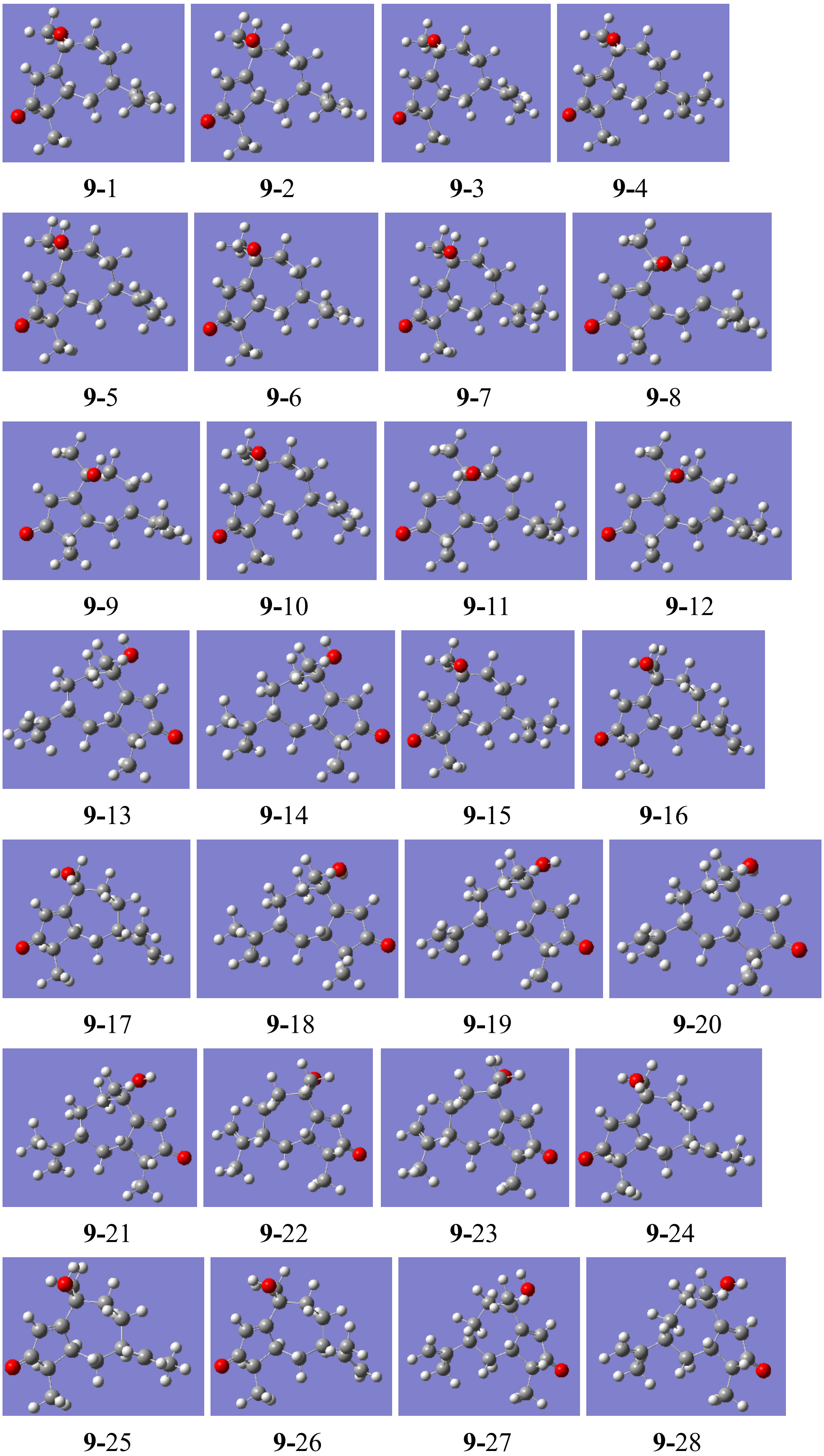

Figure S14. B3lyp/6-31g(d) optimized low-energy conformers of **9**.

S15. Calculation of ECD Spectra for **10**.

In general, the conformations of the model compounds were searched through the molecular Merck force field 94 (MMFF94) force field with an energy cutoff of 5.0 kcal/mol using CONFLEX software. The results showed ten lowest energy conformers for both compounds. Subsequently, the searched results were optimized with b3lyp/6-31g(d) methods in methanol using the polarizable conductor calculation model (SMD) by the Computing Center High performance computing server, Shanghai Institute of Ceramics, Chinese Academy of Science. And ECD calculated with m062x/def2tzvp in methanol by the Computing Center High performance computing server. Furthermore, averaged the simulated spectra of the conformers according to the Boltzmann distribution theory and their relative Gibbs free energy (ΔG) to obtain the final spectrum [1,2]. Ultimately, the absolute configuration of the chiral center was determined to be by comparing the experiment spectra with the calculated ECD spectra using SpecDis V1.70.1 and Origin 2018 programs.

Table S15. Energy Analysis for the Conformers of **10**.

Conformers	Relative energy (kcal/mol)	Boltzmann distribution (%)	Single point energy (a.u.)
00000014(10-1)	0.000844	28.96	-735.2447281
00000015(10-2)	0.000000	27.93	-735.2455725
00000011(10-3)	0.000339	21.63	-735.2452331
00000009(10-4)	0.001768	8.18	-735.2438049
00000010(10-5)	0.001078	6.86	-735.2444943
00000006(10-6)	0.001362	5.60	-735.2442103
00000012(10-7)	0.006354	0.15	-735.2392187
00000020(10-8)	0.005404	0.08	-735.2401682
00000025(10-9)	0.006251	0.07	-735.2393214
00000024(10-10)	0.004687	0.06	-735.2408851

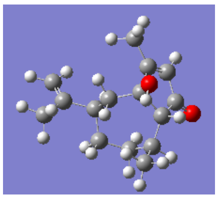
10-1

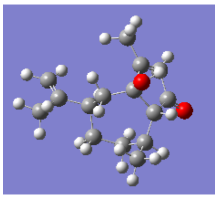
10-2

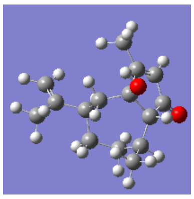
10-3

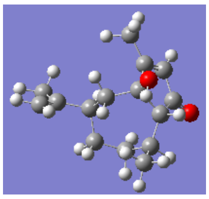
10-4

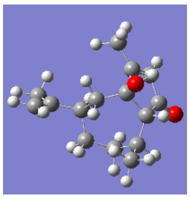
10-5

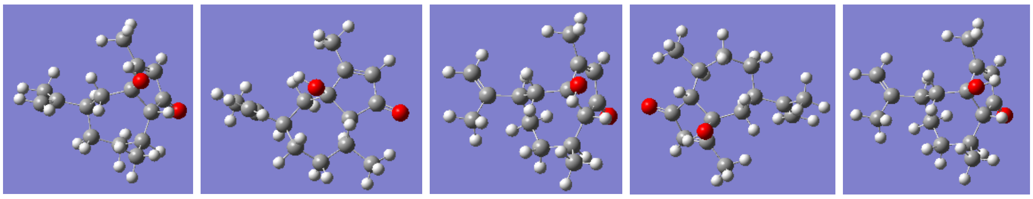

10-6 **10-7** **10-8** **10-9** **10-10**

Figure S15. B3lyp/6-31g(d) optimized low-energy conformers of **10**.

S16. Calculation of ECD Spectra for **11**.

In general, the conformations of the model compounds were searched through the molecular Merck force field 94 (MMFF94) force field with an energy cutoff of 5.0 kcal/mol using CONFLEX software. The results showed seventy lowest energy conformers for both compounds. Subsequently, the searched results were optimized with b3lyp/6-31g(d) methods in methanol using the polarizable conductor calculation model (SMD) by the Computing Center High performance computing server, Shanghai Institute of Ceramics, Chinese Academy of Science. And ECD calculated with m062x/def2tzvp in methanol by the Computing Center High performance computing server. Furthermore, averaged the simulated spectra of the conformers according to the Boltzmann distribution theory and their relative Gibbs free energy (ΔG) to obtain the final spectrum [1,2]. Ultimately, the absolute configuration of the chiral center was determined to be by comparing the experiment spectra with the calculated ECD spectra using SpecDis V1.70.1 and Origin 2018 programs.

Table S16. Energy Analysis for the Conformers of **11**.

Conformers	Relative energy (kcal/mol)	Boltzmann distribution (%)	Single point energy (a.u.)
00000002(11a-1)	0.002533	22.41	-886.9449088
00000019(11a-2)	0.001210	15.95	-886.9462325
00000016(11a-3)	0.001991	11.41	-886.9454514
00000025(11a-4)	0.000590	8.86	-886.9468521
00000066(11a-5)	0.004380	5.32	-886.9430626
00000142(11a-6)	0.004234	4.46	-886.9432082
00000004(11a-7)	0.004250	3.54	-886.9431926
00000026(11a-8)	0.001574	2.25	-886.9458681
00000034(11a-9)	0.003627	1.84	-886.9438153
00000005(11a-10)	0.005104	1.77	-886.9423382
00000141(11a-11)	0.003494	1.66	-886.9439484
00000122(11a-12)	0.000000	1.43	-886.9474421
00000110(11a-13)	0.001579	1.36	-886.9458631
00000029(11a-14)	0.000981	1.25	-886.9464612

00000020(11a-15)	0.002051	1.16	-886.9453907
00000003(11a-16)	0.004365	1.15	-886.9430767
00000128(11a-17)	0.001045	0.99	-886.9463974
00000017(11a-18)	0.003037	0.95	-886.9444050
00000086(11a-19)	0.001196	0.90	-886.9462460
00000037(11a-20)	0.001461	0.72	-886.9459807
00000008(11a-21)	0.004195	0.70	-886.9432472
00000028(11a-22)	0.000749	0.69	-886.9466929
00000193(11a-23)	0.005664	0.68	-886.9417782
00000135(11a-24)	0.001488	0.62	-886.9459544
00000036(11a-25)	0.001488	0.49	-886.9459541
00000149(11a-26)	0.003709	0.46	-886.9437332
00000060(11a-27)	0.001537	0.45	-886.9459050
00000044(11a-28)	0.000497	0.37	-886.9469448
00000134(11a-29)	0.002005	0.36	-886.9454371
00000051(11a-30)	0.003690	0.36	-886.9437523
00000075(11a-31)	0.003843	0.33	-886.9435995
00000041(11a-32)	0.005082	0.33	-886.9423603
00000117(11a-33)	0.002525	0.33	-886.9449166
00000133(11a-34)	0.004911	0.29	-886.9425309
00000001(11a-35)	0.005074	0.26	-886.9423682
00000109(11a-36)	0.000904	0.25	-886.9465381
00000082(11a-37)	0.004531	0.23	-886.9429115
00000059(11a-38)	0.001393	0.20	-886.9460492
00000132(11a-39)	0.004700	0.19	-886.9427422
00000074(11a-40)	0.003619	0.18	-886.9438230
00000050(11a-41)	0.003644	0.18	-886.9437978
00000057(11a-42)	0.005928	0.17	-886.9415142
00000154(11a-43)	0.002933	0.16	-886.9445088
00000024(11a-44)	0.005305	0.16	-886.9421371
00000081(11a-45)	0.004466	0.15	-886.9429758
00000140(11a-46)	0.003414	0.15	-886.9440278
00000127(11a-47)	0.001067	0.14	-886.9463755
00000236(11a-48)	0.004101	0.14	-886.9433413
00000023(11a-49)	0.006334	0.12	-886.9411081
00000091(11a-50)	0.003705	0.11	-886.9437372
00000198(11a-51)	0.005658	0.10	-886.9417838
00000069(11a-52)	0.003111	0.09	-886.9443315
00000115(11a-53)	0.004119	0.09	-886.9433230
00000068(11a-54)	0.002853	0.08	-886.9445896
00000157(11a-55)	0.003299	0.08	-886.9441427
00000056(11a-56)	0.006217	0.08	-886.9412249
00000092(11a-57)	0.002519	0.06	-886.9449233
00000101(11a-58)	0.002527	0.06	-886.9449155
00000040(11a-59)	0.005489	0.05	-886.9419534
00000200(11a-60)	0.005753	0.05	-886.9416895
00000126(11a-61)	0.004915	0.05	-886.9425268
00000070(11a-62)	0.006439	0.04	-886.9410029

00000162(11a-63)	0.005772	0.04	-886.9416704
00000096(11a-64)	0.003516	0.04	-886.9439260
00000107(11a-65)	0.003917	0.03	-886.9435254
00000208(11a-66)	0.003082	0.03	-886.9443604
00000009(11a-67)	0.004432	0.03	-886.9430103
00000032(11a-68)	0.005484	0.02	-886.9419585
00000007(11a-69)	0.003504	0.02	-886.9439385
00000010(11a-70)	0.004556	0.02	-886.9428860
00000029(11c-1)	0.000898	11.52	-886.9447314
00000004(11c-2)	0.001001	10.68	-886.9446283
00000003(11c-3)	0.002589	9.73	-886.9430407
00000001(11c-4)	0.001695	8.19	-886.9439341
00000092(11c-5)	0.001228	6.46	-886.9444015
00000015(11c-6)	0.001755	6.02	-886.9438749
00000100(11c-7)	0.000845	4.89	-886.9447847
00000049(11c-8)	0.000000	4.43	-886.9456295
00000135(11c-9)	0.002370	4.16	-886.9432596
00000136(11c-10)	0.002049	3.85	-886.9435801
00000022(11c-11)	0.001995	3.29	-886.9436345
00000039(11c-12)	0.000134	2.58	-886.9454952
00000021(11c-13)	0.002698	2.07	-886.9429313
00000101(11c-14)	0.000423	1.87	-886.9452065
00000002(11c-15)	0.002474	1.87	-886.9431559
00000016(11c-16)	0.002459	1.62	-886.9431704
00000062(11c-17)	0.000100	1.44	-886.9455297
00000095(11c-18)	0.001545	1.40	-886.9440847
00000056(11c-19)	0.001617	1.39	-886.9440123
00000014(11c-20)	0.001991	1.33	-886.9436387
00000059(11c-21)	0.001676	1.06	-886.9439539
00000017(11c-22)	0.000637	0.95	-886.9449930
00000009(11c-23)	0.001250	0.88	-886.9443797
00000064(11c-24)	0.000710	0.78	-886.9449195
00000007(11c-25)	0.003237	0.59	-886.9423923
00000005(11c-26)	0.002142	0.53	-886.9434876
00000024(11c-27)	0.001834	0.48	-886.9437954
00000018(11c-28)	0.001030	0.45	-886.9445998
00000025(11c-29)	0.002509	0.44	-886.9431202
00000031(11c-30)	0.000967	0.40	-886.9446621
00000085(11c-31)	0.001317	0.33	-886.9443121
00000038(11c-32)	0.001988	0.32	-886.9436419
00000094(11c-33)	0.000868	0.32	-886.9447617
00000010(11c-34)	0.001634	0.28	-886.9439955
00000148(11c-35)	0.001970	0.27	-886.9436594
00000032(11c-36)	0.001673	0.25	-886.9439562
00000057(11c-37)	0.000712	0.22	-886.9449177
00000109(11c-38)	0.001572	0.19	-886.9440573
00000070(11c-39)	0.002279	0.18	-886.9433501
00000044(11c-40)	0.003539	0.17	-886.9420900

00000061(11c-41)	0.001387	0.15	-886.9442421
00000045(11c-42)	0.003715	0.15	-886.9419140
00000099(11c-43)	0.002179	0.15	-886.9434502
00000075(11c-44)	0.003552	0.15	-886.9420776
00000077(11c-45)	0.002087	0.12	-886.9435425
00000087(11c-46)	0.001342	0.10	-886.9442878
00000037(11c-47)	0.002891	0.09	-886.9427383
00000115(11c-48)	0.001408	0.09	-886.9442218
00000130(11c-49)	0.001616	0.09	-886.9440139
00000071(11c-50)	0.002855	0.08	-886.9427744
00000116(11c-51)	0.001077	0.08	-886.9445529
00000153(11c-52)	0.001731	0.07	-886.9438984
00000048(11c-53)	0.001586	0.07	-886.9440433
00000131(11c-54)	0.002120	0.06	-886.9435095
00000183(11c-55)	0.002527	0.05	-886.9431022
00000142(11c-56)	0.002378	0.05	-886.9432517
00000013(11c-57)	0.005045	0.05	-886.9405847
00000102(11c-58)	0.002054	0.04	-886.9435753
00000132(11c-59)	0.005110	0.04	-886.9405192
00000093(11c-60)	0.002207	0.04	-886.9434223
00000043(11c-61)	0.003643	0.03	-886.9419867
00000072(11c-62)	0.005879	0.03	-886.9397501
00000219(11c-63)	0.001684	0.03	-886.9439460
00000200(11c-64)	0.001455	0.02	-886.9441748
00000141(11c-65)	0.004250	0.02	-886.9413791
00000078(11c-66)	0.005604	0.02	-886.9400254
00000227(11c-67)	0.003986	0.02	-886.9416436
00000107(11c-68)	0.005281	0.02	-886.9403482
00000035(11c-69)	0.005055	0.02	-886.9405745
00000108(11c-70)	0.002100	0.01	-886.9435292

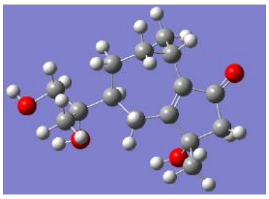

11a-1

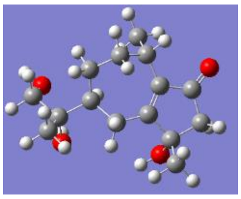

11a-2

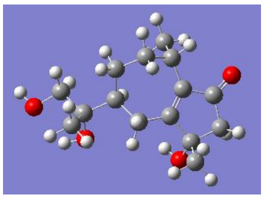

11a-3

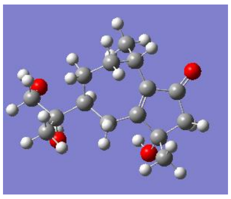

11a-4

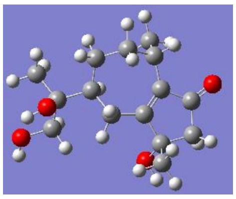

11a-5

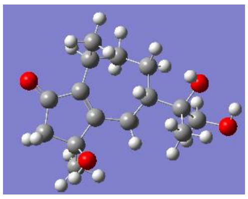

11a-6

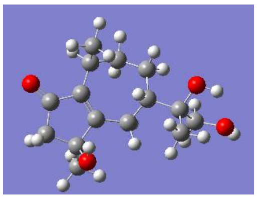

11a-7

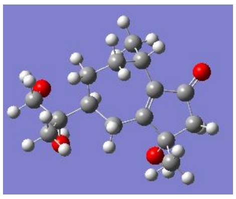

11a-8

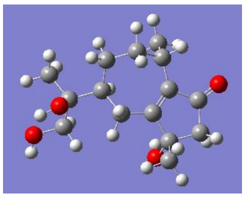
11a-9
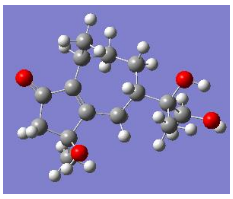
11a-10
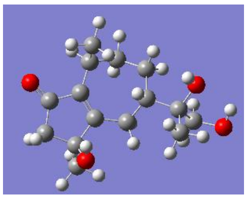
11a-11
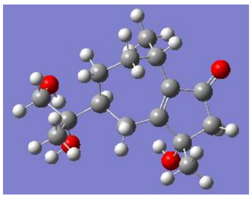
11a-12
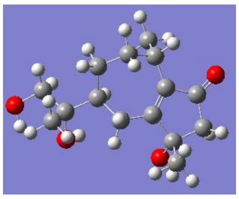
11a-13
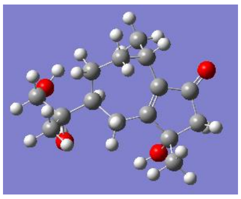
11a-14
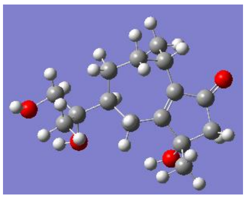
11a-15
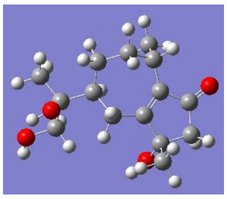
11a-16
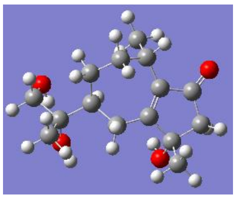
11a-17
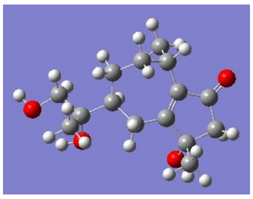
11a-18
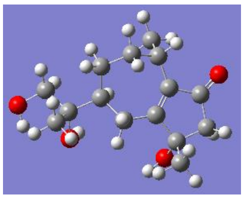
11a-19
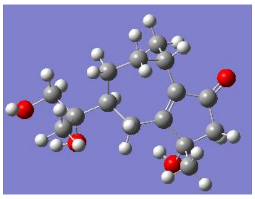
28a -20
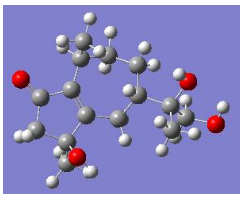
11a-21
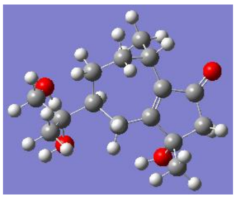
11a-22
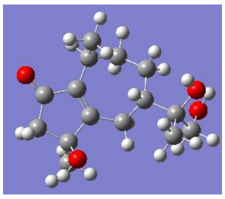
11a-23
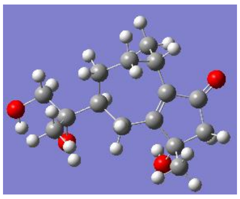
11a-24
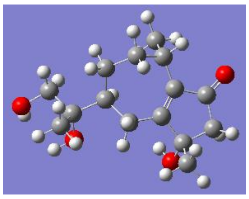
11a-25
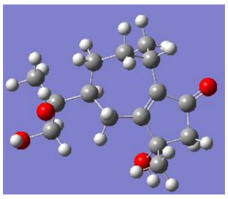
11a-26
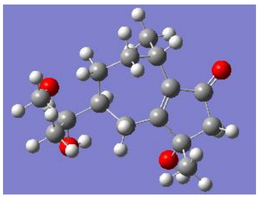
11a-27
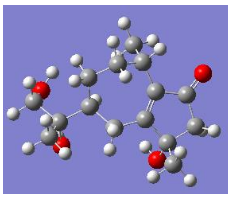
11a-28
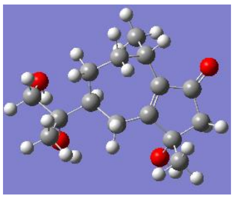
11a-29
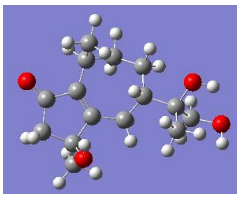
11a-30
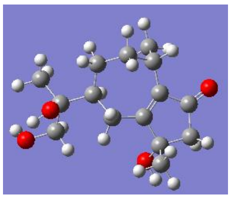
11a-31
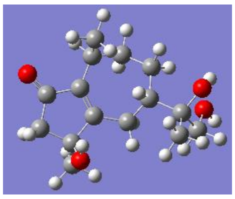
11a-32
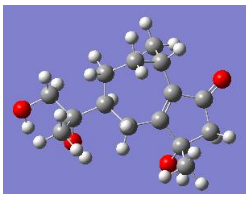
11a-33
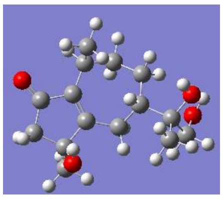
11a-34
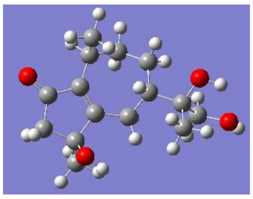
11a-35
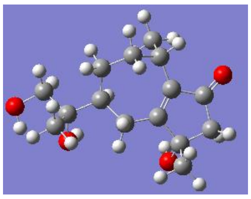
11a-36

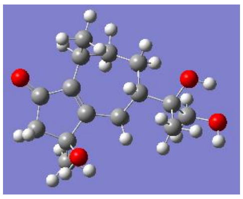

11a-37

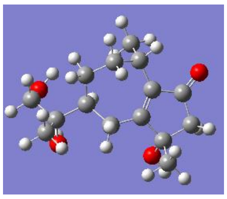

11a-38

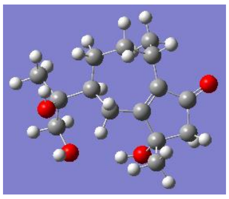

11a-39

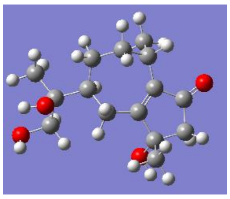

11a-40

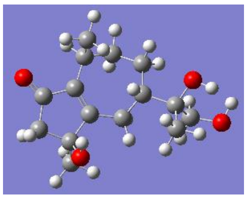

11a-41

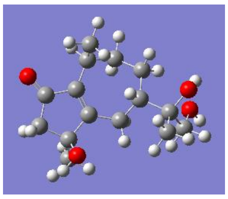

11a-42

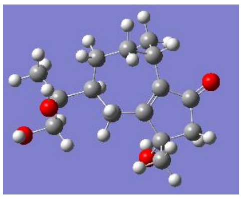

11a-43

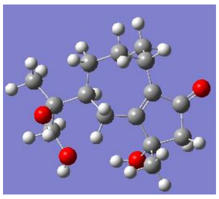

11a-44

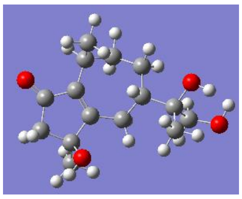

11a-45

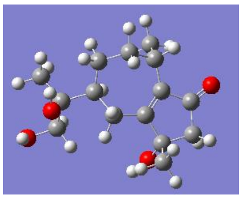

11a-46

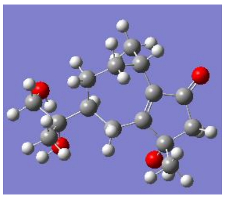

11a-47

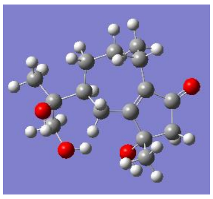

11a-48

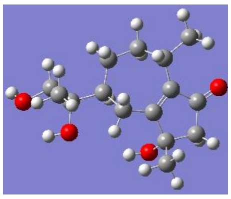

11a-49

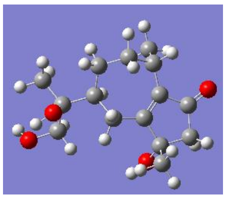

11a-50

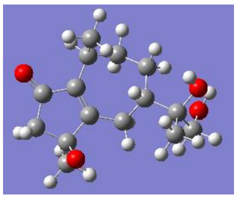

11a-51

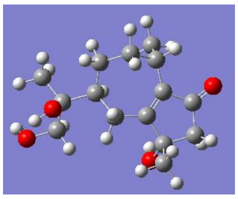

11a-52

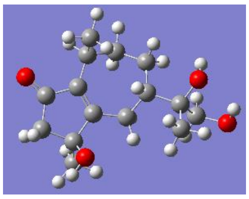

11a-53

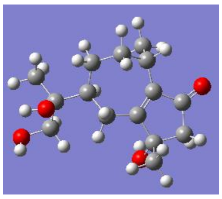

11a-54

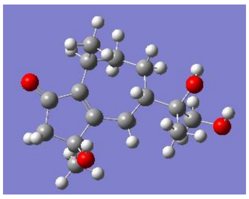

11a-55

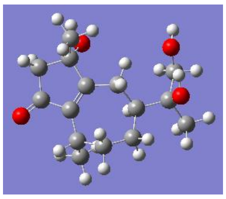

11a-56

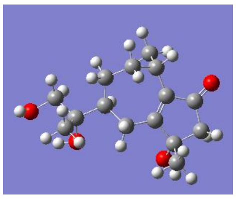

11a-57

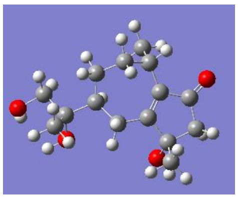

11a-58

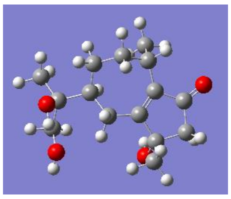

11a-59

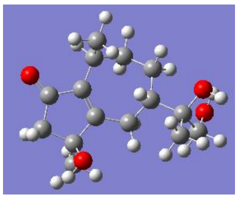

11a-60

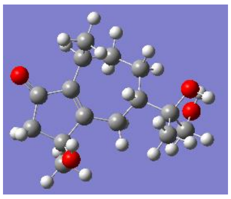

11a-61

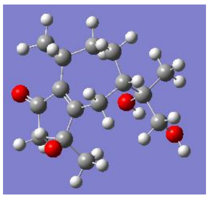

11a-62

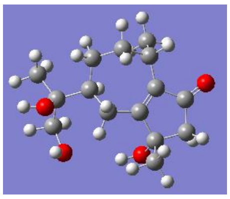

11a-63

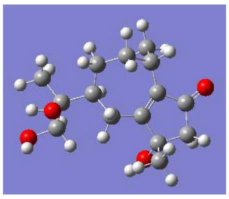

11a-64

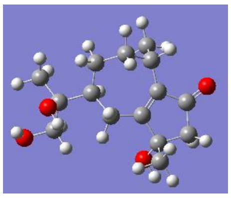

11a-65

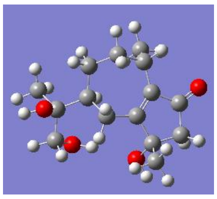

11a-66

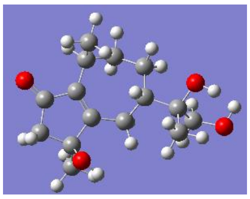

11a-67

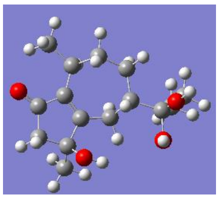

11a-68

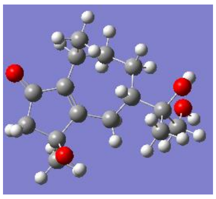

11a-69

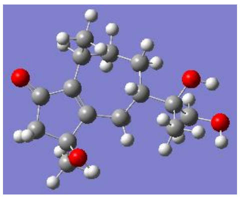

11a-70

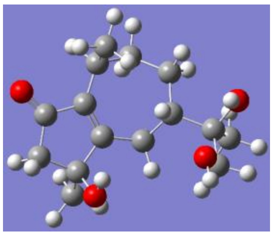

11c-1

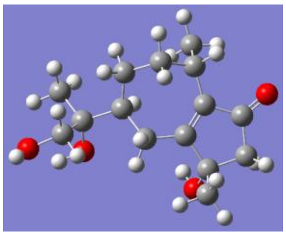

11c-2

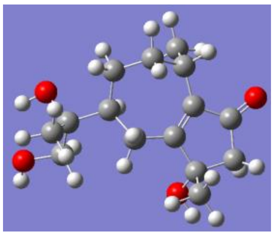

11c-3

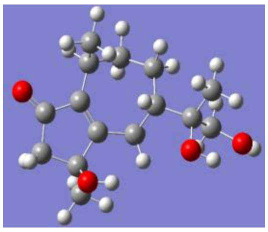

11c-4

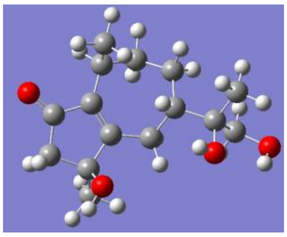

11c-5

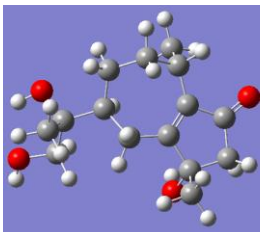

11c-6

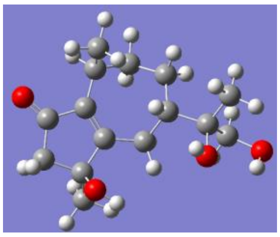

11c-7

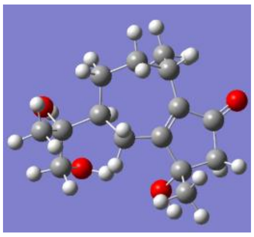

11c-8

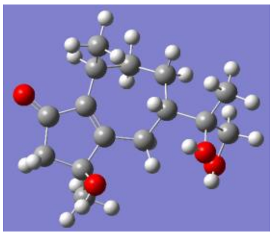

11c-9

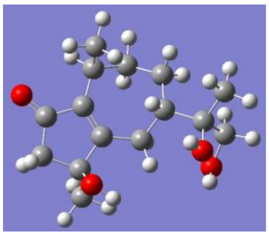

11c-10

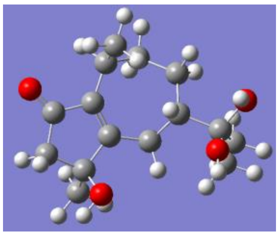

11c-11

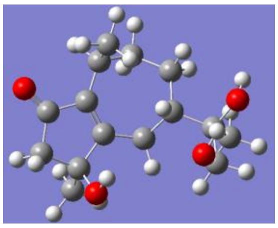

11c-12

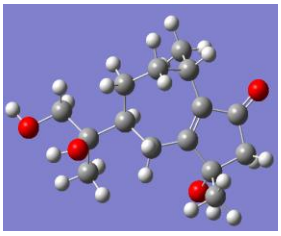

11c-13

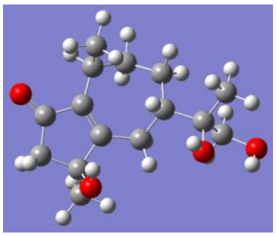

11c-14

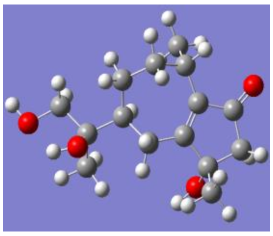

11c-15

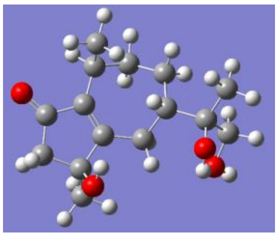

11c-16

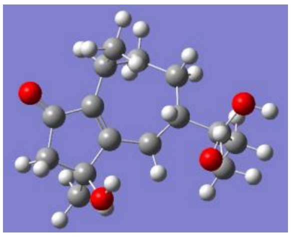

11c-17

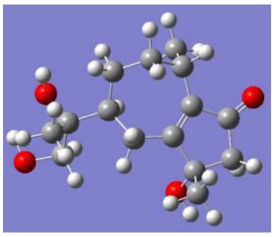

11c-18

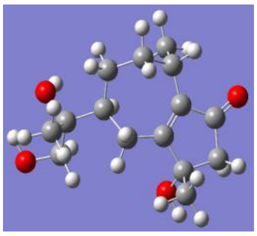

11c-19

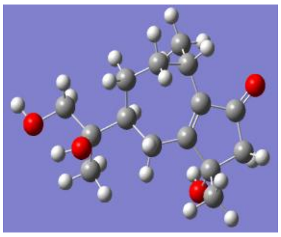

11c-20

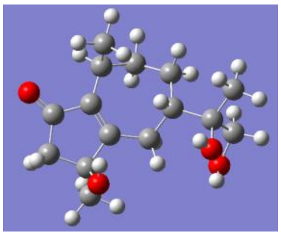

11c-21

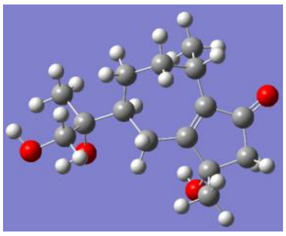

11c-22

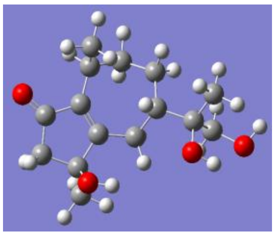

11c-23

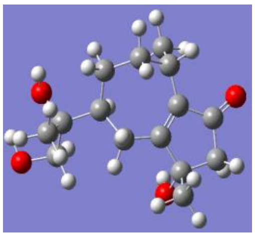

11c-24

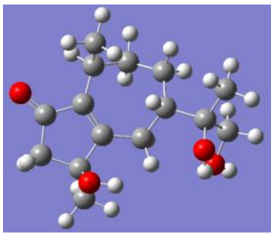

11c-25

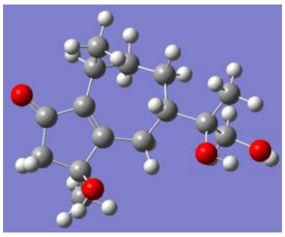

11c-26

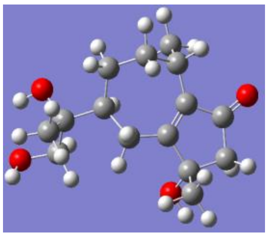

11c-27

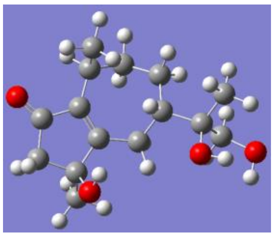

11c-28

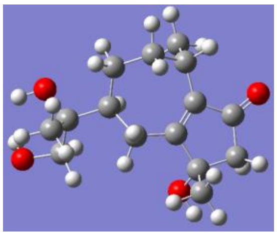

11c-29

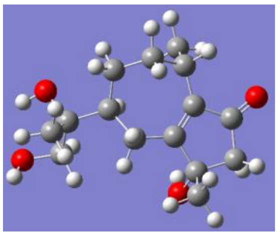

11c-30

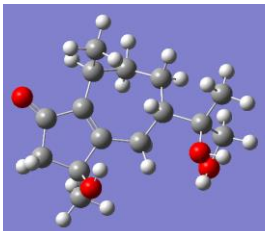

11c-31

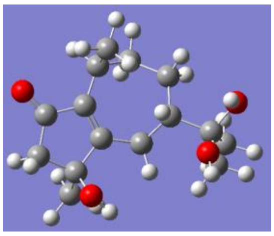

11c-32

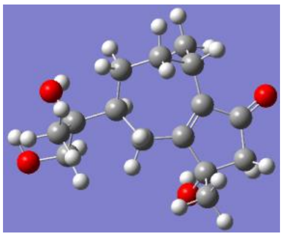

11c-33

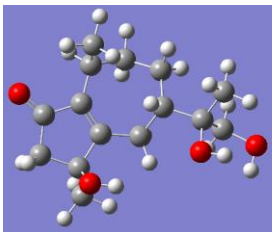

11c-34

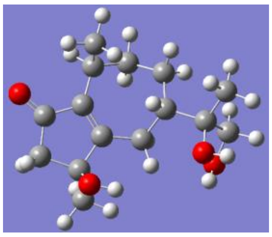

11c-35

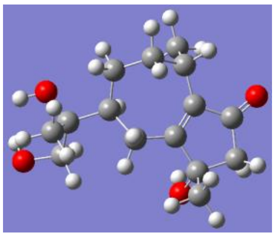

11c-36

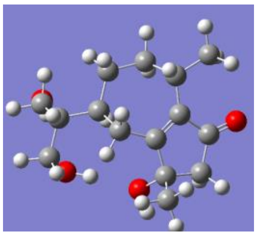

11c-37

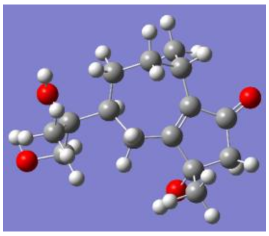

11c-38

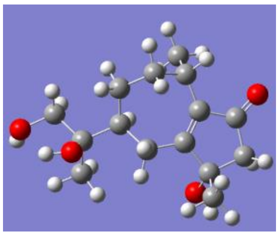

11c-39

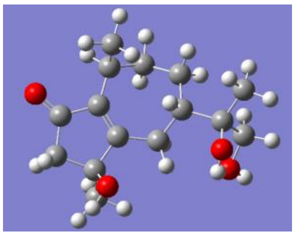

11c-40

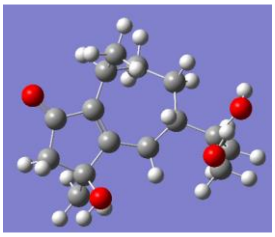

11c-41

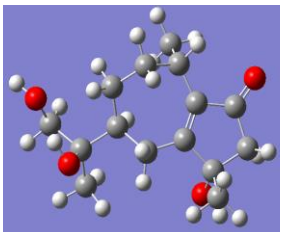

11c-42

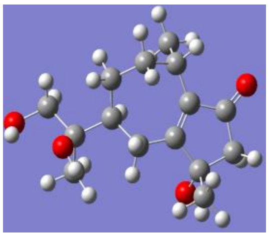

11c-43

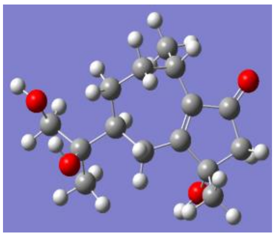

11c-44

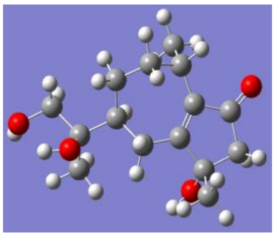

11c-45

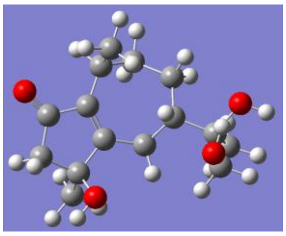

11c-46

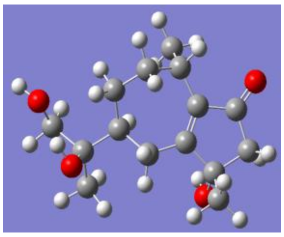

11c-47

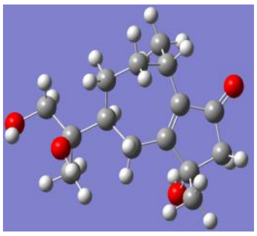

11c-48

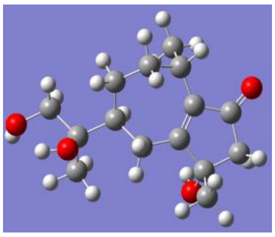

11c-49

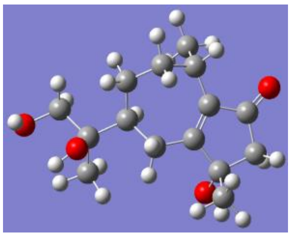

11c-50

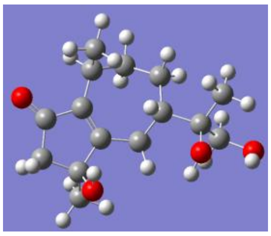

11c-51

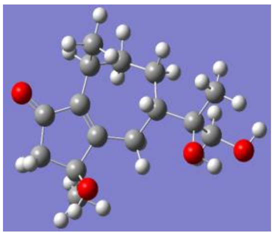

11c-52

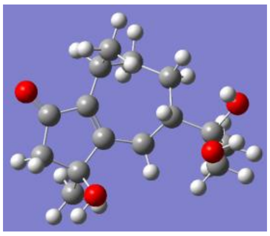

11c-53

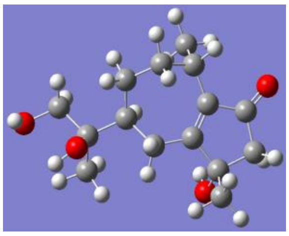

11c-54

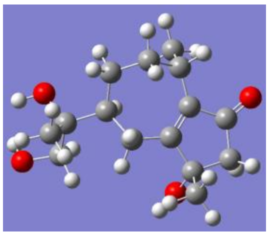

11c-55

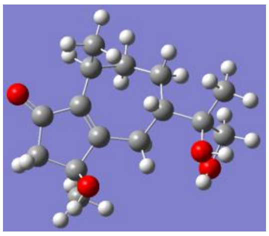

11c-56

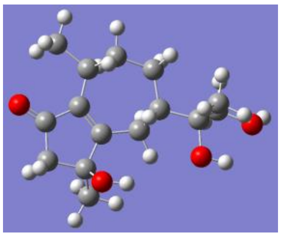

11c-57

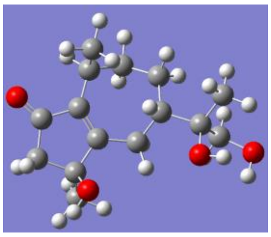

11c-58

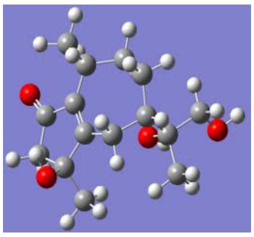

11c-59

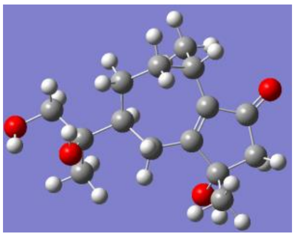

11c-60

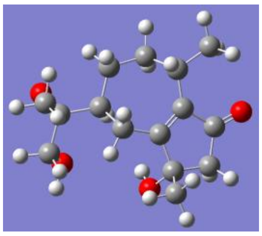

11c-61

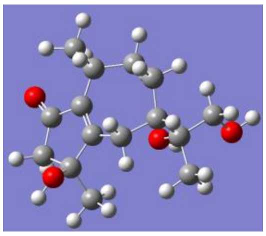

11c-62

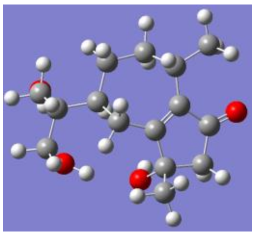

11c-63

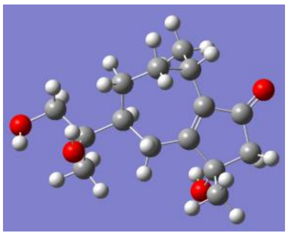

11c-64

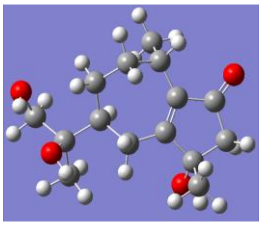 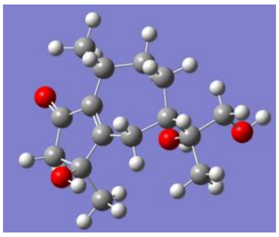 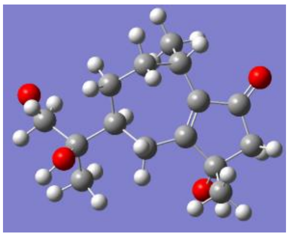 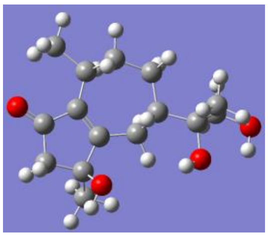

11c-65 **11c**-66 **11c**-67 **11c**-68

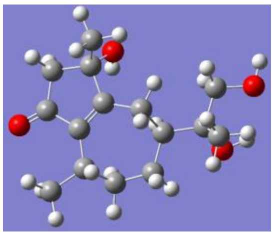 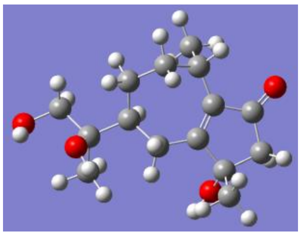

11c-69 **11c**-70

Figure S16. B3lyp/6-31g(d) optimized low-energy conformers of **11**.

S17. Calculation of ECD Spectra for **12**.

In general, the conformations of the model compounds were searched through the molecular Merck force field 94 (MMFF94) force field with an energy cutoff of 5.0 kcal/mol using CONFLEX software. The results showed twenty-four lowest energy conformers for both compounds. Subsequently, the searched results were optimized with m062x/6-311g(d,p) methods in methanol using the polarizable conductor calculation model (SMD) by the Computing Center High performance computing server, Shanghai Institute of Ceramics, Chinese Academy of Science. And ECD calculated with m062x/def2tzvp in methanol by the Computing Center High performance computing server. Furthermore, averaged the simulated spectra of the conformers according to the Boltzmann distribution theory and their relative Gibbs free energy (ΔG) to obtain the final spectrum [1,2]. Ultimately, the absolute configuration of the chiral center was determined to be by comparing the experiment spectra with the calculated ECD spectra using SpecDis V1.70.1 and Origin 2018 programs.

Table S17. Energy Analysis for the Conformers of **12**.

Conformers	Relative energy (kcal/mol)	Boltzmann distribution (%)	Single point energy (a.u.)
00000025(12-1)	0.003383	13.03	-736.4526486
00000008(12-2)	0.001009	12.29	-736.4550221
00000012(12-3)	0.000745	12.21	-736.4552864

00000011(12-4)	0.000222	12.01	-736.4558092
00000018(12-5)	0.000000	11.97	-736.4560314
00000028(12-6)	0.002849	7.79	-736.4531826
00000016(12-7)	0.003969	7.62	-736.4520627
00000024(12-8)	0.001409	5.39	-736.4546227
00000007(12-9)	0.002220	5.28	-736.4538116
00000022(12-10)	0.003365	4.28	-736.4526666
00000014(12-11)	0.003890	3.42	-736.4521412
00000020(12-12)	0.003357	1.92	-736.4526745
00000029(12-13)	0.004100	1.38	-736.4519316
00000033(12-14)	0.004692	0.72	-736.4513399
00000038(12-15)	0.004664	0.34	-736.4513669
00000031(12-16)	0.006528	0.09	-736.4495037
00000030(12-17)	0.006430	0.07	-736.4496014
00000043(12-18)	0.005863	0.04	-736.4501687
00000041(12-19)	0.006851	0.03	-736.4491804
00000006(12-20)	0.006806	0.03	-736.4492251
00000042(12-21)	0.006845	0.03	-736.4491864
00000035(12-22)	0.006761	0.02	-736.4492706
00000045(12-23)	0.006160	0.01	-736.4498716
00000047(12-24)	0.006245	0.01	-736.4497866

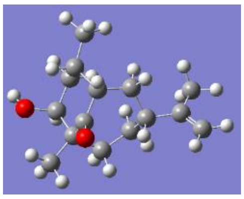
12-1

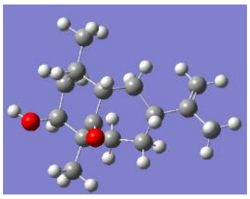
12-2

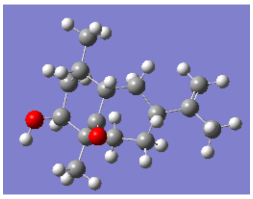
12-3

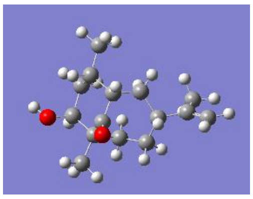
12-4

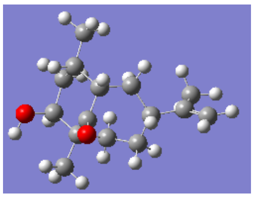
12-5

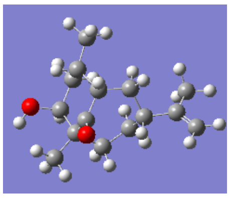
12-6

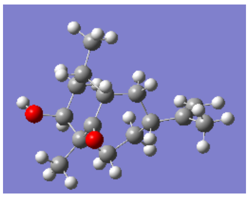
12-7

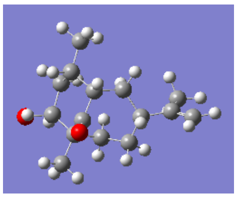
12-8

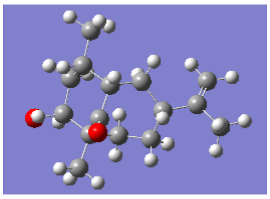
12-9

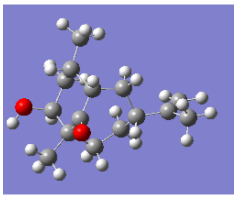
12-10

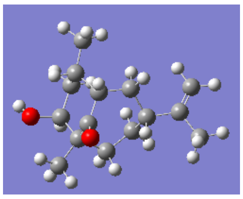
12-11

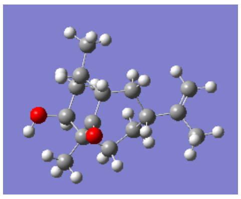
12-12

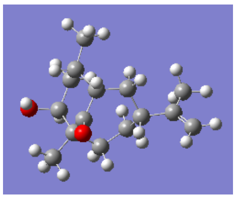
12-13

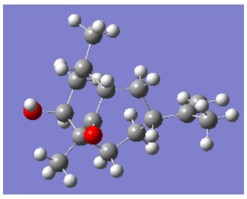
12-14

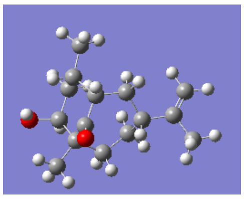
12-15

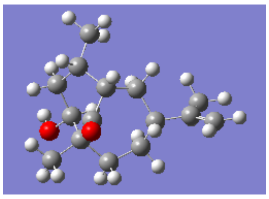
12-16

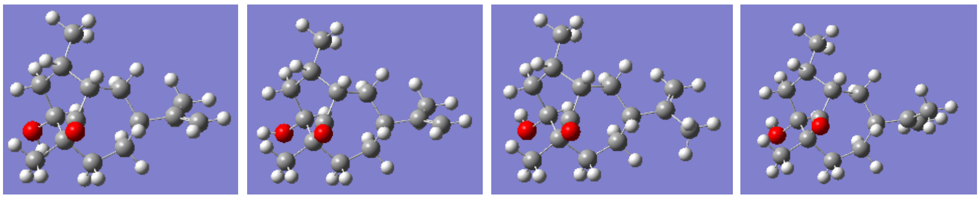

12-17 **12**-18 **12**-19 **12**-20

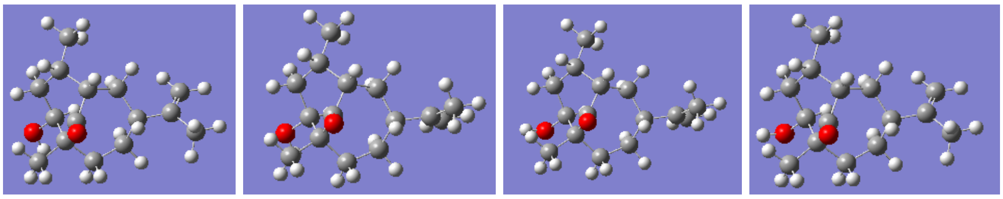

12-21 **12**-22 **12**-23 **12**-24

Figure S17. M062x/6-311g(d,p) optimized low-energy conformers of **12**.

S18. UPLC-Q-TOF-MS/MS results of *S. officinalis*

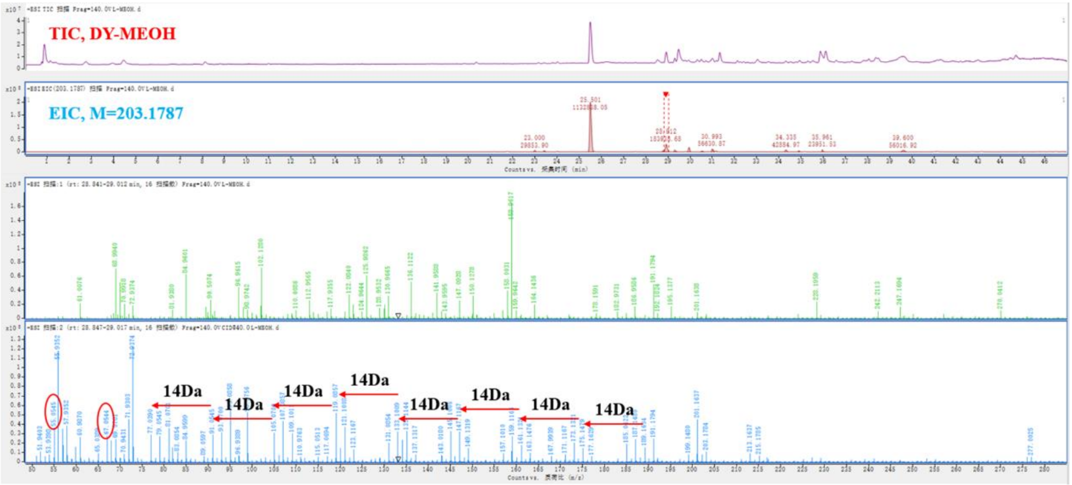

Figure S18. UPLC-Q-TOF-MS/MS results of *S. officinalis*

(a) Total ion chromatogram (TIC) of *S. officinalis* in positive ion mode; (b) Extracted ion chromatogram (EIC) of *m/z* 203.1787; (c) First-order mass spectrum at EIC *m/z* 203.1787; (d) Secondary ion mass spectrum at EIC *m/z* 203.1787.

Table S18. Elemental constituents of major product ions for patchoulol-type sesquiterpenoids (compounds **3−5**, **14**, **15** and **19**).

**Comp.**	**Molecular formula**	**Molecular weight (*m/z*)**	**Observed mass (*m/z*)**	**Quasi-molecular Ion**	**Calculated mass (*m/z*)**	**Error (ppm)**	**MS^1 fragments**	**Major MS^2 fragments**
**3**	$C_{15}H_{26}O_3$	254.1882	219.1737	$[M-2H_2O+H]^+$	219.1743	2.74	237.1835, 219.1737, 201.1619	147.1142, 133.0995, 119.0838, 105.0685, 91.0530, 77.0374,

								67.0532, 55.0533, 53.0381
**4**	$C_{15}H_{26}O_3$	254.1882	201.1634	$[M-3H_2O+H]^+$	201.1638	1.99	237.1824, 219.1735, 201.1634	147.1133, 133.1002, 119.0854, 105.0701, 91.0545, 77.0388, 67.0544, 55.0544, 53.0387
**5**	$C_{14}H_{24}O_2$	224.1776	207.1743	$[M-H_2O+H]^+$	207.1743	0	207.1743, 189.1632	147.1161, 133.1009, 119.0851, 105.0694, 91.0538, 77.0383, 67.0540, 63.0226, 55.0540, 53.0387
**14**	$C_{15}H_{26}O_2$	238.1933	203.1787	$[M-2H_2O+H]^+$	203.1794	3.45	221.1896, 203.1787	147.1157, 133.1004, 119.0854, 105.0694, 91.0540, 77.0386, 67.0543, 63.0228, 55.0544, 53.0389
**15**	$C_{15}H_{26}O_2$	238.1933	221.1898	$[M-H_2O+H]^+$	221.19	0.9	221.1898, 203.1796	147.1165, 133.1006, 119.0852, 105.0696, 91.0541, 77.0387, 67.0543, 63.0231, 55.0543, 53.0389
**19**	$C_{15}H_{26}O_2$	238.1933	203.1776	$[M-2H_2O+H]^+$	203.1794	8.86	221.1880, 203.1776	147.1145, 133.1052, 119.0841, 105.0683, 91.0528, 77.0374, 67.0531, 63.0220, 55.0532, 53.0378

S19. Inhibition rate of melanin production in B16F10 cells

Table S19. Melanogenesis levels of compounds **1−37** in IBMX-stimulated B16F10

melanoma cells.

Compounds[a]	melanogenesis level (%)[c]	Compounds	melanogenesis level (%)	Compounds	melanogenesis level (%)
HQ[b]	57 ± 6.0**	**13**	47 ± 27.6**	**26**	76 ± 3.0**
**1**	81 ± 4.8	**14**	93 ± 19.4**	**27**	51 ± 9.3**
**2**	79 ± 1.3**	**15**	-14 ± 18.3	**28**	53 ± 6.3**
**3**	59 ± 1.8**	**16**	64 ± 4.4**	**29**	110 ± 2.2**
**4**	60 ± 7.8**	**17**	40 ± 23.5*	**30**	65 ± 7.3**
**5**	47 ± 12.4**	**18**	-8 ± 24.9	**31**	36 ± 13.2*
**6**	27 ± 16.6**	**19**	43 ± 10.9**	**32**	41 ± 13.4*
**7**	58 ± 2.4	**20**	39 ± 11.2	**33**	59 ± 18**
**8**	65 ± 4.3**	**21**	68 ± 12.8**	**34**	68 ± 3.6**
**9**	62 ± 9.5**	**22**	92 ± 2.4**	**35**	71 ± 7.7**
**10**	47 ± 14.1**	**23**	73 ± 3.1**	**36**	72 ± 10.2**
**11**	40 ± 6.2**	**24**	-12 ± 15.0	**37**	32 ± 6.0
**12**	81 ± 4.8*	**25**	82 ± 1.1**		

[a] Except for compound **5** with a concentration of 6.25 μM and **27** with 25 μM, all other compounds have a concentration of 50 μM.

[b] Positive control, hydroquinone (HQ, 10 μM).

[c] *p < 0.05, **p < 0.01 compared with the model group (0%). Values are presented as the mean ± SEM (n = 3).

S20. Results of B16F10 cells viability assay

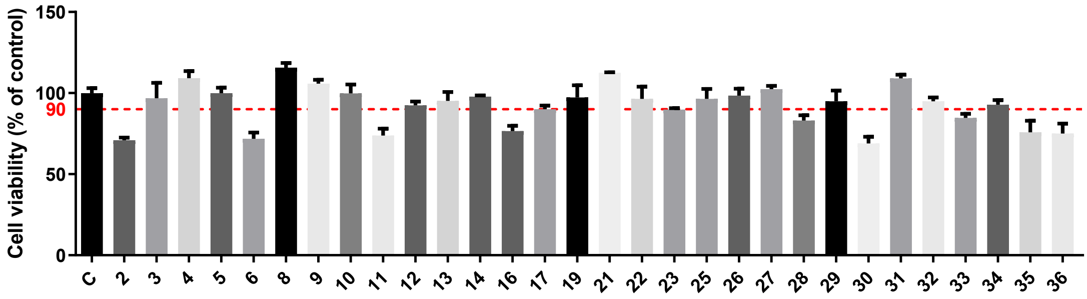

Figure S20. The compounds **2−6**, **8−14**, **16**, **17**, **19**, **21−23** and **25−36** were treated at 50 μM for 48 h (except for compound **5** at 6.25 μM and **27** at 25 μM) on B16F10

cells, the cytotoxicity was calculated compared with control group by CCK-8 assay.

S21. General experimental procedures

Optical rotations were performed on Rudolph Autopol VI automatic polarimeter with MeOH (Rudolph, USA). Electronic circular dichroism (ECD) spectra were recorded on an Applied photophysics brighttime chirascan spectrometer (Applied Photophysics, UK). X-ray crystallographic data were recorded on a Bruker Apex II CCD diffractometer (Bruker, Germany) using Cu–Kα radiation (λ = 1.54178 Å). 1D and 2D NMR spectra were measured on Bruker-Avance 600 instrument (Bruker, Germany) and chemical shifts were given as δ (ppm) with reference to the solvent peaks as CD_3OD (δ_H 3.31, δ_C 49.0). A Q–TOF–Ultima mass spectrometer (Agilent Milford, MA, USA) was used to measure the HRESIMS data. Semi-preparative HPLC was carried out on an Agilent 1260 system (Agilent, USA) equipped with a DAD detector using an Agilent Zorbax SB–C_{18} column (250 mm × 9.4 mm, 5 μm, USA) or a YMC–Pack ODS–A C_{18} column (250 mm × 10 mm, 5 μm, Japan). Column chromatography (CC) was performed using Reversed-phase (RP) C_{18} silica gel (50 μm, YMC Co., Ltd. Japan), silica gel (100–200, 200–300 and 300–400 mesh, Qingdao Haiyang Chemical, China), Sephadex LH-20 (40–70 μm, GE Healthcare, Bio-Sciences AB, Sweden), Microporous (MCI) resin gel (CHP–20 P, Mitsubishi Chemical Industries Co., Ltd., Japan), polyamide resin (30–60 and 60–90 mesh) and macroporous resin (D101 type, Sinopharm Chemical Reagent Co., Ltd., China). On silica gel HSGF254 plates (20 × 20 cm; Yantai Jiangyou Silicone Development Co., Ltd., China), thin layer chromatography (TLC) analysis and preparative TLC isolation were carried out. All CC-applied chemicals were analytically appropriate reagents purchased from Sinopharm Chemical Reagent Co., Ltd., China.

S22. Extraction and isolation of the known compounds **13−37**

The dried roots of *S. officinalis* (20 kg) were powdered and subjected to reflux extraction with 95% EtOH at 85 °C for three times (200 L, 1.5 h each). After concentration, a crude residue (6.0 kg) was produced. The following was then suspended in water (20 L) and subsequently partitioned with PE (3 × 20.0 L) and EA (3 × 20.0 L) to obtain EA-soluble fractions.

The EA fraction (2578 g) was dispersed in water (5.0 L) and then processed on a 25.0 L Diaion 101 macroporous resin CC eluted with 0%, 20%, 40%, 60%, 80% and 95% EtOH and detected by TLC to yield six fractions (Fr. I–VI). Fr. II (978 g) was chromatographed on a polyamide CC (100 × 22 cm, 10.0 kg) eluting with 0%, 20%, 40%, 60%, 80% and 95% EtOH to afford six major fractions (Fr. IIA–F).

Fr.IIA (369 g) was separated on a silica gel (200–300 mesh) column and using a gradual slope of CH_2Cl_2–MeOH (200:1 to 1:5) to give ten fractions (Fr.IIA1–10). Fr.IIA3 (3120 mg) was conducted on an ODC CC (MeOH–H_2O, 10:90 to 100:0) to yield seventeen parts (Fr.IIA3A–Q). Fr.IIA3L (40 mg) was chromatographed on Sephadex LH-20 (MeOH–H_2O, 70:30), affording compound **16** (14 mg). Fr.IIA7 (5560 mg) was conducted on a silica gel CC, eluting CH_2Cl_2–MeOH (100:1 to 5:1) to obtain seven fractions (Fr.IIA7A–G). Fr.IIA7G (2094 mg) was subjected to ODC CC (MeOH–H_2O, 20:80 to 100:0) to afford twelve fractions (Fr.IIA7G-1–12). Fr.IIA7G-8 (121 mg) was conducted on a Sephadex LH-20 (MeOH–H_2O, 70:30), and subsequently refined using semi-preparative HPLC (CH_3CN–H_2O, 17:83, 3.0 mL/min) to produce compound **20** (11 mg, t_R = 18.50 min).

Fr. IIB (69 g) was chromatographically separated using a silica gel (55 cm × 10 cm) and produced nine fractions (Fr.IIB1–9) by eluting using a CH_2Cl_2–MeOH gradient system (500:1 to 5:1, v/v). Fr.IIB1 (3.527 g), Fr.IIB2 (0.280 g) and Fr.IIB3 (0.835 g) were isolated utilizing Sephadex LH-20 CC using PE–CH_2Cl_2–MeOH isocratic system (5:5:1) to obtain the nine sub-fractions Fr.IIB1A–C, Fr.IIB2A–C and Fr.IIB3A–C. Fr.IIB1B&2B (233 mg) was separated by MCI CC using MeOH–H_2O (40:60 to 100:0, v/v) to yield compound **14** (26 mg) and Fr.IIB1B&2B-1. Fr.IIB1B&2B-1 (145 mg) was chromatographed on a silica gel CC and using

cyclohexane (CYH)–EA (50:1 to 2:1, v/v), and subsequently purified by ODS CC eluted with MeOH–H_2O (70:30, v/v) to give compounds **15** (62 mg) and **19** (9 mg). Fr.IIB1A (3314 mg) was isolated on a silica gel CC using step CYH–EA mixed-solvent system (100:1 to 1:1, v/v) to give ten fractions (Fr.IIB1A-1–10). Fr.IIB1A–5 (122 mg) was used ODS CC with a shifty solvent system of MeOH–H_2O (30:70 to 100:0, v/v) to attain seven fractions (Fr.IIB1A-5a–g). Fr.IIB1A-5d (40 mg) was conducted on a prep-TLC and developed with CH_2Cl_2-MeOH (20:1) and further refined by semi-prep-HPLC (MeOH–H_2O, 45:55, 3.0 mL/min) to yield compound **18** (12 mg, t_R = 18.59 min). Fr.IIB1A–6 (81 mg) was separated via semi-prep-HPLC (CH_3CN–H_2O, 29:71, 3.0 mL/min) to gain compound **24** (8 mg, t_R = 29.09 min). Fr.IIB1A-8 (77 mg) was separated by MCI CC and using a stepwise gradient of MeOH–H_2O (40:60 to 100:0, v/v) resulting four fractions (Fr.IIB1A-8a–d). Fr.IIB1A-8c (24 mg) was separated by preparative TLC and developed with CH_2Cl_2–MeOH (20:1), afterwards further purified via semi-prep-HPLC (MeOH–H_2O, 42:58, 3.0 mL/min), yielding compound **37** (3 mg, t_R = 17.01 min). Fr.IIB1A–9 (122 mg) was separated by semi-prep-HPLC (CH_3CN–H_2O, 30:70, 3.0 mL/min) to obtain compounds **25** (4 mg, t_R = 54.42 min), **26** (6 mg, t_R = 59.13 min), **27** (8 mg, t_R = 65.68 min) and **28** (6 mg, t_R = 67.30 min). Fr.IIB3B (387 mg) was fractionated on MCI CC using MeOH–H_2O step mixed-solvent system (40:60 to 100:0) to give Fr.IIB3B-1 and Fr.IIB3B-2. Fr.IIB3B-2 (206 mg) was further fractionated on a silica gel CC eluted with CYH–EA (50:1 to 2:1) and refined by semi-preparative HPLC (CH_3CN–H_2O, 32:68, 3.0 mL/min) to yield compound **32** (3 mg, t_R = 25.49 min). Fr.IIB5 (1007 mg) was separated into twelve subfractions (Fr.IIB5A–L) after an ODS CC using a stepwise gradient of MeOH–H_2O from 20:80 to 100:0. Fr.IIB5D (63 mg) was chromatographed over preparative TLC (CH_2Cl_2–MeOH, 20:1), subsequently refined via ODS CC (MeOH–H_2O, 70:30) to isolate compound **21** (29 mg). Fr.IIB5J (167 mg) was isolated by silica gel CC eluted with CYH–EA (10:1 to 1:2) to afford four fractions (Fr.IIB5J-1–4). Fr.IIB5J-1 (13 mg) was recrystallized from MeOH for obtaining the compound **17** (5 mg). Fr.IIB5J-2 (19 mg) and Fr.IIB5J-3 (68 mg) were conducted on a Sephadex LH-20 (MeOH–H_2O, 70:30) separate into **29** (6 mg) and **13**

(66 mg), respectively. Fr.IIB7 (2.006 g) was conducted on an ODS CC using MeOH–H_2O from 20:80 to 100:0, giving nineteen sub-fractions (Fr.IIB7-1–19). Three sub-fractions (Fr.IIB7-8A–C) were obtained by applying Fr.IIB7-8 (40 mg) on silica gel CC that had been eluted with CYH–EA (10:1 to 2:1). Fr.IIB7-8A (6 mg) was refined via ODC CC (MeOH–H_2O, 70:30), affording **22** (5 mg). Fr.IIB7-12 (63 mg) was separated by preparative TLC (CYH–EA, 1:5) and subjected to further purification by Sephadex LH-20 (MeOH–H_2O, 70:30) CC, yielding compounds **23** (3 mg) and **36** (4 mg). Compound **31** (62 mg) were obtained by repeating the same procedure with Fr.IIB7-14 (70 mg). Fr.IIB7-16 (40 mg) was chromatographed over preparative TLC (CYH–EA, 1:5) and purified by ODC CC (MeOH–H_2O, 70:30), yielding **30** (8 mg). Fr.IIB7-17 (26 mg) and Fr.IIB7-18 (31 mg) were processed using the same separation method, obtaining **34** (6 mg) along with **35** (10 mg), respectively. Compound **33** (9 mg) was obtained via purifying Fr.IIB7-19 (38 mg) using Sephadex LH-20 (MeOH–H_2O, 70:30) after it was chromatographed on a silica gel CC (CYH–EA, 10:1 to 1:1).

Reference

[1] Nugroho A E, Morita H. Circular dichroism calculation for natural products [J]. *J Nat Med*, 2014, 68(1): 1-10.

[2] Pescitelli G, Bruhn T. Good computational practice in the assignment of absolute configurations by TDDFT calculations of ECD Spectra [J]. *Chirality*, 2016, 28(6): 466-474.
